# Proceedings from the 11th Annual Conference on the Science of Dissemination and Implementation: Washington, DC, USA. 3-5 December 2018

**DOI:** 10.1186/s13012-019-0878-2

**Published:** 2019-03-25

## I1 Introduction

### Gila Neta^1^, David Chambers^1^, Lisa Simpson^2^

#### ^1^National Cancer Institute, Rockville, MD, USA; ^2^AcademyHealth, Washington, DC, USA

In December 2018, the National Institutes of Health (NIH) and AcademyHealth co-hosted the 11^th^ Annual Conference on the Science of Dissemination and Implementation (D&I) in Health, “Scaling up Effective Health and Healthcare: Advancing the Research Agenda and Necessary Infrastructure.” Reflecting increased interest in the field and growth in our research community, we had record attendance with nearly 1,300 researchers, practitioners and policymakers convening in Washington, DC, including a large number of attendees who had not previously taken part in the conference series. This eleventh iteration of the conference balanced its three-day research agenda among concurrent paper sessions, hundreds of posters, a keynote speaker and three plenary panels, all touching upon the conference theme of scaling up effective health and healthcare as well as building capacity and the necessary infrastructure in the field. This supplement contains the abstracts from the concurrent sessions, reflecting the variety of dissemination and implementation research supported and disseminated by our conference sponsors, including the National Institutes of Health (NIH), AcademyHealth, the Agency for Healthcare Research and Quality (AHRQ), the Patient Centered Outcomes Research Institute (PCORI), the Robert Wood Johnson Foundation (RWJF), and the US Department of Veterans Affairs (VA). Although not included in this supplement, an additional 405 papers were presented in poster format and can be viewed at https://academyhealth.confex.com/academyhealth/2018di/meetingapp.cgi/ModulePosterSessions/0.

We marked the fifth year of a partnership between the NIH and AcademyHealth in co-hosting the conference and were assisted by a multidisciplinary program planning committee which informed the plenary session development, recruited key speakers and developed the topics for workshops, one of several new elements of the conference this year. We organized concurrent sessions across ten tracks, with two to three leads per track developing the call for abstracts, and chairing review panels to select thematic sessions. We also convened a scientific advisory panel to advise on the overall conference, particularly highlighting the theme of scaling up effective health and healthcare in community, public health, and healthcare settings, with a focus on low resource settings.

We were pleased to host Dr. Amy Abernethy, then chief medical officer, chief scientific officer and senior vice president, oncology, at Flatiron Health, who delivered the keynote address “Making learning health systems a reality: Data, technology, analysis, implementation, and iteration.” Dr. Abernethy showcased the value of predictive analytics to tailor treatment, demonstrating how real-world clinical data that is typically unstructured and often disconnected across healthcare systems can be integrated in real time to promote the implementation of personalized care. Further, she highlighted the importance of meeting the needs of end-users where they are, a sentiment echoed by other plenary speakers across the three plenary panel sessions. These sessions included one focusing on scaling up effective interventions particularly in low resource settings, and two focusing on building capacity for D & I science within community and healthcare settings, respectively. Each plenary panel enabled active dialogue between presenters and with the audience, further deepening the discussion and surfacing many of the nuances and issues in D&I science. Major themes across all the plenaries included the importance of building relationships and trust among stakeholders, focusing on the end-user’s needs, working across sectors to facilitate implementation and scale up, and understanding the value of processes to inform generalizable strategies for implementation.

The thematic tracks through which concurrent sessions were organized once again included Behavioral Health, Big Data and Technology for Dissemination and Implementation Science, Clinical Care Settings (separated into two tracks: Patient-Level Interventions and System-Level Interventions), Global Dissemination and Implementation Science, Promoting Health Equity and Eliminating Disparities, Health Policy Dissemination and Implementation Science, Prevention and Public Health, and Models, Measures and Methods, and Building the Future of D & I Science: Training, Infrastructure, and Emerging Research Areas. This supplement is once again organized by these track themes.

The call for abstracts generated 787 submissions, including individual paper presentations, individual posters and panel presentations spread across the ten tracks. Over one hundred reviewers from multiple disciplines, sectors, settings and career stages devoted their time to ensuring a comprehensive review, and reviews were conducted within each track and coordinated by the track leads. For the final program, 127 papers across 32 oral abstract sessions, 41 papers across 13 panels, and 405 posters were presented over the three-day meeting, in addition to a “poster slam.” Slides for the oral presentations and panels (with the agreement of the authors) were posted on the conference website and all abstracts were included on the conference webapp. For the third year in a row, a poster slam was held, which enabled the ten top scoring posters to be presented in rapid succession, sharing key findings in five minutes each. New this year, we also hosted a Best of D&I Session which brought together five papers presented during the conference across five different tracks to highlight major lessons learned and their value to the field. We also offered poster walks with leading experts in the field and selected a Best Poster award for which the top ranked poster in each track competed. A final innovation this year, was the provision of a limited number of scholarships for patients to attend the conference as well as for participants from low and middle income countries (thanks in part to support from the Fogarty Center).

This supplement has compiled the abstracts for presented papers and panel sessions from the 11th Annual Conference on the Science of Dissemination and Implementation in Health -- Scaling up Effective Health and Healthcare: Advancing the Research Agenda and Necessary Infrastructure. We are pleased to have the combined proceedings from the conference together in one volume once again, and look forward to the 12th Annual meeting, anticipated for December 2019 in Washington, DC, which we hope will show ever increasing interest in the field and quality of the work underway.

## Behavioral Health

### S1 A randomized trial of deimplementation in response to new technology: The teachtown study

#### David Mandell^1^, Melanie Pellecchia^1^, Gwendolyn Lawson^1^, Nathaniel Williams^2^, Rinad Beidas^1^

##### ^1^University of Pennsylvania, Philadelphia, PA, USA; ^2^Boise State University, Boise, ID, USA

###### **Correspondence:** David Mandell (mandelld@upenn.edu)

**Background:** The growing number of students with autism has resulted in a proliferation of computer-assisted interventions (CAI) to increase treatment access. There is little rigorous study of how introducing this new technology affects teachers’ use of existing evidence-based practice (EBP). Teachers may, inaccurately, see CAI as replacing EBP, and therefore deimplement EBP. Conversely, they may use CAI to implement EBP with some students while others are occupied on the computer. We conducted a mixed methods study to examine the effects of introducing CAI on teachers’ use of EBP.

**Methods:** We conducted a randomized field trial of one CAI, TeachTown, and found that it was not effective in improving student outcomes. We then examined how its implementation affected teachers’ use of two one-to-one EBP and one classroom-wide EBP, visual schedules. Seventy-three classrooms were randomized to TeachTown or control. All classrooms received ongoing training and coaching in the three EBP prior to and throughout the trial. Teachers’ EBP use was measured monthly. Hierarchical models were used to test changes in EBP use over the year. Semi-structured interviews were conducted with teachers in the TeachTown condition at the end of the year, using a modified grounded theory approach, to explain the quantitative findings.

**Findings:** During the course of the school year, teachers in the control group reported significant increases in their use of one-to-one EBP (*p*’s < .05). While teachers in the TeachTown group showed higher use of these practices at baseline, their use decreased slightly during the course of the year, such that growth trajectories differed significantly between the groups (*p*’s < .05). There were no differences in use of visual schedules. In qualitative analysis, teachers revealed that they thought that TeachTown was more effective and much easier to use than one-to-one instruction, was appealing to students and parents, addressed staffing shortages, and helped teachers manage challenging behaviors.

**Implications for D&I Research:** New practices, introduced with the best intentions, can unintendedly result in deimplementation of other EBP that they were not meant to replace. Practitioners’ perceptions of intervention characteristics may trump information about effectiveness, with ease of use, consumer appeal, and ability to address urgent concerns being most important.

### S2 Implementation-focused design of an intervention targeting probationers with serious mental illnesses

#### Matthew Epperson, Leon Sawh, Emily Claypool

##### University of Chicago, Chicago, IL, USA

###### **Correspondence:** Matthew Epperson (mepperson@uchicago.edu)

**Background:** Although an estimated half million persons with serious mental illnesses (SMI) are under probation supervision in the U.S., there is not a single evidence-based intervention tailored for this population that effectively targets both mental health and criminal justice outcomes. This presentation focuses on a key “design for implementation” process necessary for the development of a treatment adherence and criminal risk intervention to be delivered directly by probation officers for probationers with SMI.

**Methods:** The design for implementation process began by convening a series of collaborative meetings with the implementation site – a specialized mental health probation unit within the Cook County (IL) Adult Probation Department. These meetings elicited probation officer tasks and roles; existing training and stated needs; examination of probation protocols and required paperwork; and officer acceptability of additional intervention concepts. Next, research staff observed intake and regular supervision sessions between probation officers and probationers with SMI, to gain a real-world sense of the context in which the intervention would be implemented. A central activity that drove the intervention design involved a series of stakeholder panels consisting of probation staff, former probationers with SMI, mental health treatment providers, advocates, and other individuals with lived experience.

**Findings:** Across the stakeholder panel meetings and alternating consultations with implementation site leadership, numerous topics were identified and discussed, including: defining key mental health and criminal justice outcomes; understanding client- and site-specific needs, strengths, and challenges; addressing comorbidities such as substance abuse and trauma; the process of client engagement in the context of probation; goal-setting and shared decision-making; criminogenic risk factors and their applicability to probationers with SMI; balancing treatment needs and supervision requirements; and expressed preferences by former clients and people with lived experience.

**Implications for D&I Research:** This iterative process provided a venue to identify critical intervention components; evaluate the acceptability, feasibility and relevance of a variety of approaches; and selection of preferred models to address mental health and criminal justice needs of the target population. This design for implementation process has resulted in an intervention that is being implemented in a real-world setting and will provide critical evidence on effectively meeting the needs of probationers with SMI.

### S3 Factors associated with US state legislators’ support for opioid use disorder parity: Implications for dissemination

#### Katherine Nelson, Jonathan Purtle

##### Drexel University Dornsife School of Public Health, Philadelphia, PA, USA

###### **Correspondence:** Katherine Nelson (Kml383@drexel.edu)

**Background:** The federal behavioral health parity law does not specify the types of services that need to be covered at parity by insurance companies. Evidence-based treatments for opioid use disorder (OUD) are often not covered at parity, but state legislators can eliminate this barrier to care by enacting comprehensive state behavioral health parity legislation (C-SBHPL) that specifies OUD coverage. The current study sought to understand how evidence about OUD treatments and C-SBHPL can be most effectively disseminated to state legislators. The study’s aim was to identity state legislator characteristics and state-level factors that predict support for OUD parity.

**Methods:** Data were from a cross-sectional, state-stratified, multi-modal (post-mail, e-mail, telephone) survey of state legislators fielded in 2017 (N=475). The dependent variable was strong support for OUD parity. Independent variables were attitudes about behavioral health treatment effectiveness, mental illness stigma, state opioid death rate, and the percent change in opioid death rate from prior year. Covariates were political party, ideology, and gender. Bivariate analyses and multivariate logistic regression were conducted.

**Findings:** Fifty-five percent of legislators strongly supported OUD parity. These legislators were significantly more likely to be female (41.1% vs 22.2%, p<.0001), Democrat (68.4% vs. 24.5%, p<.0001), have liberal ideology (62.6% vs. 17.1%, p<.0001), strongly agree that substance abuse treatments (73.6% vs. 25.4%, χ2=108.1, p<.0001) and mental health treatments (75.3% vs. 35.2%, p<.0001) were very effective, and have less mental illness stigma (p<0.0001). Higher state opioid death rate (p=.004) and higher percent change in opioid death rate (p<.0001) were associated with OUD parity support. After controlling for covariates, legislators who strongly agreed that substance abuse treatments were very effective had 4.1 higher odds of strongly supporting OUD parity (aOR=4.1, 95% CI=2.4, 7.0). The odds of OUD parity support was 0.8 lower for every one point increase in mental illness stigma score (aOR=.92, 95% CI=.85-.99).

**Implications for D&I Research:** State legislator-focused dissemination of evidence about OUD parity should include information about substance abuse treatment effectiveness and content that could reduce mental illness stigma (e.g., narratives about people affected). Dissemination strategies should consider tailoring separate materials for ideologically liberal and conservative audiences regression were conducted.

### S4 The implementation & sustainment facilitation (ISF) strategy: Effectiveness results from a 39-site cluster randomized trial

#### Bryan Garner^1^, Stephen Tueller^1^, Michael Bradshaw^1^, Steve Martino^2^, Kate Speck^3^, Denna Vandersloot^4^, Heather Gotham^5^, Michael Chaple^6^, Liz Ball^7^, Alyssa Wolfe^1^, Mat Roosa^7^, James Ford^8^

##### ^1^RTI International, Research Triangle Park, NC, USA; ^2^VA Connecticut Healthcare System, Veterans Health Administration, West Haven, CT, USA;^3^Lincoln, NE, USA; ^4^Portland, OR, USA; ^5^University of Missouri-Columbia School of Medicine, Saint Louis, MO, USA; ^6^New York, NY, USA; ^7^Syracuse, NY, USA; ^8^Madison, WI, USA

###### **Correspondence:** Bryan Garner (bgarner@rti.org)

**Background:** With substance use among individuals living with HIV/AIDS being both prevalent and problematic, improving the integration of substance use treatment within HIV service settings is needed. The Substance Abuse Treatment to HIV care (SAT2HIV) Project was funded to test an organization-focused strategy called Implementation & Sustainment Facilitation (ISF) as an adjunct to the Addiction Technology Transfer Center (ATTC) strategy. The current presentation presents effectiveness results from this cluster randomized implementation experiment.

**Methods:** Within the context of a Type 2 Effectiveness-Implementation Hybrid Trial, 39 AIDS Service Organizations (ASO) and two brief intervention (BI) staff per ASO (N = 78) were randomized to either receive (1) the ATTC strategy (ATTC only) or (2) the ATTC strategy plus the ISF strategy (ATTC+ISF). Implementation effectiveness (i.e., consistency and quality of implementation) during the 6-month implementation phase was the primary outcome measure of interest. The evidence-based treatment being implementation was a motivational interviewing-based brief intervention.

**Findings:** Adjusted multilevel regression analyses, which adjusted for several BI staff characteristics (i.e., age, gender, race, ethnicity, education, work experience, hours worked per week, motivational interviewing experience, and perceived innovation-values fit), supported the effectiveness of the ISF strategy as an effective adjunct to the ATTC strategy (β = .43, *p* = .002).

**Implications for D&I Research:** Based on findings from the current cluster randomized experiment, the ISF strategy is an effective adjunct to the ATTC’s current state-of-the-art implementation strategy. The current finding is important given that it suggests ISF as a promising strategy to improve the integration of substance use treatment within ASOs. This finding is also of importance given that the ISF intervention may hold promise for helping implement other evidence-based treatments within ASOs or for helping implement other evidence-based treatments within other settings.

### S5 An i-PARIHS evaluation of implementation fidelity for a worksite sedentary behavior intervention

#### Sarah L^1^, Sarah A Rydell^2^, Meynard JL Toledo^1^, Paul A Estabrooks^3^, Mark A Pereira^2^, Matthew P Buman^1^

##### ^1^Arizona State University, Phoenix, AZ, USA; ^2^University of Minnesota, Minneapolis, MN, USA; ^3^University of Nebraska Medical School, Omaha, NE, USA

###### **Correspondence:** Matthew P Buman (slmullan@asu.edu)

**Background:** Interventions to reduce sedentary time in the workplace have emerged as an important public health priority. *Stand&Move@Work* - a multi-component, social-ecological behavioral intervention to support sit-stand workstation use in the workplace was recently proven to be efficacious in reducing sedentary time. However, within-arm variations in the magnitude of change in sedentary time were apparent. Our purpose was to identify whether these variations were attributed to differences in implementation fidelity.

**Methods:** We evaluated 12 worksites randomized to the Stand+ arm (multi-component behavioral intervention with a sit-stand workstation). Worksite changes in sedentary behavior over 12-months were objectively measured using the activPAL3 micro accelerometer (PAL Technologies, Glasgow, Scotland). Community readiness phone interviews (n=132), employee surveys (n=2006) and quarterly “advocate” (champions of the program) surveys (n=200), were conducted. Fidelity was calculated as a percentage by triangulating these data sources to include elements of both *adherence* (15 items) and *competence* (11 items)*.* Worksites were ranked according to the magnitude of change in sedentary time. The highest 25% were categorized as the top performing worksites and the lowest 25% were categorized as the bottom performing worksites. The integrated- Promoting Action on Research Implementation in Health Services (i-PARIHS) framework was used to parse implementation differences between the top and bottom performing worksites according to innovation, context, recipients and facilitation.

**Findings:** There was a substantive difference in sedentary time over 12-months between the top and bottom performing worksites (-86.6[7.6]vs.-26.9[2.4]min/8h workday). Mean levels of adherence (75.3[6.2]vs.71.9[5.7]%), competence (60.3[8.8]vs.44.9[20.6]%) and overall fidelity (67.9[6.9]vs.58.4[13.1]%) were higher in the top performing worksites. Innovation differences indicated greater adherence to organizational (97.8[3.8]vs.86.5[9.7]%) and cultural (86.1[1.7]vs.77.9[1.2]%) strategies. Context differences indicated higher community readiness (4.3[1.9]vs.3.9[1.7]/9) and greater cultural support for ‘desk breaks’ (93.9[5.6]vs.60.4[12.4]%). Recipient (advocate) differences were aligned with facilitation. Advocates in top performing worksites were more experienced facilitators eliciting greater employee interaction (60.0[14.0]vs.32.5[3.5]%), employee knowledge (95.6[7.7]vs.80.6[27.4]%) and overall advocate quality (54.9[11.3]vs.32.4[29.1]%).

**Implications for D&I Research:** Fostering expert facilitation at the worksite level may be an important mechanism to enhance the competence of the worksite advocates and improve overall implementation fidelity without compromising generalizability of the intervention. These findings have direct implications for scaling up efficacious worksite interventions.

### S6 The effects of interventions targeting multiple health behaviors on smoking cessation outcomes: A rapid realist review

#### Nadia Minian^1^, Mathangee Lingam^1^, Tricia Corrin^1^, Wayne deRuiter^1^, Rosa Dragonetti^1^, Heather Manson^2^, Valerie Taylor^3^, Laurie Zawertailo^4^, Osnat Melamed^1^, Terri Rodak^1^, Margaret Hahn^1^, Peter Selby^4^

##### ^1^Centre for Addiction and Mental Health, Toronto, ON, Canada; ^2^Public Health Ontario, Toronto, ON, Canada; ^3^Women’s College Research Institute, Women’s College Hospital, Toronto, ON, Canada; ^4^University of Toronto, Toronto, ON, Canada

###### **Correspondence:** Nadia Minian (nadia.minian2@camh.ca)

**Background:** Multiple health risk behaviors have a synergistic negative influence on health resulting in higher rates of premature mortality and increased morbidity. Although there have been hundreds of interventions that address multiple health behaviors, much remains unknown about how to optimize these interventions. Realist synthesis is an approach of reviewing and synthesizing research evidence on complex social interventions to provide an explanatory analysis of how and why interventions work, or don’t work, in particular contexts or settings. The aim of this review was to identify behavior change mechanisms associated with effectively changing tobacco use in conjunction with targeting two or more additional risk behaviors.

**Methods:** Based on the evidence from seven large-scale multi-factorial cardiovascular disease and cancer risk factor interventions reviewed by our expert panel we generated our initial program theory. To test this initial program theory, a systematic literature search was conducted in several bibliographic databases. Two reviewers screened titles and abstracts for relevant research, and the selected full papers were used to extract data (context, mechanisms and outcomes) and assess the quality of evidence. Mechanisms were categorized using Michie et al.’s COM-B system - a framework for understanding behavior; where capability, opportunity, and motivation interact to generate behavior.

**Findings:** Based on predetermined inclusion and exclusion criteria, 195 articles were included in the review. We present our findings by the three categories used to identify the mechanisms of change: capability, opportunity, and motivation. 1) Universally, increasing opportunities for participants to engage in healthy behaviours (by providing free medication, gym memberships, increasing smoke free places, providing social support) was successful in helping people quit smoking. 2) In North America, interventions that target primary prevention and that aim to increase participant capabilities (such as capacity to plan, and enhanced knowledge) help people quit smoking. 3) Interventions that had a multidisciplinary team and had a motivational component, helped individuals to quit smoking.

**Implications for D&I Research:** By emphasizing underlying mechanisms, the results of this realist synthesis can contribute to the development of evidence-based and effective interventions, in conjunction with other established methods.

### S7 Adaptation of an implementation strategy and a psychosocial intervention for va's supportive housing program: A mixed-methods realist evaluation

#### Vera Yakovchenko^1^, Sonya Gabrielian^2^, Jeffrey Smith^3^, Kathryn Bruzios^1^, David Smelson^1,4^, Megan McCullough^1^

##### ^1^BridgeQUERI, Veterans Health Administration, Bedford, MA, USA; ^2^Veterans Health Administration, Los Angeles, CA, USA; ^3^Central Arkansas Veterans Healthcare System (North Little Rock), Veterans Health Administration, North Little Rock, AR, USA; ^4^University of Massachusetts Medical School, Worcester, MA, USA

###### **Correspondence:** Vera Yakovchenko (vera.yakovchenko@va.gov)

**Background:** Maintaining Independence and Sobriety through Systems Integration, Outreach, and Networking- (MISSION) is an evidence-based intervention that uses a case manager and peer specialist dyad to deliver psychosocial treatment for homeless Veterans with co-occurring mental health and substance use disorders. A stepped wedge study comparing MISSION with usual implementation versus implementation facilitation is being conducted in two Department of Veterans Affairs facilities (3 sites with 7 total locations). Given the highly flexible and context-driven implementation of MISSION, we adapted and evaluated the clinical intervention (MISSION) and the implementation strategy (facilitation) in real-time.

**Methods:** We conducted a mixed-methods realist evaluation guided by the Consolidated Framework for Implementation Research (CFIR). An organizational readiness to change and implementation climate survey was administered to staff trained in MISSION (n=70). Semi-structured qualitative interviews with a purposive subset of clinical staff (n=37) and study team members (n=5) were conducted. Facilitation activities were systematically logged on a tracking sheet.

**Findings:** Qualitative interviews identified key decision elements for adaptation in implementation, mapped to CFIR constructs: 1) intervention: staff were frequently discouraged by MISSION’s complexity and perceived lack of relative advantage; 2) outer setting: national policy and patient needs were aligned with MISSION goals; 3) inner setting: organizational infrastructure (leadership, staffing, resources) could not always accommodate implementation and staff felt unprepared to implement because training was not commensurate with expectations; 4) individual: frontline staff did not feel empowered to implement; and 5) process: there was insufficient engagement of leadership and staff to ensure implementation occurred as intended. There were significant site and staff variation on culture and climate items of the organizational survey.

**Implications for D&I Research:** While not ideal, concurrent adaptation and implementation happens in real-world interventions. Despite considerable effort to support implementation through facilitation, uptake has been modest due to complexities of the intervention and site context. We identified a series of decision points that could have aided adoption, but did not because they did not occur during pre-implementation. To address the barriers faced, we developed a pre-implementation guide and tool to support future intervention rollout and ensure that fidelity was maintained while adaptations are made to fit the context.

### S8 Defining and operationalizing the collaborative chronic care model: Development of a framework for assessing a hybrid II controlled trial of team-based mental health care implementation

#### Bo Kim^1^, Christopher Miller^1^, Jennifer Sullivan^2^, Rani Elwy^1^, Karen Drummond^3^, Samantha Connolly^4^, Rachel Riendeau^1^, Mark Bauer^1^

##### ^1^VA Boston Healthcare System, Veterans Health Administration, Boston, MA, USA; ^2^Boston University School of Public Health, Boston, MA, USA; ^3^Central Arkansas Veterans Healthcare System, Little Rock, AR, USA; ^4^Harvard Medical School, Boston, MA, USA

###### **Correspondence:** Bo Kim (bo.kim@va.gov)

**Background:** The evidence-based Collaborative Chronic Care Model (CCM) is being increasingly implemented for mental healthcare. Evaluating CCM implementation requires assessing the extent to which CCM elements – work role redesign, patient self-management support, provider decision support, clinical information systems, linkages to community resources, and organizational/leadership support – are implemented. This is challenging, since the elements are guiding concepts not predefined care processes. We therefore aimed to develop operational definitions and clinical examples of CCM elements, with a detailed rating scheme, to be used for assessing the extent of CCM implementation based on qualitative interviews.

**Methods:** Guided by the Centers for Disease Control and Prevention’s Codebook Development Methodology for Team-Based Qualitative Analysis, we developed a codebook defining each element based on systematically-reviewed CCM literature. We added care process examples for each element, based on 18 interviews with mental health clinicians at three Veterans Health Administration (VA) medical centers, purposively selected for their varied extent of CCM implementation. We developed a 1-5 rating scheme that differentiated the three sites on how stably and broadly each element is established. We applied the codebook and rating scheme toward assessing nine VA sites in a subsequent hybrid II controlled trial of CCM implementation.

**Findings:** Our codebook specified (i) the general extent of elements expected at all VA sites (i.e., without CCM implementation), (ii) examples of elements to look for at the trial sites, and (iii) “close but no” specifications of care processes that seemingly exhibit, but not qualify, as an indication of successful implementation. Applying the codebook and rating scheme to analyzing 27 interviews across the nine trial sites enabled reliable differentiation of the sites into those with higher (n=4) and lower (n=5) extents of CCM implementation.

**Implications for D&I Research:** Our codebook and rating scheme provide criteria for assessing the extent of CCM implementation, based on real-world examples of care processes that do or do not embody CCM elements. Future work should explore their (i) reliability in assessing CCM implementation outside VA and (ii) applicability to data beyond interviews. Our approach for their development serves as a guide for other evidence-based care models that need assessments of their extent of implementation.

### S9 Assessing collaborative care in mental health teams: Qualitative analysis to guide future implementation

#### Christopher Miller^1,2^, Jennifer Sullivan^1^, Bo Kim^1^, Rani Elwy^3^, Karen Drummond^4^, Samantha Connolly^2^, Rachel Riendeau^1^, Mark Bauer^1^

##### ^1^VA Boston Healthcare System, Veterans Health Administration, Boston, MA, USA; ^2^Harvard Medical School, Boston, MA, USA; ^3^Boston University School of Public Health, Boston, MA, USA; ^4^Central Arkansas Veterans Healthcare System, Little Rock, AR, USA

###### **Correspondence:** Christopher Miller (Christopher.Miller8@va.gov)

**Background:** The Collaborative Chronic Care Model (CCM) is a flexible, evidence-based approach to organizing care for chronic health conditions. There are six CCM elements: workrole redesign, patient self-management support, provider decision support, clinical information systems, linkages to community resources, and leadership support. To inform efforts to implement CCM-based mental health care, it is crucial to better understand how clinicians pursue the CCM elements in real-world clinical settings.

**Methods:** We conducted semi-structured qualitative interviews with members of 10 VA outpatient mental health teams across the country (N = 32 staff) at the outset of a hybrid type II implementation/effectiveness trial. We analyzed data using directed content analysis, based on the six CCM elements. Our goal was to identify provider views of these elements, and to assess the extent to which care was seen as consistent, or inconsistent, with the elements themselves. To our knowledge this is the first study of the CCM to use in-depth, one-on-one qualitative interviews.

**Findings:** Few participants were familiar with the CCM by name, but many reported delivering care that was consistent with one or more of its elements. Common examples of this included the application of evidence-based psychotherapies (CCM elements: provider decision support and patient self-management support), as well as active follow-up after patient no-shows (CCM element: work role redesign). Other CCM elements appeared to be pursued less consistently. For example, few participants reported having access to a registry for their team, complicating efforts to track treatment progress across patients (CCM element: clinical information systems). Overall, endorsements of CCM-consistent care were frequently ad hoc rather than systematic.

**Implications for D&I Research:** In this geographically diverse sample of VA-based outpatient mental health staff, there was variety in the extent to which care was aligned with the evidence-based elements of the CCM. Some of the CCM elements (e.g. provider decision support and patient self-management support) were endorsed much more consistently than others. While the evidence base for the CCM is strong, implementing some CCM elements in outpatient mental health care will be more challenging than others. These findings underscore the importance of understanding providers’ views of the CCM before implementation efforts begin.

### S10 Sustaining depression collaborative care in academic primary care settings across New York State: Implementation metrics key to sustainability

#### Nathalie Moise^1^, Ravi Shah^2^, Susan Essock^2^, Amy Jones^3^, Danielle Chapman^3^, Jay Caruthers^3^, Lloyd Sederer^3^, Lauren Peccoralo^4^

##### ^1^Medicine, Columbia University Medical Center, New York, NY, USA; ^2^Psychiatry, Columbia University, New York, NY, USA; ^3^New York State, Office of Mental Health, New York, NY, USA; ^4^Medicine, Mount Sinai School of Medicine, New York, NY, USA

###### **Correspondence:** Nathalie Moise (nm2562@cumc.columbia.edu)

**Background:** In one of the largest state-wide initiatives of its kind, New York State implemented collaborative care (CC) in 32 academic primary care clinics from 2012-2014. It then supported its sustainability through payment reforms from 2015 onwards in 26 of the 32 clinics opting to sustain CC. Few studies have analyzed CC sustainability efforts. We aimed to assess whether clinics opting in (vs. out) of sustainability differed in key implementation fidelity measures.

**Methods:** Clinics reported metrics quarterly: 1) depression screening rates/calendar year; 2) care manager full time equivalents (FTEs); 3) percent of clinic population screened for depression/calendar year; 4) number of patients currently enrolled in CC program; 5) percent of screen positive patients enrolled in CC 6) percent of patients enrolled ≥16 weeks with a Patient Health Questionnaire-9 <10 and 7) percent of enrolled individuals with a psychiatric consult per quarter. We used descriptive statistics to assess differences in metrics at the end of implementation between clinics that opted in (n=26) vs. out (n=6) of the sustainability program.

**Findings:** At the end of the 2 year implementation period, clinics opting in (vs. out) of a CC sustainability program had higher median care manager FTEs (1.0 vs. 0.50, p=0.002) and clinical improvement rates (46% vs. 7.5%, p=0.004), but we found no difference in depression screening rates (97% vs. 87%, p=0.51), CC program enrollment rates (43% vs. 34%, p=0.22), enrolled patients with psychiatric consultations (100% vs. 90%, p=0.53) or enrolled (128 vs. 113, p=0.75) and depressed (275 vs. 334, p=0.39) patients per care manager FTE.

**Implications for D&I Research:** In one of the largest state CC initiatives to-date, New York State successfully advanced behavioral integration in primary care. We found that early investment in care manager FTEs and ability to meet key outcome metrics (e.g., improvement rates) may be key to CC sustainability. Future research should focus on identifying implementation fidelity measures that predict CC sustainability.

### S11 A hybrid II randomized stepped wedge trial to implement the collaborative chronic care model in VA mental health clinics

#### Mark Bauer^1^, Christopher Miller^2^, Bo Kim^1^, Robert Lew^1^, Kelly Stolzmann^1^, Jennifer Sullivan^3^, Rachel Riendeau^1^, Samantha Connolly^4^, Rani Elwy^1^, Kendra Weaver^5^

##### ^1^VA Boston Healthcare System, Veterans Health Administration, Boston, MA, USA; ^2^Harvard Medical School, Boston, MA, USA; ^3^Boston University School of Public Health, Boston, MA, USA; ^4^Harvard Medical School, Boston, MA, USA; ^5^Office of Mental Health and Suicide Prevention, Department of Veterans Affairs, Mountain Home, TN, USA

###### **Correspondence:** Mark Bauer (mark.bauer@va.gov)

**Background**: Collaborative Chronic Care Models (**CCMs**) have extensive evidence for effectiveness in improving outcomes in a variety of health conditions, including serious mental illnesses treated in mental health clinics. However, data come predominantly from randomized controlled trials. There is little evidence regarding CCM impact on mental health conditions when implemented in typical practice conditions, and almost all such studies are limited to assessment of primary care depression treatment.

**Methods**: We conducted a randomized stepped wedge trial to implement a CCM for a mixed-diagnosis population treated in mental health clinics in the US Department of Veterans Affairs. We hypothesized that implementation support would be associated with evidence of CCM implementation and improvement in Veteran health status. Implementation support consisted of blended facilitation, combining a study-funded external facilitator with a facility-funded internal facilitator working with a designated provider team. The external facilitator conducted a site evaluation remotely followed by a 1.5-day face-to-face site visit. S/he then worked with the internal facilitator and team via videoconference and telephone for twelve months. Nine sites received facilitation, with start-time randomized across three waves of three sites each balanced on key site characteristics. Using a hybrid II design we assessed both implementation outcomes (team function, CCM-concordant processes) and intervention outcomes (Veteran health status and hospitalization rates). Data sources included Veteran interviews, provider surveys, and administrative data. The primary health outcome variable was the Mental Component Score (MCS) of the VR-12. Data were analyzed using mixed effects models for continuous and binary data.

**Findings**: For implementation outcomes, blended facilitation was associated with significant improvements in provider-rated team function. Facilities varied in percentage of CCM-concordant processes achieved (44-89%) by the end of facilitation. For intervention outcomes, facilitation was associated with a robust reduction in mental health hospitalization rate. MCS did not change over time.

**Implications for D&I Research**: Blended facilitation led to improved team function plus variable CCM process improvement, which were associated with heterogeneous impact on Veteran-level outcomes. The next challenge for this and other implementation trials is to “look beyond the mean” to understand inter-site heterogeneity and link specific provider and system changes to clinical improvements.

### S12 Implementation of measurement-based care: Creation of an implementation planning guide

#### Katherine Dollar^1^, JoAnn Kirchner^2^, Pearl McGee-Vincent^3^, Dominick DePhilippis^4^, Sandra Resnick^5^

##### ^1^Center for Integrated Healthcare, Veterans Health Administration, Syracuse, NY, USA; ^2^Central Arkansas Veterans Healthcare System (North Little Rock), Veterans Health Administration, North Little Rock, AR, USA; ^3^National Center for PTSD, Veterans Health Administration, Menlo Park, CA, USA; ^4^CeSATE, Veterans Health Administration, Philadelphia, PA, USA; ^5^NEPEC, Veterans Health Administration, West Haven, CT, USA

###### **Correspondence:** Katherine Dollar (katherine.dollar@va.gov)

**Background:** Measurement Based Care (MBC) is the systematic process of collecting patient self-report data and using that information to monitor treatment progress, and inform shared clinical treatment decisions over time. The Department of Veterans Affairs started a national initiative to implement MBC in mental health (MH) in 2016. Subsequently, beginning January 1, 2018, The Joint Commission (TJC) revised their standards to require MBC implementation. There are multiple, well-documented benefits of MBC in mental health (MH) services, including improved outcomes and quality. However, significant gaps remain in implementation of MBC. To address these gaps, an Implementation Planning Guide was developed.

**Methods:** Semi-structured qualitative interviews, developed to capture barriers, facilitators, and steps for successful MBC implementation, were conducted with eight MBC leaders at six VHA facilities. After conducting these key informant interviews, researchers identified recurring themes across interviews and implementation steps. These findings were translated into actionable items and informed the development of the implementation planning guide.

**Findings:** Qualitative themes include the need for a collaborative process that ensures active participation from front line clinicians and leadership, the importance of communicating vision, need for thoughtful measure selection that ensures use of targeted (clinically actionable) measures, provision of sufficient training, the importance of local champions, and the value of protected time for those implementing MBC. These themes and action steps were then used to inform the development of the planning guide, designed to assist programs making key decisions for successful MBC implementation.

**Implications for D&I Research:** The MBC implementation planning guide provides a step-by-step sequence of key decisions for consideration to support successful implementation of MBC within the context of on-going local qualitative improvement process. This MBC implementation planning guide has applicability across settings to support implementation of MBC consistent with TJC policy requirements. This Planning Guide not only enhances local MBC implementation, but provides a structure for future research focusing on dissemination and implementation of MBC.

### S13 Influencers of staff perceptions of organizational sustainability capacity in a quality improvement collaborative

#### James Ford, Aaron Gilson

##### School of Pharmacy, University of Wisconsin-Madison, Madison, WI, USA

###### **Correspondence:** James Ford (jhfordii@wisc.edu)

**Background:** An organization’s sustainability capacity (SC) – the ability to implement and maintain change – is influenced by internal attributes, environmental contextual influencers, and intervention attributes. Dissemination and implementation research has generally not explored changes over time in staff perceptions about SC, and how quality improvement collaborative (QIC) participation influences those changes. This project addresses this gap, using the British National Health Services sustainability index to measure staff SC perceptions at four time points (baseline and every 9 months) for participants of the NIATx200 initiative, a QIC involving 201 substance use providers.

**Methods:** Respondent demographic information (Organization ID, Job Function, First Initial, Day of Birth, Employment Status) allowed matching individual survey responses, so all analyses represent responses from the same staff members (n=908, representing 2,329 total cases) across the evaluation timeframe. A mixed linear model repeated measures analysis fit three separate statistical models to assess potential predictors of SC perceptions: Time (Models I-III); NIATx200 intervention, staff job function, and tenure (Models II &III); and low versus high NIATx200participation (Model III).

**Findings:** Model I yielded strong overall predictive significance for Time (F=9.367, p<.0001), with staff perceptions of SC increasing throughout most of the study (t_4,1_=4.488, p<.0001; t_4,2_=2.100, p<.036; t_4,3_=0.157, p=ns). Model II did not change the overall Time effect from Model I. However, the assigned NIATx200 intervention (t=2.578, p<.010) and staff job function (t=3.096, p<.002) were significant; that is, combined services and organization administrators were associated with greater perceptions of SC. The addition of participation levels in Model III demonstrated the importance of high participation (t=2.506, p<.013), but led to non-significance for all other model variables.

**Implications for D&I Research:** Although there was minimal staff exposure to sustainability principals within NIATx200, staff perceptions about their organization’s sustainability capacity (SC) significantly differed over time. However, an organization’s participation level in a QIC became the principal predictor of staff SC perceptions, regardless of other factors. Given these findings, it is possible to develop and introduce specific sustainability content within the structure of a QIC as a means to assess the impact on staff SC perceptions over time and the sustainment of organizational change.

### S14 Identifying supervisory strategies to improve provider adoption of person-centered care planning in behavioral health services: A mixed methods study

#### Mimi Choy-Brown^1^, Victoria Stanhope^2^, Deborah Padgett^2^

##### ^1^School of Social Work, University of Minnesota, St. Paul, MN, USA; ^2^Silver School of Social Work, New York University, New York, NY, USA

###### **Correspondence:** Mimi Choy-Brown (mimichoybrown@gmail.com)

**Background:** Leadership has been identified as an important predictor for provider adoption of evidence-based practices (EBP), but less is understood about mechanisms between providers and their direct supervisors that improve EBP adoption. Utilizing Normalization Process Theory (NPT) as a sensitizing theory, this study examined supervisory strategies to embed person-centered care planning into provider practice in the context of a multi-state implementation effort in behavioral health settings.

**Methods:** Embedded within a large-scale randomized controlled trial of person-centered care planning (PCCP), this sequential mixed methods study used quantitative data from provider surveys to sample supervisors with maximum variation on their implementation leadership for the qualitative phase. In-depth qualitative interviews (*N*= 34) with both supervisors (*N*= 12) and their supervisees (*N*= 22) were triangulated with direct observation of each supervisor. Interviews were transcribed and co-coded in Atlas.ti by two researchers with supervisory experience. Analyses employed a modified grounded theory approach and constant comparative analyses. Multi-perspective data triangulation identified convergence and divergence within these data and enriched findings. Strategies for rigor (e.g., memos and prolonged engagement) were employed throughout data collection and analyses.

**Findings:** Three supervisory strategies to improve provider adoption were identified: chipping away, practicing together, and knowing your audience. Chipping away encompassed supervisors’ efforts to make sense of PCCP within their local context and to keep infusing reminders for providers to change their practice. Critical to supervisors’ capacity to engage and motivate providers in practice change was their attunement to providers (knowing their audience) and their active engagement with PCCP and supervisees (practicing together). These strategies improved accurate calibration of their interactions to the dynamic contextual and individual needs during the implementation process and communicated supervisory support of PCCP.

**Implications for D&I Research:** Findings contribute practice-based knowledge from a critical segment of the behavioral health workforce about how interactions between providers and their direct supervisors support an implementation process. In particular, supervisors were key PCCP champions utilizing both learning and social processes to support provider PCCP adoption consistent with NPT. Targeting these supervisory activities holds promise as an effective implementation strategy to build provider buy-in and adoption.

### S15 How implementable is that evidence-based practice? a methodology for assessing complex innovation usability

#### Aaron Lyon^1^, Julie Chung^2^, Kelly Koerner^2^

##### ^1^University of Washington, Seattle, WA, USA; ^2^Evidence-Based Practice Institute, Seattle, WA, USA

###### **Correspondence:** Aaron Lyon (lyona@uw.edu)

**Background:** Innovation-level determinants (e.g., design quality, complexity) are critical to implementation, but little research has focused on assessing and addressing barriers at this level. A recent systematic review of implementation measures found few instruments that addressed the innovation level, and none that addressed design quality and packaging (Lewis et al., 2015). Nevertheless, usability – the extent to which a product can be used by specified users to achieve specified goals with effectiveness, efficiency, and satisfaction – is a key “upstream” determinant of implementation and service outcomes. Drawing from the field of user-centered design, this session will present data from a novel and pragmatic methodology for evaluating the usability of complex psychosocial interventions in behavioral health.

**Methods:** The Usability Evaluation for Evidence-Based Psychosocial Interventions (USE-EBPI) methodology comprises four steps: (1) identify users; (2) define EBPI components; (3) plan and conduct the evaluation; and (4) organize usability issues within the User Action Framework (UAF). USE-EBPI was applied to evaluate exposure procedures for treating anxiety. Stratified sampling identified clinicians (*n*=10) with differing levels of experience with exposure (beginner, *n*=3; intermediate, *n*=4; advanced, *n*=3). EBPI components prioritized for testing included in-session exposure procedures, subjective distress ratings, and a written exposure guide. Clinicians participated in web-facilitated usability testing sessions during which they reviewed materials using “think aloud” methods, applied exposure procedures in a behavioral rehearsal, and rated the procedures with the Intervention Usability Scale (IUS).

**Findings:** The average IUS rating was 78.61 (possible range: 10-100), indicating acceptable usability, but also room for improvement, based on norms. Ratings for beginner and intermediate participants were comparable (77.50), with advanced users providing somewhat higher ratings (82.50). Usability issues identified included problematic exposure guide information design that was non-intuitive or did not directly facilitate decision-making, a need for improved error detection supports, and inadequate homework support/specificity. Most usability issues were reflected the Translation (translating plans into actions) or Actions (successfully performing tasks) domains of the UAF.

**Implications for D&I Research:** Innovation-level determinants are understudied in implementation science. Systematic methods for assessing innovation usability have the potential to (1) predict subsequent implementation outcomes and (2) drive contextually-appropriate innovation redesign to improve adoption and sustainment.

### S16 The sustained program of alcohol-related care (SPARC) trial: Simultaneous implementation of behavioral health integration to improve the relative value of the innovation

#### Katharine Bradley^1^, Amy Lee^1^, Carol Achtmeyer^2^, Evette Ludman^1^, Julie Richards^1^, Paula Lozano^1^, Emily Williams^3^, Gwen Lapham^1^, Jennifer Bobb^1^, Joseph Glass^1^, Rebecca Parrish^4^, Ryan Caldeiro^4^

##### ^1^Kaiser Permanente Washington Health Research Institute, Seattle, WA, USA; ^2^Veterans Affairs Puget Sound, Veterans Health Administration, Seattle, WA, USA; ^3^University of Washington, Department of Health Services, Seattle, WA, USA; ^4^Behavioral Health Services, Kaiser Permanente Washington, Seattle, WA, USA

###### **Correspondence:** Katharine Bradley (katharine.a.bradley@kp.org)

**Background:** Implementation researchers often use multicomponent interventions when seeking to improve primary care (PC) for specific conditions. These interventions require operations-research partnerships, but the target condition (e.g. unhealthy alcohol use) may not be a priority for clinical leaders. Simultaneously implementing innovations that are a priority for leaders (“Co-innovations”) could help. This presentation describes how a pragmatic trial focused on improving alcohol-related care implemented Behavioral Health Integration (BHI) as a co-innovation. We describe results of quality improvement metrics for BHI as well as alcohol-related care.

**Methods:** SPARC is a stepped-wedge trial to implement evidence-based prevention and treatment of unhealthy alcohol use in PC that ends 7/31/2018 (NCT02675777). After a 3-clinic pilot, all remaining PC clinics of Kaiser Permanente Washington (n=22) were randomized to 7 waves (4 months apart) beginning 4/2016. The intervention, designed based on Greenhalgh’s model, included enhanced practice coaching, EHR support, and performance feedback. Operations partners requested that the intervention also support care for depression, cannabis and other drugs (BHI). SPARC therefore implemented BHI screening (PHQ-2; AUDIT-C; single-item cannabis and drug screens) and follow-up assessments (PHQ-9; suicide risk]; DSM-5 Symptom Checklists for alcohol or substance use disorders [AUD/SUD]. Performance metrics include mean clinic: % of PC patients screened and % assessed among those with high-risk screening results.

**Findings:** No clinic had routine screening or assessment prior to the trial. In May 2018 (0-20 months after active implementation ended) screening metrics were: depression, 89%; unhealthy alcohol use, 88%; cannabis, 88%; and drugs, 88%. Assessment metrics were: depression 95%; suicide risk, 86%, AUD, 69%, and SUD 63%. Clinics initially expressed multiple concerns about implementing BHI; positive local patient stories, often unrelated to alcohol, helped PC teams recognize the value of BHI (e.g. unexpected identification of acutely suicidal patients).

**Implications for D&I Research:** SPARC was designed based on Greenhalgh’s Model which highlights the importance of features of the innovation and linkage between change agents and delivery systems. While “fuzzy boundaries” of innovations are often adapted, we know of no prior pragmatic trial in which additional “co-innovations”—in this case BHI—have been implemented. In this trial, implementation of BHI seemed to markedly improve implementation and sustainment of alcohol-related care.

### S17 Statistical innovations in pragmatic trials of health-system implementation interventions

#### Joseph Glass, Jennifer Bobb, Gwen Lapham, Katharine Bradley

##### Kaiser Permanente Washington Health Research Institute, Seattle, WA, USA

###### **Correspondence:** Joseph Glass (Joseph.E.Glass@kp.org)

**Background:** Pragmatic implementation trials need to maintain rigorous trial design and analysis, while simultaneously addressing health system needs. SPARC is a stepped-wedge pragmatic trial of a novel intervention to implement evidence-based care for unhealthy alcohol use (UAU) in primary care (PC). This presentation addresses two statistical issues in the design of SPARC: (1) the potential for identification bias (a form of selection bias that can occur when the interventions being compared differentially affect identification of the sample across study arms); and (2) response to requests from the health system for specific constraints on randomization.

**Methods:** SPARC is testing strategies to implement evidence-based alcohol-related care—including alcohol screening and follow-up interventions. This abstract analyzed data from 3 pilot clinics. (1) Implementation “reach” should be assessed with a known denominator, which is often a screen-positive population per electronic health records in pragmatic trials. Because the intervention intended to increase screening (and could thus change the denominator), we investigated the potential for identification bias. Analyses evaluated whether the proportion of patients who screened positive for UAU and high-risk UAU differed in the post- versus pre-implementation periods. (2) Health system leaders asked to select 9 clinics that would implement the intervention year 1, as well as for additional constraints year 2. We developed an innovative randomization scheme within these constraints to maintain equivalent randomization probabilities by year.

**Findings:** (1) Data from pilot sites suggested that identification bias was indeed a concern: pre-implementation, 35% of 7,868 screens were positive (5.3% high risk) versus 24% of 69,926 positive post-implementation (2.2% high risk). This suggests that the screen-positive population changed from before to after the intervention. (2) To address constraints placed on randomization, a stratified randomization scheme was developed in which health system leaders identified clinics that would be randomized year 1 of the study (waves 1-3), with the remaining sites randomized year 2 (waves 4-7), with an equivalent probability of each clinic being assigned to each wave within each year.

**Implications for D&I Research:** Trial design in partnered research is enhanced through statistical innovations, including tailored randomization schemes and selection of trial denominators to mitigate the potential for identification bias.

### S18 The sustained program of alcohol-related care (SPARC) trial’s use of enhanced practice coaching to implement and sustain alcohol-related care in primary care

#### Amy Lee^1^, Carol Achtmeyer^2^, Evette Ludman^1^, Julie Richards^1^, Katharine Bradley^1^

##### ^1^Kaiser Permanente Washington Health Research Institute, Seattle, WA, USA; ^2^Veterans Affairs Puget Sound, Veterans Health Administration, Seattle, WA, USA

###### **Correspondence:** Amy Lee (Amy.K.Lee@kp.org)

**Background:** Practice coaches are effective for supporting quality improvement in primary care (PC). Typically, PC teams possess the clinical expertise, and the coach’s role is to help teams implement improvement processes to apply that expertise. But what if PC teams do not have the clinical knowledge needed to improve care? This presentation describes an expanded model of enhanced practice coaching used in the SPARC trial. Findings are presented for the first 9 clinics randomized in the trial “Year 1.”

**Methods:** SPARC is a stepped-wedge pragmatic trial to implement evidence-based care for unhealthy alcohol use in 22 PC clinics. Before SPARC, 19% of PC patients had alcohol screening and 0% had standardized assessment for alcohol use disorder (AUD) in PC. The intervention had three components: front-line PC support by practice coaches, electronic health record (EHR) tools, and performance feedback. Practice coaches met weekly with implementation teams from each clinic for up to 6 months for quality improvement (“active implementation”). Practice coaches also addressed clinical knowledge gaps, including modeling destigmatizing language, and collaborated with operations partners on EHR tools and performance metrics. We report findings on performance metrics for 9 clinics that completed active implementation Year 1 (by 5/2017), and one year later (5/2018) reflecting sustainment. Measures are: % with alcohol screening, among patients seen in PC; and % completing a standardized tool assessing DSM-5 AUD symptoms, among patients with high-risk alcohol screening scores.

**Findings:** Practice coaches met with teams 15 times on average (range: 9-19). Practice coach collaboration with programmers helped enhance EHR tools and corrected EHR and metric problems identified by teams. Following active implementation, operations partners continued quarterly quality improvement meetings with PC teams (without coaches). After SPARC implementation, alcohol screening rates were 88% in 5/2017 and 87% one year later. Standardized assessment rates for AUD were 72% of high-risk patients in 5/2017, and 64% one year later. Based on sustainment, and staff and leader satisfaction, this implementation model has become a “gold standard” for this health system.

**Implications for D&I Research:** An enhanced practice coach model can create linkages between different elements of multi-component interventions and with operations partners, leading to sustained implementation in PC.

## Big Data and Technology for Dissemination & Implementation Science

### S19 Development and implementation of a novel technology-enabled care coordination model to address social determinants of health for senior patients

#### Jessa Engelberg^1^, Andrea Morris^1^, Florence St-Onge^2^, Sara Pashaee^2^, Sonia Seghal^2^, Giovanni Corzo^3^, Dara Sorkin^2^, Dana Mukamel^2^, Lisa Gibbs^2^

##### ^1^West Health Institute, La Jolla, CA, USA; ^2^University of California, Irvine, Orange, CA, USA; ^3^Home and Care Services, SeniorServ, Anaheim, CA, USA

###### **Correspondence:** Jessa Engelberg (jengelberg@westhealth.org)

**Background**: It is widely acknowledged that traditional approaches to healthcare are often siloed and do not consider the social determinants of health (SDOH), despite the outsized impact on well-being, health and costs. As part of a multi-phase collaborative research study, the Gary and Mary West Health Institute (WHI), the University of California, Irvine Senior Health Center (SHC), and SeniorServ developed and implemented an innovative, technology-enabled care coordination model (i.e. 360° Caregiving Solution) to address the full range of needs among senior patients, including SDOH.

**Methods**: In Phase 1, a formative evaluation was conducted at the SHC and SeniorServ to understand staff perspectives, current processes and opportunities to identify and address seniors’ SDOH-related needs. Findings informed Phase 2 efforts, which included the development of a senior-specific SDOH-screener, new workflows to identify and address SDOH-needs within the SHC, and a customized electronic care coordination platform with research, clinical and community-based partner input. A community-based social worker (i.e. care navigator) was embedded within the SHC to administer the SDOH-screener, assess unmet needs, and connect patients to community-based supports. The electronic platform was designed to facilitate the identification of community-based services, streamline service referrals and enable data-sharing across clinical and community settings. Initial feasibility and user testing of the SDOH-screener, SHC workflows and electronic platform was conducted using the Plan-Do-Study-Act (PDSA) framework.

**Findings**: Phase 1 findings showed SDOH-related needs were informally identified in both settings, but there was a lack of standardized processes and minimal communication across settings. During Phase 2, the SDOH-screener was used to identify at-risk patients (about 10% screened positive), refine the care navigator’s workflow, and establish that the electronic platform could successfully facilitate the receipt of and response to referrals across settings.

**Implications for D&I Research:** The ongoing collaboration with multi-disciplinary partners and assessment of processes and factors led to the development of the 360° Caregiving Solution, designed to identify and address SDOH-related needs for senior patients. It is now being piloted with all SHC patients during Phase 3. As a pragmatic trial, it includes ongoing collection and analysis of process measures to ensure successful implementation, as well as outcome measures to assess impact.

### S20 Using spatial analyses to inform implementation activities

#### Mary Bollinger^1,2^, Sara Landes^2^

##### ^1^Central Arkansas Veterans Healthcare System, Veterans Health Administration, North Little Rock, AR, USA; ^2^University of Arkansas for Medical Sciences, Little Rock, AR, USA

###### **Correspondence:** Mary Bollinger (mjbollinger@uams.edu)

**Background:** When implementing evidence-based practices with limited resources at a broad level, such as across a state, region, or large multi-site healthcare system, it can be difficult to identify where to implement first. Community-level data can be used to identify initial implementation sites and then to understand any variation in the use of an evidence-based practice once scaled up fully. Though not broadly used by implementation scientists, spatial modeling can provide community context data to inform scale up of EBPs by identifying where to target interventions as well as evaluation activities by identifying geographies with uneven uptake. Using suicide as an exemplar, we show how sites could be selected for the implementation of suicide prevention program.

**Methods:** Suicide data for 2014-2016 by county was acquired from the Arkansas Department of Health as was county-level opioid prescribing rates. The 2017 County Health Rankings provided data on the county-level measures used in our analyses. These measures included whether the county was rural, the proportion of the population in deep poverty, the proportion of high school graduates, the rate of firearm deaths. Geographic/map data was obtained from the US Census Bureau. County-specific rates were estimated using a novel Bayesian disease mapping method, the Integrated Nested Laplace (INLA) approximation within the R program for Statistical Computing. Results of the INLA procedure were mapped using ARCGIS 10.5

**Findings:** We identified 2 clusters of counties with higher than average suicide rates once we controlled for the geographic relationship between counties. The first cluster was located in the northwest corner of the state and included 11 counties. The second cluster occurred in the southwest edge of the state and included 5 counties. Initial suicide outreach activities would be strengthened by targeting the clusters of counties identified in the spatial analyses.

**Implications for D&I Research:** Implementation of EBPs could more efficiently be accomplished by knowing where initial investments could be made to address population needs. Spatial analysis is particularly helpful identifying ‘hot spots’ where services/programs are critically needed and can provide insight into the factors involved in diffusion and implementation.

### S21 Visualization of the reach of an intervention: Use of geographic information systems (GIS) in implementation research

#### Lexus Ujano-De Motta^1,2^, Chelsea Leonard^3^, Brandi Lippmann^1^, Lynette Kelley^1^, Marina McCreight^1^, Ashlea Mayberry^1^, Andrew Coy^1^, Robert Burke^4,5^, Heather Gilmartin^2^

##### ^1^VA Eastern Colorado Health Care System, Veterans Health Administration, Aurora, CO, USA; ^2^Denver-Seattle Center of Innovation, Veterans Health Administration, Denver, CO, USA; ^3^VA Eastern Colorado Health Care System, Veterans Health Administration, Aurora, CO, USA; ^4^VA Center for Health Equity Research and Promotion (CHERP)^.^ Veterans Health Administration, Philadelphia, PA, USA; ^5^University of Pennsylvania Perelman School of Medicine, Philadelphia, PA, USA

###### **Correspondence:** Heather Gilmartin (heather.gilmartin@va.gov)

**Background:** Evaluating the reach of an intervention is an important implementation science measure. The Veterans Health Administration (VA) Transitions Nurse Program (TNP) is a national care coordination program focused on rural Veterans. TNP stakeholders request feedback on the locality of participants to understand program impact. Geographic information systems (GIS) is a data analytic framework that renders complex information into interactive maps. The purpose of this project was to assess the value of GIS mapping as a communication tool to provide feedback regarding the reach of the TNP program.

**Methods:** GIS maps were built using ArcGIS Enterprise to determine the location of enrollees (n=192) at one TNP site from April 2017 to May 2018. Enrollee geocoded residential addresses were matched to USDA’s 2000 Rural-Urban Commuting Areas (RUCA) to characterize their residence as urban, rural, or highly rural. RUCA is a rurality standard used in the VA. Static heat maps and an interactive point map were presented to stakeholders. The interactive point map uses a web-based application allowing users to analyze the data by turning on/off layers of data, zooming into specific areas, and adding more data. Value of the maps as a communication tool was assessed by rapid thematic analysis of stakeholder feedback.

**Findings:** Stakeholders reacted positively to the interactive point map due to the level of detail presented and the ability to explore spatial patterns that were not apparent in the raw data or static heat maps. The heat maps presented enrollment density, which was less valued than the physical location of the enrollee. One stakeholder stated, “all I look at is names and numbers – to see this on a map is incredible.” Post-presentation discussion included the following themes: value of maps for presentations to VA leadership and Veterans, role of maps in maintenance efforts, confirmation of current TNP recruitment strategies.

**Implications for D&I Research:** Interactive GIS point maps were deemed a valuable communication tool that required little explanation to TNP stakeholders. The innovative approach facilitated the visualization of the reach of TNP. This has the potential to positively impact maintenance efforts.

### S22 Using mobile phones to deliver mental health services to homeless young adults

#### Stephen Schueller (s.schueller@uci.edu)

##### University of California at Irvine, Irvine, CA, USA

**Background:** Young adults experiencing homelessness have significant mental health needs but low access to and uptake of mental health services. BITs offer a promising avenue to reach this population. However, the relative lack of BITs for this population requires a focus on early stage outcomes, like acceptability and feasibility, to guide future work. Acceptability refers to consumer-perceived usefulness or satisfaction. Feasibility refers to whether the evidence-based practice can be successfully used or carried out within a given context.

**Methods:** We assessed BIT acceptability and feasibility for the implementation of a remotely-delivered mobile phone intervention for sheltered homeless young adults aged 18 to 24 years. Participants were provided a mobile phone, service and data plan, suite of mobile apps, and one-month of remotely-delivered support from a provider consisting of phone sessions, text messages, and phone check-ins. Acceptability was measured by self-report and an in-app rating system. Feasibility was measured through passively collected system use data as well as attendance in phone therapy sessions and engagement with the provider.

**Findings:** We found high rates of acceptability, with 100% of participants indicating that they would recommend participation to someone else and 52% reporting they were “very” or “extremely” satisfied with their participation. Participants were most enthusiastic about the daily tips, with 64% indicating they liked them “quite a bit” or “a lot.” Participants were least enthusiastic about the mobile apps, with only 26% indicating they liked them “quite a bit” or “a lot.” Most participants (57%) completed all three of their therapy phone sessions. Participants sent an average of 15 text messages to the therapist, while receiving 19 text messages from the therapist during the intervention period.

**Implications for D&I Research:** This example of BIT implementation measurement demonstrates the active and passive data collection methods used for the assessment of acceptability and feasibility. Acceptability relies on the perceptions of users, but can be measured using methods both within and external to the BIT. Feasibility assessment includes context-dependent participant use of the BIT, the measurement of which can be facilitated by the technology itself. Acceptability and feasibility measurement can inform the design of BITs, accompanying services, and implementation strategies.

### S23 Measuring adoption and fidelity of a digital measurement feedback system in an outpatient pediatric behavioral health system

#### Aaron Lyon (lyona@uw.edu)

##### University of Washington, Seattle, WA, USA

**Background:** Measurement feedback systems (MFS) are BITs that support the patient interactions and clinical decisions of behavioral health providers via routine administration of assessments. The use of MFS improves patient outcomes, but only if providers and patients use them as intended. In the context of BITs, adoption is the initiation of technology use. Fidelity to BITs reflects the extent to which actual use matches expected use, as determined by the BIT developer. This presentation will report on the implementation of a MFS in an outpatient pediatric behavioral health system with attention to the operationalization and measurement of MFS adoption and adherence (a dimension of fidelity) from both provider and patient perspectives.

**Methods:** A MFS providing standardized assessment of symptoms and functioning and graphical feedback was implemented among N = 70 outpatient providers using training and post-training support strategies (e.g., consultation, incentives) in the context of ongoing plan-do-study-act cycles. Over a five-year implementation period, these providers had contact with N = 5119 patients. Provider adoption was measured by having at least one patient on their caseload with a MFS account. Patient adoption was measured by completion of at least one measure in the MFS. MFS adherence for providers was measured by consistency of (weekly) system logins. For patients, adherence was measured as the completion of baseline (intake) and repeated measures over time.

**Findings:** Provider adoption rose from 10% to 100% during the first 16 months. Provider adherence increased from 48% to 75% of providers logging in at least once a week over a 22-week period during months 11 through 16. Over the five-year period, the percentage of new and existing patients completing baseline and repeated measures, respectively, increased. Baseline measure completion improved from 35% to 80% (R2=0.80; p<.001) and repeated assessments from 14% to 40% (R2=0.76; p<.001).

**Implications for D&I Research:** Efficient and objective measurement of adoption and adherence at both the provider and consumer levels in a long-term study of BIT implementation is possible using readily accessible data passively collected by the BIT. In the future, such data may be used to drive additional strategies for BIT implementation, such as ongoing audit and feedback.

### S24 Implementing a mobile health system to integrate the treatment of addiction into primary care: Cost, penetration, and sustainability

#### Andrew Quanbeck (arquanbe@wisc.edu)

##### University of Wisconsin – Madison, Madison, WI, USA

**Background:** A research trial assessed the costs and effects associated with implementing an evidence-based BIT for addiction treatment, an mHealth system named “Seva.” This presentation describes the measurement of costs, penetration, and sustainability associated with implementing Seva.

**Methods:** Seva was introduced over 36 months in three primary care clinics across the U.S. Providers offered Seva to a maximum of 100 patients with addiction at each clinic. Penetration was defined as the proportion of patients accessing Seva weekly and the proportion of providers referring to Seva. Sustainability was defined as continued use of Seva after the first 12-months of grant-funded use. Cost was addressed from the perspective of a healthcare system administrator. The cost analysis included operating costs (e.g., mobile phones, data plans, provider time) and implementation costs (e.g., site visits, teleconferences). Implementation costs were organized using the Cost of Implementing New Strategies model.

**Findings:** Table 1 reports patient and provider penetration as well as implementation and operating costs. Patients used Seva at high levels, while provider referral and use was limited. Only 1-3 providers regularly used Seva in each clinic, and behavioral health providers made the most referrals. Although two of the three clinics wanted to sustain Seva use, they struggled to find ongoing funding after research funding lapsed, and use ultimately dropped to zero at all sites. Overall cost/patient across all sites was approximately $1,400; for comparison, the average cost/patient of an episode of outpatient addition treatment in 2008 was $2,325.

**Implications for D&I Research:** The study operationalized and measured the outcomes of penetration, sustainability, and cost, areas where systematic BIT implementation research is particularly scarce. From the perspective of health system administrators, cost maybe virtually synonymous with sustainability. Key challenges remain in determining how to promote BIT use among providers and fund ongoing use of such systems.

**Table 1 (abstract S24).** Seva Penetration and Cost

	Site 1	Site 2	Site 3
Penetration (%)			
Patients using	60	53	55
Primary care providers referring	8	92	24
Behavioral health providers referring	92	8	76
Cost (US Dollars)			
Operating	113,636	117,150	83,177
Implementation	9,948	23,345	28,121
Total clinic	123,584	140,495	111,298
Cost per patient	1,274	1,405	1,568

## Building the Future of D&I Science: Training, Infrastructure, and Emerging Research Areas

### S25 Building social networks to support implementation: A systematic scoping review of network interventions

#### Alicia Bunger^1^, Reza Yousefi-Nooraie^2^, Lisa Juckett^1^, Elena Navarro^3^

##### ^1^College of Social Work, Ohio State University, Columbus, OH, USA; ^2^Toronto, ON, Canada; ^3^Kaiser Permanente Washington Health Research Institute, Seattle, WA, USA

###### **Correspondence:** Alicia Bunger (bunger.5@osu.edu)

**Background**: Since social networks transmit knowledge, influence, and resources among patients, professionals, and organizations, network interventions (interventions that change social networks) have potential to be used as implementation strategies that promote adoption, implementation, and sustainment. Yet, the types, mechanisms, and effectiveness of these network interventions are unclear, limiting our understanding and ability to implement innovations within complex healthcare contexts. This systematic scoping review identifies, characterizes, and describes network interventions, and their effects to advance effective strategies for implementing change.

**Methods**: forward and backward citation tracking of seminal papers on network interventions and conducted a bibliometric search using a sensitive search strategy. Two authors independently applied inclusion/exclusion criteria based on title/abstract. Inclusion criteria: studies examining interventions intended and/or expected to change social networks, network measurements at multiple time points, and in English. Exclusion criteria: reviews, theoretical papers, studies focused on IT, neural, or genetic networks. During full-text reviews, information about the network interventions, actors, ties, and main findings were extracted.

**Findings**: Our search yielded 2048 studies. We included 18 studies out of 464 that were screened through citation tracking. Fourteen focused on networks among individuals (professionals or patients), and 4 focused on inter-organizational networks. In 7 studies, changing social networks was not the main intention; instead, these studies mainly focused on training and capacity building which affected the networks as by-products. Of the 11 studies that intended to change networks, 2 used the baseline network maps to inform intervention tailoring and adjustment (e.g. by focusing on observed gaps). The rest used strategies for cohesion building/strengthening (e.g. small group activities to facilitate communication and support), recognition building (e.g. increasing the centrality of certain actors/champions), partnership/coalition development (e.g. facilitation of advice-seeking and referrals), expansion of ego-networks (e.g. by facilitating online socialization activities in socially isolated individuals), and cutting ties (e.g. reducing the publicity of bullies at schools).

**Implications for D&I Research**: Networks are ubiquitous throughout implementation frameworks and theories, yet interventions that leverage or change networks are under-developed and utilized in the field. Advancing these interventions will require greater theoretical specification, development of strategies that target professionals and organizations, and studies that examine the impact on implementation outcomes.

### S26 Experiences and outcomes of the UCSF training program in implementation science

#### Priya Shete, Margaret Handley, Ralph Gonzales, Sara Ackerman, Adithya Cattamanchi

##### University of California San Francisco, San Francisco, CA, USA

###### **Correspondence:** Priya Shete (priya.shete@ucsf.edu)

**Background:** The UCSF Implementation Science (ImS) Training program introduced six graduate-level courses in 2008 that can be taken individually or as part of a Certificate Program in Implementation Science. The courses and Certificate program have also been offered in an online format for external trainees since 2016. We evaluated outcomes of trainees who have completed the Certificate program.

**Methods:** All students who completed the in-person Certificate program (2008-2015), or the online Certificate program (2016-2017) were eligible. In order to assess the potential impact of the Certificate program on the professional development of trainees, we surveyed participants on their self-reported level of comfort with pre-defined competencies and on academic productivity since completion of the Certificate program.

**Findings:** Of eligible trainees, 54 in-person (77%) and 13 (100%) online Certificate participants completed surveys. In-person trainees reported a total of 147 implementation science-related publications in peer-reviewed journals (median 3 publications/trainee, IQR 1-15). Thirty-four trainees (63%) reported being a Principal Investigator (PI) of 64 funded implementation science-related grants (median 2 grants/trainee, IQR 1-4). 15% (n=8) of participants reported being PI an NIH grant, including R01 or P01 level funding (n= 4, 7%) and K awards (n=3, 6%). The median level of competence for in-person trainees was reported at 4 (high confidence) for 9 of the 12 competencies assessed, and at 3 or 3.5 (moderate confidence) for the remaining three. The median level of competence following certificate training for online trainees was reported at 4 (high confidence) for 5 of the 12 competencies assessed, and at 3 or 3.5 (moderate confidence) for the remaining 7. Lower level of competency was reported for confidence in skills aligning with later stages of pilot projects and trials for both groups.

**Implications for D&I Research:** Trainees completing the UCSF Certificate Program in Implementation Science reported moderate to high confidence in all competencies assessed and reported a high level of academic productivity. These data support the benefit of intensive, graduate-level training focused on applied methods to support career development of implementation scientists, and that such training can be delivered in an online learning format to increase accessibility.

### S27 A tiered training model to build systemwide capacity in implementation science - current learning and future research

#### Rohit Ramaswamy, Byron Powell

##### Gillings School of Public Health, University of North Carolina, Chapel Hill, NC, USA

###### **Correspondence:** Byron Powell (bjpowell@unc.edu)

**Background:** Globally, there is a need to provide implementation science training and coaching to a range of stakeholders across all career levels in research, practice, and policy. Many training programs are targeted at researchers, are competitive, and involve long duration immersive training. This is neither practical, nor may be necessary for all audiences, signaling a need for flexible scalable models with diverse pedagogies and delivery methods.

**Methods:** In the past four years, we have trained 38 graduate students and over 50 participants from research institutions, implementing agencies and the government through a combined online/face-to-face 10-day course at Wits University in South Africa. Course participants called for a more nuanced approach to training that addresses diverse learning needs. We identified a four-tier hierarchy of training candidates including organizational leaders (Tier 1); policy makers (Tier 2); front line implementers (Tier 3); and researchers and implementation specialists (Tiers 4a and 4b). We created a learning needs matrix for each tier, and developed ten design principles (e.g. development of tier based competencies, common body of knowledge for each tier, coaching etc.) for a successful targeted program. We created a multi-tier integrated capacity building model aligned with these principles. To date, we have developed programs for Tiers 1(0.5 days), 3 (2.5 days) and 4 (10 days).

**Findings:** Participant evaluations indicate that that our model meets the diverse learning needs of trainees at each tier. Additionally, we have gained experience on how to structure the content modules for each tier, and on adaptable delivery mechanisms that provide topic-specific case studies and post-training consulting. We will present and discuss these findings and the structure of future models incorporating case study libraries, collaborative researcher/practitioner training and learning networks that have not been tested to date.

**Implications for D&I Research:** For implementation science to have impact, leaders and policy makers, researchers and implementers must all be trained to work together. Our tiered program is one of the only models that intentionally targets the needs of various stakeholders and explicitly creates coaching and collaborative models to optimize learning and use. With additional testing, lessons learned can be an exemplar for scaling up implementation science training globally.

### S28 System factors and their influence on implementation success, provider skill attainment and fidelity: What is truly “malleable”?

#### Eric Bruns^1^, Elizabeth Parker^1^, Jonathan Olsen^1^, Spencer Hensley^1^, Michael Pullmann^1^, Marlene Matarese^2^, Kim Estep^2^, Michelle Zabel^2^

##### ^1^University of Washington School of Medicine, Seattle, WA, USA; ^2^University of Maryland School of Social Work, Baltimore, MD, USA

###### **Correspondence:** Eric Bruns (ebruns@uw.edu)

**Background:** Implementation frameworks consistently identify variables in the “outer context” proposed to influence implementation and uptake of evidence-based practices (EBP). However, research on the actual influence of these factors is sparse compared to “inner context” (i.e., organization variables). This session will present new research that (1) examines what malleable (and unmalleable) state factors are associated with EBP implementation, research investments, and data policies; and (2) illustrates this phenomenon through a multi-state study of the influence of states’ behavioral health policy and finance context on implementation success, provider skills attainment, and fidelity.

**Methods:** Study 1: Annual state surveys by the NASMHPD Research Institute (NRI; response rates 86% - 98%) provided data on use of EBPs and data and research investments; publicly available datasets provided data on other state factors (e.g., per capita income, Medicaid expansion, budget status, political party control). Study 2: As part of its workforce and implementation support efforts in states, the National Wraparound Implementation Center collects data using the Stages of Implementation Completion and the Wraparound Fidelity Index, a validated measure of fidelity.

**Findings:** In Study 1, multilevel models found “unmalleable” factors significantly (p < .05) related to state EBP investment including state per capita income; Democratic control of the state; and state Medicaid expansion status. “Malleable” factors included state behavioral health authority independence, direct SBHA funding of services, and collaboration among state agencies. Study 2 provides an illustrative example of such dynamics: After controlling for other factors, results of multiple regression models found more rapid implementation success and higher fidelity in 6 states directly funded and supported wraparound through “care management entities” (CMEs) versus Community Mental Health Centers (n=5).

**Implications for D&I Research:** There are interpretable state “outer context” predictors of EBP investment and success, some of which are malleable. Better understanding of these dynamics may aid in setting policy and research priorities, as well as refining implementation frameworks and predictive models for implementation research.

### S29 The relationality of intervention, context, and implementation: A prospective case study Examining the adoption of an evidence-informed nursing care model

#### Miriam Bender^1^, Paige Burtson^2^, Deborah Lefkowitz^1^

##### ^1^University of California, Irvine, Irvine, CA, USA; ^2^University of California San Diego Health, San Diego, CA, USA

###### **Correspondence:** Miriam Bender (miriamb@uci.edu)

**Background:** Implementation science recognizes that evidence-informed interventions, context (people, things, and processes that characterize where the intervention is introduced), and implementation (processes of intervention integration) interact with each other in complex ways. However, there is limited research focusing on how these interactions occur in healthcare change. This study explored how one health system changed their nursing model of care, paying equal attention to the nursing intervention, the frontline clinical context, and implementation processes.

**Methods:** Prospective case study design was used to study the model’s implementation in 5 ‘pods’ within a newly-opened 364-bed academic medical center in California. The model embedded Clinical Nurse Leaders (CNL) into the nursing care team. CNL is a Registered Nurse with masters-level competencies in clinical leadership, care environment management, and clinical outcomes management, utilized in an evidence-informed clinical workflow to improve frontline clinical processes. Data collected between 2016-2018 include interviews (n=21), focus groups (n=1), open-ended survey responses (n=31), and observation (4 planning meetings, 16 hours clinical observation). Data were analyzed using deductive (operationalizing intervention, context, and implementation) and inductive (identifying influences between concepts) qualitative content analysis.

**Findings:** Implementation involved system-level strategies, including service-line leadership and resource provision, pod-level manager engagement, and education. Nonetheless, the CNL workflow was initially rejected after rollout by each pod’s multidisciplinary clinicians, and CNLs reverted to familiar task assistance activities (e.g. administering a medication). However, over time, with continued system-level support, CNLs and clinicians developed new workflows together. Existing clinician practices shaped, but were also shaped by, the CNL practices that were adopted (e.g. pods with CNL-coordinated interdisciplinary rounding structures). This resulted in heterogeneous CNL workflows, with differing levels of evidence-informed CNL practices across pods.

**Implications for D&I Research:** A focus on interactions between intervention, context, and implementation made visible a relational process whereby the intervention was translated into context-specific workable practices, which explains the heterogeneity of implementation results. Findings suggest interventions can be conceptualized as potential resources rather than robust healthcare solutions, and that inquiry into resource use, framed by an understanding of the interdependence of interventions, contexts, and implementation efforts, can provide insights into mechanisms of change that are generalizable.

### S30 The sustainability of quality improvement initiatives for older adults: A mixed methods mapping of contexts, antecedents and consequences

#### Tim Rappon, Alyssa Indar, Erica Bridge, Whitney Berta

##### University of Toronto, Toronto, ON, Canada

###### **Correspondence:** Tim Rappon (tim.rappon@mail.utoronto.ca)

**Background:** Quality Improvement (QI) initiatives are proposed as key vehicles to shift our health system from disease-focused, episodic care to comprehensive care for older adults living with chronic conditions. With the proportion of older Canadians set to double over the next two decades, the need for QI is pressing, yet there is a dearth of research on whether QI yields long-term benefits for older patients and residents. Our study responds to Proctor *et al.*’s (2015) call to investigate factors predicting sustainability.

**Methods:** We employed a novel combination of structured coding, thematic analysis and a Kohonen self-organizing map (an artificial neural network) to identify patterns of contexts, implementation strategies and adaptations associated with long-term use of, and benefits from, QI interventions. To populate our map, we searched Medline, PsychINFO and CINAHL for articles which reported on the long-term (1+ years post-implementation) sustainability of QI programs which targeted older adults (over 65) or geriatric syndromes (e.g. dementia, incontinence). After screening 3127 abstracts, 91 papers were selected for inclusion. Two coders independently extracted study characteristics, including measures of sustainability, implementation and post-implementation strategies (coded using the Powell *et al.* compilation), and adaptations (using the Stirman Adaptations Framework). Extracted articles were used to train a Kohonen self-organizing map, which groups similar studies together in 2D space. Heatmaps were analyzed for relationships between implementation context, (post-)implementation strategies, adaptations and successful sustainment and/or sustainability.

**Findings:** We report on clinical targets & settings for QI, how sustainability was defined and measured, adaptations, costing (ROI), and (post-)implementation strategies. Although we did not find clear relationships between implementation strategies or adaptations and sustainability, we did find that post-implementation use of educate or restructure strategies was associated with sustainability across multiple contexts (i.e. homecare, nursing homes, hospitals, primary care). Our results suggest that the ability of an intervention to demonstrate ongoing benefits to stakeholders, continuing staff education and a QI-friendly implementation context are key predictors of long-term, sustainable QI.

**Implications for D&I Research:** Our study presents a novel method for investigating relationships between (post-)implementation factors and sustainability, which could be extended (with a larger training dataset) to produce predictions of intervention sustainment/sustainability

### S31 The persistence of low-value HIV prevention interventions: Are organizations hanging on?

#### Virginia McKay, Todd Combs, Sarah Sedlack, Chao Cao

##### Brown School of Social Work, Washington University in St. Louis, St. Louis, MO, USA

###### **Correspondence:** Virginia McKay (virginia.mckay@wustl.edu)

**Background:** The extent to which low-value interventions persist in practice settings with the availability of more effective or efficient interventions is unclear. The effectiveness and efficiency of evidence-based interventions promoted by the Centers for Disease Control and Prevention (CDC) to control the HIV epidemic in the United States has evolved over the last several decades – providing an ideal opportunity to examine the persistence and de-implementation of low-value interventions. We will present the results from an ongoing study to describe the persistence of low-value HIV prevention interventions in public health settings.

**Methods:** We will have surveyed organizations in at least 15 metropolitan statistical areas with the highest HIV incidence. Organizations are recruited from the CDC’s website gettested.org and are eligible to participate if the organization had provided at least one of 37 HIV prevention interventions identified as inefficient by the CDC. One staff member with intervention oversight is invited to respond. Participants are asked about intervention implementation and the decision to de-implement or continue the intervention. Based on preliminary results from 8 cities and 201 recruited organizations, 66% of organizations responded (n=133). Of responding organizations, 41% were eligible (n= 55) and reported on 145 low-value interventions.

**Findings:** We will present the current results from our survey to describe how often organizations opted to continue or de-implement a low-value intervention, the characteristics of organizations more likely to continue low-value interventions, and the kinds of interventions most likely to be continued (e.g., individual or group-level interventions). Based on our preliminary results, 47% of organizations opted to continue at least one low-value intervention. Individual-level interventions, like risk reduction counseling, were continued approximately 62% of the time.

**Implications for D&I Research:** Many organizations continue low-value interventions and some interventions are more likely to be continued, like individual-level interventions, than others. Using the limited available resources for HIV prevention and other pressing public health issues in the most effective and efficient manner is imperative. Understanding how and why low-value interventions persist will help determine whether organizations require support for de-implementation alongside efforts to implement more effective or efficient interventions.

### S32 Familial hypercholesterolemia: A prototype for implementation science in genomics

#### Whitney Barfield^1^, Mindy Clyne^2^, Muin Khoury^3^

##### ^1^National Heart, Lung, and Blood Institute, National Institutes of Health, Bethesda, MD, USA; ^2^National Cancer Institute, National Institutes of Health, Rockville, MD, USA; ^3^Centers for Disease Control and Prevention, Atlanta, GA, USA

###### **Correspondence:** Whitney Barfield (whitney.barfield@nih.gov)

**Background:** Familial hypercholesterolemia (FH) is a common genetic disorder that increases risks of premature cardiovascular disease and death, affecting every 1 in 250 persons in the U.S. Despite the clinical severity of FH, very few individuals and their relatives are screened and diagnosed for effective large-scale management of the disease through early intervention with LDL lowering medications, such as statins and ezetimibe. Only about 50% of adults with definite/probable FH are actively on statin therapy. Cascade screening is highly effective for national family screening efforts, as a Tier 1 evidenced-based genomic screening application, yet many barriers and challenges prevent optimal implementation. A better understanding of the current NIH research landscape surrounding FH is needed to guide strategic planning and future programmatic efforts.

**Methods:** We performed a 20-year systematic portfolio analysis of NIH grants (1998-2017) focused on, or related to FH using the NIH Research Portfolio Online Reporting Tools Expenditures and Results (RePORTER) system to mine titles, abstracts, and terms. After generating search results, three coders manually coded grants by its place within the translational spectrum (T0-T4) and described the implementation science focal area (if relevant).

**Findings:** There were a total of 99 extramural grants supporting FH related research over the last 20 years. Unfortunately, only three grants fell within the T3 translational phase. These grants focused on several topics including electronic health records for surveillance/diagnosis of FH, family-centered communication around dissemination of results, and tools to facilitate patient/provider shared-decision making. There were zero grants in the late-stage T4 translational research phase.

**Implications for D&I Research:** The portfolio analysis identified major gaps in implementation science research for FH with limited studies focused on late-stage translation, despite evidenced-based genomic screening and available treatments. Robust late stage translational research initiatives are sorely needed to address barriers to adoption, sustainability, and scalability of proven-effective applications such as cascade screening in FH research.

### S33 Co-production of applied health research to ensure its implementation: A UK perspective

#### Roman Kislov, Sarah Knowles

##### Alliance Manchester Business School, University of Manchester, Manchester, United Kingdom

###### **Correspondence:** Roman Kislov (roman.kislov@manchester.ac.uk)

**Background:** Co-production, involving collaboration with policymakers, clinicians and managers throughout the research process, is increasingly seen across the globe as a promising approach to translating evidence into better healthcare. Despite the growing importance of the impact agenda and the proliferation of collaborative research partnerships, awareness about the practical realities of co-production remains low. The aim of this study was to explore the processes, mechanisms and consequences of co-production between researchers and practitioners as an approach facilitating the implementation of research in healthcare organisations.

**Methods:** A multiple case study was conducted in 2016-2018 in a large-scale UK-based collaborative research partnership bringing together producers and users of applied health research. Four applied health research projects were selected, reflecting variation both in *the type of research* conducted (exploratory research vs implementation research) and the perceived *strength of relationships* between the research teams and the partner organisations (established partnerships vs new collaborations). In total, 41 semi-structured face-to-face interviews were conducted; these were supplemented by observation (60 hours) and documentary analysis.

**Findings:** Co-production approaches differed depending on the stage(s) of the research process in which they were deployed as well as on the type of stakeholders involved; all of them required a number of compromises directly affecting the collaborators. Contrasting the expectations of healthcare practitioners with the researchers’ way(s) of doing things, we categorise these compromises into three broad groups: (1) complementing ‘research’ by ‘non-research activities’, such as improvement and education; (2) opening up the research team to include project managers, practitioners, and service users as well as to bring together researchers espousing different epistemological and methodological paradigms; and (3) adapting to a practice-driven agenda and embracing impact as an essential component of evaluation and research.

**Implications for D&I Research:** This study highlights the complexities of deploying co-production as an implementation strategy: (1) Co-production is a time- and labour-intensive approach; (2) compromise can extend through to interpretation and reporting of results, whereby negative results may be sensitive for the healthcare organisations; (3) junior staff are more vulnerable to the negative consequences of co-production. These considerations should be taken into account when deploying co-production approaches and developing ‘implementation-savvy’ researchers.

### S34 Integrated knowledge translation: A Canadian perspective on co-production

#### Anita Kothari^1^, Ruta Valaitis^2^, Marlene Janzen Le Ber^3,4^, Selma Tobah^5^

##### ^1^School of Health Studies, Western University, London, ON, Canada; ^2^School of Nursing, McMaster University, Hamilton, ON, Canada; ^3^School of Leadership and Social Change, Brescia University College, London, ON, Canada; ^4^Ivey Business School, Western University, London, ON, Canada; ^5^Faculty of Health Sciences, Western University, London, ON, Canada

###### **Correspondence:** Anita Kothari (akothari@uwo.ca)

**Background:** Integrated knowledge translation (IKT) is a model of collaborative research where researchers work with knowledge users who identify a problem and have the authority to implement the research recommendations. The purpose of this presentation is to reflect on our experiences of using IKT in a complex policy environment and highlight broader lessons about IKT as a promising implementation approach.

**Methods:** A retrospective, multiple case study of three IKT research projects was conducted; each project/case was situated in public health practice and policy. Case 1 was done in partnership with municipal government; Case 2 represented a partnership across two provinces while Case 3 was carried out with provincial policymakers. The primary research question was: *To what extent is IKT a promising implementation approach?* Participant observation, team de-briefings with each of the three research teams, and analytical auto-ethnography were the main methods of data collection. Supplementary semi-structured interviews with research team members and the social impact model of co-produced research also informed the analysis.

**Findings:** Seven tensions related to the IKT approach and health policymaking are identified: (1) dealing with positivity bias; (2) ‘doing implementation’ versus contributing to the science of implementation; (3) accepting high involvement costs; (4) providing ‘extras’ to partners to secure their buy-in; (5) resolving power issues; (6) balancing rigour against responsiveness when sharing preliminary findings; and (7) trade-off between serendipity and strategy when activating policymakers’ networks. These tensions are discussed in relation to the evidence base, policy actors and decision-making process.

**Implications for D&I Research:** Implications are drawn for different stakeholder groups (e.g., funders, government, scientists) to address each tension, highlighting the overarching need for IKT as an implementation approach to be targeted and tailored. The extent to which these tensions can be resolved varies in scope. Addressing some tensions may require difficult conversations and memorandums of understanding among partners and other stakeholders, while dealing with other tensions would benefit from further scientific inquiry. The learnings from this case study point to how IKT might be improved to be a more effective implementation approach and highlight the need for rigorous evaluations of its processes and outcomes in different policy contexts.

### S35 Applying principles of co-production to solve wicked problems in healthcare: An Australian perspective

#### Gill Harvey, Amy Marshall

##### School of Nursing, The University of Adelaide, Adelaide, Australia

###### **Correspondence:** Gill Harvey (gillian.harvey@adelaide.edu.au)

**Background:** Wicked problems in healthcare are issues that are complex, challenging, understood differently by diverse stakeholders and hence difficult to solve. Improving the integration of services across the spectrum of care delivery represents one example of a wicked problem facing healthcare systems across the globe. Multiple initiatives have been introduced, yet evidence of effectiveness remains mixed and inconclusive. Adopting principles of co-production to engage all stakeholders and develop local solutions that are contextually relevant offers an alternative way to more typical broad-brush policy approaches.

**Methods:** We present the findings of a case study conducted in 2014-2018 in an Australian healthcare context. The study addressed the problem of fragmented care for older people who are frequent users of acute hospital services and involved a strong reflective component aiming to explore the realities of co-production as an implementation strategy. Multiple methods of data collection were employed throughout the co-production process, including notes of regular stakeholder meetings, medical record review to map patient pathways (n=17), semi-structured interviews with 10 older people, observation of 5 focus groups and ‘living laboratory’ events with consumers and care providers.

**Findings:** There is significant will and desire amongst all stakeholder groups to improve implementation across multiple boundaries. However, translating this stated objective into achievable improvement is fraught with challenges, not least because of the prevailing policy context. For example, within Australia, there are different funding mechanisms for acute and primary care services, a lack of shared platforms for accessing patient records and ongoing reforms to the provision of health and social care for older people. This presents a dilemma in terms of how to move from co-production of research solving real-life problems at a local level towards overcoming the system-level barriers that impede the realisation of local solutions.

**Implications for D&I Research:** Co-production is not for the faint-hearted! It takes time, patience and resilience on the part of researchers - characteristics not valued by conventional academic metrics. Moreover, researchers have to consider the moral issue of mobilising knowledge and action at a local level if the prevailing policy context presents insurmountable barriers.

### S36 Fast-tracking the future: Advancing the translation of personalized genomics-informed smoking cessation treatment

#### Alex Ramsey, Ami Chiu, Li-Shiun Chen, Laura Bierut

##### Washington University School of Medicine, St. Louis, MO, USA

###### **Correspondence:** Alex Ramsey (aramsey@wustl.edu)

**Background:** The efficacy of smoking cessation pharmacotherapy is in part driven by one's genetic makeup. Using clinically-valid genomic applications to inform medication response can optimize treatment, but implementation requires acceptability of genetic testing among smokers. This study examined (1) consumer demand for genetic susceptibility testing for health risks and nicotine dependence, (2) receipt of returned genetic results, and (3) desire to take smoking cessation medication when hypothetical genetic results predict the pharmacogenetic medication response.

**Methods:** Current smokers from a genetic nicotine dependence study (*n*=1306) and a smoking cessation trial (*n*=209) were surveyed on their desire to receive health- and smoking-related genetic testing results. A subset of current smokers (*n*=705) was given the opportunity to access personalized genetic results online; another subset (*n*=474) was surveyed on the desire to take medication given hypothetical below- or above-average pharmacogenetic medication responses.

**Findings:** Most current smokers reported high desire to receive personalized genetic results on health risks (85.8%) and on their chances of quitting smoking (84.8%). Factors associated with high interest included age ≥40 years, having a college degree, and ≥1 medical condition. Despite high interest, only 189 (27%) accessed their personalized genetic results online. Smokers more likely to access results included Caucasians, women, and those with a high school diploma and household income above federal poverty level. In the smoking cessation trial, when the expected medication response changed from below- to above-average, significantly more smokers reported a desire to take medication (from 61.0% to 97.5%, *p*<.0001).

**Implications for D&I Research:** Among smokers, a group concentrated in lower socioeconomic status, there is high demand for genomics-informed smoking cessation treatment. Dedicated efforts must address barriers to access, however. Although gender, race, and income were not associated with smokers’ desire for genetic results, these factors influenced the likelihood of actually receiving genetic results. A positive hypothetical pharmacogenetic response to smoking cessation medication increases desire to take genetically-efficacious medication. These data provide insights on the acceptability and clinical utility of smoking-related genomic applications, which are key drivers in designing for accelerated translation (DART) of myriad emerging precision medicine innovations. Studying the implementation of genomics-informed treatment in clinical settings represents a key next step.

### S37 Accelerating translation of self-management strategies through automated telehealth and mhealth to reduce health disparities for high risk populations

#### Stephen Bartels^1^, Sarah Pratt^2^, Kelly Aschbrenner^3^, Karen Fortuna^4^

##### ^1^Dartmouth Institute for Health Policy and Clinical Practice, Geisel School of Medicine at Dartmouth, Lebanon, NH, USA; ^2^Geisel School of Medicine at Dartmouth, Concord, NH, USA; ^3^Geisel School of Medicine at Dartmouth, Nashua, NH, USA; ^4^Geisel School of Medicine at Dartmouth, Lebanon, NH, USA

###### **Correspondence:** Stephen Bartels (Stephen.bartels@dartmouth.edu)

**Background:** People with serious mental illness have reduced life expectancy of 11 to 25 years, largely due to cardiovascular disease and diabetes and related risk factors including high rates of obesity, tobacco use, and sedentary behavior. Illness self-management interventions have been shown to be effective in enhancing positive health behaviors and adherence to self-monitoring and effective treatments. However, intensive self-management interventions specifically tailored for this high-risk group are challenging to deliver and to sustain over time due to the required workforce and ongoing training and support of the affected individuals. Automated telehealth and mobile health interventions present practical solutions to expedite the implementation, scalability, and sustainability of effective self-management and peer support strategies.

**Methods:** This presentation will provide an overview of a series of formative studies and ongoing clinical trials specifically targeting concurrent self-management of both physical and mental health conditions by people with serious mental illness including automated telehealth and peer supported mobile health interventions.

**Findings:** A preliminary six-month pre-post study of 106 adults with serious mental illness demonstrated improved psychiatric self-management and psychiatric symptoms, improved health self-efficacy, improved diabetes self-management, and decreased acute service use. Based on these results, two subsequent randomized trials are being conducted. Baseline characteristics and the study design will be described for these two concurrent trials of automated telehealth including (1) a study of 300 participants with psychiatrically unstable serious mental illness to improve psychiatric symptom and acute service use outcomes; and (2) a study of 300 participants with serious mental illness and chronic health conditions to improve medical and psychiatric self-management and acute service use outcomes. Preliminary results from a study adapting automated telehealth for mobile delivery suggests high potential to maximize intervention timeliness, reach, and sustainability through a trained workforce of peers with serious mental illness.

**Implications for D&I Research:** In fitting with the designing for accelerated translation (DART) conceptual architecture, automated telehealth with algorithmically triggered self-management strategies and early intervention by health professionals, in conjunction with peer support, is a highly feasible and promising approach to enhancing the timely delivery, use, reach, and sustainability of self-management strategies by people with serious mental illness and medical comorbid health conditions.

### S38 The opioid epidemic: If ever there was a dire need for rapid scale up of evidence-based approaches

#### Mark McGovern^1^, Kendall Darfler^2^

##### ^1^Stanford University School of Medicine, Stanford, CA, USA; ^2^UCLA Integrated Substance Abuse Programs, Los Angeles, CA, USA

###### **Correspondence:** Mark McGovern (mpmcg@stanford.edu)

**Background:** In 2017, conservative estimates of death attributable to opioid overdose in the US was 80,000 persons. While mortality rates continue to escalate, negative public health and societal consequences accrue. Substantial federal and local efforts dedicated to address this epidemic have focused on guideline changes in opioid prescribing, increased availability of rescue medication (naloxone) and expanded access to three treatment medications: methadone, buprenorphine, and naltrexone. These evidence-based medications are associated with reduction in overdose death but are still not widely available. Intervention delivery strategies have been developed to increase reach and adoption of buprenorphine and naltrexone in specialty addiction care and primary care settings and systems. Implementation strategies have been designed and deployed to overcome contextual barriers in both types of settings and systems.

**Methods:** One intervention delivery strategy to increase access is the “Hub and Spoke Model” which originated in Vermont, and is being adapted and launched by multiple states and territories through US 21^st^ Century Cures Act funds. This model organizes networks of primary care and specialty providers to offer expert consultation, ease care transitions and patient flow, and foster adoption of addiction medicines by primary care providers. In this presentation, the original Vermont model and an adapted version now deployed in California are described. Select implementation outcome data of reach and adoption, as a function of barriers encountered and implementation strategies delivered, are reported.

**Findings:** From 2013 to 2016, the number of persons in Vermont on methadone or buprenorphine increased from 800 to 8,000, specialty care organizations prescribing methadone increased from 3 to 5, and number of physicians prescribing buprenorphine increased from 90 to 200. In California, 19 hubs and 153 spoke practices implemented the Vermont model in August 2017. Through June 2018, the number of new patients initiating methadone or buprenorphine increased in both specialty care (3,312) and primary care settings (1,689). Multi-component implementation strategies have addressed some but not all barriers to increase reach and adoption.

**Implications for D&I Research:** Despite effective treatment availability, the access to care gap persists. The opioid epidemic poses an unprecedented challenge and opportunity for D&I research that designs for accelerated translation (DART) of evidence-based delivery strategies.

### S39 An implementation research logic model: A step toward improving scientific rigor, transparency, reproducibility, and specification

#### J.D. Smith (jd.smith@northwestern.edu)

##### Northwestern University Feinberg School of Medicine, Chicago, IL, USA

**Background:** Implementation research (IR) studies are inherently challenging to describe and specify due to the multi-level nature of attempting to change the behaviors, structures, and climate of various individuals within healthcare delivery systems. Improving the specification and description of the various components of an IR study, whether during the planning and proposal stage or its final reporting, can improve rigor, transparency, and reproducibility of the results. Further, uniform descriptions of IR study components using logic models and intervention mapping principles will facilitate the synthesis of findings from the wide-ranging field of D&I.

**Methods:** Using principles drawn from logic models used by the Centers for Disease Control and Prevention and from intervention mapping (Bartholemew et al. 2011), the authors created an IR-specific logic model combining the critical elements of an IR study: determinants and barriers/facilitators (Damschroder et al. 2009), implementation strategies (Powell et al. 2015), mechanisms (Lewis et al. 2018), implementation outcomes, and downstream service or clinical outcomes; Proctor et al. 2011). An iterative process of refinement using an ongoing study examining the impact of implementing a radical new model of care in a large rehabilitation hospital setting was used in the development of the logic model.

**Findings:** The resulting IR Logic Model represents a flexible, comprehensive means of specifying the elements involved in nearly any IR study. It forms the basis for the conceptual foundation of the research and is readily extended to specifying the measurement and analytic plans. A process for indicating hypothesized relationships between elements that relate to the specific aims, hypothesis, and causal pathways is also described.

**Implications for D&I Research:** The IR Logic Model is useful for planning an implementation trial or evaluation; for grant proposals and manuscripts to aide reviewers; and for the reporting of completed trials. Use of a uniform guiding model for each of these steps in the process from conceptualization to primary outcomes reporting is critical for enhancing scientific rigor, transparency, and reproducibility of studies. These are now scored aspects of NIH grant proposals (https://grants.nih.gov/reproducibility/index.htm) and are increasingly emphasized by leading scientific journals in the peer-review process (Nosek et al. 2015). The IR Logic Model offers a solution.

### S40 Mapping the future for dissemination and implementation measurement

#### Cara Lewis (lewis.cc@ghc.org)

##### MacColl Center for Health Care Innovation, Kaiser Permanente Washington Health Research Institute, Seattle, WA, USA

**Background:** Despite significant advances in dissemination and implementation (D&I) science (e.g., strategy compilations, determinants frameworks, reporting standards), measurement continues to lag behind. D&I measures, used to evaluate determinants and outcomes, are typically not developed using gold standard methods, their psychometric evidence is lacking, and they are underspecified in reporting on their empirical uses. These interrelated measurement issues make it difficult to accumulate knowledge across studies.

**Methods:** This presentation provides a synthesis of seven systematic reviews of D&I measures, results from a study to develop the pragmatic measures construct, and new measure of key implementation outcomes. Seven systematic reviews of contextual factors have been conducted in the past decade. This synthesis was supplemented by a review of reporting standards for measurement-related recommendations.

**Findings:** Three of these reviews focused on single constructs, one on key organization-level constructs, and three on numerous constructs depicted in an established D&I model. Only two studies evaluated measures’ content validity and found that 56% and 58.14% of the measures, respectively, had established content validity evidence. Two cross-cutting findings emerged from these reviews: (1) the majority of measures are not psychometrically strong or they have never been tested for their psychometric quality and (2) measures are typically used only in a single study. These findings suggest that the majority of D&I studies are reporting on measures that may not be assessing their intended construct, all facets of the known construct, and may not operate consistently within and across studies. These issues are compounded by the fact that measurement reporting standards are essentially non-existent.

**Implications for D&I Research:** Reporting standards are commonly used as a mechanism for enhancing the transparency, replicability, and quality of research. In addition, by promoting “complete” reporting of a minimum standard across a single type of study, readers are better able to critically appraise and interpret the findings. This presentation puts forth measurement reporting recommendations including internal consistency, construct validity, and criterion validity, and offers measurement best practices to enhance the science of D&I.

### S41 Mapping the future for dissemination and implementation training and workforce development

#### Ross Brownson^1,2^ (rbrownson@wustl.edu)

##### ^1^Brown School, Prevention Research Center, Washington University in St. Louis, St. Louis, MO, USA; ^2^Alvin J. Siteman Cancer Center, Washington University School of Medicine, St. Louis, MO, USA

**Background:** As the field of dissemination and implementation (D&I) science continues to grow, there is increasing urgency and need for new and expanded approaches for building D&I research capacity. Workforce capacity has typically been built via some combination of graduate courses, degree programs, training institutes, workshops, conferences, and online resources.

**Methods:** This presentation provides a synthesis of training approaches, summary of lessons, and gaps for future workforce capacity building needs. The training needs and priorities are based on qualitative findings, empirical evaluations from training programs, and a Concept Mapping exercise to determine current and future needs. In part, findings are based on our collective history in developing graduate D&I coursework and experience from three training programs: Dissemination and Implementation Research in Health, the Implementation Research Institute, and Mentored Training for Dissemination and Implementation Research in Cancer.

**Findings:** Our field is quickly developing a variety of evidence-based approaches to D&I workforce development. Concept Mapping among 120 researchers and practitioners identified nine essential concept clusters: Communicate Research Findings, Improve Practice Partnerships, Make Research More Relevant, Strengthen Communication Skills, Develop Research Methods and Measures, Consider and Enhance Fit, Build Capacity for Research, and Understand Multilevel Context. Based on experience from multiple trainings, competency-based education and mentored training programs appear to be effective in building D&I research skills, developing and sustaining networks of collaborators, and increasing research output (e.g., grants, publications).

**Implications for D&I Research:** The current supply of D&I training programs is insufficient to meet the growing demand. A number of activities are needed to address future capacity building needs. Among these we should: 1) refine existing D&I training competencies to inform future training; 2) create a clearinghouse of materials, including distance training approaches; 3) expand and sustain networks across field; 4) identify new and creative methods to expand training to lower resource settings; 5) enhance evaluation of existing training programs; and 6) explore new methods of training that better link D&I researchers with individuals who implement programs.

## Clinical Care Settings: Patient-level Interventions

### S42 Development and testing of implementation strategies to support community pharmacist-initiated prescribing and dispensing of naloxone to reduce overdose by opioids

#### Benjamin Teeter^1^, Geoffrey Curran^1^, Bradley Martin^1^, Nickolas Zaller^1^, Mary Thannisch^1^, Duane Jones^2^

##### ^1^University of Arkansas for Medical Sciences, Little Rock, AR, USA; ^2^Pharmacy, Harps Food Stores, Inc., Springdale, AR, USA

###### **Correspondence:** Benjamin Teeter (BTeeter@UAMS.edu)

**Background:** Opioid use, abuse, diversion, and overdose deaths have increased dramatically over the past 30 years. Rapid implementation of interventions that mitigate overdose risk are needed. Arkansas Act 284 allows pharmacists to prescribe and dispense naloxone, an overdose antidote, but it is not yet widely implemented. Colleagues at Brown University conducted a successful study in 400+ CVS pharmacies that developed and deployed materials to encourage patients to ask their pharmacist about naloxone. Building on their work, this study selected and locally adapted these materials for use in Arkansas, determined implementation strategies, and tested a proactive intervention for pharmacists to initiate conversations with patients at high-risk for opioid overdose.

**Methods:** This study utilized Evidence Based Quality Improvement (EBQI) to adapt materials and select implementation strategies from the list created by the Expert Recommendations for Implementing Change (ERIC) study. Four 2-hour-long EBQI sessions were attended by the research team, our partner pharmacy’s district manager, 2 pharmacy managers, 2 community informants, and the Arkansas Pharmacists Association’s Vice President for Practice Innovation. The resulting materials and strategies were piloted in 2 pharmacies. Implementation outcomes were evaluated using data from 4 pharmacies (2 pilot/2 comparison). Quantitative naloxone dispensing rates (i.e., level of adoption) and qualitative interviews to explore feasibility, acceptability, and appropriateness of the materials and implementation strategies were used for evaluation.

**Findings:** EBQI resulted in selection, adaptation, and development of 4 posters, 2 pamphlets, a vial sticker, and scripts for conversations with high-risk patients. Analysis of quantitative data 1 month post-implementation found pilot sites initiated 124 conversations with high-risk patients; 40 were prescribed and dispensed naloxone (32.3%). Zero patients were prescribed and dispensed naloxone at comparison sites during the same time period.

**Implications for D&I Research:** Results suggest EBQI was beneficial for adaptation of evidence-based interventions for use in the community pharmacy setting. The pilot of the adapted materials and implementation strategies was very positive. Therefore, at the request of our partner organization, the trial was stopped and development of a roll out strategy to disseminate the materials and strategies to the 29 other pharmacies within their organization has begun. Qualitative evaluation will inform additional modifications needed prior to widespread dissemination.

### S43 Engaging patients – promising strategies to bridge the patient safety chasm in primary care

#### Kelly Smith^1^, Kelley Baker^1^, Margie Shofer^2^, Christine Goeschel^1^

##### ^1^MedStar Institute for Quality and Safety, Columbia, MD, USA; ^2^Agency for Healthcare Research and Quality, Rockville, MD, USA

###### **Correspondence:** Kelly Smith (kelly.m.smith@medstar.net)

**Background:** Breakdowns in communication, diagnosis, medication management, and care coordination result in patient safety gaps within primary care. Engaging patients and families may bridge these gaps. We describe the implementation feasibility of the Guide to Improve Patient Safety in Primary Care Settings by Engaging Patients and Families (the Guide) in medium and large primary care practices from across the U.S.

**Methods:** We conducted a 6-month field test of the Guide strategies to evaluate ease of implementation, acceptability, and usability. Fifteen medium (4-8 clinicians) and large (9 or more clinicians) practices agreed to implement two of the four strategies within the Guide (Teach-Back, Be Prepared to Be Engaged, Create a Safe Medicine List Together, and Warm Handoff Plus) with minimal technical assistance. Site visits were conducted 3 and 6 months post-implementation and included direct observation of clinicians, as well as interviews and focus groups with administrators (n=10), clinicians (47), practice staff (63), and patients, family, and caregivers (51). Optional bi-weekly support calls were also held. Eleven of the 15 practices completed the field test. Withdrawing practices reported changes in leadership or leadership commitment as primary cause of withdrawal.

**Findings:** The field test yielded several important findings. First, practices found the Guide strategies beneficial for patient safety and patient engagement. Second, all stakeholder reported limited time for quality improvement and patient and family engagement (PFE). The pressures for productivity (to see more patients and maintain a short visit time) overshadowed the commitment to PFE. Third, while the strategies were designed to be low complexity, practices were often overwhelmed by the number of materials and had trouble selecting which strategies to implement. Fourth, successful practices used a combination of training and peer coaching as part of their implementation. Fifth, while frontline teams were engaged and enthusiastic about the strategies, leaders needing convincing of the business case.

**Implications for D&I Research:** Field testing identified important barriers to implementation of the Guide, many of which could be mitigated by strategy redesign. The systemic barriers of time, production pressure, training, and leadership engagement are important for primary care researchers to address when planning interventions.

### S44 Development of the IRIS-AR intervention to improve rates of accrual and retention for a patient-directed study intervention

#### Dr. Christine Fahim^1^, Danielle Hylton^2^, Marko Simunovic^2^, John Agzarian^2^, Christian Finley^2^, Wael C Hanna^2^, Yaron Shargall^2^

##### ^1^Johns Hopkins University, Baltimore, MD, USA; ^2^McMaster University, Hamilton, ON, Canada

###### **Correspondence:** Christine Fahim (cfahim1@jhu.edu)

**Background:** The VTE-PRO randomized trial is a pilot study evaluating the impact of extended-duration prophylaxis on venous thromboembolic events for patients undergoing lung cancer resection. Enrolled VTE-PRO participants self-inject either low-weight molecular heparin or a saline placebo for 30 days postoperatively. Initial analyses demonstrated low rates of accrual and retention for the VTE-PRO pilot. Therefore, the purpose of this study was to develop a knowledge translation (KT) intervention to improve VTE-PRO pilot trial accrual and retention.

**Methods:** Eligible participants were surveyed to identify the barriers to VTE-PRO participation. The Theoretical Domains Framework was used to categorize these barriers. Barriers were mapped to the COM-B Behavioral Change Wheel to identify potential interventions to support trial accrual and retention. The resulting KT intervention was titled IRIS-AR. Key informant interviews with patients were held to confirm the validity of identified barriers and perceived acceptability of the proposed IRIS-AR intervention. IRB approval was granted for this study.

**Findings:** The resulting intervention was comprised of: Information booklets and counseling sessions to identify unique participant challenges to trial participation (***I****nform*); daily reminders to administer injections (***R****emind*); involvement of family/caregivers in study processes (***I****nvolve*); and leverage of an existing Integrated Comprehensive Care (ICC) nursing program to provide injection support when needed (***S****upport*). Twenty-six key informants were interviewed. The most common barriers to trial participation included lack of social support and fear of needle injection. Participants generally supported use of information booklets, involvement of family/caregivers, and support by ICC nurses; however, they did not support the use of daily reminders.

**Implications for D&I Research:** The IRIS-AR presents a novel, patient-centred intervention that leverages existing programs to promote trial engagement. The proposed strategy can likely be adapted to improve compliance with other patient-directed interventions. We will pilot IRIS-AR to evaluate its impact on VTE-PRO trial accrual and retention rates.

### S45 Impact of an acute patient portal: Results of a randomized clinical trial

#### Fernanda Polubriaginof^1^, Beatriz Ryan^1^, Lisa Grossman^2^, Ruth Masterson Creber^2^, Min Qian^2^, Susan Restaino^2^, Suzanne Bakken^2^, George Hripcsak^2^, David Vawdrey^1^

##### ^1^NewYork-Presbyterian Hospital, New York, NY, USA; ^2^Columbia University Medical Center, New York, NY, USA

###### **Correspondence:** Fernanda Polubriaginof (fep7003@nyp.org)

**Background:** Millions of hospitalizations occur every year in the United States. Providing patients with access to their health information during the hospitalization increases transparency and may improve patient activation and engagement with health information. The purpose of this study was to determine the impact of an acute care portal on patient activation and patient engagement with health information.

**Methods:** We conducted a randomized clinical trial in two cardiac medical-surgical units at an academic medical center in New York City between March 2014 and May 2017. Participants (n=426) were randomized to one of three study arms: 1) usual care, 2) tablet with general internet access, and 3) tablet with access to the acute care portal. All participants received evidence-based medical treatment. Participants completed baseline and follow-up questionnaires to assess changes in patient activation (primary outcome). Secondary outcomes included the 30-day hospital readmission rate, engagement with health information and patient satisfaction. We also administered a survey to assess healthcare provider perceptions of usefulness and impact on care delivery.

**Findings:** There was no evidence of a difference in patient activation among patients assigned to the acute care portal intervention. There was evidence of a difference in patient engagement with health information between the acute care portal and tablet-only group, including better access to health information online (89.6% vs. 51.8%; p<0.001). Patients assigned to the acute care portal group had lower 30-day hospital readmissions (5.5% vs. 12.9% tablet-only and 13.5% usual-care; p=0.045). Patients and healthcare providers reported high satisfaction with the portal. Healthcare providers reported that patients found the portal useful and that the portal did not negatively impact healthcare delivery.

**Implications for D&I Research:** Access to an acute care portal was associated with lower 30-day hospital readmissions and improved patients’ access to their health information. Patients reported high satisfaction and healthcare delivery was not adversely impacted by hospitalized patients having access to an acute care portal. These results illustrate the value of providing hospitalized patients with real-time access to their electronic health record data. Next steps include conducting a formal Reach Effectiveness Adoption Implementation and Maintenance (RE-AIM) evaluation of the acute care portal to identify strategies for implementation across institutions.

### S46 Facilitating implementation of research evidence (FIRE): A randomised controlled trial and process evaluation of two models of facilitation informed by the promoting action on research implementation in health services (PARIHS) framework

#### Kate Seers^1^, Jo Rycroft-Malone^2^, Gillian Harvey^3^, Cox Karen^4^, Nicola Crichton^5^, Rhiannon Tudor Edwards^6^, Ann Catrine Eldh^7^, Carole Estabrooks^8^, Claire Hawkes^1^, Carys Jones^6^, Alison Kitson^9^, Brendan McCormack^10^, Christel McMullan^11^, Carole Mockford^1^, Theo Niessen^4^, Paul Slater^12^, Angie Titchen^12^, Teatske van der Zijpp^4^, Lars Wallin^13^

##### ^1^University of Warwick, Warwick, United Kingdom; ^2^Bangor University, Bangor, Gwynedd, United Kingdom; ^3^University of Adelaide, Adelaide, Australia; ^4^Fontys University of Applied Sciences, Eindhoven, Netherlands; ^5^London South Bank University, London, United Kingdom; ^6^Bangor University, Bangor, United Kingdom; ^7^Linkoping University, Linkoping, Sweden; ^8^University of Alberta, Edmonton, AB, Canada; ^9^Flinders University, Adelaide, Australia; ^10^Queen Margaret University Edinburgh, Edinburgh, United Kingdom; ^11^University of Birmingham, Birmingham, United Kingdom; ^12^Ulster University, Belfast, United Kingdom; ^13^Dalarna University, Falun, Sweden

###### **Correspondence:** Gillian Harvey (gillian.harvey@adelaide.edu.au)

**Background:** The PARIHS framework proposes that successful implementation of research evidence results from the complex interplay between the evidence to be implemented, the context of implementation and the facilitation processes employed. Facilitation is defined as a role (the facilitator) and a process (facilitation strategies/methods). Empirical evidence comparing different facilitation approaches is limited; this paper reports a trial of two different types of facilitation represented in the PARIHS framework.

**Methods:** A pragmatic cluster randomised controlled trial with embedded process evaluation was undertaken in 24 long-term nursing care settings in four European countries. In each country, sites were randomly allocated to standard dissemination of urinary incontinence guideline recommendations and one of two types of external-internal facilitation, labelled Type A and B. Type A facilitation was a less resource intensive approach, underpinned by improvement methodology; Type B was a more intensive, emancipatory model of facilitation, informed by critical social science. The primary outcome was percentage documented compliance with guideline recommendations. Process evaluation was framed by realist methodology and involved quantitative and qualitative data collection from multiple sources.

**Findings:** Quantitative data were obtained from reviews of 2313 records. Qualitative data included over 332 hours of observations of care; 39 hours observation of facilitation activity; 471 staff interviews; 174 resident interviews; 120 next of kin/carer interviews; and 125 stakeholder interviews. There were no significant differences in the primary outcome between study arms and all study arms improved over time. Process data revealed three core mechanisms that influenced the trajectory of the facilitation intervention: alignment of the facilitation approach to the needs and expectations of the internal facilitator and colleagues; engagement of internal facilitators and staff in attitude and action; and learning over time. Data from external facilitators demonstrated that the facilitation interventions did not work as planned, issues were cumulative and maintenance of fidelity was problematic.

**Implications for D&I Research:** Evaluating an intervention - in this case facilitation - that is fluid and dynamic within the methodology of a randomised controlled trial is complex and challenging. For future studies, we suggest a theoretical approach to fidelity, with a focus on mechanisms, as opposed to dose and intensity of the intervention.

### S47 Increasing clinician-initiated smoking cessation treatment: A randomized trial of implementation of EHR-based referral vs. fax-based referral to a tobacco quit line from primary care clinics

#### Mark Zehner, Rob Adsit, Danielle McCarthy, Timothy Baker, Michael Fiore

##### School of Medicine & Public Health, University of Wisconsin, Madison, WI, USA

###### **Correspondence:** Mark Zehner (mark.zehner@ctri.wisc.edu)

**Background:** Public Health Service Guidelines recommend using primary care encounters to increase engagement in evidence-based smoking cessation treatment for the nearly 70% of smokers who visit their doctor in a given year. Recent expansion of EHR adoption has increased assessment and recording of smoking status, but clinicians often fail to respond to this vital sign by providing assistance in quitting. Interoperable EHR platforms enabled with automated best practice prompts, guided workflows, and integrated electronic referral orders implemented with professional training, hold promise to expand the delivery of smoking cessation treatment via primary care.

**Methods:** Rate of referral of adult patients who smoked to the Wisconsin Tobacco Quit Line (WTQL) was the primary outcome. Twenty-three primary care clinics from two regional healthcare systems were randomized to either usual care (paper-based Fax to Quit [F2Q] referral) or to an entirely EHR-based referral approach (eReferral). Each clinic received a 30-minute training session focused on increasing clinician offers of referral to WTQL tobacco cessation treatment. Clinic staff were trained to engage smokers in WTQL services by either faxing paper referral forms (F2Q) or eReferral. Data were extracted from each health system’s EHR for six months following their training along with summary enrollment data from the WTQL.

**Findings:** During the observation period, 3,020 adult patients were documented as smokers in F2Q clinics and 3,415 in eReferral clinics. In both healthcare systems, clinicians in eReferral clinics referred significantly more adult smokers to the WTQL than did those in F2Q clinics (Healthcare System A: 17.9% of smokers referred via eReferral versus 3.8% via F2Q, p < 0.001; Healthcare System B: 18.9% of smokers referred via eReferral versus 5.2% F2Q, p < 0.001).

**Implications for D&I Research:** eReferral to smoking cessation quitline treatment was highly feasible and markedly increased referrals versus the previous standard of care, fax-based referrals. Incorporating EHR-based alerts, auto-populated forms, and guided workflows along with integrated (interoperable) records exchange between the clinic and the quit line provide implementation advantages for delivering guideline recommended care.

### S48 Innovation is not a guarantee of implementation success: Clinical nurses adhere more to a standard education strategy than to gamification

#### Heather Greysen, Ryan Greysen, Mary Naylor, Roy Rosin, Meghan Lane-Fall

##### University of Pennsylvania, Philadelphia, PA, USA

###### **Correspondence:** Heather Greysen (hgreysen@upenn.edu)

**Background**: Communication of mobility goals is an evidence-based practice (EBP) known to improve patient outcomes. Nurses may not communicate mobility goals to hospitalized patients because of time constraints and role identity. The MobiliLand study is a CFIR-informed prospective mixed-methods study of two complementary implementation strategies to facilitate uptake of this EBP; one strategy is a standard approach using educational pamphlets and the other is an innovative communication tool based on principles of behavioral psychology and gamification. The goal of this study was to assess nurse use and acceptance of the two approaches.

**Methods**: (Pre-study contextual inquiry indicated that nurses did not feel empowered to educate patients about mobility.) On a cardiology ward in an academic hospital, we instructed nurses to communicate mobility goals by (1) using the standard communication approach of providing patients with an educational pamphlet and (2) engaging patients in the use of a game designed to educate and engage the patient in appropriate mobility goals. Fidelity was defined as the nurse’s adherence to each implementation strategy and was characterized with descriptive statistics. Nurse interviews were conducted to elicit barriers and facilitators of adherence to and acceptance of the strategies. Qualitative data were analyzed using grounded theory.

**Findings:** Fidelity to the standard communication approach using pamphlets was (164/240) 68.3% and to the gamified approach was (19/240) 7.9%. We conducted 11 nurse interviews. All of the nurses agreed that patient communication about mobility was important; however, the gamified mobility communication strategy required more investment of nurse time than anticipated due to patient questions. Nurse perceived facilitators to use of the educational pamphlet to communicate mobility goals included: ease of strategy fit within work flow; simplicity of strategy; barriers to use of the gamified mobility communication strategy included: lack of time, competing priorities, unfamiliarity with implementation strategy.

**Implications for D&I Research**: While gamification is a novel strategy, its unfamiliarity and lack of compatibility with workflow present challenges to its effective use. Future research is needed to determine how to incorporate innovative strategies such as gamification into clinical implementation efforts.

### S49 Scaling out ehealth for HIV prevention: Two hybrid trials of programs for young gay/bisexual men

#### Brian Mustanski, Thomas Remble, Krystal Madkins

##### Northwestern University, Chicago, IL, USA

###### **Correspondence:** Brian Mustanski (brian@northwestern.edu)

**Background:** eHealth interventions have recently proven to be efficacious at reducing HIV risk among adolescent and young men who have sex with men (YMSM). Very little is known about how to scale-up eHealth HIV-prevention efforts as their delivery does not conform to traditional CBO HIV prevention service infrastructures. The aim of this talk is to present study designs that can answer important implementation questions for eHealth HIV interventions.

**Methods:** I will present two case studies of eHealth HIV prevention interventions being delivered nationally to YMSM. The first study, SMART, is a type I effectiveness–implementation hybrid trial targeted toward 13-18 year olds. SMART uses a sequential multiple assignment randomized trial design to test the effectiveness of a stepped care package of increasingly intensive intervention for YMSM while gathering information to inform future implementation. The second study, Keep It Up! (KIU!), is a type III effectiveness–implementation hybrid design targeted to YMSM 18-29 years old. This implementation trial uses a cluster-RCT design to compare two implementation strategies (direct to consumer vs. uptake and use by CBOs) while gathering information and test results to confirm its effectiveness.

**Findings:** In SMART, the primary effectiveness outcome is condomless anal sex. Implementation outcomes include constructs of the RE-AIM model and collection of cost effectiveness data. Data on implementation readiness, barriers and facilitators, reach, effectiveness; and costs associated with start-up, ongoing delivery, and program sustainment will provide critical information on how to implement the SMART Program. In KIU!, we are using mixed-methods to capture multiple metrics of adoption and implementation, while outcomes are focused on reach, uptake, AND effectiveness comparing 2 approaches to content-delivery, as well as sustainability. We will discuss a novel modeling approach for a single primary outcome that integrates information across reach and effectiveness.

**Implications for D&I Research:** Hybrid, adaptive, and cluster trials are well suited to test the implementation strategies for eHealth interventions. The biggest challenge we face in eHealth interventions are the ever-changing technology landscape and lack of community capacity for eHealth. These two cased studies illustrate examples of hybrid trials that either prioritize collection of effectiveness or implementation outcomes.

### S50 The southern initiative: Implementing a community health worker model to improve HIV care and prevention services among minority populations in four southern states

#### Lindsay Senter, Vanessa Arenas, Emily Leung, Dawn Middleton, Gretchen Weiss

##### Cicatelli Associates Inc (CAI), New York, NY, USA

###### **Correspondence:** Lindsay Senter (lsenter@caiglobal.org)

**Background:** Funded by the Secretary’s Minority AIDS Initiative Fund and HRSA’s HIV/AIDS Bureau, *The Southern Initiative* aims to improve HIV care among priority populations served by organizations in four Ryan White HIV/AIDS Part A Jurisdictions (Atlanta, Houston, Memphis, and New Orleans). The National Association of County and City Health Officials (NACCHO) and Cicatelli Associates Inc. (CAI) serve as the Coordination and Technical Assistance Center (CTAC) to support agencies’ implementation of an evidence-based Community Health Worker (CHW) care integration model to increase retention and ART adherence.

**Methods:** CTAC provides training and technical assistance (TTA) to 2 FQHCS and 2 ASOs Part A recipients to facilitate implementation of the CHW model using the EPIS framework. For the **Exploration** phase (March-November 2017), CTAC conducted site-specific assessments to assess readiness for CHW model implementation using Hexagon Tool and the Consolidated Framework for Implementation Research, and developed TTA structures and tools to support the **Preparation** phase. During the **Implementation and Sustainability** phases (December 2017-Present), CTAC uses blended TTA approaches (cluster face-to-face TTA sessions, monthly CHW/CHW Supervisor community of practice sessions (CoPs), and practice facilitation coaching). Evaluation data from CHW-Client Encounter Forms (CEF), clinical records, staff surveys, and agency implementation plan/dashboards, are reviewed during TTA to inform progress. Implementation and clinical outcomes include: unique clients served, encounters/client, staff participation/satisfaction, leadership engagement, and client viral load.

**Findings:** CEF data reveal increases in clients served and encounters, and improved identification of barriers impacting retention, indicating enhanced delivery of services to address complex issues. At baseline, CHWs saw 22 unique clients through 40 encounters (average 1.8 encounters/client); by month 5, 146 unique clients and 652 encounters (average 4.5 encounters/client). CTAC completed 52 coaching sessions with change-agents; 9 CoPs with CHW Supervisors (91% attendance rate) and 12 CoPs with CHWs (80% attendance rate) with high satisfaction in post-CoP surveys. Review of agencies’ customized plans/dashboards, indicate strong buy-in from staff and leadership for sustainment of the CHW model.

**Implications for D&I Research:** This initiative demonstrates the promise and utility of applying implementation science theories and frameworks to address health disparities in HIV; an area needing further development in HIV care and prevention.

### S51 National implementation of evidence-informed interventions for people living with HIV across 26 sites

#### Alex Keuroghlian (akeuroghlian@partners.org)

##### The Fenway Institute, Boston, MA, USA

**Background:** This session will explore national implementation of evidence-informed interventions for people living with HIV (PLWH) through the new HRSA HIV/AIDS Bureau (HRSA/HAB) initiative entitled *Using Evidence-Informed Interventions to Improve Health Outcomes among People Living with HIV* (E2i). Through E2i, HRSA/HAB aims to improve HIV outcomes (e.g., viral suppression, retention in care) by conducting rapid and sustainable implementation, and rigorous evaluation, of effective and culturally tailored interventions for PLWH in four focus areas: transgender women, Black men who have sex with men, behavioral health integration into primary medical care, and identifying and addressing trauma.

**Methods:** The E2i initiative utilizes the strategies described by Proctor and colleagues to idenitify implementation strategies that mediate the effect of select interventions on client-level outcomes in Ryan White-funded agency settings. We will discuss strategies for rapid and sustainable implementation of evidence-informed interventions for PLWH, specifically including the following interventions: (1) *transgender women*: Healthy Divas; Transgender Women Engagement and Entry to Care Project (TWEET), (2) *Black men who have sex with men*: Motivational Interviewing Peer Outreach; Project Connect; Retention through Enhanced Contacts and Text Messaging Intervention to Improve Antiretroviral Adherence among HIV-Positive Youth (TXTXT), (3) *behavioral health integration into primary care*: Clinic-based Buprenorphine Treatment; Psychiatric Collaborative Care Management (CoCM); Screening, Brief Intervention, and Referral to Treatment (SBIRT), and (4) *identifying and addressing trauma*: Trauma Informed Approach & Coordinated HIV Assistance and Navigation for Growth and Empowerment (TIA/CHANGE); Cognitive Processing Therapy; Seeking Safety.

**Findings:** The workshop will present results from a 26-site organizational assessment identifying needs across the four focus areas and strategies to improve the implementation of evidence-informed interventions across a broad range of Ryan White HIV/AIDS Program grantees. These examples can help inform when, where, how, by whom, and under what circumstances we can promote the translation of evidence that takes into account the needs and knowledge of community-based organizations and clinical partners responsible for services at the local level.

**Implications for D&I Research:** We discuss how these D&I examples can be used to translate evidence into practice, increase adoption and reach of interventions in local areas, and optimize sustainability.

### S52 Automated text messaging with patients in VHA specialty clinics: A hybrid type 2 effectiveness-implementation study

#### Vera Yakovchenko^1^, Dr. Timothy P. Hogan^2,3,4^, Thomas Houston^3,5^, Lorilei Richardson^4^, Beth Ann Petrakis^4^, Chris Gillespie^4^, Jessica Lipschitz^6,7^, D. Keith McInnes^4,8^

##### ^1^BridgeQUERI, CHOIR, Veterans Health Administration, Bedford, MA, USA; ^2^Edith Nourse Rogers Memorial Veterans Hospital, Veterans Health Administration, Bedford, MA, USA; ^3^University of Massachusetts Medical School, Worcester, MA, USA; ^4^CHOIR, Veterans Health Administration, Bedford VA Medical Center, Bedford, MA, USA; ^5^Bedford & Boston VA Center for Healthcare Organization & Implementation Research, Veterans Health Administration, Bedford VA Medical Center, Bedford, MA, USA; ^6^Brigham and Women’s Hospital, Boston, MA, USA; ^7^Harvard Medical School, Boston, MA, USA; ^8^Boston University School of Public Health, Boston, MA, USA

###### **Correspondence:** Vera Yakovchenko (vera.yakovchenko@va.gov)

**Background:** Mobile phone texting is rapidly becoming an accepted means of asynchronous communication between healthcare systems and patients. The Veterans Health Administration (VHA) seeks to nationally deploy an automated texting system to promote patient self-management. To date, however, few trials have evaluated implementation strategies to support the adoption of such technology and engage patients and their clinical teams in its use. Guided by the Practical, Robust, Implementation and Sustainability Model (PRISM), we conducted a mixed-methods, randomized, effectiveness-implementation hybrid type 2 study to compare the effectiveness of usual facilitation versus augmented facilitation to support uptake of VHA’s new texting system.

**Methods:** Seven VHA hepatitis C virus (HCV) specialty clinics were randomized to usual or augmented facilitation and two comparison clinics did not receive either the texting system or facilitation. Implementation outcomes included: acceptability, adoption, feasibility, fidelity, and sustainability. Effectiveness outcomes included: patient engagement, self-reported medication adherence, health perceptions and behaviors, and sustained virologic response (SVR) indicating HCV “cure.”

**Findings:** *Implementation*: There were 293 facilitation events conducted with clinics, using a core set of 12 facilitation-based implementation strategies including developing and distributing an implementation toolkit, preparing champions, and tailoring the texting system to local context. The texting system was largely accepted, deemed appropriate, easy to use, and helpful for patients and clinicians. Nevertheless, there was a substantial patient enrollment gap at all clinics (33%, 65/197 were enrolled). Enrollment challenges included low perceived need for self-management support, clinician misalignment of expectations, motivations and efforts, and clinic workflow incompatibility. *Effectiveness*: Once enrolled, more patients at augmented than usual clinics adopted the texting system (83% vs 61%, p=0.038) and had sustained use (13% vs 4%, p=0.045). Patients who texted self-reported less distress about failing HCV treatment (89% vs 36%, p=0.003), less frequently forgetting to get bloodwork drawn (84% vs 36%, p=0.027), and excellent adherence to HCV medication (77% vs 30%, p=0.023). SVR between facilitation arms and between patients texting and not texting were not different.

**Implications for D&I Research:** Augmented facilitation resulted in greater sustained engagement and an indication of improved process and clinical outcomes. While novel technologies like texting systems have considerable potential, behavioral, social and technical factors present scale-up challenges.

### S53 Vetconnect: Victories and pitfalls implementing telehealth between VA and non-VA facilities

#### Anne Hale^1^, Leah Haverhals^1,2^, Cari Levy^1^

##### ^1^Seattle-Denver Center of Innovation, Veterans Health Administration, Denver, CO, USA; ^2^Rocky Mountain Regional VA Medical Center, Denver, CO, USA

###### **Correspondence:** Anne Hale (anne.hale@va.gov)

**Background:** Veterans residing in Department of Veterans Affairs (VA) contracted community nursing homes (CNHs) and state Veterans nursing homes (SVHs) receive primary care from CNHs and SVHs, but travel to Veterans Affairs Medical Centers (VAMCs) for specialty care. This project aimed to improve access to specialty care for these Veterans in Colorado and Oklahoma through implementing a telehealth program. The VetConnect program facilitates video rather than in-person visits for Veterans with VA providers, to overcome barriers such as long travel or wait times. Specialties provided include geriatrics, mental health, wheelchair clinic, and palliative care.

**Methods:** Multidisciplinary VA project staff (nurses, physicians, social workers, researchers, and facility teleheath coordinators) partnered with staff at CNHs and SVHs for program implementation. We conducted in-person visits in Colorado and Oklahoma to generate interest and buy-in, and held regular weekly in-person and monthly phone project meetings, allowing processes to develop and adapt. This facilitated program uptake and increased collaboration with CNHs and SVHs, allowing for troubleshooting to overcome challenges. We assessed the following implementation metrics: number of visits conducted; percentage of technologically successful visits; process map analysis; qualitative interviews of Veterans and VA and CNH staff; field notes from project nurses; and cost savings analyses to determine feasibility and quality of visits.

**Findings:** Video visits have been conducted with Veterans in nine CNHs and two SVHs in Colorado, and five SVHs in Oklahoma. Beginning in June 2017, Colorado has conducted N=306 visits (in Geriatrics, Mental Health, and Palliative Care) with an 84% success rate (based on dropped calls). In Oklahoma, N=182 visits (in Wheelchair, Psychiatry, and Social Work) have occurred. Successful implementation strategies include: cultivating buy-in from key players (providers, telehealth staff, and CNH/SVH staff), rapidly adapting per unique situations (adjusting technology used and adding a team nurse, providers, and services), and ongoing communication targeting challenges and new program applications.

**Implications for D&I Research:** Implementation of VetConnect increased access to specialty care for nursing home Veterans and has potential for nation-wide expansion to other VAMCs. Assessment of implementation of Vet Connect can provide useful insights for healthcare systems outside the VA desiring to provide similar services.

### S54 The effectiveness of replicating effective programs (REP) as a strategy for implementing the diabetes prevention program for women veterans: A mixed-method evaluation

#### Erin Finley^1^, Tannaz Moin^2^, Bevanne Bean-Mayberry^3^, Jessica Moreau^3^, Karen Dyer^2^, Melissa Farmer^3^, Alison Hamilton^4^

##### ^1^South Texas VA Healthcare System, Veterans Health Administration, San Antonio, TX, USA; ^2^VA Greater Los Angeles, Veterans Health Administration, Los Angeles, CA, USA; ^3^VA Greater Los Angeles, Veterans Health Administration, North Hills, CA, USA; ^4^UCLA, Los Angeles, CA, USA

###### **Correspondence:** Erin Finley (erin.finley@va.gov)

**Background:** Although Replicating Effective Programs (REP) was developed as a framework to guide tailoring and implementation of evidence-based interventions in the 1990s, relatively few studies have assessed its effectiveness as an implementation strategy. In 2016-2018, we followed the REP framework to tailor and implement the Diabetes Prevention Program (DPP) for women Veterans, as part of a VA QUERI-funded quality improvement project in VA Greater Los Angeles women's primary care (PC) clinics. We conducted a mixed-method program evaluation to assess patient engagement, weight loss, and satisfaction associated with use of REP to implement the tailored DPP program.

**Methods:** We screened electronic medical record data for women veterans with prediabetes, identifying 541 women veterans potentially eligible for DPP. We reached 302 veterans by phone and offered a choice between participating in (a) peer-led women-only groups or (b) an online version of the intervention. Patient and implementation outcomes were assessed using contact tracking, patient semi-structured interviews at baseline and follow-up, and attendance and weight assessments collected as part of DPP sessions.

**Findings:** 216 (72%) of women veterans reached by phone expressed interest in tailored DPP. More women favored online DPP (N=160, 74%) than in-person, peer-led DPP (N=51, 24%). 119 women were enrolled between June 2016-March 2017. In peer-led DPP, 25% of women completed 9+ sessions/modules, with mean weight loss of 1.8lbs (as treated). In online DPP, 64% of women completed 9+ sessions/modules, with mean weight loss of 7 lbs (as treated). Both completers and non-completers reported increased knowledge regarding prediabetes and effective management (i.e., diet and exercise) and high satisfaction with program participation.

**Implications for D&I Research:** Tailoring DPP intervention delivery to better meet the needs of high-risk sub-groups may help to extend the reach of ongoing national efforts to prevent type 2 diabetes. Following the REP framework throughout this two-year quality improvement study resulted in delivery of a tailored DPP intervention to a higher-than-anticipated number of women Veterans, showing feasibility for delivery in VA PC settings and resulting in increased knowledge and weight reduction among participants in both conditions, despite greater engagement in online DPP. These findings suggest REP has utility in achieving effective implementation of tailored interventions.

### S55 Lessons learned from the field: Audit and feedback with rapid prototyping helped redesign a care coordination program during scale-up

#### Lindsay Miller^1^, Tuula Kallioniemi^1^, Roman Ayele^2^, Marina McCreight^2^, Ashlea Mayberry^2^, Heidi Sjoberg^1^, Catherine Battaglia^3,4^

##### ^1^Denver-Seattle Center of Innovation, Veterans Health Administration, Aurora, CO, USA; ^2^VA Eastern Colorado Healthcare System, Veterans Health Administration, Aurora, CO, USA; ^3^Denver VA Medical Center, Veterans Health Administration, Denver, CO, USA; ^4^Colorado School of Public Health, Denver, CO, USA

###### **Correspondence:** Lindsay Miller (lindsay.miller8@va.gov)

**Background:** Veterans are accessing healthcare services across healthcare systems, at both the Veterans Health Administration (VA) and non-VA community hospitals. To ensure continuity of care, we implemented a nurse-led Community Hospital Transitions Program (CHTP) to facilitate the transitions from community hospitalizations back to VA primary care for these “dual-use” Veterans. Our objective is to share lessons learned using audit and feedback with rapid prototyping to facilitate the recalibration of a transitions of care intervention.

**Methods:** Rapid scale-up of the CHTP created barriers to implementation, including difficulties completing the intervention and lack of engagement in targeted community hospitals. To overcome these barriers, we recalibrated the intervention based on audit and feedback with key stakeholders and guided by the Lean method of *Define*, *Measure*, *Analyze*, *Improve*, *Control.* We performed workload assessment of the Community Hospital Transitions Nurse (CHTN) supported by process observations and rapid prototyping to change the scope of the CHTP to ensure appropriate implementation and sustainability.

**Findings:** During implementation, ongoing communication with the implementation team and with VA and community stakeholders indicated a need to recalibrate the scope of the CHTP as well as the role of the CHTN. Lessons learned from audit and feedback with rapid prototyping helped redesign the CHTP and clearly define the intervention core components, which now include: 1) Notification of community hospitalization, 2) Information transfer, 3) Follow-up coordination, 4) Hand-off to VA PCP. We scaled back the CHTP from serving 37 down to 5 high-volume community hospitals and worked to identify which dual-use Veterans were most vulnerable and needed care coordination during the transitions process. Since the program rolled out in June 2017, the CHTP has touched over 1465 Veterans. After recalibration, the number of new notifications decreased by 51.2% and time to complete the intervention decreased by 77.6%. The percentage of Veterans completing all four core components increased by 4.8%.

**Implications for D&I Research:** Continuous audit and feedback indicated that rapid expansion jeopardized the CHTP impact. Scaling back our intervention allowed more manageable workflow and improved program implementation outcomes.

### S56 Increasing access to oral health education and dental care through Michigan WIC

#### Holli Seabury (hseabury@mcmillenhealth.org)

##### McMillen Health, Fort Wayne, IN, USA

**Background:** The Special Supplemental Food Program for Women, Infants and Children (WIC) provides an ideal location to educate parents about the importance of oral health and dental visits for their young children. WIC serves low-income families whose children are at higher risk for dental decay. However, WIC staff often do not feel as though they have been properly trained to give oral health guidance, nor do they feel they have the appropriate educational resources. Children enrolled in the WIC program are predominately enrolled in Medicaid, and have fewer dental visits than children covered by private insurance.

**Methods:** McMillen Health partnered with Michigan WIC and Altarum to implement an oral health pilot project in Detroit. Staff in five WIC clinics—serving 23,000 children, or 10% of the state’s WIC participation, received a two-hour training to integrate oral health education and dental referrals into the nutrition education provided to mothers with young children. Staff were given McMillen’s teaching flip chart, parent handouts, and other teaching resources to initiate parent-led discussions on oral health. WIC also changed their tracking system to include oral health education and dental referrals and visits.

**Findings:** This was the first oral health training for 95% of WIC staff. Staff demonstrated increased knowledge regarding the recommended age for a child's first dental visit (43% to 95%). Staff showed increased comfort discussing dental issues (36% to 86%). One hundred percent of WIC staff said they would recommend the training to a colleague. Dental referrals were made for over 1,000 children under 5 years old. Results after linking WIC and Medicaid data, showed 25.1% of WIC pilot participants had a dental visit compared to an 18.2% dental visit rate for matched controls; an increase of 38%.

**Implications for D&I Research:** Increasing dental visits and home hygiene is critical to increasing oral health status in children. This intervention required little time and effort on behalf of staff and has the potential of reaching WIC sites nationwide. The project has since expanded to 4 additional Michigan counties, with another proposal submitted to expand into rural areas (reaching an additional 30% of Michigan WIC participants).

### S57 Predicting user engagement with daily interactive text messages in a diabetes self-care support intervention

#### Lyndsay A. Nelson, Michael T. Ackerman, Robert A. Greevy, Jr., Kenneth A. Wallston, Lindsay S. Mayberry

##### Vanderbilt University Medical Center, Nashville, TN, USA

###### **Correspondence:** Lyndsay A. Nelson (lyndsay.a.nelson@vanderbilt.edu)

**Background:** Evidence for efficacy of text messaging interventions to improve self-management of chronic conditions is accumulating, but little is known about user engagement with these interventions. Examining patterns and predictors of engagement can support scale-up and implementation by informing which users will engage with text messaging programs and for how long. We examined user engagement with daily text messages asking about diabetes medication adherence for 6 months.

**Methods:** We recruited adults with type 2 diabetes from Federally Qualified Health Centers and an academic medical center. Participants self-reported demographics, health literacy, numeracy, and medication adherence (Adherence to Refills and Medications Scale for Diabetes), and completed a hemoglobin A1c test. Participants were assigned to receive text messages alone or with phone coaching for 6 months. We modeled text message response rates throughout the intervention and examined unadjusted associations between patient characteristics and response rates with 95% confidence intervals.

**Findings:** The sample (N=245) had a mean age of 55.7 ± 9.8 years, was 55% female, and 39% Black. Forty-one percent had ≤ a high school degree, 41% had incomes <$25K, and the mean A1c was 8.6 ± 1.8%. On average, participants responded to 84% (IQR: 80-97%) of the text messages. Average response rate was stable around 89% throughout the first 4 months, but began to wane after that, approaching 76% at the end of 6 months. Response rates were 7% (1.4, 12.1) lower among Black participants compared to White participants. For every standard deviation increase in baseline adherence, engagement was 6% higher (3.3, 8.2), and for every 1.0% increase in baseline A1c (e.g., 8.0 to 9.0%), engagement was 2% lower (-3.2, -0.5). Participants’ age, gender, education, income, diabetes duration, health literacy, numeracy, clinic site, and assigned condition were not associated with engagement.

**Implications for D&I Research:** Findings support uptake and sustainability of text messaging interventions for at least 6 months among socioeconomically diverse patients. Engagement was high regardless of patients’ age, education, income, health literacy, clinic site, and receipt of phone coaching. Disparities in engagement by race, medication adherence, and A1c were relatively small but potential customizations focused on these differences could improve engagement.

### S58 Shared decision making in routine clinical use may not result in fewer surgeries among hip and knee osteoarthritis patients

#### Vanessa Hurley^1^, Hector Rodriguez^2^, Yue Wang^3^, Stephen Shortell^2^, Ming Leung^4^

##### ^1^Georgetown University, Washington, DC, USA; ^2^University of California - Berkeley, School of Public Health, Berkeley, CA, USA; ^3^Center for Healthcare Organizational and Innovation Research, University of California, Berkeley, Berkeley, CA, USA; ^4^Haas School of Business, University of California, Berkeley, Berkeley, CA, USA

###### **Correspondence:** Vanessa Hurley (vh151@georgetown.edu)

**Background:** Hip and knee osteoarthritis are among the most prevalent and quickly growing chronic conditions within the United States. Consequently, knee and hip replacements are among the most commonly performed orthopedic procedures in the U.S. and frequently appear among near the top of lists of the most prevalent and costly procedures for both commercially and publicly insured patients. We study whether the use of Decision Aids (DAs) in the context of shared decision-making (SDM) is associated with lower propensity for hip or knee osteoarthritis patients to receive surgery within 6 months compared with an unexposed comparison group of patients receiving care in the same health care systems.

**Methods:** We leveraged data from patients with hip and knee osteoarthritis within High Value Healthcare Collaborative (HVHC) systems between 2012-2015. Optimal variable propensity-score matching followed by multivariate logistic regression estimated the relationship between DA exposure and surgical utilization, controlling for patient characteristics.

**Findings:** The use of Decision Aids (DAs) in the context of shared decision-making (SDM) was not associated with lower propensity for hip or knee osteoarthritis patients to receive surgery within 6 months compared with an unexposed group of patients. In adjusted models, knee patients who were exposed to the SDM intervention had greater odds of undergoing surgery compared with unexposed knee patients (OR = 1.24, p<0.001), as did hip patients (OR = 2.59, p<0.001). Although knee patients with diabetes had lower odds of undergoing arthroplasty compared to patients not living with diabetes (OR = 0.84, p<0.05), knee patients with depression had one and a half times the odds of having surgery compared with patients without depression (OR = 1.59, p<0.001). Hip and knee patients with depression had twice the odds of having surgery (OR = 2.36, p<0.001 and OR = 2.12, p<0.001, respectively).

**Implications for D&I Research:** SDM using DAs in routine practice settings may not sway hip and knee patients toward more conservative treatment modalities, on average. When DAs are used as part of SDM in routine care settings, health care payers and administrators should not expect reduced surgical utilization.

### S59 Observation of real-time communication and behaviors during implementation of an interprofessional bedside rounding model

#### Jing Li^1^, Kevin Real^1^, Sarah Bell^1^, Preetham Talari^2^, Barbara Latham^2^, Mark Williams^1^

##### ^1^University of Kentucky, Lexington, KY, USA; ^2^University of Kentucky HealthCare, Lexington, KY, USA

###### **Correspondence:** Jing Li (jingli.tj@uky.edu)

**Background:** Observation of real-time communication and behaviors during the implementation of an interprofessional bedside rounding model is a valuable tool for understanding how innovations are sustained within hospital settings. This study observed interdisciplinary team rounding behaviors and communication processes in the Interprofessional Teamwork Innovation Model (ITIM) as teams rounded on patients in a hospital medical floor. ITIM teams were comprised of patient/family, hospitalist, bedside nurse, pharmacist, and case manager. ITIM rounds follow a tested, structured approach to improve information sharing, engagement, and interprofessional communication. Our analysis draws upon systems theory for understanding how structural, process and outcome factors drive implementation in complex healthcare environments.

**Methods:** This study was conducted at a 30-bed general medical unit within a 302-bed community-based hospital in an academic health system. The research team observed over 120 hours of interprofessional rounds to 405 patients by 47 interprofessional teams. Observational measures included structural factors (e.g., information sharing) and process factors (team communication). Patient outcomes included length of stay (LOS), 30-day same-hospital readmission, and patient satisfaction. LOS and readmission data were obtained from the enterprise data warehouse and patient satisfaction was assessed by a same-day 17-item survey. Statistical analysis of observational data employed a general linear model approach.

**Findings:** Correlational analysis revealed that general plan-of-care review was negatively related to LOS (r=-.16, *p*<.001) and positively associated with patient satisfaction (r=.31, *p*<.05) and patients’ perceptions of their ITIM team’s communication (r=.26, *p*<.10) and concern (r=.29, *p*<.05). Readmission was negatively associated with team communication (r=-.11, *p*<.05). Results from hierarchical linear regression with LOS as dependent variable indicate significant relationship with structural factors (plan-of-care and discharge reviews) and process factors (team communication and rapport building).

**Implications for D&I Research:** This research suggests several important implications for implementation research in healthcare contexts. First, structured implementation defines clear roles and responsibilities, can enhance fidelity and lead to desired intervention delivery to patients. Second, there are several components during the rounds may be more important than others for better outcomes. Third, when there is an interactive plan of care discussion among team members, patients perceive as effective team-oriented communication. The structure-process-outcome can be an effective model in evaluating implementation of innovations.

## Clinical Care Settings: System-level Interventions

### S60 Testing two implementation strategy bundles for addressing site developed quality improvement plans in juvenile justice settings: Adaptive system change efforts

#### Jennifer Becan^1,2^, Danica Knight^1^, Ricky Valdes Velasco^1^, Tisha Wiley^3^

##### ^1^Institute for Behavioral Research, Texas Christian University, Fort Worth, TX, USA; ^2^Karyn Purvis Institute of Child Development, Texas Christian University, Fort Worth, TX, USA; ^3^National Institute on Drug Abuse, Bethesda, MD, USA

###### **Correspondence:** Jennifer Becan (j.becan@tcu.edu)

**Background:** Juvenile justice departments strive to meet the substance use service needs of youth, which often requires referral to external behavioral health (BH) providers. In multi-agency collaboratives such as these, system change often occurs in a non-linear manner, requiring ongoing assessment and modification of initial plans. The JJ-TRIALS protocol uses data-driven decision making (DDDM) strategies to facilitate change, and offers a rare exploration of the execution of quality improvement plans. The Behavioral Health Services Cascade (Cascade) and the Dynamic Adaptation Process model (DAP; based on EPIS), serve as theoretical frameworks for tailoring system change efforts within diverse systems of care. Primary questions are: Compared to Core strategies, are Enhanced strategies more effective in promoting improvement in quality indicators along the cascade and recursive use of DDDM?

**Methods:** Using a delayed-start cluster randomized trial, 36 community supervision agencies from 7 states were randomly assigned to one of two conditions: *Core* (training on DDDM use) or core plus *Enhanced* strategies (expert facilitation of local change teams in using data-driven decision making). Interagency workgroup efforts to address their identified service gaps and their use of JJ-TRIALS implementation strategies were documented via phone interviews with site liaisons across 18 months. The Cascade served as a measurement model to uniformly align goals and steps to best practices across the diverse systems.

**Findings:** Data from 609 interviews indicate that Enhanced sites more actively addressed three Cascade domains: screening, referral, and initiation. Enhanced sites also exhibited greater use of adaptive strategies while working toward their goal (e.g., modifying their implementation plans, using DDDM) and were more likely to strengthen their capacity to use DDDM through improvements to their management information systems and communication practices with BH partners.

**Implications for D&I Research:** The current study advances implementation science through development of new measurement approaches that map onto existing theoretical frameworks (Cascade, DAP) and documents specific ways in which Enhanced approaches (i.e., facilitation of DDDM) promotes the implementation of site-identified goals. Future work is needed linking progress toward goals with service outcomes on closing targeted service gaps (using administrative youth records).

### S61 Main results from a cluster randomized trial testing the effectiveness of implementation strategies to improve adherence to tobacco treatment guidelines in public health dental care

#### Jamie Ostroff^1^, Donna Shelley^2^, Danielle Khalife^1^, Yuelin Li^1^, Elizabeth Schofield^1^, Christina Kyriakos^2^, Alena Campo^2^, Sarah Borderud^1^

##### ^1^Memorial Sloan Kettering Cancer Center, New York, NY, USA; ^2^New York University School of Medicine, New York, NY, USA

###### **Correspondence:** Jamie Ostroff (ostroffj@mskcc.org)

**Background:** Despite American Dental Association recommendations, national surveys demonstrate that tobacco use assessment and treatment (TUT) has not been integrated into routine dental care.

**Methods:** We conducted a cluster randomized controlled trial evaluating the effectiveness of systems-level implementation strategies for improving delivery of TUT in public health dental clinics. Guided by the Theoretical Domains Framework, 2,743 patient exit interviews were collected from 18 dental clinics, serving predominantly low income smokers, randomized to one of three intervention conditions: Current Best Practices (CBP) (i.e. staff training, clinical reminder system, and Quitline referral workflow); CBP + Performance Feedback (PF) (i.e. quarterly feedback on provider delivery of TUT); and CBP + PF + Pay-for-Performance (P4P) (i.e. financial incentives for TUT delivery). Patient surveys conducted at the point of service assessed dental providers’ TUT behaviors (primary outcome) at baseline and 9 months following study enrollment. Nine TUT behaviors were assessed: asked about current smoking status (ask), advised current smokers to quit (advise), assessed smokers’ readiness to quit (assess), provided brief cessation counseling, gave written information about tobacco cessation, discussed/prescribed cessation medications (assist), and referred smokers for tobacco treatment such as Quitline (arrange). Dental provider surveys (n=476) assessed pre-, post-changes in organizational priority. Site-specific focus groups assessing barriers/facilitators, acceptability and sustainability of TUT delivery were also conducted.

**Findings:** All three implementation strategies demonstrated significant improvement in TUT delivery over time with the most sizable increase observed in prescription or recommendation for cessation medication (OR = 1.98, CI = [1.17, 3.34]. With the exception of Ask (p=.03), there were no significant intervention effects found. There was a statistically significant (p < .05) interaction found between intervention condition and change in organizational priority, with PF + P4P demonstrating the greatest improvement. Organizational priority was a significant robust predictor of improvement in TUT delivery.

**Implications for D&I Research:** Staff training, clinical reminder system and establishing clinic workflow for referring current smokers for treatment improves TUT delivery. Audit and performance feedback may also improve the assessment of tobacco use in dental care settings. Changes in organizational priority may be driving mechanism by which these implementation strategies improve quality of TUT delivery.

### S62 Implementation strategies associated with stronger implementation of a patient safety bundle to reduce primary cesarean delivery rates at Maryland hospitals

#### Jennifer Callaghan-Koru^1^, Andreea Creanga^2^, Bonnie DiPietro^3^, Katrina Mark^4^, Ardy Sowe^5^, Nour Aboumatar^6^, Ann Burke^7^, Geoffrey Curran^8^

##### ^1^University of Maryland-Baltimore County, Baltimore, MD, USA; ^2^Johns Hopkins Bloomberg School of Public Health, Baltimore, MD, USA; ^3^Maryland Patient Safety Center, Elkridge, MD, USA; ^4^University of Maryland School of Medicine, Baltimore, MD, USA; ^5^Howard University School of Medicine, Washington, DC, USA; ^6^University of Maryland, Baltimore County, Baltimore, MD, USA; ^7^Obstetrics & Gynecology, Holy Cross Health, Silver Spring, MD, USA; ^8^University of Arkansas for Medical Sciences, Little Rock, AR, USA

###### **Correspondence:** Jennifer Callaghan-Koru (jck@umbc.edu)

**Background:** To promote the adoption of evidence-based practices for improved maternal health outcomes, the Alliance for Innovation in Maternal Health has developed patient safety bundles in several areas of obstetric care. Thirty-one hospitals in Maryland are participating in a quality improvement collaborative to implement the “Safe Reduction of Primary Cesarean Births” bundle. We conducted an evaluation of the first year of implementation of the bundle and assessed whether hospital characteristics and implementation strategies are associated with bundle implementation during the collaborative.

**Methods:** The collaborative leaders at each participating hospital completed an online survey of implementation 12 months after the start of the collaborative. Leaders reported the status of implementation of 25 practices included in the bundle as well as hospital characteristics and their use of 15 additional implementation strategies not specifically recommended in the bundle. Descriptive statistics of bundle implementation were calculated and Wilcoxon rank-sum tests were employed to assess whether hospital characteristics and implementation strategies were associated with bundle implementation.

**Findings:** Twenty-five of 31 hospitals (80.1%) provided complete survey responses and two provided partial responses. The average number of the practices that reporting hospitals had fully implemented before the collaborative was 8.6 (SD: 5.5; range: 0 to 17) and during the collaborative was 2.4 (SD: 2.3; range: 0 to 7). Twenty-four of 25 hospitals reported using at least 1 of 15 implementation strategies we assessed. Three of the 15 supplemental strategies were associated with implementing a significantly greater number of practices during the collaborative. Hospitals that developed a formal implementation blueprint, hospitals who identified and prepared champions, and hospitals who conducted consensus discussions, implemented 3.2, 2.0, and 1.9 more practices, respectively, during the collaborative’s first year compared with hospitals that did not use those strategies (all p<0.05).

**Implications for D&I Research:** While three implementation strategies were associated with stronger implementation, these associations may be a result of unmeasured factors such as hospitals’ implementation capacity or culture. More research is needed to understand these relationships and uncover how hospital staff learn of and select among available implementation strategies for this and other patient safety bundles.

### S63 Results from a randomized trial comparing strategies for helping community health centers implement guideline-concordant cardioprotective care

#### Rachel Gold^1,2^, Arwen Bunce^1^, Stuart Cowburn^2^, James V. Davis^1^, Joan Nelson^3^, Christine Nelson^2^, Elisabeth Hicks^4^, Deborah Cohen^4^, James Dearing^5^, Michael Horberg^6^

##### ^1^Kaiser Permanente Center for Health Research, Portland, OR, USA; ^2^Practice Based Research Network, OCHIN, Inc., Portland, OR, USA; ^3^OCHIN, Inc., Portland, OR, USA; ^4^Oregon Health & Science University, Portland, OR, USA; ^5^Michigan State University, East Lansing, MI, USA; ^6^Mid-Atlantic Permanente Medical Group, Kaiser Permanente, Rockville, MD, USA

###### **Correspondence:** Rachel Gold (rachel.gold@kpchr.org)

**Background:** Statins can reduce cardiovascular event risk in patients with diabetes (DM), but statin prescribing lags behind care guidelines. We compared how effectively several implementation strategies supported community health centers’ (CHCs) adoption of a suite of electronic health record (EHR) clinical decision support tools that targeted guideline-concordant statin prescribing in DM. The EHR tools (the ‘CVD Bundle’) were adapted from a previously successful intervention.

**Methods:** A mixed-methods, pragmatic trial; 29 CHCs cluster-randomized to receive: 1) an Implementation Toolkit (instructions on using the CVD Bundle, and on QI practice change techniques); 2) Toolkit + 2-day in-person training of clinic ‘point people’ on the CVD Bundle, with follow-up training webinars; or, 3) Toolkit, training, webinars, + multiple on-site practice facilitation visits. Study CHCs were followed for three years post-implementation support (July 2015-June 2018). We compared statin prescribing (concordant with 2013 AHA/ADA guidelines) in the 12 months pre-intervention versus 36 months post-intervention, and gathered qualitative process data via on-site observations and interviews, and regular calls with clinic point people.

**Findings:** In preliminary analyses, all study clinics’ guideline-concordant statin prescribing improved somewhat in the pre- through post-periods; we found no significant differences between study arms, but some difference between clinics. Qualitative data suggests possible reasons for these results. Variation in staff EHR skills (especially in effective use of data reports) and leadership engagement impacted adoption. Clinics’ practice change culture did not always support adapting workflows to review care recommendation ‘alerts.’ Issues with the EHR tools diminished their utility: there were initial technical difficulties, and a CVD risk calculator was not initially included. The implementation strategies also faced challenges: the Toolkit was rarely used, attendance at webinars was poor, staff turnover diminished training impacts, and few Arm 3 clinics were prepared to ‘use’ facilitation. Level of implementation support appeared less impactful than individual clinics’ readiness to make the targeted changes.

**Implications for D&I Research:** Commonly-used implementation strategies failed to support CHCs’ adoption of EHR tools targeting guideline-concordant cardioprotective prescribing. Guideline dissemination efforts should start with formative evaluation of intended adopters’ needs / preferences so that subsequently deployed implementation strategies are met with receptivity.

### S64 Dissemination and implementation strategies used in pragmatic clinical trials embedded in learning health systems

#### Leah Tuzzio (Leah.Tuzzio@kp.org)

##### Kaiser Permanente Washington Health Research Institute, Kaiser Permanente Washington, Seattle, WA, USA

**Background:** The NIH Health Care Systems Research Collaboratory’s pragmatic clinical trials (PCTs) take place in diverse real-world health care settings. A valuable environment for PCTs is learning health systems (LHSs) where clinical practice influences research and vice versa. LHSs operationalize evidence generated by research, particularly PCTs, into improvements that are sustained after a trial ends. Few investigations have documented the dissemination and implementation approaches used in PCTs to plan for and implement evidence-based advancements to sustain practice change within LHSs.

**Methods:** We interviewed the principal investigators of nine NIH Collaboratory PCTs. We also engaged them and their health system partners in a workshop at the NIH in 2017 about dissemination, implementation and sustainability of evidence-based practices into care. We asked them to describe strategies they used in the design and roll-out of their trials to facilitate future dissemination, implementation and sustainability. Themes emerging from the interviews were summarized into a set of guidance principles for future pragmatic trials and integrated into the NIH Workshop.

**Findings:** The PCTs included implementation aims and methods to help the researchers and health system partners understand how the interventions could be put into place, how they might affect the outcomes and disseminate and implement the findings. They identified six essential strategies: 1) pilot testing, 2) creating a shared purpose, 3) building relationships with stakeholders, 4) developing sustainable resources and infrastructure, 5) evaluating barriers, facilitators, adjustments and acceptability of the intervention, and 6) using national policy levers of effectiveness trial results.

**Implications for D&I Research:** Partnerships between researchers and health systems are a critical element of PCTs so that interventions are designed to fit the needs of and contribute to providing quality care within LHSs. Consistency of the intervention from design to implementation is ideal but challenging since contexts and interventions interact and will inevitably change, this dynamism is at the heart of the LHS. These findings can help research teams build adaptive designs and include questions, decisions and outcomes that are important and relevant to the LHS and improve the system’s abilities to provide effective research that improves health outcomes at the population level.

### S65 VA quality enhancement research initiative: Enhancing learning health systems through implementation of provider and leadership priorities

#### Amy Kilbourne (Amy.Kilbourne@va.gov)

##### VA Center for Clinical Management Research, Veterans Health Administration, Ann Arbor, MI, USA

**Background:** The goal of VA’s Quality Enhancement Research Initiative (QUERI) is to accelerate implementation of effective practices into routine care. QUERI has achieved this goal primarily by identifying effective practices and supporting their implementation through individual projects. However, initiatives that align leadership priorities (“top-down”) with local provider buy-in (“bottom-up”) are essential to achieving the Learning Health System goal of continuous improvement and sustainability over time. We describe two QUERI bottom-up initiatives to promote the sustainability of effective practices in routine care: selection of top clinical priorities by local leadership and the rigorous evaluation of implementation strategies to support provider in the adoption of effective practices that address these priorities.

**Methods:** QUERI solicited nominations for clinical priorities via VA network director interviews (N=12). Facility-level VA providers (N=60) then completed a web-based survey to rank their top clinical priorities. A live voting process was used at a VA regional leader meeting to select 1-3 priorities for QUERI to support further implementation studies. QUERI center leaders (N=15) were also surveyed to identify implementation strategies (defined as highly-specified, theory-based methods to improve uptake of effective practices) used to scale up and spread effective practices related to these priorities.

**Findings:** The top three clinical priorities identified by VA providers were suicide prevention, opioid use disorder, and community care coordination. QUERI centers implemented over 50 effective practices in 2017 related to these priorities. Key implementation strategies tested by QUERI centers via rigorous designs included Replicating Effective Programs (REP), Audit and Feedback, Facilitation, and Evidence-based Quality Improvement (EBQI). Involvement of VA leadership in nomination and selection of clinical priorities that QUERI led to the launching of several randomized evaluations of implementation strategies demonstrating their effectiveness compared to usual dissemination practice (e.g., training alone). VA performance plans subsequently adopted the requirement that local facility leaders implement best practices identified through QUERI.

**Implications for D&I Research:** QUERI’s integration of top-down and bottom-up strategies to identify, implement, and rigorously evaluate effective practices addressing top clinical priorities enhances VA’s evolution towards a Learning Health System, notably by ensuring that local providers, implementation experts, and national leaders have ownership in the process.

### S66 A learning health system’s core set of implementation strategies and change package to scale and evaluate a pragmatic intervention in primary care

#### Katie Coleman (Katie.F.Coleman@kp.org)

##### Kaiser Permanente Washington Health Research Institute, Seattle, WA, USA

**Background:** In 2017, Kaiser Permanente Washington (KPWA) launched a Learning Health System (LHS) program to leverage research capabilities including data analytics, implementation design and support, scientific consultation and evaluation to support patient experience and health. The Community Resource Specialist (CRS) initiative is an example of what is possible when care delivery and research partner in structured ways. In 2013, researchers at KPWA received PCORI funds to co-design, implement, and evaluate a staff team role that would enhance primary care’s ability to identify and link patients with community resources. The role was called a Community Resource Specialist (CRS) and was pilot tested in three primary care sites. After 18 months of implementation, more than 600 patients had been referred to a community resource. Patients reported high levels of satisfaction, and 80% reported making progress toward their goals. The LHS identified the CRS as a high-potential program for spread. In 2017, leadership decided to embed the CRS role in its 25 owned and operated medical centers.

**Methods:** The LHS team articulated a core set of key implementation strategies for use across medical centers (e.g., identify clinical champions, audit and feedback, behavioral rehearsal fidelity assessment and feedback) and created a change package that utilized practice facilitation to build relationships with primary care teams, characterize the unique contextual factors of influence, and identify and tailor strategies to target determinants. A type 2 mixed methods evaluation is being conducted and includes a retrospective cohort design for examining effectiveness and a multiple case study approach to studying implementation.

**Findings:** Nineteen full time CRSs have been embedded in primary care practice with a total of 2942 unique adult patients seen. Each CRS is using a standard form to identify social needs. LHS supported implementation will begin August 2018.

**Implications for D&I Research:** Better designed, more relevant research is not enough to precipitate system change. For health systems to integrate changes to care delivery, attention must be paid to what happens after the research is done – how findings align with organizational goals, how science can support the implementation of promising work, and how rapid assessment can offer opportunities for improvement.

### S67 Veterans health administration (VHA) diffusion of excellence initiative: Motivation and context for development of innovative practices

#### George Jackson^1,2^, Brandolyn White^2^, Caitlin Reardon^3^, Andrea Nevedal^4^, Ryan Vega^5^, Heather King^1,2^, Jennifer Lindquist^2^, Sarah Cutrona^6,7^, Thomas Houston^6,7^, Allen Gifford^7,8^, Kathryn DeLaughter^6,7^, Elizabeth Orvek^6,7^, Lindsay White^7^, Megan Shaheen^9^, Thalia Sirjue^9^, Laura Damschroder^10^

##### ^1^Duke University, Durham, NC, USA; ^2^Durham VA Health Care System, Veterans Health Administration, Durham, NC, USA; ^3^VA Ann Arbor Healthcare System, Veterans Health Administration, Ann Arbor, MI, USA; ^4^VA Palo Alto Health Care System, Menlo Park, CA, USA; ^5^Diffusion of Excellence, Veterans Health Administration, Washington, DC, USA; ^6^University of Massachusetts Medical School, Worcester, MA, USA; ^7^Bedford VA Medical Center, Veterans Health Administration Bedford, MA, USA; ^8^Boston University, Boston, MA, USA; ^9^Atlas Research, Washington, DC, USA; ^10^VA Ann Arbor Center for Clinical Management Research, Ann Arbor, MI, USA

###### **Correspondence:** George Jackson (george.l.jackson@duke.edu)

**Background:** The Veterans Health Administration (VHA), the largest integrated delivery system in the US, strives to empower frontline staff to develop innovations and practices to improve outcomes for Veterans and staff. The VHA Diffusion of Excellence Initiative (DEI) works to identify these practices by asking staff from across the VHA to submit successful practices to a “Shark Tank” competition, in which VHA facility directors bid on the opportunity to implement one of these practices with external facilitation support. In the first three rounds, 1,054 promising practices were submitted and 36 were designated as Gold Status Practices. We sought to examine the motivations for developing and seeking to spread practices/innovations.

**Methods:** We conducted semi-structured telephone interviews with 22 of 23 Gold Status Facility Fellows (staff that developed practices) for Gold Status Practices identified through the two most recent Shark Tanks. Data were collected and analyzed using the Consolidated Framework for Implementation Research and Weiner Theory of Organizational Readiness for Change.

**Findings:** Gold Status Facility Fellows were typically intrinsically motivated (Personal Readiness) to develop practices when they identified a staff and/or patient need though personal observations (Tension for Change). While there were reports of corresponding performance measures and supporting research (External Policies and Incentives), most felt a personal and professional duty to help improve VHA systems and processes to provide the best care and experiences possible to Veterans (Patient Needs & Resources). They collaboratively developed best practices, which resulted in strong support from staff (Engaging: Key Stakeholders). In addition, facility leaders provided resources to make implementation possible (Available Resources) and created an environment in which staff felt safe to try new things because e.g., they were encouraged by their clinical leaders to innovate (Learning Climate). Successful implementation in Gold Status Facilities and a desire to enhance processes across the system, with encouragement from leaders, led them to share the practice in the Shark Tank.

**Implications for D&I Research:** Intrinsic motivation of frontline staff for developing and implementing new innovations/practices, coupled with supportive contextual factors, all contributed to achieving Gold Status Practice designation. These findings point to opportunities to leverage individual intrinsic motivation to develop and spread innovations.

### S68 Arming the frontline workforce to support a culture of implementing improvement: Results from a virtual training program

#### Laura Damschroder, Claire H. Robinson, Michelle M. Barbaresso, Nicholas Yankey, Michael Palmer, Julie C. Lowery

##### VA Ann Arbor Center for Clinical Management Research and PROVE QUERI, Veterans Health Administration, Ann Arbor, MI, USA

###### **Correspondence:** Laura Damschroder (laura.damschroder@va.gov)

**Background:** Integrating quality improvement (QI) into everyday practice is a building-block skill for organizations to transform into true learning health systems receptive to evidence-based innovations. However, learning and applying quality improvement skills is challenging for providers working in busy clinical environments and within the realities of conflicting priorities. The Learn. Engage. Act. Process. (LEAP) Program is a frontline team-level intervention, designed to empower staff to implement and enhance evidence-based programs by building QI skills within the context of significant constraints on time and resources. The aim of this study was to evaluate the ability of LEAP to increase QI skills among teams leading weight management programs in Veterans Affairs (VA).

**Methods:** LEAP is a 21-week structured program designed to coach frontline staff in applying foundational QI methods through weekly hands-on participation in a team-developed QI project. The curriculum is delivered via an online platform in addition to group and individual QI coaching via video conferencing calls. The LEAP curriculum is adapted from a course developed by the Institute for Healthcare Improvement and Harvard. QI skill level was self-assessed before and after participation in LEAP. Semi-structured interviews were conducted with team leaders six months after graduation from LEAP.

**Findings:** 17 teams across 4 cohorts, comprising 100 providers and staff participated in LEAP. Team members reported significant improvements in all six categories of QI skills (e.g., ability to construct and interpret a run chart) after participation in LEAP compared to baseline (p’s<0.01). Lack of time was the biggest challenge for all participating teams amid pressures of fulfilling other clinical and administrative responsibilities. Nonetheless, all team leaders planned to continue in the maintenance program (LEAP*On*) to support their ongoing QI efforts.

**Implications for D&I Research:** LEAP successfully increased QI skills among frontline participants though they struggled with significant time constraints. LEAP is a scalable program that increases capability of frontline workforce to engage in QI, creating a receptive foundation for implementing evidence-based practices. But the challenges of implementing change within a healthcare delivery system highlight the clash that occurs when the theory of implementation research intersects with the pragmatics of QI, including the realities of overburdened clinical settings.

### S69 De-implementing long-term opioids for chronic pain: Results of a clinical trial

#### Michael Parchman^1^, Laura-Mae Baldwin^2^, Robert Penfold^1^, Brooke Ike^3^, David Tauben^3^, Kari Stephens^3^, Mark Stephens^4^

##### ^1^Kaiser Permanente Washington Health Research Institute, Seattle, WA, USA; ^2^University of Washington, Institute of Translational Health Sciences, Seattle, WA, USA; ^3^University of Washington, Seattle, WA, USA; ^4^Change Management Consulting, Seattle, WA, USA

###### **Correspondence:** Michael Parchman (Michael.X.Parchman@kp.org)

**Background:** Little is known about the use of defined implementation strategies to de-implement an existing practice such as long-term opioid therapy (LtOT) for chronic pain. We previously described “Six Building Blocks” for clinic re-design to address opioid medication management based on visits to exemplar primary care clinics. Here we report on the results of a trial using multiple strategies to implement the Six Building Blocks program to address overuse of LtOT for patients with chronic non-cancer pain.

**Methods:** Six rural primary care organizations with 20 clinic locations across eastern Washington and central Idaho were enrolled. Ten Expert Recommendations for Implementing Change (ERIC) strategies were employed over 15 months: an implementation blueprint (the Six Building Blocks program), assess for readiness and identify barriers, conduct local consensus discussions, practice facilitation, capture/share local knowledge, a learning collaborative, development of tools for quality monitoring, identify/support clinical champions, promote adaptability, and obtain formal commitments. Opioid prescribing data were extracted from the electronic health records of each clinic. Outcomes were monthly trends in: 1) the proportion of patients on high-dose opioids (≥ 100 MED), and 2) the total number of patients on LtOT. Analyses consisted of interrupted time series (ITS) both within study sites and a difference-in-difference using a non-equivalent control group of members of a large health plan who resided in the same Primary Care Service Area

**Findings:** The proportion of patients on high-dose LtOT declined from 11.8% to 9.6% across the study clinics. The within study site ITS analysis approached significance: p=0.0596. This rate of decline was faster in study clinics than the non-equivalent control group: (p=0.018). The rate of decline in the total count of patients receiving an opioid prescription each month, from 2,065 to 1,776, was significant both within the study sites (p<0.001) and when compared to the non-equivalent control group (p<0.001).

**Implications for D&I Research:** A multi-component de-implementation support intervention for LtOT in chronic pain patients was effective in both reducing the dose of opioids prescribed and the total number of patients using LtOT. De-implementation will most likely require the use of multiple strategies simultaneously within primary care teams.

### S70 Developing a rubric for matching implementation strategies to barriers: Cross-walking implementation theory and practice

#### Craig Rosen^1^, David Riggs^2^, Alan Peterson^3^, Stacey Young-McCaughan^3^, Elisa Borah^4^, William Brim^2^, Kate Comtois^5^, Jeffrey Cook^2^, Carrie Adrian Davis^6^, Katherine Dondanville^3^, Erin Finley^7^, Lisa French^2^, Melissa Mistretta^2^, Andrea Neitzer^6^, Shannon Wiltsey-Stirman^1^, Carmen McLean^6,8^

##### ^1^VA Palo Alto Health Care System, National Center for PTSD Dissemination & Training Division, Veterans Health Administration, Menlo Park, CA, USA; ^2^Center for Deployment Psychology, Bethesda, MD, USA; ^3^University of Texas Health Sciences Center at San Antonio, San Antonio, TX, USA; ^4^University of Texas Austin, Austin, TX, USA; ^5^University of Washington, Seattle, WA, USA; ^6^National Center for PTSD, Menlo Park, CA, USA; ^7^South Texas Veteran Health Care System, Veterans Health Administration, San Antonio, TX, USA; ^8^Stanford University, School of Medicine, Palo Alto, CA, USA

###### **Correspondence:** Craig Rosen (craig.rosen@va.gov)

**Background:** Producing system change often requires tailoring implementation to local context. Methods have been explored for mapping strategies to barriers, however frameworks for profiling relevant barriers and facilitators, such as the Consolidated Framework for Implementation Research (CFIR), are not linked to taxonomies of change strategies, such as Expert Recommendations for Implementing Change (ERIC). Change strategies are therefore often selected based on the facilitators’ or local team’s judgment. We developed a toolkit-guided facilitation approach (Targeted Assessment and Context-Tailored Implementation of Change Strategies, TACTICS), which includes a rubric for selecting implementation strategies based on local needs assessment. We here describe our process for developing that rubric, including a comparison of field- and theory- based barriers and strategies.

**Methods:** A mapping and consensus approach was used to link barriers observed in the field with corresponding categories in the CFIR framework. Barriers were derived from a previously developed summary of common barriers to implementing evidence-based psychotherapies (EBPs) in military mental health settings.^1^ Initial maps were developed by one staff member, reviewed iteratively by multiple study team members, then discussed to reach consensus on the final rubric. A similar approach was used to map field-based implementation strategies from the same summary with the ERIC taxonomy.

**Findings:** Cross-walking the field manual, CFIR, and ERIC illuminated areas of convergence and divergence. Some field-identified barriers and strategies were not represented in the CFIR and ERIC frameworks. CFIR highlighted important contextual factors not considered in the field-based manual. ERIC also provided additional solutions for barriers not fully addressed in the manual.

**Implications for D&I Research:** Mapping change strategies (ERIC) to specific barriers (CFIR) provides a logical model for assessing whether particular strategies have their intended impact on barriers they were meant to address. They also highlight the value of using insights from both theory and practice to identify appropriate strategies. TACTICS will be tested in a stepped-wedge EBP-implementation trial. If successful, such rubrics may facilitate more systematic matching of implementation strategies to local conditions.

1. Center for Deployment Psychology. Lessons Learned Manual. https://deploymentpsych.org/system/files/member_resource/FINAL_Lessons_Learned_Manual_Aug16.pdf. Published 2015. Accessed July 24, 2018.

### S71 Implementing improved cardiovascular preventive care in primary care practices

#### Deborah Cohen^1^, Shannon Sweeney^2^, William Miller^3^, Jennifer Hall^1^, Tanisha Woodson^1^, Bijal Balasubramanian^4^, Miguel Marino^1^, Rachel Springer^1^, Benjamin Crabtree^2^, Leif Solberg^5^

##### ^1^Oregon Health & Science University, Portland, OR, USA; ^2^Rutgers Robert Wood Johnson Medical School, New Brunswick, NJ, USA; ^3^Lehigh Valley Hospital, Allentown, PA, USA; ^4^University of Texas School of Public Health, Dallas, TX, USA; ^5^Institute for Education and Research, HealthPartners, Bloomington, MN, USA

###### **Correspondence:** Deborah Cohen (cohendj@ohsu.edu)

**Background:** Primary care is the ideal setting for evidence-based cardiovascular disease (CVD) prevention, but delivery rates aren’t optimal and implementation of systematic approaches requires understanding what improves care delivery.

**Methods:** 1500 smaller (<10 clinician) primary care practices located across seven different US regions (Cooperatives) participating in EvidenceNOW, a national test of improvement interventions. From 2016-2018, Cooperatives delivered support to improve appropriate aspirin prescribing (A), blood pressure control (BP), and smoking cessation counseling (S). We collected CMS A, BP, S measures from practices quarterly. Cluster analyses identified practice subgroups with similar longitudinal trajectories in CMS metrics. These data were used to purposively select practices with a positive change in A, BP and/or S. Practices selected also varied by location, size, and ownership to increase representativeness. We conducted interviews with practice coaches and/or a practice member. We coded reported practice changes to improve CVD prevention and used Qualitative Comparative Analysis (QCA) procedures to analyze CMS and transposed qualitative data.

**Findings:** Of the 1,047 practices that submitted A, BP, S data at ≥4 timepoints, cluster analyses revealed a large proportion of practices (83% for A, 53% for BP, 54% for S) did not change significantly or performance decreased over time. For BP, 22% of practices demonstrated steady, modest improvement (change over time <10%); only 3% showed large and rapid improvement. 8% and 20% of practices showed large, dramatic improvements (>30% change in performance over two years) in A and S measures respectively; notably, none demonstrated steady improvement over time. QCA findings showed that documentation change was one approach for improving A and S measures, but not for improving BP, possibly explaining the large, dramatic A and S changes observed. Improving BP involved extensive practice change, including steps to assure accuracy of readings and to re-engage patients with high readings, which may explain the steady, small increases observed in BP.

**Implications for D&I Research:** Documentation changes may improve CMS measures for A and S, but more extensive approaches for practice change may be needed to improve BP outcomes. Understanding variations in approaches for cardiovascular prevention improvement is important for dissemination and implementation of this essential preventive care.

### S72 Spreading VA’s emergency department -rapid access clinics (ED-RAC) intervention: Key factors for success

#### Lauren Penney^1,2^, Isomi Miake-Lye^3^, Davis Lewis^4^, Adiran D'Amico^4^, Kelli Lee^4^, Brianna Scott^4^, Susan Kirsh^5^, Kristina Cordasco^6^

##### ^1^South Texas Veterans Health Care System, Veterans Health Administration, San Antonio, TX, USA; ^2^UT Health San Antonio, San Antonio, TX, USA; ^3^Veterans Health Administration, Greater Los Angeles Healthcare System, Los Angeles, CA, USA; ^4^Pittsburgh VAMC, Veterans Health Administration, Pittsburgh, PA, USA; ^5^Office of Access, Veterans Health Administration, Washington, DC, USA; ^6^US Department of Veterans Affairs Greater Los Angeles Healthcare System, Veterans Health Administration, Los Angeles, CA, USA

###### **Correspondence:** Lauren Penney (Lauren.Penney@va.gov)

**Background:** The Veterans Administration’s (VA) Emergency Department (ED) Rapid Access Clinic (ED-RAC) intervention, which facilitates Veterans’ access to urgent specialty care follow-up after ED visits, was developed and implemented in one VA site. We conducted a formative evaluation of an initiative aimed at spreading ED-RAC to 24 additional VA sites.

**Methods:** We assessed implementation progress, barriers and facilitators in spread sites, August 2017-June 2018. We used serial questionnaires of spread-site champions, administered every 6-8 weeks; semi-structured interviews with 12 (purposively-selected) spread-site champions (fast, slow and non-adopters); and fieldnotes of group and one-on-one coaching meetings between spread-site champions and the spread support team. Data was analyzed using matrix and thematic analyses.

**Findings:** Eleven months following launch of the spread initiative, four sites (17%) had implemented ED-RAC in two or more specialties, seven (29%) in one specialty, and 13 (54%) had not yet implemented. Factors that were helping or challenging to local implementation success were the spread-site champion’s: (1) ability to gain buy-in from, and coordinate efforts across local specialty, administrative, and informatics leaders; (2) having time to devote to implementation; and (3) ability to overcome implementation challenges. The importance of relationships was an additional cross-cutting theme. Approaches and mechanisms for obtaining stakeholder buy-in, and coordinating efforts, varied by locality-specific organizational configurations. Champions were more effective when processes similar to ED-RAC were already in place locally, and/or ED-RAC aligned with current local priorities. In addition, champions with pre-existing relationships spanning services could leverage them for obtaining buy-in and problem-solving support. Champions lacking these factors encountered bureaucratic and buy-in hurdles that delayed or stalled progress. These effects were compounded when champions lacked sufficient time to devote to implementation efforts.

**Implications for D&I Research:** Local context and champion-specific factors are key to the success of initiatives spreading innovations in VA. Providing targeted, individualized coaching from a spread team was helpful in overcoming some barriers. However, further study is needed to determine which coaching strategies would be most effective in assisting champions in navigating local bureaucracy and relationships to facilitate implementation success.

### S73 Understanding determinants of acute stroke thrombolysis using the tailored implementation for chronic diseases framework: A qualitative study

#### Lesli Skolarus^1^, Gina Neshewat^1^, Lacey Evans^1^, Molly Green^1^, Narmeen Rehman^1^, Zach Landis-Lewis^1^, Jillian Welsh-Schrader^2^, Anne Sales^1^

##### ^1^University of Michigan, Ann Arbor, MI, USA; ^2^Hurley Medical Center, Flint, MI, USA

###### **Correspondence:** Lesli Skolarus (lerusche@umich.edu)

**Background:** The Tailored Implementation in Chronic Disease (TICD) framework is a comprehensive framework describing the determinants of implementation success that has been used extensively in primary care settings. We explored the utility of the TICD to identify determinants of practice in an acute care setting, namely guideline-concordant acute stroke thrombolysis in a large, low-resourced, predominately minority serving Emergency Department (ED).

**Methods:** Through brainstorming and expert review, we developed an interview guide informed by the TICD framework. We then conducted semi-structured interviews with data collected through written transcripts, audio transcripts or interviewer notes based on participant availability. Three independent coders then performed a content analysis using template analysis, but open to new determinants that arose from the data, into the TICD framework.

**Findings:** We performed a total of 15 semi-structured interviews with ED acute stroke providers including medical technicians, nurses, and physicians. Participants with less schedule autonomy (direct care nurses and technicians) had shorter interviews and we used interview notes to collect data. We identified Guideline Factors, Individual Health Professional Factors, and Patient Factors as negative determinants or barriers to following guidelines for thrombolysis treatment. The domain Professional Interactions was a positive determinant or facilitator for treatment. We identified three determinants, Health Care Professional Burnout, Staff Turnover and Surrogate Decision Making, that are not currently part of the TICD framework.

**Implications for D&I Research:** Most determinants of use of acute stroke thrombolysis are included within the TICD framework. Inclusion of Health Care Professional Burnout, Staff Turnover and Surrogate Decision Making may assist in expanding the TICD to the acute medical setting.

### S74 Dissemination of CAPABLE model of care in a Medicaid waiver home and community based waiver program to improve function

#### Dr. Sandra Spoelstra^1,2,3^, Monica Schueller^1^, Laura Gitlin^4^, Sarah Szanton^2^

##### ^1^Grand Valley State University, Grand Rapids, MI, USA; ^2^John Hopkins University, Baltimore, MD, USA; ^3^University of Toronto, Toronto, MI, USA; ^4^Drexel University, Philadelphia, PA, USA

###### **Correspondence:** Sandra Spoelstra (spoelsts@gvsu.edu)

**Background:** There are 39 million Americans over age 65; and 42% of older adults report problems with function, which can lead to difficulty with activities of daily living (ADLs), falls, and nursing home (NH) placement. Implementing evidence-based models like CAPABLE, focused on aging-in-place, is a public health priority. Prior work adapted CAPABLE for the Medicaid Home and Community-Based waiver in Michigan.

**Methods:** Kotter’s 8-Step Change Model guided this 3-year participatory mixed method 3-group design trial. We examined site-level outcomes of rate of use of CAPABLE components after deploying implementation strategies of education, coalition building, audit and feedback, and external facilitation. We also examined beneficiary-level outcomes (ADL/IADLs, falls, hospitalizations). We built multiple coalitions: waiver directors, waiver supervisors, by-discipline groups (RN, OT, SW), and policymakers/quality reviewers. Clinicians (N=34) were trained and CAPABLE was provided to beneficiaries (N=270) at 4-waiver sites then compared to a usual care matched (age, gender, race) cohort (N=1,350). Data were analyzed using descriptive statistics, t-test, chi-square, ANOVA, and logistic regression.

**Findings:** Clinician preferred learning method varied (92.3% visual, 84.6% hands-on/interactive, 69.2% audio, 38.5% reading, 23.1% role-play). Scaling-up education mode evolved from face-to-face/reading to virtual/visual/audio with reduced reading and role-play, while facilitation transitioned from external to internal with champions who built a coalition. All sites were aware of audit and feedback results, which created competition and increased adoption. OTs provided a mean of 4.16 (SD 1.98) visits with RNs providing 3.60 (SD 1.23), and social workers 0.21 (SD 0.75). There were 2.46 (SD 1.89) inter-disciplinary coordination occurrences conducted per beneficiary. Medicaid beneficiaries improved pre-/post-CAPABLE ADLs (8.51 ± 3.08 to 7.80 ± 2.86; p=0.01), IADLs (6.43 ± 1.31 to 5.62 ± 1.09; p<.001), falls (34.8% to 20.8%; p=.001), and hospitalizations (0.43 ± 1.51 to 0.23 ± 0.60; p=0.03), confirming CAPABLE effectiveness for the Medicaid program.

**Implications for D&I Research:** This study demonstrated the importance of conducting trials to examine implementation strategies in a practice environment prior to scaling-up. Sustainability may be more likely to occur if implementation strategies are examined within the setting prior to scaling-up, improving adoption.

### S75 Does adapting hospital at home to facilitate implementation reduce its effectiveness?

#### Albert Siu^1,2^, Robert Zimbroff^2^, Alex Federman^2^, Linda DeCherrie^2^, Melissa Garrido^3^, Barbara Morano^2^, Sara Lubetsky^2^, Elisse Catalan^2^, Bruce Leff^4^

##### ^1^James J. Peters VA Medical Center, Veterans Health Administration, New York, NY, USA; ^2^Icahn School of Medicine at Mount Sinai, New York, NY, USA; ^3^James J. Peters VA Medical Center, Veterans Health Administration, Bronx, NY, USA; ^4^Johns Hopkins University School of Medicine, Baltimore, MD, USA

###### **Correspondence:** Albert Siu (albert.siu@mssm.edu)

**Background:** Hospital at Home (HaH) has been shown to be safe and effective in studies, but its dissemination necessarily involves significant adaptation to local circumstances. In 2014, Mount Sinai implemented HaH for selected patients with diagnoses (e.g., pneumonia) that would otherwise be admitted. The program retained core components of HaH--delivering hospital-level services at home, daily RN and clinician home visits, and 24/7 on-call availability. Over 33 months, a series of program adaptations were implemented, including addition of 30-day follow up, expanded services, altered staffing arrangements, and incorporating telehealth. We tested whether program benefits diminished over time either due to “program drift” (i.e., deviations from research conditions) and/or “voltage drop” (i.e., implementation in heterogeneous settings).

**Methods:** Patients were eligible for HaH if they were ≥18 years, had eligible coverage, and required inpatient admission. Patients were excluded if they were clinically unstable, required cardiac monitoring or intensive care, lived in an unsafe environment, or resided outside Manhattan. Outcomes assessed were length of stay (LOS), 30-day readmissions and emergency department (ED) visits, escalations (i.e., needing to transfer the patient to the hospital), and patient ratings of care. We used logistic or linear regression to model the average marginal effect for the numerical quarter of enrollment after an initial 6-month pilot phase. The models controlled for season, patient demographics, insurance, physical function, general health, and diagnosis.

**Findings:** 295 subjects received HaH. The average LOS was 3.2 days. 12.2% had escalations, 8.6% had readmissions, 5.8% had ED revisits, and 68.8% provided the highest rating for overall hospital care. Controlling for patient characteristics, there was no change over nine quarters in LOS (-0.005 days; p=0.81), escalations (OR 1.07, p=0.08), 30-day ED visits (OR 1.05, p=0.08), 30 day readmission (OR 0.98, p=0.30), and rating for overall hospital care (OR 0.98, p=0.32).

**Implications for D&I Research:** The implementation of evidence-based programs necessarily requires adaptation. We made serial adaptations to HaH. Despite this, outcomes were similar to those reported in previous studies and we did not observe changes over time in effectiveness across several measures. Adaptations to evidence-based programs can be made without necessarily causing diminished benefits due to ‘program drift’ or ‘voltage drop.’

### S76 Scaling up medication treatment for opioid use disorder in 25 county health safety net clinics

#### Mehret Assefa (mehret.assefa@stanford.edu)

##### Stanford University, Palo Alto, CA, USA

**Background:** Federally-qualified health centers (FQHCs) provide a unique opportunity to reach persons with opioid use disorder (OUD) seeking primary care health care. However, many FQHCs face challenges and barriers to providing treatment for OUD patients. Therefore, implementation strategies to: expand the capacity of safety net clinics to reach this vulnerable population; increase the number of patients prescribed addiction medication (buprenorphine); increase the number of addiction medication prescribers (x-waivered); and improve patient outcomes is imperative.

**Methods:** 25 safety net clinics FQHCs across California participated in an implementation support initiative. Data were obtained from: 1) retrospective quantitative and qualitative reports, and 2) prospective key informant interviews. Multicomponent implementation strategies included: learning collaboratives, expert external coaching, and interactive trainings. Primary implementation outcomes assessed over a two-year period included: 1) number of patients on addiction medications – reach; and 2) number of x-waivered prescribers – adoption. Potential mediators and moderators of implementation outcomes were identified using the Consolidated Framework for Implementation Research (CFIR) Index.

**Findings:** We report on: 1) changes in the number of patients on addiction medication and the number of x-waivered prescribers over time; 2) the degree of participation by clinics in implementation strategies by activity type; and 3) organizational and patient level barriers, i.e. mediators and moderators, by frequency over time.

**Implications for D&I Research:** These data provide insight into barriers to scaling up addiction medications for OUD in primary care settings. Strategies tailored to specifically address barriers rather than one-size-fits all may result in better implementation outcomes.

### S77 Payer and provider practices that facilitate scale-up of opioid use disorder pharmacotherapy treatment

#### Todd Molfenter (todd.molfenter@wisc.edu)

##### University of Wisconsin - Madison, Madison, WI, USA

**Background:** Payers and specialty use disorder (SUD) treatment organizations are expected to play a key role in the implementation of opioid use disorder (OUD) pharmacotherapy treatment in response to the opioid crisis. Yet, <40% of SUD treatment organizations provide this service, representing a significant implementation gap. This workshop will share data from two implementation research studies that the UW-Madison Center Health Enhancement System Studies, (CHESS) conducted with 100+ SUD treatment sites in three states attempting to implement OUD pharmacotherapies.

**Methods:** A mixed methods approach of descriptive statistics and qualitative analysis using grounded theory was used to identify factors and traits associated with OUD pharmacotherapy implementation. Quantitative data was then collected and logistic regression analysis was used to test for impact and associations.

**Findings:** The findings describe the a) payer processes of influence, b) organizational facility characteristics, c) organizational support processes, and d) organizational change management behaviors that facilitate and inhibit the adoption of OUD pharmacotherapy.

**Implications for D&I Research:** The resulting set of factors and principles will provide insights into a multi-level framework that can be applied to scaling-up OUD pharmacotherapy treatment.

### S78 Implementation of medication-assisted treatment for opioid use disorder in the veterans health administration

#### Amanda Midboe^1^, William Becker^2^, Karen Drexler^3^, Eric Hawkins^4^, Mark McGovern^5^

##### ^1^Ci2i VAPAHCS, Veterans Health Administration, Menlo Park, CA, USA; ^2^VA New Haven, Veterans Health Administration, New Haven, CT, USA; ^3^Office of Mental Health and Suicide Prevention, Veterans Health Administration, Atlanta, GA, USA; ^4^VA CESATE, Veterans Health Administration, Seattle, WA, USA; ^5^Stanford University School of Medicine, Stanford, CA, USA

###### **Correspondence:** Amanda Midboe (amanda.midboe@va.gov)

**Background:** Within the Veterans Health Administration (VHA), there is considerable variation in Veterans engagement in evidence-based treatment for opioid use disorder (OUD). There are local and national implementation efforts underway to enhance access to evidence-based treatment, with a focus on enhancing medication assisted treatment (MAT) for OUD. Local efforts include facility-level work that relies on tailoring implementation strategies to local settings and context. National efforts include a national train-the-trainer conference for MAT for OUD, with follow-up implementation strategies to target low-performing facilities. Both local and national efforts rely on implementation facilitation (IF), an evidence-based implementation strategy, to support low-performing sites. Through the tailoring of IF activities to site-specific needs, it is anticipated that the variation in Veteran engagement in MAT for OUD will decrease.

**Methods:** VHA administrative data is used to identify variation in facility-level performance in MAT for OUD, which allows for implementation efforts to be targeted to low-performing facilities. A national conference will take place in August 2018 to train clinical staff in each of the 21 VHA service networks in delivery of MAT for OUD. Pre-implementation work is underway and the implementation phase includes not only the training conference but a blending of process improvement techniques and other implementation strategies. For sites identified as low-performing following the national training conference, IF will be used. The IF strategy will be modeled after local implementation work that began in April 2018 in two low-performing facilities in the northeast.

**Findings:** Pre-implementation formative evaluation reveals actionable targets for IF, including (1) gaps in non-specialist staff’s knowledge, skills and attitudes related to MAT provision, (2) enhancement of care coordination pathways for movement of patients who are stabilizing or de-stabilizing to appropriate care as needed, and (3) a need to train staff in how to engage patients with OUD who are on long-term opioid therapy in patient-centered opioid reassessment techniques.

**Implications for D&I Research:** In a large national healthcare system, it is important to understand how to address variation in not just performance but implementation needs of sites. This work will provide insight into the value of blending process improvement techniques and IF strategies that target low-performing sites.

## Global Dissemination and Implementation Science

### S79 Student, teacher, and caregiver perspectives on implementing mental health services in Ugandan primary schools

#### Catherine Carlson^1^, Sophie Namy^2^, Olive Musoni^1^, Violet Nkwanzi^1^, Milton Wainberg^3^, Janet Nakuti^2^, Katharina Anton-Erxleben^2^, Dipak Naker^2^

##### ^1^University of Alabama, Tuscaloosa, AL, USA; ^2^Raising Voices, Kampala, Uganda; ^3^New York State Psychiatric Institute, Columbia University, New York, NY, USA

###### **Correspondence:** Catherine Carlson (ccarlson5@ua.edu)

**Background:** Situated throughout urban and rural areas, schools are found in communities with few-to-no other social or health services and provide a critical platform for mental health service delivery in low and middle-income countries. However, a gap exists in the global literature on effective implementation strategies for delivering child and adolescent mental health interventions in schools.^1-2^ The Good School Toolkit (GST) is an evidence-based violence prevention program used throughout schools in Uganda.^4^ The GST prevents violence by working to change school climate, improve relationships between teachers and students, and develop transparent and participatory decision-making.^5^

**Methods:** We conducted a qualitative study to explore students’, teachers’, and caregivers’ perspectives on implementing group-based mental health interventions within the GST, drawing upon the Consolidated Framework for Implementation Research (CFIR)^6^. Data collection occurred in February 2018, in two schools in Kampala, Uganda that have previously implemented the Good School Toolkit. Trained, local researchers facilitated four focus group discussions with caregivers (n=22), four focus group discussions with teachers (n=25), and in-depth interviews with primary school students, class 5-7 (n=12). After transcription and translation, verbatim transcripts were analyzed using framework analysis approach.

**Findings:** Results indicated the potential usefulness of implementing a mental health intervention within an existing program (the GST) that already addresses implementation factors across key domains of the CFIR model (such as school climate and decision making). Other findings reveal the need to involve parents as part of a successful implementation strategy, as well as the need to embed specific mental health literacy and promotion activities in any implementation strategy to overcome mental health stigma and misconceptions. Other findings reveal important considerations for the characteristics of the intervention (i.e. non-stigmatizing language, timing, and delivery of intervention) and characteristics of the intervention facilitators (i.e. specific teachers).

**Implications for D&I Research:** Results from this study will inform the development and pilot of an implementation strategy for delivering mental healthcare in Ugandan schools. Implications for D&I research indicate the potential value of leveraging an existing program already addressing key implementation factors for the implementation of school-based mental health interventions.

### S80 Factors influencing vaccine coverage in Tshwane District, South Africa: An application of the Consolidated Framework for Implementation Research

#### Heather Lynne Fraser^1^, Ntombizodwa Ndlovu^1^, Mashaole Makwela^2^, Rohit Ramaswamy^3^, Latifat Ibisomi^1^

##### ^1^University of the Witwatersrand, Johannesburg, South Africa; ^2^Department of Health, Gauteng Provincial Government, Johannesburg, South Africa; ^3^Gillings School of Public Health, University of North Carolina, Chapel Hill, NC, USA

###### **Correspondence:** Heather Lynne Fraser (307537@students.wits.ac.za)

**Background:** It has been well established that immunisation is essential in preventing illnesses and reducing childhood mortality. In Tshwane District in the Gauteng Province of South Africa, vaccine coverage for the population under one is sub-optimal, with some areas reporting coverage levels as low as 44%, and others reporting coverage levels well over 100%. There has been no systematic exploration of the issues that have led to this sub-optimal coverage. This study explores the factors that influence vaccine coverage, both in terms of the implementation of the vaccine programme and the process of coverage estimation, in Tshwane District, using the Consolidated Framework for Implementation Research (CFIR).

**Methods:** A qualitative approach was applied, through guided in-depth interviews with key informants involved in vaccine programme implementation and coverage estimation, including nurses; managers at facility, sub-district, district and provincial levels; and information system managers. The framework approach, a form of deductive analysis, was used, with the CFIR domains and constructs guiding the development of the interview guides. The transcripts were then coded and analysed, allowing themes to emerge, in order to fully reflect the responses of the participants - an important aspect of the framework approach.

**Findings:** Using the constructs of the CFIR, barriers to the implementation of the vaccine programme were found to be: availability of resources – particularly human resources and equipment such as computers; structural characteristics – particularly hierarchical decision-making and the division of the healthcare system into private and public sectors; and patient needs – particularly in terms of access to clinics. Complexity of the process of population estimation was found to be a barrier to vaccine coverage estimation. Based on the factors identified, recommendations were made to improve the quality of implementation of the vaccine programme, and to improve coverage estimation.

**Implications for D&I Research:** Further research should be done to assess whether these factors and the recommended implementation strategies may apply and or be adapted to other settings, respectively. A quality improvement project, based on the recommendations, would be of value to apply the findings of this study to vaccine coverage in Tshwane District.

### S81 Evaluating applications of the consolidated framework for implementation research (CFIR) in low- and middle-income country settings

#### Arianna Means, Christopher Kemp, Marie Claire Gwayi-Chore, Sarah Gimbel, Kenneth Sherr, Brad Wagenaar, Judith Wasserheit, Bryan Weiner

##### University of Washington, Seattle, WA, USA

###### **Correspondence:** Christopher Kemp (kempc@uw.edu)

**Background:** The Consolidated Framework for Implementation Research (CFIR) is frequently utilized to evaluate the spectrum of factors influencing implementation effectiveness. However, it was not initially designed for low-and-middle income country (LMIC) settings^1^. We conducted a systematic review with three primary objectives: (1) to characterize CFIR applications in LMIC contexts, (2) to identify which CFIR constructs are compatible or incompatible with global implementation science, and (3) to identify specific opportunities to improve CFIR relevancy in LMICs.

**Methods:** We searched Medline, EMBASE, CINAHL, SCOPUS, Web of Science, and PsycINFO until February 2018 to identify articles that mentioned or cited the original CFIR publication^1^ within a LMIC. Data were abstracted using a standardized data abstraction tool. We also provided an online questionnaire to authors of included studies to determine (1) why they chose the CFIR as a guiding framework, (2) which domains and constructs they found to be compatible, incompatible, or irrelevant to their research and why, and (3) ideas for how the CFIR could be optimized or updated for use in LMIC contexts. Conference abstracts were reviewed to identify potential questionnaire participants with pending publications.

**Findings:** Twenty-seven (10%) of 266 unique articles met inclusion criteria following a two-stage process of title and abstract review, followed by full text review. The majority (52%) of CFIR applications were applied post-implementation, as opposed to pre- or mid-implementation. Similarly, 37% and 32% of CFIR applications were used to guide data analysis or contextualize study findings, respectively. Only 2 (7%) studies investigated outcomes linked to specific CFIR constructs and 5 (19%) of studies provided some justification for selecting the CFIR constructs used. Twelve (44%) of 27 contacted authors responded to the study questionnaire. The construct most frequently reported to be compatible for use in LMICs was “intervention source”, while the most frequently reported incompatible construct was “patient needs and resources”. Common feedback regarding potential LMIC framework improvements included the need to introduce constructs related to health system factors influencing intervention sustainability (ex. multi-organization partnerships).

**Implications for D&I Research:** Evaluating and optimizing one of the major IS frameworks for use within LMIC contexts is likely to expand and enhance the impact of IS in LMICs.

### S82 Effectiveness of strategies to improve health care provider practices in low- and middle-income countries: A systematic review

#### Alexander Rowe^1^, Samantha Rowe^1^, David Peters^2^, Kathleen Holloway^3^, Dennis Ross-Degnan^4^

##### ^1^Centers for Disease Control and Prevention, Atlanta, GA, USA; ^2^Johns Hopkins Bloomberg School of Public Health, Baltimore, MD, USA; ^3^Institute of Development Studies, University of Sussex, Brighton, United Kingdom; ^4^Harvard Medical School and Harvard Pilgrim Health Care Institute, Boston, MA, USA

###### **Correspondence:** Alexander Rowe (axr9@cdc.gov)

**Background:** Inadequate health care provider (HCP) performance is a major challenge to delivering health care in low- and middle-income countries (LMICs). The Health Care Provider Performance Review (HCPPR) is a systematic review of strategies to improve HCP performance in LMICs. The HCPPR aims to advance the science of disseminating clinical guidelines and implementing strategies to promote guideline adherence. We present results on improving HCP practice outcomes expressed as continuous measures or percentages (e.g., percent of patients treated correctly).

**Methods:** We searched 52 databases and 58 document inventories for published and unpublished studies from 1960s–2016. Eligible study designs were controlled trials and interrupted time series. Effect sizes were calculated as absolute percentage-point (%-point) changes. The summary measure for each study comparison was the median effect size (MES) for all primary outcomes. Strategy effectiveness was described with weighted medians of MES.

**Findings:** We screened 216,444 citations and included 637 reports from 327 studies of 115 strategies in 63 countries. Most studies (>80%) were published after 2000. The median MES across all studies were improvements of 11.2 and 11.1 %-points for percentage and continuous outcomes, respectively. For facility-based HCPs, strategy effects were near zero for only implementing a technology-based strategy or only providing printed information for HCPs. For percentage outcomes, training or supervision alone typically had moderate effects (9.5–14.9 %-points), whereas combining training and supervision had somewhat larger effects (17.8–18.3 %-points). Effects of training alone appeared to wane over time. Group problem solving alone typically led to moderate to large improvements. Several multi-faceted strategies had large effects, but multi-faceted strategies were not always more effective than simpler ones. For community health workers, the effect of training alone was modest (7.3 %-points). Strategies with larger effect sizes included community support plus HCP training (10.4–113.7 %-points). Contextual and methodological heterogeneity made comparisons difficult, and most strategies had low-quality evidence.

**Implications for D&I Research:** The impact of strategies to improve HCP practices varied substantially, although some approaches were more consistently effective. The HCPPR’s results and publicly available database will support local decision-makers and implementation science researchers in their efforts to scale-up and sustain improved HCP practices in resource-constrained settings.

### S83 Can mhealth messages improve management of diabetes and hypertension through a peer educator model in Cambodia?

#### Lesley Steinman^1^, Annette Fitzpatrick^1^, Maurits van Pelt^2^, Heang Hen^2^, Mayuree Rao^3^, Nicole Ide^1^, Vannarath Te^4^, Jim LoGerfo^1^

##### ^1^School of Public Health, University of Washington, Seattle, WA, USA; ^2^MoPoTsyo, Phnom Penh, Cambodia; ^3^School of Medicine, University of Washington, Seattle, WA, USA; ^4^National Institute of Public Health, Ministry of Health (Cambodia), Phnom Penh, Cambodia

###### **Correspondence:** Lesley Steinman (lesles@uw.edu)

**Background:** The burden of non-communicable diseases (NCDs) is increasing, particularly in low- and middle-income countries (LMICs) in epidemiological transition. mHealth (mobile health) interventions can improve NCD management in LMICs given limited health system infrastructure and high cell-phone coverage. We conducted a hybrid effectiveness-implementation study in Cambodia to evaluate an mHealth intervention for diabetes and hypertension.

**Methods:** Using RE-AIM, we partnered with Cambodian NGO (MoPoTsyo) on a cluster randomized controlled trial. MoPoTsyo trains people with NCDs as peer educators (PEs) to provide self-management education, support, and healthcare linkages. We conducted interviews and focus groups with patients and PEs to develop mHealth message content and format. Clusters of 25 PEs were randomized to mHealth or usual care from Sept 2017–Feb 2018. Our binary independent variable is receipt of >/= one message. Primary dependent variables are changes in pre/post SBP, DBP and FBG. Intermediate outcomes are medication adherence, labs and doctor’s visits. We used linear mixed effects and GLM to estimate patient- and cluster-level effects. To evaluate implementation, we collected process data from MoPoTsyo’s database and in Aug 2018 are conducting semi-structured interviews using CFIR constructs (intervention and individual characteristics; inner and outer setting) with 20 patients that did/did not receive messages.

**Findings:** There were 3,604 participants (1,355 treatment, 2,249 control): mean age 57.4 (SD 11), 65% female, 54% rural. Patients had well-controlled BP (SBP 127, DBP 79) and poor glucose control (FBG 167). Patients were sent 30 (SD 14) messages and received 14 (SD 8); 40% received no messages due to phone number changes. Preliminary analyses suggest no significant improvements in primary or intermediate outcomes; we are currently evaluating effectiveness of targeted, tailored messages (e.g., Rx adherence reminders for Rx non-adherers). Our presentation will share these sub-analyses and facilitators and barriers to implementation and sustainability from the interviews.

**Implications for D&I Research:** We evaluated an NCD mHealth intervention in an LMIC (for which evidence is mixed) using established D&I frameworks. mHealth alone may be insufficient for improving outcomes for people and systems with limited resources. However, this study provides important implementation learnings for future efforts to improve NCD management in Cambodia.

### S84 Implications for adapting a family-completed well-visit tool for the Mexican public healthcare system

#### Lisa Schalla^1^, Christina Bethell^2^

##### ^1^Fundación Punta de Mita, Bahía de Banderas, Mexico; ^2^Johns Hopkins Bloomberg School of Public Health, Baltimore, MD, USA

###### **Correspondence:** Lisa Schalla (lisa@fundacionpuntademita.org)

**Background:** Pediatric care is essential for promoting early and lifelong health among children and youth, and with a poverty level of over 43%, particularly vulnerable families in Mexico face an array of challenges that prevent policy on preventive health from being enacted in practice. We introduced an evidence-based, online tool designed to promote trust and mutual respect between patients and healthcare providers, thus increasing the likelihood that parents continue with regular well visits. The already existing Spanish version of the Well-Visit Planner was revised for language use more appropriate to the local setting and changes were made in accordance with contextual practices. We then used implementation facilitation over 6 months with two public healthcare facilities and 40 families in three semi-rural communities in western Mexico.

**Methods:** We applied surveys to parents (n=44) and conducted 7 focus groups (n=29) to assess attitudes about current family health practices. We also interviewed 3 public health practitioners before and after the implementation of the Well Visit Planner in their clinics. The implementation team, consisting of a coordinator and three community leaders, introduced the tool to parents and had weekly meetings to discuss progress. We conducted semi-structured interviews with community leaders (n=3) and reviewed meeting notes. To guide our analysis, we used the Phases of Implementation Facilitation model (Ritchie et.al., 2017) and made adjustments to our model accordingly.

**Findings:** Although the intervention was well received among some participants, contextual organizational and individual barriers persisted that interfered with the implementation strategy. We identified primary enablers and disablers to implementation and 5 areas to improve for further effectiveness: 1) invitations for increased participation of state-level stakeholders, 2) application of a stronger focus on implementation fidelity within the cultural context, while 3) further adapting documentation and processes for cultural relevance, 4) development of further resources and next-step strategies following well visits, and 5) invitations for increased stakeholder participation for implementation refinement.

**Implications for D&I Research:** Opportunities exist for further multi-sector and international partnerships to further refine this implementation strategy with families both in Mexico and where Mexican families reside in the U.S. and Canada.

### S85 Hiding in plain sight: The health system costs of transitioning to extended antiretroviral therapy refills for HIV in Africa

#### Kaitlyn McBride^1^, Corrina Moucheraud^1^, Risa Hoffman^3^

##### ^1^UCLA Fielding School of Public Health, Los Angeles, CA, USA; ^2^David Geffen School of Medicine at the University of California, Los Angeles, CA, USA

###### **Correspondence:** Kaitlyn McBride (kaitlynbmcbride@gmail.com)

**Background:** When new health care approaches are implemented, new costs may be introduced -- but these are not well characterized in traditional cost-effectiveness analyses. Here we use differentiated models of care, which are being adopted in high HIV-prevalence settings to rapidly increase the number of people receiving antiretroviral therapy (ART), as a case study for a new approach to quantifying these system costs; namely: what are the health system costs of expanding infrastructure to allow multi-month dispensing (MMD) which extends refills of ART to longer intervals of up to six months (e.g., supply chain, storage, transportation, etc.). Our objective is to propose a standardized framework that incorporates all cost components from a health system perspective, which can be used for future economic evaluations and cost-effectiveness studies of MMD and other new models of care.

**Methods:** A targeted literature review of existing costing methodologies, conceptual frameworks, and economic evaluations on the costs of scaling-up health interventions was conducted to inform the development of a new costing framework. Key principles were compared across various health interventions and models based on the scale-up of HIV, reproductive health and immunization programs. To operationalize our proposed framework, principal cost components across existing models were identified and categorized according to the World Health Organization health system building block model.

**Findings:** The literature contains no existing framework to quantify system costs that accompany new care models; thus current cost-effectiveness estimates may omit important expenses, and cost estimates may underestimate the budgetary impact to health systems. The proposed framework integrates health system components to capture the costs and financing involved for MMD, and demonstrates applicability to other care models.

**Implications for D&I Research:** Accurate costing data is essential for cost-effectiveness analyses, to inform policies and resource allocation decisions. Failure to comprehensively include all health system costs may impact the long-term financial sustainability of new programs, and miscalculate the actual “cost-effectiveness” of a particular intervention. A standard costing framework to estimate the total costs to the health care system will enable more robust and inclusive cost-effectiveness studies in the future.

### S86 Scaling up interventions to improve management and control of hypertension and diabetes in primary care settings: The context matters

#### Vilma Irazola (virazola@iecs.org.ar)

##### Institute for Clinical Effectiveness and Health Policy (IECS), Buenos Aires, Argentina

**Background:** Diabetes and hypertension are important global health challenges because of an increasing prevalence in low- and middle-income countries (LMIC) and high cardiovascular disease (CVD) disease burden. According to the International Diabetes Federation (IDF), 425 million of the global population have diabetes and three quarters live in LMICs. The prevalence is higher in LMICs but is steady or decreasing in high-income countries. Additionally, hypertension is a global public health challenge because of its high prevalence and the concomitant increase in risk of CVD. The highest estimated prevalence of hypertension in the world is in Latin America: 40.7% in men and 34.8% in women. Hypertension is a leading global risk factor for CVD and premature death. Given the high rates of hypertension and diabetes and their highly prevalent coexistence, this project focuses on scaling up two successful interventions at the primary care level in the public health system in Argentina.

**Methods:** Considering key contextual factors and challenges in delivering services oriented to the management of highly prevalent chronic conditions such as hypertension and diabetes, two successful interventions approaching barriers at the health system, health care provider and community levels will be presented. The first is a comprehensive intervention to improve management of poorly controlled hypertensive patients based on task shifting, self-monitoring and m-health. The second intervention is a comprehensive strategy for management of diabetic patients focused on capacity building, m-health and patient registries. Both are being adapted for a broader implementation using a sequential approach. Key factors influencing the process of adaptation and scaling up will be discussed.

**Findings:** Several key barriers and facilitators to the integration of complex multifactorial interventions directed to improving management of chronic conditions in vulnerable populations were identified.

**Implications for D&I Research:** There are key factors that can help advance D&I science in vulnerable populations, in particular the assessment of context in a more systematic way so as to make interventions more feasible and scalable.

### S87 Scaling up or scaling out: Using interagency collaborative teams to integrate and sustain evidence-based mental health interventions linked to youth employment programs in Sierra Leone

#### Theresa Betancourt (theresa.betancourt@bc.edu)

##### School of Social Work, Boston College, Chestnut Hill, MA, USA

**Background:** An estimated 10-20% of children globally are affected by mental health disorders. The mental health of children and youth and its links to other domains of social functioning such as success in school and employment, is gaining growing recognition as a priority area for major development actors and governments. In conflict-affected regions, mental health issues facing children and youth are even greater given significant loss and trauma suffered by such populations and massive treatment gaps. Sierra Leone has limited mental health care infrastructure, a shortage of mental health workers and a considerable treatment gap. Given high rates of mental health problems and limited access to mental health services, this study aims to implement a model leveraging lay workers and using an alternative delivery platform to scale up access to an evidence-based mental health intervention.

**Methods:** In consideration of the contextual realities and challenges in delivering mental health services in Sierra Leone, coupled with the government’s commitment to youth employment and economic development, the Youth FORWARD scale-up study aims to investigate the delivery of the cognitive-behavioral therapy-based Youth Readiness Intervention (YRI) within the alternate setting of youth employment programs tied to regional economic development. The study will also examine the use of an Interagency Collaborative Team Approach (ICTA) as an implementation strategy that addresses the human resource, access to care, and capacity challenges in low-resource settings. A Hybrid Type 2 Effectiveness-Implementation Cluster Randomized Three-arm trial involving 1200 youth will be employed with a focus on process and implementation elements including a costing analysis, measures of fidelity, and the sustained delivery of intervention delivery, training and supervision.

**Findings:** The EPIS (Exploration, Preparation, Implementation, and Sustainment) model and qualitative elements of the Hybrid Type II Design illuminated key barriers and facilitators to integration of mental health into youth employment programs in Sierra Leone.

**Implications for D&I Research:** There are exciting innovations that can help to advance D&I science in fragile and post-conflict settings, these include the use of alternative delivery platforms, approaches to triangulating qualitative and quantitative data, fidelity monitoring to ensure quality improvement and sustainment of best practices using strategies such as interagency collaborative teams.

### S88 Bringing evidence-based science to scale in the maternal child health space

#### Sarah Gimbel, Kenneth Sherr

##### University of Washington, Seattle, WA, USA

###### **Correspondence:** Sarah Gimbel (sgimbel@uw.edu)

**Background:** With the scale up of evidence-based interventions, under-5 mortality has reduced dramatically globally, but gains have stagnated due to persistent neonatal mortality. Evidence-based interventions delivered at or around the time of birth exist; however, a greater focus on improving the quality of how implementation strategies are scaled is needed to ensure adoption, routine use and impact on population-level health.

**Methods:** A comparative case study will present two ongoing scale-up studies in Mozambique (Integrated District Evidence to Action, or IDEAS; and Scaling up the Systems Analysis and Improvement Approach, or SAIA-Scale), to highlight drivers and barriers for scale-up/scale-out and provide actionable evidence for national decision makers. Our analysis will focus on comparative designs, roles of actors, the implementation process, and specifically how implementation sciences methods can improve the transferability of results both horizontally and vertically.

**Findings:** Using Aaron’s ‘scaling-up’ versus ‘scaling-out’ framework, we found differences when expanding the reach of an evidence-based intervention to a similar population (IDEAS) versus introducing an evidence-based intervention to a new population of implementers (SAIA-Scale). In the former, a traditional cluster RCT design provides more robust evidence, with higher internal validity but quasi-experimental design is better suited to the scaling-out model (IDEAS), which required understanding variations in improvement rather than simply average change. Budget impact analysis targeting the needs of policymakers is crucial in both study designs, and implementation science frameworks such as RE-AIM support transferability. CFIR and process evaluation methods document fidelity to intervention as well as identification of which intervention components remain core as they are brought to scale.

**Implications for D&I Research:** This comparative analysis of two scale-up studies in maternal and child health highlights drivers for scale and the importance of actors and design elements to efficiently and effectively expand evidence-based interventions. Highlighting differing challenges between scaling-up to scaling-out (to new populations/users, geographic settings, or service delivery areas) can improve the transferability of findings as implementation moves towards becoming the science of going to scale.

### S89 Using dissemination and implementation research to address the demands of rapid scale up of multiple evidence-based interventions in the context of HIV prevention care and treatment programs

#### Laura Guay^1,2^, Vincent Tukei^3^, Appolinaire Tiam^1^, Lynne Mofenson^1^

##### ^1^Elizabeth Glaser Pediatric AIDS Foundation, Washington, DC, USA; ^2^George Washington University School of Public Health, Washington, DC, USA; ^3^Elizabeth Glaser Pediatric AIDS Foundation, Maseru, Lesotho

###### **Correspondence:** Laura Guay (lguay@pedaids.org)

**Background:** Significant progress has been made in the prevention of mother-to child transmission of HIV (PMTCT) globally due to the implementation of medical and programmatic interventions based on rigorous randomized study data. However, inefficient, poorly coordinated and fragmented services may contribute to the lack of expected benefits achieved with the scale-up of evidence-based interventions (EBI). The IMPROVE study in Lesotho is evaluating a multidisciplinary approach to improve the effectiveness of existing facility and community-based interventions on maternal and child health (MCH) and PMTCT program outcomes.

**Methods:** The IMPROVE intervention includes: (1) Multidisciplinary integrated management teams to coordinate patient-focused and outcome-oriented PMTCT and MCH services in select facilities; (2) Enhanced *Positive Health, Dignity, and Prevention-focused* counseling, skills building, and job aids for health workers; and (3) Increased early community-based support for first-time antenatal clinic (ANC) attendees. This cluster randomized study includes 12 facilities that receive either the IMPROVE intervention or routine services. 620 HIV-positive and 400 HIV-negative pregnant women were enrolled with follow-up until their child reaches 12-24 months of age. The objectives are to determine the effect of the intervention on selected HIV prevention and treatment (re-testing, retention, viral suppression) and MCH outcomes (ANC attendance, facility delivery, family planning, and immunization coverage).

**Findings:** Lessons learned during the conduct of this study include challenges faced by facility-level health care workers with managing multiple new interventions at the same time, addressing EBI fidelity vs. adaptability in their setting, and increasing data collection and reporting demands. Significant gaps remain in the actual vs. expected outcomes achieved with implementation of known-effective interventions in the context of rapid emergence and demands for scale-up of new strategies to improve HIV prevention, care and treatment services.

**Implications for D&I Research:** Research utilization needs to include real-time feedback of important lessons learned during the conduct of the study to Ministries of Health and program implementers so that D&I research adds value to stakeholders throughout the process, not just at the time of final study results. In the context of rapidly changing guidelines and increasing focus on achieving program outcome targets, designing D&I research that is responsive to the needs of the end-users is critical.

### S90 Scalability assessment of innovations in primary health care: a cross-sectional study

#### Ali Ben Charif^1^, Kasra Hassani^2^, Sabrina T Wong^2^, Hervé Tchala Vignon Zomahoun^3^, Martin Fortin^4^, Adriana Freitas^5^, Alan Katz^6^, Claire Kendall^7^, Clare Liddy^8^, Kathryn Nicholson^9^, Bojana Petrovic^10^, Jenny Ploeg^11^, France Legare^12^

##### ^1^Université Laval, Centre de recherche sur les soins et les services de première ligne de l'Université Laval (CERSSPL-UL), Quebec, QC, Canada; ^2^University of British Columbia, School of Nursing and Centre for Health Services and Policy Research and School of Nursing, Vancouver, BC, Canada; ^3^Université Laval, The Quebec SPOR-SUPPORT Unit, Quebec, QC, Canada; ^4^Université de Sherbrooke, Department of Family Medicine and Emergency Medicine, Sherbrooke, QC, Canada; ^5^Université Laval, Tier 1 Canada Research Chair in Shared Decision Making and Knowledge Translation, Quebec, QC, Canada; ^6^University of Manitoba, Departments of Community Health Sciences and Family Medicine, Rady Faculty of Health Sciences, Winnipeg, MB, Canada; ^7^C.T. Lamont Primary Health Care Research Group, Bruyère Research Institute, Ottawa, ON, Canada; ^8^Family Medicine, C.T. Lamont Primary Health Care Research Centre, Bruyère Research Institute, Ottawa, Ontario, Canada, Ottawa, ON, Canada; ^9^McMaster University, Department of Health Research Methods, Evidence, and Impact, Hamilton, ON, Canada; ^10^University of Toronto, Department of Family and Community Medicine and Dalla Lana School of Public Health, Toronto, ON, Canada; ^11^McMaster University, School of Nursing, Faculty of Health Sciences, Hamilton, ON, Canada; ^12^Université Laval, Department of Family Medicine and Emergency Medicine, Québec, QC, Canada

###### **Correspondence:** Ali Ben Charif (ali.ben-charif.1@ulaval.ca)

**Background:** Over the past five years, the Canadian Institutes of Health Research have funded 12 community-based primary healthcare teams (“12-Teams”) to develop evidence-based innovations (EBIs). Before EBIs can benefit larger populations, their scalability must be assessed. We sought to take an in-depth look at the scalability potential of these EBIs.

**Methods:** In a cross-sectional study, we invited the 12-Teams to rate their EBIs for scalability potential. Based on a systematic review, we developed a self-administered questionnaire with 16 scalability assessment criteria grouped into five dimensions (theory, impact, coverage, setting, and cost). The teams completed distinct questionnaires for each of their EBIs. We analyzed data using simple frequency counts and a hierarchical cluster analysis. We calculated mean number and standard deviation (SD) of EBIs that met criteria within each dimension including more than one criterion. The analysis unit was the EBI.

**Findings:** Eleven responding teams evaluated 33 EBIs (median=3, range=1-8 per team). Most EBIs were health interventions (n=21), followed by analytical methods (n=4), conceptual frameworks (n=4), measures (n=3), and research capacity building strategies (n=1). Most EBIs met criteria in the theory dimension (n=29), followed by impact (mean=22, SD=6), setting (mean=22, SD=9), cost (mean=18, SD=2), and lastly, coverage (mean=14, SD=4). On average, the EBIs met 10 of the 16 scalability assessment criteria. Adoption was the least assessed criterion (n=9). Most EBIs were highly ranked for scalability potential (n=20).

**Implications for D&I Research:** Scalability potential varied among EBIs, suggesting the readiness for scale up was suboptimal for some EBIs. Coverage is a dimension that remains largely unaddressed; consequently future evaluations of the teams’ activities should investigate criteria relating to this critical dimension.

### S91 Identifying optimal approaches to scale up cervical cancer screening in sub-saharan africa: A multimethod approach

#### Sujha Subramanian (ssubramanian@rti.org)

##### RTI International, Waltham, MA, USA

**Background:** Around the world a women dies of cervical cancer about every 2 minutes, with more than 500,000 new cases and 250,000 deaths. Sub-Saharan African countries experience high mortality rates and although several cervical cancer screening programs have been initiated, there is limited evidence on the role of integrated HIV and cervical cancer interventions to efficiently scale up services to address the dual burden of these diseases.

**Methods:** We initiated a multimethod study to systematically address the objective of identifying optimal, cost-effective strategies to reduce cervical cancer burden. First, we developed a novel microsimulation model to assess cost-effectiveness of strategies to scale up cervical cancer prevention and screening at the population level in Sub-Saharan Africa under ‘real-world’ conditions that include HIV prevention and treatment interventions, human papillomavirus (HPV) vaccination and cervical cancer screening. Second, we conducted multiple focus groups and interviews with HIV positive and HIV negative women eligible for screening to gain a qualitative understanding of barriers and facilitates to screening from a demand generation perspective. Third, we performed a process evaluation of the government implemented program in Zambia to assess supply-side barriers to the delivery of high-quality services along the entire continuum of care.

**Findings:** Results from the microsimulation model indicate that screening strategies cost between $508 to $1,810 per life year saved, making the majority of these interventions highly cost-effective. HPV vaccination results in cost savings but will need to be combined with screening as the benefits will not be achieved for at least 20 years. HIV prevention is generally less cost-effective than cervical cancer screening because of the low effectiveness of many interventions in preventing HIV transmission. Our qualitative findings highlight the need for targeted education as women are likely to only consider screening when they experience symptoms rather than seeking screening to prevent cervical cancer. From a programmatic perspective, training and maintaining core staff with cervical cancer screening expertise and experience is a major challenge for scale up and sustainability.

**Implications for D&I Research:** Lessons learned can support future program implementation by ensuring steps are taken to address barriers identified to support both effectiveness and sustainability of cervical cancer screening delivery.

### S92 A novel strategy to implement community-based HIV counseling services: Engaging traditional healers to translate clinical messages to a low-literacy population

#### Carolyn Audet^1^, Jose Salato^2^

##### ^1^Vanderbilt University Medical Center, Nashville, TN, USA; ^2^Community Health, Friends in Global Health, Quelimane, Mozambique

###### **Correspondence:** Carolyn Audet (carolyn.m.audet@vanderbilt.edu)

**Background:** In rural Mozambique, 35% of newly diagnosed people living with HIV (PLHIV) abandon treatment within six months. It has been hypothesized that PLHIV need longer and more frequent counseling sessions to facilitate engagement in care. With few trained counselors at each facility, demand has outstripped supply. Traditional healers, adversaries-turned-supporters of the health system, have expressed interest in providing psychosocial support.

**Methods:** We adapted a formal HIV counseling program for use by semi-literate traditional healers in Namacurra district, Mozambique. Interested healers were provided three weeks of counseling and HIV education training. Newly diagnosed patients selected one of 42 traditional healers via “facebook”-style picture books. Healers conducted counseling sessions at the patients home every two weeks for the first two months, and monthly thereafter for one year. They received 25 USD/month and a bicycle as compensation.

**Findings:** We offered 160 patients the opportunity to work with a traditional healer; 152 accepted (95% acceptance rate). Only 27 of the 42 healers (64%) were selected as treatment supporters. Patients had a median age of 30 (IQR: 24-39), 77 were women (51%), and 97 (64%) were married. Since October 2017, healers have conducted 771 home-based counseling sessions (median time: 27 minutes (IQR: 19-31)). One hundred fifteen patients (77%) have received every recommended counseling session, with 26 patients (17%) receiving more than the required number of sessions. Healers have conducted at least one family-based session with 146 (94%) of their patients. Healers have delivered more than counseling support: 14 (52%) report picking up patient medications, 12 (44%) have provided food to patients in need, and 19 (70%) have accompanied a patient back to the health facility for follow-up services. Patients reported high levels of satisfaction with their healer, noting their appreciation for the auxiliary services provided (specifically drug pick-up).

**Implications for D&I Research:** Healers can provide feasible, acceptable, and relatively inexpensive additional counseling support to newly diagnosed HIV patients in Mozambique. Comparison of patient health outcomes and retention in care with a group of patients who selected but did not receive healer support will determine if this strategy should be implemented in areas were the health system is under-capacitated.

### S93 Evaluation of the national scale up of chlorhexidine cord cleansing in Bangladesh

#### Jennifer Callaghan-Koru^1^, Marufa Khan^2^, Munia Islam^2^, Ardy Sowe^3^, Jahrul Islam^4^, Imteaz Mannan^2^, Joby George^2^

##### ^1^University of Maryland-Baltimore County, Baltimore, MD, USA; ^2^Save the Children International, Dhaka, Bangladesh; ^3^Howard University School of Medicine, Washington, DC, USA; ^4^Ministry of Health and Family Welfare, Bangladesh, Dhaka, Bangladesh

###### **Correspondence:** Jennifer Callaghan-Koru (jck@umbc.edu)

**Background:** Chlorhexidine (CHX) cleansing of the umbilical cord stump is an evidence-based intervention that reduces newborn infections and is recommended for high-mortality settings. Bangladesh is the first country to adopt CHX and this study evaluates the implementation outcomes for the scale up.

**Methods:** An adapted RE-AIM framework, that incorporates elements from the WHO/ExpandNet model of Scale Up, guided this study. Adoption and incorporation milestones were assessed through program documents and interviews with national stakeholders (n=25). Program records of national provider training served as a measure of reach. Implementation was assessed through a post-training assessment of public facilities (n=4,479) and routine data on the proportion of all live births at public facilities (n=813,607) that received CHX. Six rounds of a rolling household survey with recently-delivered women in four districts (n>6,000 per round) measured the effectiveness and maintenance of the scale up in increasing coverage of CHX in those districts.

**Findings:** More than 80,000 providers, supervisors, and managers across all 64 districts received a half-day training on CHX and essential newborn care. Delays with institutionalization within the supply distribution system were a major bottleneck for scale up—many providers were trained six months to a year before CHX was available at their facilities. On the day of assessment, 74% of facilities had at least 70% of maternal and newborn health providers with CHX training, while only 46% had CHX in stock. The provision of CHX to newborns delivered at public facilities steadily increased from 15,059 newborns (24%) in December 2016 to 71,704 (72%) in November 2017. In the final household survey of four districts, 33% of newborns received CHX, and babies delivered at public facilities had 5.04 times greater odds (95% CI: 4.45, 5.72) of receiving CHX than those delivered at home.

**Implications for D&I Research:** The findings from this study highlight the importance of institutionalization milestones for successful scale up. The example of commodity procurement and distribution bottlenecks during the scale up of CHX in Bangladesh demonstrates that these institutionalization outcomes are critical in achieving scale up. Institutionalization milestones should be included as outcomes in implementation studies and frameworks.

## Health Policy Dissemination and Implementation Science

### S94 How do medical groups use externally required measures in their own quality improvement efforts?

#### Linda Bergofsky^1^, Dr. Peggy Chen^2^, Michael I. Harrison^1^, Denise St.Clair^3^, Russ Mardon^4^, Laura Raaen^2^, Andrada Tomoaia-Cotisel^2^, Lisa Lentz^4^, Andrew Hinzman^4^, Joshua Rubin^4^, Mark Friedberg^5^

##### ^1^Agency for Healthcare Research and Quality, Rockville, MD, USA; ^2^RAND, Santa Monica, CA, USA; ^3^Centers for Medicare & Medicaid Services, Baltimore, MD, USA; ^4^Westat, Rockville, MD, USA; ^5^RAND, Boston, MA, USA

###### **Correspondence:** Linda Bergofsky (linda.bergofsky@ahrq.hhs.gov)

**Background:** Payer and regulatory policies require physician practices to report on increasingly complex measures of quality, cost, and patient experience as part of paying for value. These policies may promote internal efforts to improve performance, but little is known about how medical groups drive internal improvements. In the context of ubiquitous “external” measure requirements, why do some groups gather extra “internal” measures, and how do they use these data to drive improvement? In response to such questions, we conducted a national, exploratory study.

**Methods:** We conducted 83 semi-structured phone interviews with 91 informants in 37 small, medium, and large medical groups that engaged in some form of internal measurement. Interviewers used a guide reflecting previous instruments, expert panel advice, and piloting in 5 practices. Informants included chief executives, medical directors, quality officers, information/data leads, clinic managers, and clinicians. A multi-disciplinary team used cyclical coding to identify interview themes.

**Findings:** Eight groups – mostly ones with limited measurement experience - reported measures in response to external requirements, with limited use of internal measures to drive improvement. Other groups used additional measures of clinical quality, business processes, and member experience internally to guide improvement efforts targeted to both external quality reports and internal strategic targets. Groups used internal measures to improve clinical processes, check accuracy of external reports and/or provide feedback and guidance to low-performing clinics and clinicians. Some groups added internal measures in anticipation of the Medicare Access and CHIP Reauthorization Act. None yet used standardize patient-centered outcome measures. Substantial infrastructure for data management, experience sharing data within the organization, and alignment of measures with organizational strategic goals characterized the groups best able to benefit from internal performance measurement

**Implications for D&I Research:** Current payment and reporting policies influence measurement activities of medical groups. However, the leading groups in this study did not find externally required measures sufficient for their own mission-driven needs, and hence often collected additional measures for internal performance monitoring and improvement. Respondents discussed the burden of misaligned external measurement requirements. Research is needed to assess how payment and reporting policies influence measurement and improvement activities in less advanced groups.

### S95 Increasing transparency about ACOs: findings from Massachusetts’ ACO certification program

#### Catherine Harrison, Kelsey Brykman, Courtney Anderson, Vivian Haime, Katherine Barrett

##### Massachusetts Health Policy Commission, Boston, MA, USA

###### **Correspondence:** Catherine Harrison (catherine.harrison@mass.gov)

**Background:** In 2017, the Massachusetts Health Policy Commission (HPC) launched a first-in-the-nation certification program that sets statewide standards for accountable care organizations (ACOs) designed to encourage the provision of value-based, high quality care for all ACO members. The ACO Certification program recognizes providers that demonstrate key capabilities such as patient-centered, accountable governance, participation in quality-based risk contracts, population health management, and cross-continuum care. In the first year of the program, the HPC certified 17 Massachusetts ACOs. An important aim of the program is to increase transparency about ACO structures and operations and help identify and disseminate innovative approaches. To that end, the HPC is publishing policy briefs and ACO profiles summarizing information from the 2017 certification applications.

**Methods:** HPC staff analyzed 17 applications to identify trends in ACO characteristics and operations. The applications include a mix of qualitative data (e.g., narrative text and official documents) and quantitative data (e.g., multiple choice questions and risk contract information). Data were systematically extracted and cohort-level findings compiled into a series of policy briefs and one-page profiles.

**Findings:** The policy briefs and profiles introduce the landscape of certified ACOs and explore topics such as population health management and risk contract experience. Examples of key findings include: Approximately 1.9 million commercial or Medicare patients and some 850,000 Medicaid members in Massachusetts are served by HPC-certified ACOs; Certified ACOs have a total of 66 commercial, 17 Medicaid, and 11 Medicare risk contracts; Over 80% of certified ACOs are anchored by an academic or teaching hospital; Most ACOs collect patient data on socio-demographic factors such as race, ethnicity, language, and history of abuse/trauma; fewer than half collect information on housing status, income, food insecurity and other social determinants of health (SDoH); Most ACOs include payer reports, claims, and clinical data in their risk stratification methods; few incorporate factors related to behavioral health or SDoH.

**Implications for D&I Research:** The HPC’s ACO Certification program provides a framework for increasing transparency and disseminating information about ACOs in order to accelerate care delivery transformation. Findings can help guide states in developing policies to promote high-value health care, and support payers and providers in managing risk contracts.

### S96 Leveraging High Value Healthcare Collaborative assets to adopt new payment models

#### Jay Knowlton^1^, Thomas Belnap^2^, Stephen Kearing^1^, Ronald Russell^1^, Gregory Bennett^3^

##### ^1^High Value Healthcare Collaborative, Portland, ME, USA; ^2^Intermountain Healthcare, Salt Lake City, UT, USA; ^3^Northwell Health, Manhasset, NY, USA

###### **Correspondence:** Jay Knowlton (jay.s.knowlton@dartmouth.edu)

**Background**: In January 2018, Medicare announced the Bundled Payments for Care Improvement Advanced initiative (“BPCI-A”) along with a short timeline for providers to assess the program opportunity and apply. As providers work to determine which bundles to participate in, data is key to understand opportunity and manage risk. Members of the High Value Healthcare Collaborative (“HVHC”), a group of ten healthcare delivery systems across the US, are working together to conduct detailed analyses beyond – and complementary to – data provided by CMS to understand the impact of adopting the BPCI-A payment model.

**Methods**: HVHC analysts constructed eligible episodes for each BPCI-A inpatient bundle using Medicare claims from 2013-2016. Reports were generated for all HVHC Member facilities, which included acute and post-acute service utilization metrics (e.g., length of stay, discharge disposition, readmissions rates) as well as episode cost metrics. Member performance was compared to a proxy national benchmark to indicate opportunities for improvement on key performance indicators. HVHC also convened subject matter experts from each Member system and conducted several meetings to review reports and discuss delivery system strategies for the BPCI-A program application.

**Findings**: While CMS provided BPCI-A data to providers who submitted applications, leveraging HVHC’s CMS data allowed Members to receive reports sooner and compare respective internal data to HVHC reports. HVHC provided enhanced drill-down and customization capability as well as the ability to assess BPCI-A opportunities compared to those of other CMS programs. HVHC ongoing monitoring capability allows Members to monitor performance for all bundles, regardless of participation in BPCI-A, to help them assess opportunities for the next application cycle. Six of ten HVHC Members submitted applications for the BPCI-A program and all Members indicated value in using HVHC analyses for advanced insights, robust benchmarking, and shared learning.

**Implications for D&I Research**: Provider collaboratives with access to claims data are uniquely positioned to rapidly assess new payment models, disseminate shared learnings, and facilitate implementation of promising practices.

### S97 Exploring the implementation of coordinated care within a context of systems change – a need for ‘project resilience’

#### Laura Holdsworth (l.holdsworth@stanford.edu)

##### Stanford University School of Medicine, Stanford, CA, USA

**Background:** To meet the multidimensional needs to patients, health services are increasingly implementing complex collaborative programs of care through partnerships between public, private and voluntary sector organizations. However, there is a gap in understanding how complex, multi-innovative programs are implemented by partnerships, particularly since such interorganizational complexity can compound the threat from political, social, and economic changes. This study explored the implementation process, of a regional health and social care partnership project aimed at implementing the aims of the UK’s National End of Life Care Program to coordinate end of life care in the South East of England.

**Methods:** The study adopted a pragmatic, pluralist design using primarily qualitative methods including: document review, observations, interviews, and focus group with stakeholders. Implementation theory, particularly drawing on the Consolidated Framework for Implementation Research, provided the research framework.

**Findings:** While progress was made towards greater collaboration in the provision of end-of-life care, regional coordination of care among the thirteen partners was not achieved as envisioned within the two-year project period. Low engagement among stakeholders, stemming from national health system changes, delayed decision making and shifted partners’ priorities away from the project and towards internal organizational goals. Individual stakeholder interest and personal motivation to improve care carried the elements that were successful. The external political and economic environment hindered the involvement of some of the partners and suggests that a concept of ‘project resiliency’ is particularly important for complex, multi-organizational projects that are implemented over time and by multiple stakeholders from different sectors. Project resilience appears to be the product of implementation strategies, innovation adaptiveness, and implementer characteristics.

**Implications for D&I Research:** Project resiliency is a new concept that bridges a gap in understanding how time-limited multi-organizational projects function amid a changing political and organizational environment. Future research should look further at what contributes to project resilience and whether it might be operationalized so that projects can develop resilient factors to ensure their success.

### S98 C.H.A.R.T. playbook: Operational insights for population health management derived from 27 Massachusetts community hospital care transformation initiatives

#### Gabriel Malseptic, Tayler Bungo, Kevin Comeau, Louise Secordel, Daniel Feldman, Jessica Lang, Kathleen Connolly

##### Massachusetts Health Policy Commission, Boston, MA, USA

###### **Correspondence:** Gabriel Malseptic (gabriel.malseptic@mass.gov)

**Background:** Massachusetts’ community hospitals are increasingly engaging in alternative payment models and developing population health management programs in the context of constrained resources, high public payer mix, and complex patient populations. The Massachusetts’ Health Policy Commission (HPC) created the Community Hospital Acceleration, Revitalization, and Transformation (CHART) program to advance community hospitals’ capabilities in population health management to prepare hospitals to operate more efficiently and effectively under value-based payment models and to reduce unnecessary acute utilization. The second phase of CHART invested nearly $60 million and dedicated technical assistance across 27 community hospitals in Massachusetts. To disseminate lessons from the program, a qualitative analysis was conducted of quarterly reports provided by the hospitals. The goal of the analysis was to identify successes, challenges, and patient and provider experiences. Themes and promising practices emerged, which were refined through feedback from HPC staff, technical experts, and hospital program managers. Based on these empirically-identified practices, the HPC developed the CHART Playbook as a practical guide to some approaches to implementing population health management for patients with complex social, behavioral, and medical needs.

**Methods:** Hospitals submitted monthly and quarterly reports on operational and organizational performance. Inductive qualitative coding was used to identify frequently described challenges. These themes were used to group the solutions and successes reported by teams along with tools and resources used to support their models.

**Findings:** The five domains of the Playbook are: “Identifying Patients”: various strategies and considerations when identifying high-risk/need patients; “Engaging Patients”: guidance on how to engage patients in novel ways, where the healthcare system has typically failed; “Collaborating with Patients”: extensive detail about high-value services and ways of deploying them; “Managing a Team”: guidance for managers of multi-disciplinary, community-based care teams serving vulnerable populations; “Data-driven Operations”: detailed guidance on how to use data to inform operations and communicate with stakeholders.

**Implications for D&I Research:** Population health is a critical component of value-based health care, but granular, operational details on implementation at the provider level (e.g. hospital, accountable care organization) are still developing. As a practical, empirically-developed guide, the CHART Playbook provides important information to frontline clinical teams charged with population health management.

### S99 The Medi-Cal incentives to quit smoking project: Impact of statewide outreach through health channels

#### Elisa Tong^1^, Susan Stewart^1^, Dean Schillinger^2^, Maya Vijayaraghavan^2^, Melanie Dove^1^, Anna Epperson^3^, Cynthia Vela^1^, Susan Kratochvil^4^, Christopher Anderson^5^, Carrie Kirby^5^, Shu-Hong Zhu^5^, Jessica Safier^2^, Gordon Sloss^6^, Neal Kohatsu^6^

##### ^1^University of California Davis, Sacramento, CA, USA; ^2^University of California, San Francisco, San Francisco, CA, USA; ^3^Stanford University, Stanford, CA, USA; ^4^Sacramento, CA, USA; ^5^California Smokers’ Helpline, La Jolla, CA, USA; ^6^California Department of Public Health, Sacramento, CA, USA

###### **Correspondence:** Elisa Tong (ektong@ucdavis.edu)

This abstract is not included here as it has already been published [1].

Reference

[1] Tong et al. The Medi-Cal Incentives to Quit Smoking Project: Impact of Statewide Outreach Through Health Channels. American Journal of Preventive Medicine. 2018;55(6:2):S159-S169.

### S100 Engaging local health departments in peer-to-peer learning strategies to address the opioid crisis

#### Michael Fischer^1^, Bevin Shagoury^1^, Chevelle Glymph^2^, Aleta Christensen^3^

##### ^1^Brigham and Women's Hospital, Boston, MA, USA; ^2^National Association of County and City Health Officials, Washington, DC, USA; ^3^Centers for Disease Control and Prevention, Atlanta, GA, USA

###### **Correspondence:** Michael Fischer (MFISCHER@BWH.HARVARD.EDU)

**Background:** The opioid crisis reflects a wide range of challenges, driven by economic, social, and political factors, local medical practice patterns, and many other considerations. Identifying and implementing effective solutions requires understanding how individual challenges interact and what strategies are most effective in specific situations.

**Methods:** Four pilot sites were selected based on high rates of opioid overdose and high opioid prescribing rates. The pilots were supported by the Centers for Disease Control (CDC), the National Association of County and City Health Officials (NACCHO) and the National Resource Center for Academic Detailing (NaRCAD). The sites included two rural counties and two moderate-sized urban centers (population 100,000-150,000). NaRCAD, CDC, and NACCHO supported public health officials in recruiting health professionals to be trained in NaRCAD’s 2-day academic detailing training course, customized for each site. NaRCAD provided ongoing technical assistance for sites’ implementation of academic detailing opioid safety interventions and designed and implemented an ongoing virtual learning community platform for public health officials and site detailers (https://www.narcad.org/opioid-toolkit.html).

**Findings:** Trainings were conducted at each site in spring 2018; the 24 total trainees came from diverse backgrounds, including pharmacists, nurses, public health officials, and students in the health professions, including pharmacy, dental, and medical school students. Plans for implementing AD varied by site depending on the local health care environment; some sites focused more heavily on appropriate prescribing of opioids by clinicians, while others prioritized increasing treatment of opioid use disorder, especially medication-assisted treatment. As of July 2018, a total of over 100 detailing visits had been conducted, with high variability across sites. One rural and one urban site had over 50 completed visits each, while the other two sites had 10 or fewer. Predictors of success included strong health department leadership, pre-existing connections to health-care providers and networks in the local community, and trainees’ prior familiarity with opioid-related clinical content.

**Implications for D&I Research:** Strategies to address the opioid crisis will require diverse approaches that are customized to the specific needs and challenges of a given region or population. These considerations should be used when developing implementation plans and must be included in the analytic framework for intervention evaluation.

### S101 Economic evaluation of a sun protection promotion program in California elementary schools

#### Richard Meenan^1^, Kim Reynolds^2^, David Buller^3^, Kim Massie^2^, Julia Berteletti^3^, Mary Buller^3^, Jeff Ashley^4^

##### ^1^The Center for Health Research, Kaiser Permanente Northwest, Portland, OR, USA; ^2^Claremont Graduate University School of Community and Global Health, Claremont, CA, USA; ^3^Health Communication, Klein Buendel, Inc., Golden, CO, USA; ^4^Dermatology, Sun Safety for Kids, Burbank, CA, USA

###### **Correspondence:** Richard Meenan (richard.meenan@kpchr.org)

**Background:** The Surgeon General’s 2014 Call to Action to Prevent Skin Cancer emphasized the importance of sun safety for schools; however, limited cost data exist to inform implementation decisions regarding school sun safety practices. The Sun Safe Schools (SSS) program provided technical assistance to California public elementary schools interested in implementing sun safety practices consistent with district board policy for sun safety. Based on a randomized trial of SSS that assessed its effectiveness in promoting implementation, we report preliminary results of an economic evaluation of SSS.

**Methods:** Fifty-eight intervention schools and 60 controls participated. Intervention school principals received regular phone and email contact from trained SSS coaches over 20 months to support implementation of the principals’ selected sun safety practices. Rolling recruitment and intervention occurred over 47 months (2/2014-1/2018). Study outcome data are from a posttest survey of school principals. Intervention delivery costs were virtually all labor (SSS coach and principal time). Implemented practices were organized into 10 categories (e.g., student education, outdoor shade) and micro-costed using a project-developed template. Required school labor and non-labor resources for implementation were estimated for each practice. Three elementary school principal consultants reviewed the template for appropriateness. Data analysis occurred in 2018.

**Findings:** Implemented practices reported at posttest were 157 (mean 2.85, median 2) for controls and 234 (mean 4.42, median 4) for intervention schools. Intervention delivery costs totaled $20.9K ($7.6K for research staff and $13.3K for schools), averaging $0.76 per intervention school student. Costs of implemented practices totaled $620K for intervention schools (mean $11.7K, median $6.0K per school) and $518K for control schools (mean $9.4K, median $3.3K per school). Principals’ beliefs about the importance of sun protection were positively correlated with policy implementation, both in numbers of implemented policies (r=.474, p=.000) and overall dollars invested (r=.326, p=.017).

**Implications for D&I Research:** Preliminary results indicated that a low-cost program of regular phone and email coaching of school administrators can successfully stimulate implementation of sun safety practices in elementary schools at a reasonable cost. Our results can assist administrators with selecting and implementing appropriate sun safety practices in their schools.

### S102 Network modeling to understand predictors of evidence use in policymaking

#### Matthew Weber^1^, Brandon Kramer^2^, Itzhak Yanovitzky^2^

##### ^1^Hubbard School of Journalism and Mass Communication, University of Minnesota, Minneapolis, MN, USA; ^2^Rutgers University, New Brunswick, NJ, USA

###### **Correspondence:** Matthew Weber (msw@umn.edu)

**Background**: This study traces the sharing of research evidence amongst policymakers over a 15-year period in order to understand the characteristics of research evidence sharing. Where evidence is used in policymaking efforts, relatively little is known about why certain research evidence is used more in the long run, nor is the sharing of research evidence among policymakers well understood.

**Methods**: Data analyzed in this study were extracted from a comprehensive set of 190 Congressional hearings concerning federal policies to decrease childhood obesity from 2000-2014. Documents in the dataset were manually coded to extract specific instances of research evidence use (N = 1,809 references). References were coded for scope, type, context, timing, and nature of evidence use. Subsequently, data were transformed into bi-modal social network data, mapping the flow of research evidence (first mode) between members of Congress, researchers, and other policy stakeholders (second mode).

**Findings**: Statistical (exponential random graph) modeling of the network data shows that political branch, political party, and committee membership have a statistically significant impact on evidence exchange pertaining to the formation of childhood obesity policies from 2000-2014. The results show that senators are likely to share research evidence with other senators, but members of the house are not likely to do the same. Moreover, democrats have a statistically significant likelihood of sharing research evidence with other democrats, while republicans do not do the same. Committee membership was also a key determining factor. Finally, previous communication patterns did not affect the likelihood of the sharing of research evidence.

**Implications for D&I Research**: The results of this analysis shed light on the conditions that predict when research evidence is likely to be shared among members of Congress. Moreover, the findings establish the sharing of research evidence is an insular activity; members of Congress turn to other members of Congress, and of their own parties, to learn about new research evidence. Secondary interview data associated with this research points to staffers as a critical conduit for accessing research evidence from outside stakeholders.

### S103 Exploring the determinants of evidence use within clinical network stewardship models: A q methodology study

#### Jade Hart^1^, Margaret Kelaher^1^, Helen Dickinson^2^

##### ^1^University of Melbourne, Victoria, Australia; ^2^University of New South Wales, Australian Capital Territory, Australia

###### **Correspondence:** Jade Hart (j.hart1@student.unimelb.edu.au)

**Background:** The Australian health care system can be characterised as fragmented, with variable integration between health system participants, and a perception that health policies do not always reflect existing evidence. Clinical networks are state government initiatives that pursue improvements by convening multidisciplinary clinicians, consumers, and stakeholders for an areas of focus (e.g. pediatric, cardiovascular, or emergency services), operating as a collective, and enacting system stewardship functions. Evidence use is considered foundational. This raises questions as to what evidence is, and what does evidence use by Australian clinical networks involve when exploring system level rather than clinical practice level change.

**Methods:** A Q methodology study was conducted to identify common viewpoints in relation to the determinants of evidence use among three case study clinical networks. Twenty-six network participants rank-ordered 40 clustered text statements and provided explanatory information on selection. Text statements were informed by the literature and qualitative study involving of 22 clinical network representatives. Key themes included i) network management, ii) network participation, iii) the evidence base, and iv) functional processes. Following piloting, Q-sort administration was conducted via electronically and in-person interview. Data analysis was conducted using PQMethod software (statistical method: principal components analysis with varimax rotation, automatic and manual flagging (P=<0.01), Pearson coefficient, and eigenvalue cut-off >1.0). Factors, or salient viewpoints on the determinants of evidence use within clinical networks were interpreted.

**Findings:** There were four prominent viewpoints of the key determinants of evidence use (providing 45% explanatory variance). Factor 1 focused on evidence stewardship as a form of good governance. Factor 2 considered evidence as a means to influence the suite of disparate and influential organisational leaders. Factor 3 regarded evidence use the means to implement the vision for whole-of-health system reform. Factor 4 placed prominence on the generation and implementation of quality evidence.

**Implications for D&I Research:** Clinical networks are focused on clinical leadership and health system stewardship. Understanding of the key factors which influence the sourcing, filtering, deliberating, and use of evidence, and realist exploration of processes using Q methodology are the unique contributions of this research. Findings provide a basis for integrated theory building.

### S104 What evidence counts in health policymaking, why, and when?

#### Itzhak Yanovitzky^1^, Matthew Weber^2^

##### ^1^Rutgers University, New Brunswick, NJ, USA; ^2^Hubbard School of Journalism and Mass Communication, University of Minnesota, Minneapolis, MN, USA

###### **Correspondence:** Itzhak Yanovitzky (itzhak@rutgers.edu)

**Background:** Use of research evidence in public policymaking processes is inherently political and strategic, with evidence being used to influence others to accept or reject policies. Accordingly, what evidence is used, when, and for what purpose is determined by the persuasive value policymakers place on different forms of evidence as well as the circumstances of the policymaking process. This study delineates practices of evidence use in policymaking by systematically examining how policymakers communicate with and about evidence.

**Methods:** Data were extracted from a comprehensive set of 190 Congressional hearings concerning federal policies to decrease childhood obesity from 2000-2014. Each document was scanned manually to extract arguments that reference either research or non-research evidence (N = 3,215 arguments). A team of trained coders analyzed the arguments for variables measuring the scope, type, context, timing, and nature (i.e., purpose and persuasive strategy) of evidence use. Content analysis findings were then corroborated and augmented through key-informant interviews (N = 10) with Congressional staffers involved in the crafting of federal legislation.

**Findings:** Statistical facts, particularly those obtained from reputable sources, are most frequently included (85% of all arguments) as authoritative evidence in arguments. However, anecdotal evidence (testimonials, personal experiences) was also commonly presented (30% of all arguments). Evidence was frequently used to establish the importance of the problem (31% of all arguments), speak to its potential causes (17%), or make the case for a particular policy solution (19%). There were negligible differences in evidence use across types of hearings (appropriation, investigative, oversight, etc.) as well as across specific policies (school nutrition mandates vs. regulating food advertising and marketing of sugary snacks and drinks). Political use of evidence increased since 2009. Use of evidence to accelerate the enactment of policies increased with the opening of policy windows (i.e., change in administration or composition of Congress).

**Implications for D&I Research:** More complete understanding of policymakers’ strategic use of research evidence – particularly, what, when, and for what purpose it is used – can more productively inform efforts to embed research evidence in public policymaking processes than are normative expectations about hoe policymakers should use evidence.

## Models, Measures, and Methods

### S105 Assembling consensus through clinical workflows: Using rapid ethnographic assessment for successful external facilitation

#### Heather Reisinger (heather.reisinger@va.gov)

##### Carver College of Medicine, University of Iowa, Iowa City, IA, USA

**Background:** Rapid Ethnographic Assessment (REA) grew out of anthropologists’ engagement with international development programs. The goal of REA was to rapidly assess cultural contexts in an effort to improve spread of successful international development projects. More recently, anthropologists have begun to introduce this implementation methodology into the field of health care and public health. The Telehealth Outreach for PTSD (TOP) is an intervention to improve rural veterans’ access to evidence-based PTSD treatment. This project employed REA to inform the external facilitation process and improve TOP implementation.

**Methods:** The external facilitation team conducted site visits at three VA healthcare systems that did not meet intervention benchmarks post-implementation. REA methodology included site visits and team-based semi-structured interviews with multiple stakeholders at each site. Interviews were conducted with local TOP leads and care managers (n=6); leadership (n=9), psychologists and psychiatrists (n=6) at VA medical centers; and mental health care providers (n=7) at outpatient clinics where veterans receive primary care. The external facilitators then used the interview data to develop clinical workflow maps as visualizations of various perspectives on the intervention. The baseline clinical workflow maps were then shared with the local intervention team and revisions were made to improve the implementation process. Revisions continued to be made to the workflow map until consensus was reached locally.

**Findings:** REA provided a framework to rapidly gather information to assemble a shared clinical workflow. Through REA, the external facilitation team quickly learned about the organizational context and barriers and facilitators local teams faced in implementing the intervention. Stakeholders appreciated sharing their perspectives on the intervention and participating in the process of revising the clinical workflow. Continued external facilitation was necessary to assist the local team in carrying out the new clinical workflow and overcome inertia to return to the original clinical process. All three sites have made substantial progress toward improving uptake of the intervention.

**Implications for D&I Research:** REA is a flexible methodology that can be paired with a variety of implementation strategies to improve intervention uptake within a short timeframe, while remaining close to the ground-level perspectives of practitioners.

### S106 Implementation science, context and ethnography: Insights from complex interventions in clinical pharmacy practice

#### Megan McCullough (megan.mccullough@va.gov)

##### Center for Healthcare Organization and Implementation Research, Veterans Health Administration, Bedford, MA, USA

**Background:** Context is dynamic, complex, and frequently a confounder in the implementation of evidence-based interventions. Linear and mechanistic models of implementation have limitations in adequately depicting complex social systems. Conceptually and methodologically, ethnography offers a systems-informed complexity approach to study how and why an intervention succeeds or fails and under what circumstances. Focusing on two clinical pharmacy interventions in the Department of Veterans Affairs (VA), this paper illustrates the methodological and conceptual contributions ethnography makes to implementation science.

**Methods:** Ethnography provides detailed descriptions of everyday life and practice, sometimes called “thick description” (Geertz 1973). Direct observation (DO) and ethnographic interviews were conducted and analyzed in two clinical pharmacy interventions: 1) a pharmacy-focused anticoagulation improvement initiative across 8 medical centers; and 2) implementation of 180 Clinical Pharmacy Specialists (CPS) across approximately 50 sites to improve access to care in mental health and primary care. The contributions of both studies to understanding the dynamism inherent in uptake, spread and impact will be presented.

**Findings:** In study 1, interviews and DO identified how key contextual elements (e.g. leadership, communication, teamwork, understanding of new evidence, etc.) interacted with each other in contributing to uptake of the evidence-based practice, often yielding results that could not be predicted by looking at just one of these elements alone. In study 2, ethnographic interviewing of CPS and clinical team members was used to evaluate the integration of CPSs in teams and the quality of that integration. Ethnographic findings about the quality and dimensions of integration added a more comprehensive understanding of integration than survey data alone could provide. Ethnography provided conceptual and methodological ways of studying the complex interactions among social actors that produce systems-level behaviors.

**Implications for D&I Research:** Ethnographic approaches are ideal for studying the dynamic properties of healthcare systems and the varying contextual characteristics that are deeply enmeshed in social practices. These approaches assist in understanding and evaluating the impact of the multiple forces, variables and influences that need to be factored into any change process. Ethnography helps us “see” the uncertainty and flexibility that are the normal properties of multivalent, intricate systems.

### S107 Using “periodic reflections” to evaluate a facilitation strategy to implement video telehealth to home for rural veterans

#### Lindsey Martin (Lindsey.Martin3@va.gov)

##### Center for Innovations in Quality, Effectiveness & Safety, Veterans Health Administration, Houston, TX, USA

**Background:** Implementing video telehealth to home (VTH) for the delivery of mental health care to rural Veterans is challenging given the diversity of settings within the Veterans Health Administration. Implementation facilitation (IF) is an evidence-based implementation strategy that is adaptable, and uses intensive stakeholder engagement, training and support as well as formative evaluation to promote adoption. To evaluate the effectiveness of IF for VTH adoption, ethnographically-informed “periodic reflections” are being used to document the subjective, nuanced and non-quantifiable aspects of this strategy while implementation occurs.

**Methods:** VTH external facilitators (EFs) complete 30-minute dyadic periodic reflections one to four times per month with the project’s anthropologist. Reflections are largely unstructured discussions as EFs recount all recent facilitation activities (e.g. phone meetings with champions), regardless of the temporal order of events. Reflections are driven by the EFs with the methodologist only using probes and follow-up questions for clarification purposes. The dyadic format encourages EFs to interact with one another, producing rich conversation. Detailed notes are taken and iteratively analyzed to inform internal process improvement efforts, and identify IF adaptations and core themes across sites.

**Findings:** EFs completed 13 periodic reflections between February and July 2018, all but one in a dyadic format. While an IF strategy typically engages stakeholders from the top-down, reflections captured how EFs were adapting their approach to a hybrid top-down and bottom-up strategy to build relationships with providers and leadership to maximize adoption. Reflections also reveal how the amount or dose of facilitation is context dependent; facilitation dose is lighter in early adopter versus late adopter sites that require greater effort by EFs to foster buy-in. Additionally, periodic reflections are more than a purely evaluative method. The actual reflection process drove implementation forward as EFs identified key action items during their discussions.

**Implications for D&I Research:** Significant gaps remain in the literature regarding fidelity to and adaptation of implementation strategies. Periodic reflections allowed documentation of “how” and “why” an IF strategy is adapted, allowing EFs to improve its effectiveness. This pragmatic, ethnographic approach to formative evaluation furthers our knowledge regarding the use and tailoring of implementation strategies in real-time.

### S108 The cognitive walk-though for implementation strategies (CWIS): A pragmatic methodology for assessing strategy usability

#### Aaron Lyon^1^, Jessica Coifman^1^, Heather Cook^1^, Freda Liu^1^, Kristy Ludwig^1^, Shannon Dorsey^1^, Kelly Koerner^2^, Sean Munson^1^, Elizabeth McCauley^1^

##### ^1^University of Washington, Seattle, WA, USA; ^2^Evidence-Based Practice Institute, Seattle, WA, USA

###### **Correspondence:** Aaron Lyon (lyona@uw.edu)

**Background:** Implementation strategies vary widely, but most are inherently complex social interventions. Unfortunately, excessive complexity can hinder usability, making some strategies difficult to apply. Pragmatic methods are needed to evaluate usability in context. Cognitive walk-throughs are an efficient method from the field of user-centered design that assess usability by activating and recording users’ internal cognitive models for specified tasks. This session will describe a novel walk-through method for assessing implementation strategy usability and its application to post-training clinical consultation procedures for supporting measurement-based care (MBC) in behavioral health.

**Methods:** The Cognitive Walk-through for Implementation Strategies (CWIS) is a streamlined, group-based procedure for assessing strategy usability. CWIS includes six steps: (1) determine preconditions, (2) conduct a task analysis, (3) prioritize tasks, (4) convert tasks to scenarios, (5) group testing, and (6) problem classification/prioritization. Twenty-four unique tasks were identified in the consultation protocol, prioritized using expert ratings of importance and error likelihood, and converted into six scenarios. An administrator conducted two CWIS testing sessions (N=10 clinicians) and walked-through 11 unique tasks. During testing, clinicians were introduced to each task, rated their anticipated likelihood of success, provided open-ended rating justifications, and completed the Implementation Strategy Usability Scale (ISUS). Usability issues were identified using qualitative content analysis.

**Findings:** Prioritized tasks included, but were not limited to: succinct case presentations; revising outcome monitoring plans based on feedback; and navigating an online message board during consultation. Across tasks, anticipated success ratings varied from 10-90% of participants indicating a high level of confidence. A task linking client intervention goals to an outcome monitoring plan received the lowest ratings. Mean ISUS score was 71.3, indicating acceptable usability, but also room for improvement. Testing indicated that the structure, novelty, and collaborative nature of the consultation protocol facilitated usability. Usability issues included potential misalignment between consultation and clinical service timelines as well as the need for tools to support real-time decision-making during consultation.

**Implications for D&I Research:** Based on the CWIS results, the MBC consultation procedures were revised to improve usability. In addition to driving implementation strategy redesign, CWIS information may be used to facilitate selection or local tailoring of strategies to meet contextual needs.

### S109 Pinpointing the specific implementation strategies that matter most for increasing HCV treatment: An applied use of comparative configurational methods

#### Vera Yakovchenko^1^, Edward Miech^2,3^, Rachel Gonzalez^4^, Angela Park^5^, Maggie Chartier^6^, David Ross^6^, Matthew Chinman^7^, Timothy Morgan^8^, Shari Rogal^9^

##### ^1^BridgeQUERI, CHOIR, Veterans Health Administration, Bedford, MA, USA; ^2^Indiana University Center for Health Services and Outcomes Research, Indianapolis, IN, USA; ^3^VA PRIS-M QUERI, Veterans Health Administration, Indianapolis, IN, USA; ^4^VA Long Beach, Veterans Health Administration, Long Beach, CA, USA; ^5^OSI|VERC, Veterans Health Administration, Boston, MA, USA; ^6^HIV, Hepatitis, and Public Health Pathogens Programs, Veterans Healthcare Administration, Washington, DC, USA; ^7^RAND Corporation, Pittsburgh, PA, USA; ^8^VA Long Beach Healthcare System, Veterans Health Administration, Long Beach, CA, USA; ^9^VA Pittsburgh Healthcare System, Veterans Health Administration, Pittsburgh, PA, USA

###### **Correspondence:** Vera Yakovchenko (vera.yakovchenko@va.gov)

**Background**: The Veterans Health Administration (VHA) cares for more patients with hepatitis C virus (HCV) than any other healthcare system in the US. In anticipation of interferon-free HCV treatments, VHA developed the HCV Innovation Team (HIT) Collaborative in 2015. Within the HIT Collaborative providers joined regional teams and conducted HCV quality improvement activities. We applied comparative configurational methods (CCMs) to identify specific implementation strategies associated with higher HCV treatment rates.

**Methods**: We operationalized quality improvement activities as implementation strategies per the Expert Recommendations for Implementing Change (ERIC) project, which defined 73 different strategies meant to promote the uptake of evidence-based practices. We conducted an electronic survey of HCV providers at 130 different sites to assess each facility’s use of each of the 73 implementation strategies to promote HCV treatment starts. We used configurational comparative methods (CCMs), a mathematical approach based on Boolean algebra and set theory, to identify the specific combinations of implementation strategies associated with higher HCV treatment starts. These analyses were conducted using the “cna” package for R.

**Findings**: Eighty (62%) of 130 sites responded to the survey and reported using a mean of 25 implementation strategies per site in 2015. The CCMs analyses identified three distinct “high-uptake paths” involving 6 implementation strategies that collectively accounted for 65% of the sites with higher HCV treatment starts with 100% consistency. One path featured a single strategy (“local technical assistance”); another, a combination of two strategies (“foster collaborative learning environment” AND “recruit, designate, train leaders”); and the third, a combination of three strategies (“activate patients” AND “create new clinical teams” AND “share the knowledge gained from quality improvement efforts with other sites”). The presence of any one of these three paths was sufficient for higher HCV treatment starts.

**Implications for D&I Research**: Through applying CCMs to a national sample of VHA sites, we pinpointed specific combinations of implementation strategies associated with increased HCV treatment. Starting with an initial set of 73 different implementation strategies, we identified 3 high-uptake paths that involved only 6 implementation strategies. CCMs provide a mathematical method for identifying specific combinations of implementation strategies that matter to implementation outcomes.

### S110 Coincidence analysis: A methodology to identify contextual conditions influencing implementation across multiple settings

#### Deborah Cragun^1^, Alanna Kulchak Rahm^2^

##### ^1^University of South Florida, Tampa, FL, USA; ^2^Geisinger, Danville, PA, USA

###### **Correspondence:** Deborah Cragun (dcragun@health.usf.edu)

**Background:** Dissemination and implementation (D&I) research is often challenging due to small to moderate sample sizes, multiple data sources, and desire to uncover complex and diverse ways in which contextual factors combine to impede or facilitate implementation and sustainability of evidence based practices (EBPs). We describe the value of Configurational Comparative Methods (CCMs) in D&I research with a focus on the utility of a new type of CCM, called coincidence analysis (CNA).

**Methods:** Based on the regularity theory of causation and Boolean algebra, CCMs take a comparative and iterative analytic approach whereby cases (e.g., individuals, organizations, geographic regions) are systematically compared across a set of conditions (e.g., contextual factors or implementation strategies employed) to identify which conditions or combinations thereof consistently make a difference in whether or not an outcome was observed (e.g., successful implementation or maintenance of an EBP). CCMs can analyze data from interviews, focus groups, surveys, and other sources using the following steps: 1) define, code and calibrate data for each condition and outcome in order to form a data matrix, 2) utilize specialized software to create a truth table showing configurations of conditions and outcomes, 3) review which cases fall within each configuration and determine whether additional data are needed, 4) run the computer algorithm to reveal minimally sufficient and necessary conditions for the outcome.

**Findings:** Examples will demonstrate the multi-step process of conducting CNA and illustrate how it can reveal the existence of more than one “recipe” to achieve an outcome. We will also discuss advantages of CNA over other CCMs, including CNA’s ability to identify underlying causal chains. For example, multiple contextual conditions can influence which implementation strategies are used; and certain strategies may, in turn, lead to successful implementation or maintenance in different contexts.

**Implications for D&I Research:** We advance implementation research by presenting a pragmatic method that allows researchers to combine qualitative and quantitative data obtained in a mixed methods study and to account for many of the complexities and challenges inherent in D&I research.

### S111 The lightning report: What a new methodological approach for rapid qualitative synthesis can tell us about prospective evaluation of dynamic system implementations

#### Cati Brown-Johnson, Nadia Safaeinili, Dani Zionts, Laura Holdsworth, Marcy Winget

##### Stanford University School of Medicine, Stanford, CA, USA

###### **Correspondence:** Cati Brown-Johnson (catibj@stanford.edu)

**Background:** Prospective evaluation of dynamic system implementation calls for immediate, actionable insights, yet standard qualitative methods are long-term. A rapidly evolving healthcare implementation requires methods and tools to facilitate prompt communication with stakeholders while maintaining methodological rigor. The Lightning Report addresses these gaps with a methodological approach and flexible framework that innovates on debriefing techniques from manufacturing. This purposeful qualitative data collection and synthesis process enables rapid feedback to healthcare partners.

**Methods:** The Lightning Report method includes: Pre-planning with evaluation partners. Revisit research questions, discuss emerging areas of interest, and tailor data collection tools; Rapid synthesis. Structured research notes surface themes and unexpected findings. Researchers discuss notes/memos, and synthesize findings using Plus/Delta debriefing, adapted from Lean pedagogies; Lightning Report creation. Components include executive summary, status of data collection, and findings that reflect Plus/Delta: what is going well with implementation, improvement opportunities and what needs to change, and suggested actions (“Insights”). Refined with stakeholder input. To understand which D&I constructs are best reflected using this method, we employed the Consolidated Framework for Implementation Science to code Lightning Reports and examined themes within CFIR constructs.

**Findings:** Of 245 implementation science-relevant excerpts in 13 reports, primary D&I constructs included Patient needs/resources (34 excerpts, care coordination and patient relationship themes), and Networks/communication (27 excerpts, communication and team cohesion/lack themes). Facilitators clustered in the primary constructs as well as Adaptability and Available Resources; themes included intervention adaptation by front-line staff, training, space, and staff experience. Barriers also clustered in primary constructs and Compatibility, Access to knowledge/information, and Complexity; themes centered on role definition (lack of, inappropriateness tasks for non-clinical staff, miscommunication/misunderstanding of new roles).

**Implications for D&I Research:** A Lightning Report approach that incorporates structured debriefing and stakeholder input may emphasize dynamic system implementation themes related to patients, relationships, and communication. This in-process implementation evaluation method may highlight logistical facilitators; barriers surfaced may indicate the need for greater intervention specification (role definition). Bridging the chasm between data collection and full data analysis/results publication, the Lightning Report facilitates rich D&I insights, can be rapidly developed from data to deliverable, and is highly valued by evaluation partners engaged in implementation, and generates stakeholder trust.

### S112 Qualitative research in implementation science (the QUALRIS project): Meeting the challenges

#### Suzanne Heurtin-Roberts^1^, Deborah Cohen^2^, Benjamin Crabtree^3^, Laura Damschroder^4^, Deborah Padgett^5^, Borsika Rabin^6^

##### ^1^National Cancer Institute, National Institutes of Health, Rockville, MD, USA; ^2^Oregon Health & Science University, Portland, OR, USA; ^3^Rutgers Robert Wood Johnson Medical School, New Brunswick, NJ, USA; ^4^VA Ann Arbor Center for Clinical Management Research, Ann Arbor, MI, USA; ^5^Silver School of Social Work, New York University, New York, NY, USA; ^6^Family Medicine and Public Health, University of California, San Diego, La Jolla, CA, USA

###### **Correspondence:** Suzanne Heurtin-Roberts (sheurtin@mail.nih.gov)

**Background:** Implementation science (IS) makes extensive use of qualitative methods, usually in mixed methods. Yet certain features of implementation science pose specific challenges to qualitative methods’ rigor and flexibility. Limited guidance has been available to meet these challenges while optimizing the methods’ use, threatening the scientific integrity and practical utility of implementation science. This presentation reports the findings of the *Qualitative Research in Implementation Science* (QUALRIS) project, whose interim findings were reported at this conference in 2016.

**Methods:** Beginning in 2015, the National Cancer Institute’s Implementation Science Team convened the QUALRIS Group, composed of experts in implementation science and/or qualitative research, to develop guidance for the improved use of qualitative methods in implementation science, and to recommend future efforts to improve rigor and utility. The group consulted best practices literature in qualitative methods and members’ own extensive experience in using these methods in implementation science. By means of various telecommunications media and an iterative consensus process, they drafted pertinent methodological guidance and recommendations for needed innovation. These findings are contained in the NCI white paper “Qualitative Methods in Implementation Science” and reported in this presentation.

**Findings:** Intrinsically change oriented, IS research is rapid and sensitive to change in complex contexts and dynamic processes. The group found that qualitative methods in IS are challenged by features such as time limitations, limited engagement with stakeholders, a priori research questions, and team science. The QUALRIS group drafted guidance in areas including the following: 1) the essential relationship of theory, question, and method 2) imperatives for qualitative sampling 3) timing and methods of qualitative data collection 4 rationale and documentation for instrumentation, data collection, and analytic processes 5) documentation of strategies to achieve and maintain rigor 6) improved presentation of qualitative findings in IS publications. The need was expressed for innovations in methods of meta-synthesis and cross context comparison

**Implications for D&I Research:** QUALRIS guidance and recommendations offer a resource for consistent, rigorous standards for using qualitative methods in IS. The QUALRIS group calls for further methodological research and innovation to advance the rigor and applicability of implementation science

### S113 Participatory modelling to inform health and social service design and implementation: Illustrating the approach using discrete event simulation modeling to decrease the burden of mental illness in jails

#### Kristen Hassmiller Lich^1^, Elizabeth Sinclair^2^, Sidd Nambiar^3^

##### ^1^University of North Carolina at Chapel Hill, Chapel Hill, NC, USA; ^2^Treatment Advocacy Center, Arlington, VA, USA; ^3^North Carolina State University, Raleigh, NC, USA

###### **Correspondence:** Kristen Hassmiller Lich (klich@unc.edu)

**Background:** Every day in the US, approximately 5,000 adults in psychiatric crisis are arrested – for reasons often attributable to their illness. Alongside the high personal cost, these arrests contribute to the top four challenges among county jails -- the high number of individuals in jail with mental illness, burden of coordinating mental health treatment in jail, and high/increasing jail costs and population sizes. Exacerbating challenges, before individuals in psychiatric crisis can be tried in court, their competence must be restored. Limited inpatient capacity in nearly every state is resulting in an average wait of 30 days, though observed wait times in excess of 6-9 months are documented. This has been a hard problem to address. Inpatient psychiatric beds, regardless of criminal involvement, are in short supply. And it is expensive to increase capacity. Other potential solutions require resource sharing across sectors. Many states are slow to address this problem, despite substantial negative consequences that in some cases include expensive lawsuits and penalties. Fortunately, simulation model analysis suggests that small changes in several places across systems can dramatically reduce wait times. However, due to the dynamic complexity of this problem, our results are not intuitive – threatening their use.

**Methods:** To address this challenge, we are engaging diverse stakeholder groups at state and regional levels with simulation models to help them understand and believe simulation results. We have created a user-friendly version of the model that, once understood, can be used within a planning meeting to advance discussion, strategic thinking, and consensus around action.

**Findings:** In this presentation, we will: (1) define “dynamic complexity,” the characteristic to which we attribute the need for a decision support model; (2) demonstrate how we grow confidence in the model, allowing you to interact with it; (3) present results on comparative impact of evidence-informed actions; and (4) share lessons learned about how best to engage stakeholders with the model as they use evidence to get decision makers to locally-appropriate action.

**Implications for D&I Research:** Others wrestling with action targeting dynamically complex challenges will learn how we describe dynamic complexity, and systematically and collaboratively study assess intervention approaches.

### S114 Participatory system dynamics for high quality VA addiction and mental health care

#### Lindsey Zimmerman^1,2^, David Lounsbury^3^, Craig Rosen^1,4^, Rachel Kimerling^1^, Andrew Holbrook^5^, Savet Hong^1^, Jane Branscomb^6^, Debra Kibbe^6^, Stacey Park^1^, Ren Kramer^8^, DC Barlow^8^, Swapandeep Mushiana^9^, Kathryn Azevedo^8^, Joyce Yang^9^, Jodie Trafton^10^, Steven Lindley^11^, Tom Rust^5^

##### ^1^National Center for PTSD, Veterans Health Administration, Menlo Park, CA, USA; ^2^University of Washington School of Medicine, Seattle, WA, USA; ^3^Albert Einstein College of Medicine of Yeshiva University, Bronx, NY, USA; ^4^Stanford University School of Medicine, Menlo Park, CA, USA; ^5^Office of Strategic Integration, Veterans Health Administration, Boston, MA, USA; ^6^Georgia Health Policy Center, Atlanta, GA, USA; ^7^University of San Francisco, San Francisco, CA, USA; ^8^VA Palo Alto Health Care System, Veterans Health Administration, Menlo Park, CA, USA; ^9^VA Palo Alto Health Care System, Veterans Health Administration, Palo Alto, CA, USA; ^10^VA Palo Alto Health Care System, Veterans Health Administration, Menlo Park, CA, USA; ^11^Veterans Affairs Palo Alto Healthcare System, Veterans Health Administration, Menlo Park, CA, USA

###### **Correspondence:** Lindsey Zimmerman (Lindsey.Zimmerman@va.gov)

**Background:** Our team enlisted participatory system dynamics (PSD) to improve the reach of evidence-based psychotherapy and pharmacotherapy in the VA outpatient mental health system. In partnership with patients, providers and policy makers, we developed system dynamics models of mental health care coordination, medication management, and psychotherapy.

**Methods:** We used a mixed-methods approach to define our system problem, using exercises drawn from the system dynamics research literature. We concluded that we reached a point of “saturation,” where no new major system dynamics were identified within our model boundary of local team care decisions. We completed several iterations of structural-behavioral validity testing to establish that the causal-descriptive mathematical formulation of our models was valid and adequate for informing stakeholders’ decisions. Our sources of data were existing health system data captured during routine care, including international classification of disorders to identify patient cohorts, and common procedural terminology to define the visits between patients and providers. Using the modeling resources we developed, we facilitated frontline, multidisciplinary addiction and mental teams in a more standardized mutual learning program entitled, *Modeling to Learn.* Finally, we used a pre/post, quasi-experimental design to assess the effectiveness of the PSD methods for improving EBP reach.

**Findings:** Our qualitative analyses showed that teams found PSD acceptable, feasible and useful for enacting change. In preliminary statistical process control analyses, we found that our two participating clinics saw a greater than 3 standard deviation improvement in the reach of evidence-based psychotherapy as compared to their 12-month baseline, and that these improvements were sustained for eight and twelve-months, respectively.

**Implications for D&I Research**: As our project continued, a wider array of VA stakeholders engaged in *Modeling to Learn*, which led to the development of national participatory modeling quality improvement training program. Our *Modeling to Learn* core principles are likely useful for other implementation researchers doing similar work. These principles are: 1) transparency across stakeholders in understanding health system data, standards and the system causes of implementation problems, 2) scalable processes that enlist local data and local decision-making to tailor solutions to local capacities and constraints, and 3) ongoing mutual learning to guide iterative improvement.

### S115 Modelling with stakeholders to inform health and social service design and implementation: A systematic scoping review of descriptions and empirical research

#### Mark Pearson^1^, Sean Manzi^2^, Laura Pickup^2^, Amanda Wanner^3^, Andy Salmon^2^, Ken Stein^2^, Iain Lang^2^

##### ^1^Hull York Medical School, University of Hull, Hull, United Kingdom; ^2^University of Exeter Medical School, University of Exeter, Exeter, United Kingdom; ^3^University of Plymouth, Plymouth, United Kingdom

###### **Correspondence:** Mark Pearson (mark.pearson@hyms.ac.uk)

**Background:** Approaches to implementing evidence-based practice using modelling techniques with strong participatory elements are becoming more widespread*.* Participatory modelling engages people (e.g. service users, health and social care professionals, managers) in the diagnostic (problem-framing and structuring), prognostic (model-building and exploration of scenarios), and motivational (organization of collective action) stages of modelling (Black 2013). Whilst model development itself is a well-documented method(s), the social processes which enable participatory modelling to be conducted are not well-understood. Modellers learn facilitation skills ‘on the job’ rather than by design.

**Methods:** Research questions: 1.What descriptions exist of participatory modelling processes? 2.What empirical research (evaluation) on participatory modelling has been conducted? A structured search strategy combining free text and indexing terms was run in major databases spanning the area of health services and operations research. Backwards and forwards citation chaining of the final included articles. Inclusion criteria: descriptive or explanatory studies reporting modelling (e.g. System Dynamics, Discrete Event Simulation, Agent Based Modelling) where users were involved. 25% of screening decisions were checked by a second reviewer. Types of participation (‘structured’, ‘involving’) were classified using the de Gooyert et al. (2017) criteria. Modelling and study characteristics (in particular, depth and robustness of evaluation) were extracted and narratively summarized.

**Findings:** 273 studies were included, covering the sectors of healthcare, social care, public health, and community development. Studies were predominantly conducted in North America, Western Europe, and Australasia. A minority of modelling processes used a fully-developed participatory approach. Our emerging findings suggest that although a number of studies acknowledged the important role played by group management processes in participatory modelling, there was little empirical exploration or evaluation of these processes.

**Implications for D&I Research:** Our review provides a map of knowledge that differentiates between participatory modelling (which problematizes service issues and manages conflict between diverse stakeholders) and more conventional forms of modelling (where the perceptions and goals of a limited group of stakeholders are used to ‘optimize’ between different scenarios). This map will enable future evaluations to focus on explaining how participatory modelling can function better as a tool within Implementation Science, and how capacity in participatory modelling expertise can be developed.

### S116 The clinical sustainability assessment tool (CSAT): Assessing sustainability in clinical medicine settings

#### Douglas Luke (dluke@wustl.edu)

##### Washington University in St. Louis, Saint Louis, MO, USA

**Background:** Although the concepts of sustainability, sustainment, and maintenance have started receiving more attention by implementation scientists, only a few comprehensive conceptual frameworks have been developed, and even fewer validated assessment tools exist. Prior to this work, there was no work conducted in clinical medicine to understand sustainability and the many factors that contribute to sustainment of practices in this unique context. This project resulted in a new clinical sustainability assessment tool that is being pilot-tested in a number of inpatient and outpatient medical settings.

**Methods:** We have developed a previous sustainability measurement instrument, the Program Sustainability Assessment Tool (PSAT). It has been used successfully to measure sustainability by over 3,000 public health, social service, and educational programs around the world. Using the PSAT as a template, we created a new measure focused on the specific challenges of sustaining evidence-based practices in clinical settings. Concept mapping was utilized with national experts in implementation research and clinical medicine to create the new measure. After initial measurement development, a pilot-study was conducted (n=52) with practitioners in clinical medicine across multiple subspecialties to ascertain instrument usability and reliability. We continue to collect pilot data and anticipate having data available for presentation by October 2018.

**Findings:** This measure, aiming to understand sustainability in clinical medicine, contains 49 statements nested in seven different domains. The domains considered important for clinical settings are: engaged staff and leadership, outcomes and effectiveness, engaged stakeholders, workflow integration, monitoring and evaluation, organizational context and capacity, and planning and implementation.

**Implications for D&I Research:** This tool allows for better understanding of what factors create the context within which clinical practices are able to sustain over time. While much work has been conducted in the fields of dissemination and implementation science, there has been less focus on the areas of sustainability. This work pushes implementation science more firmly into the clinical setting while increasing the field’s understanding of assessing and promoting sustainable implementation.

### S117 Operationalizing sustainment activities for two evidence-based practices using the stages of implementation completion (SIC)

#### Lisa Saldana (lisas@oslc.org)

##### Oregon Social Learning Center, Eugene, OR, USA

**Background:** Sustainment is one of the least understood phases of implementation, but one of the most important for achieving public health impact. It defines the period beyond program adoption and development of competency in delivering the model. Although the final phase in the implementation process, long-term sustainment is ongoing, dynamic, and recursive. The Stages of Implementation Completion (SIC), is a psychometrically sound measure of implementation processes and milestones. Previous research has shown the instrument’s ability to predict program start-up and achievement of competency in program delivery. To date, the SIC has ended with achievement of this milestone. This presentation will describe our process for extending the SIC into the Sustainment Phase.

**Methods:** Two evidence-based practices (EBPs) for the treatment of adolescent externalizing behaviors and prevention of substance abuse were recruited for participation—Multidimensional Family Therapy (MDFT) and Treatment Foster Care Oregon (TFCO). Agencies that were active or began implementing after January 1, 2013 for whom pre-sustainment SIC data was collected (MDFT = 54; TFCO = 92), are included. Mixed methods are being utilized to operationalize the Sustainment Phase for addition to the existing pre-sustainment SIC. Qualitative interviews with MDFT (n = 10) and TFCO sustaining sites’ leadership (n = 10), along with quantitative and qualitative data collected from the EBP purveyors were used to create clearly defined sustainment items and associated codes. The resulting SIC Sustainment Phase activities will be used to monitor and track newly adopting real-world MDFT and TFCO programs. Data collection is underway and will be available for presentation by November 1, 2018.

**Findings:** As defined by the SIC, 14 MDFT and 8 TFCO sites have achieved sustainment since 2013, with others in progress or discontinuing. Using these retrospective sites, presented findings will include accuracy of assessing activities necessary for sustainment, preliminary psychometric properties of the Sustainment Phase, and predictive models from pre-sustainment SIC scores to key sustainment milestones.

**Implications for D&I Research:** Measuring the sustainment of EBP implementation is necessary for both research and real-world advances. As the D&I field increases emphasis on development and evaluation of strategies for sustainable implementation, standardized methods of measuring the process of achieving this outcome are critical.

### S118 Correlates of sustainment of prevention programs and initiatives in clinical and community settings

#### Lawrence Palinkas (palinkas@usc.edu)

##### University of Southern California, Los Angeles, CA, USA

**Background:** Sustainment of prevention efforts directed at substance use and mental health problems is one of the greatest, yet least understood challenges of implementation science. Efforts to assess sustainment of prevention programs and initiatives in behavioral health service settings may be influenced by the setting (clinic versus community) and by the scope (broad initiatives versus specific evidence-based interventions). At issue is how generalizable or context specific sustainment measure can and should be.

**Methods:** We administered a draft Sustainment Measurement System, a 50-item instrument grouped into 7 categories of predictors/requirements (funding and financial support, responsiveness to community needs and values, coalitions, partnerships and networks, infrastructure and capacity, leadership, monitoring and evaluation, and positive outcomes), to 84 representatives of 75 grantees funded by 3 SAMHSA programs (Sober Truth on Preventing Underage Drinking [STOP-Act] (n = 42), Garrett Lee Smith Suicide Prevention Program (n=34), and Prevention Practices in Schools (n=8). Sustainability was assessed on the basis of three specific outcomes (continuing to operate as described in the original grant, continuing to deliver services to intended population, continuing to deliver evidence-based services) as well as an average of mean scores on these three outcomes. Outcomes and their associations with predictors were compared by setting and scope.

**Findings:** Overall, sustainment was significantly associated with support by federal, state and local government funding, responsiveness to community needs and values, coalitions, partnerships and networks, infrastructure and capacity to support sustainment, monitoring and evaluation, and positive outcomes. Leadership was significantly associated with sustainment only in PPS-funded programs. Compared to a specific EBP, broader prevention initiatives reported higher funding from government sources, coalition, partnerships and networks, infrastructure and capacity to support sustainment, leadership, and monitoring and evaluation. No differences by program setting were observed. The association between sustainment and responsiveness to community needs and values was statistically significant in broader initiatives and clinic-based programs but not in the PPS-funded EBPs and community-based initiatives, respectively.

**Implications for D&I Research:** The Sustainment Measurement System can identify and support both the *unique* requirements for improving sustainment for each program as well as for developing a *generalizable* framework comprised of core components of sustainment across diverse prevention approaches.

### S119 Conducting formative evaluation, studying implementation facilitation, & documenting model adaptation “over the shoulders” of facilitators

#### Karen Drummond^1^, Karen Oliver^2^, Eva Woodward^1^, JoAnn Kirchner^3^, Richard Owen^1^

##### ^1^Central Arkansas Veterans Healthcare System, Veterans Health Administration, Little Rock, AR, USA; ^2^William S. Middleton Memorial Veterans Hospital, Veterans Health Administration, Madison, WI, USA; ^3^Central Arkansas Veterans Healthcare System (North Little Rock), Veterans Health Administration, North Little Rock, AR, USA

###### **Correspondence:** Karen Drummond (KLDrummond@uams.edu)

**Background:** As part of a Hybrid Type 2 pragmatic effectiveness-implementation trial of Tele-PCMHI in rural community clinics in the Veterans Administration (VA), we are using weekly debriefings with our study team implementation facilitators to meet four goals: 1) to conduct a formative evaluation of the pre-implementation and implementation phases at each site; 2) to study implementation facilitation with a dual-facilitator strategy; 3) to study the process of a junior facilitator learning facilitation under senior facilitator mentoring; and 4) to examine the adaptation of our Tele-PCMHI model at each study site.

**Methods:** Our team qualitative expert/implementation scientist takes extensive fieldnotes during weekly implementation meetings and conducts weekly debriefing interviews with the two study facilitators to carefully and thoroughly document facilitation activities, pre-implementation and implementation processes and findings at each site, ongoing training/mentoring of the junior facilitator, and adaptation of the Tele-PCMHI model at each site. Detailed notes are cleaned and reorganized under major categories. Final notes are uploaded to Atlas.ti for coding using rapid analysis techniques.

**Findings:** Using these methods we are able to meet several study goals more efficiently and reduce burden on participating study sites by eliminating pre-implementation and implementation-focused interviews with site clinic personnel. We are also documenting the use of a dual-facilitator strategy (in which two facilitators work together with our participating sites), the pairing of a junior facilitator with a senior facilitator to accelerate the process of learning facilitation skills, and the process of training and mentoring. The latter results will feed into revisions of a gold-standard implementation facilitation manual and training program developed by two members of the study team. Facilitators are currently working in 4 of 6 study sites in a stepped-wedge design, and will begin engaging our final two sites in January of 2019.

**Implications for D&I Research:** This work will contribute to implementation science by: A) developing methods to more efficiently study implementation processes while also reducing perceived or actual burden on participating sites; B) expanding knowledge about implementation facilitation and the training of the next generation of facilitators, and C) studying the adaptation of a model for integrating mental health care into primary care in rural settings.

### S120 Measuring fidelity in healthyhearts NYC: A complex intervention using practice facilitation in primary care

#### Donna Shelley^1^, Allison Cuthel^1^, Melanie Corwin^1^, Nina Siman^1^, Charles Cleland^2^, Carolyn Berry^1^

##### ^1^New York University School of Medicine, New York, NY, USA; ^2^New York University Rory Meyers College of Nursing, New York, NY, USA

###### **Correspondence:** Carolyn Berry (Carolyn.Berry@nyumc.org)

**Background:** HealthyHearts NYC (HHNYC), funded through AHRQ’s national EvidenceNOW initiative, is studying the effectiveness of practice facilitation (PF) to support practice transformation in 255 small independent practices (SIPs) to improve cardiovascular disease (CVD) related outcomes. Current implementation science literature suggests that implementation fidelity moderates intervention outcomes, yet few studies measure all four subcategories of adherence ((1) Frequency (2) Duration (3) Content (4) Coverage) within the Conceptual Framework for Implementation Fidelity (CFIF) model. Retroactively applying the CFIF model, we assessed PF adherence among resource scare urban SIPs participating in the HHNYC intervention.

**Methods:** The present study uses the CFIF theoretical model to guide the evaluation of facilitators’ fidelity to the PF protocol outlined within the HHNYC stepped-wedge cluster randomized controlled trial. Pre-determined targets corresponding to the CFIF adherence subcategories of frequency and content were used to quantity fidelity of the intervention protocol. Targets were: Frequency: 13 onsite visits over the one year intervention period; Content: all CVD related tasks completed (n=39 tasks) and education of all chronic care model (CCM) practice strategies (n=27 strategies); Coverage: Defined as receiving 13 visits, and completing all CVD tasks and receiving education on all CCM strategies. No pre-set target was identified for ‘duration,’ as the program was not prescriptive with respect to length of visits or total number of hours. Due to the stringency of the coverage subcategory, the pre-determined target was 75% of all sites achieving complete coverage.

**Findings:** Of the full sample that received the intervention (n=255), 94% of sites (n=240) received the dose as intended (Frequency). Facilitators spent an average of 26.5 hours total (range 9.5 – 51.5 hours) delivering the HHNYC intervention (Duration). Practice facilitators completed all CVD tasks with 69% of the sites, and documented education of every CCM strategy in 71% of sites (Content). Over half (52.9%) of all sites that completed the HHNYC intervention received it as intended and achieved full Coverage (13 visits + completing all content (Tasks and education on CCM strategies).

**Implications for D&I Research:** As fidelity has the potential to impact intervention outcomes, future complex PF interventions should quantify all CFIF adherence measures to ensure the intervention is delivered as intended.

### S121 Time-motion analysis of implementing the collaborative chronic care model in general mental health clinics: Assessing external facilitation effort over time using continuous and interval-based data collection approaches

#### Bo Kim^1^, Christopher Miller^2^, Mona Ritchie^3,4^, Jeffrey Smith^4^, JoAnn Kirchner^4^, Mark Bauer^1^

##### ^1^Center for Healthcare Organization and Implementation Research (CHOIR), Veterans Health Administration, Boston, MA, USA; ^2^VA Boston Healthcare System,Center for Healthcare Organization and Implementation Research (CHOIR), Veterans Health Administration, Boston, MA, USA; ^3^University of Arkansas for Medical Sciences, Little Rock, AR, USA; ^4^Central Arkansas Veterans Healthcare System (North Little Rock), Veterans Health Administration, North Little Rock, AR, USA

###### **Correspondence:** Bo Kim (bo.kim@va.gov)

**Background:** Facilitation to implement evidence-based practices often involves an external facilitator (EF) who brings content and process improvement expertise to an implementation site. With facilitation being multi-faceted, activities and required time by the EF are not well known. Furthermore, collecting continuous time-motion data is challenging, particularly for relational and customized tasks that comprise much of facilitation. However, this information is essential for organizations allocating external implementation resources to sites. Thus, our objectives were to conduct a time-motion analysis of external facilitation and assess the representativeness of time-motion data collected over two-week intervals toward the implementation’s beginning, middle, and end.

**Methods:** We analyzed EFs’ time-motion data from six Veterans Health Administration (VA) mental health clinics implementing the evidence-based Collaborative Chronic Care Model (CCM). We adapted the VA Behavioral Health Quality Enhancement Research Initiative’s structured time-motion tracker to document EFs’ activities over 4-6 weeks of pre-implementation and 12 months of implementation periods. We collected continuous time-motion data for pre-implementation, followed by the aforementioned two-week interval data for implementation. To assess how closely the interval data represent external facilitation over the 12 months, we also collected continuous data throughout implementation for two of the sites.

**Findings:** EFs spent 21.8±4.5 hours/site for pre-implementation (orienting the site and assessing its contextual factors), then 27.5±4.6 hours visiting the site to initiate implementation. Based on the two-week interval data, EFs spent 2.5±0.8, 1.4±0.6, and 1.2±0.6 hours toward the implementation’s beginning, middle, and end, respectively -- 73.6% of these hours for communication (email/phone/video). For the two sites with continuous data, the computed average weekly time spent toward the implementation’s beginning, middle, and end differed from the interval data’s average by 1.0, 0.1, and 0.2 hours, respectively. Activities inconsistently captured in the interval data included irregular assessment, stakeholder engagement, and network development.

**Implications for D&I Research:** Time-motion analysis of CCM implementation showed EFs’ initial higher-intensity involvement that tapered over time, matching facilitation’s goal to transition external expertise to the site as the implementation progresses. The two-week interval data collection approach, if accounting for its underestimation of irregular activities, may be a promising efficient option for implementation studies collecting time-motion data to inform subsequent scale-up and spread.

### S122 Measuring the dose of external practice facilitation

#### Bijal Balasubramanian^1^, David Ezekiel-Herrera^2^, Shannon Sweeney^3^, Miguel Marino^2^, Rikki Ward^1^, Leah Gordon^2^, Benjamin Crabtree^3^, Leif Solberg^4^, William Miller^5^, Deborah Cohen^2^

##### ^1^University of Texas School of Public Health, Dallas, TX, USA; ^2^Oregon Health & Science University, Portland, OR, USA; ^3^Rutgers Robert Wood Johnson Medical School, New Brunswick, NJ, USA; ^4^Institute for Education and Research, HealthPartners, Bloomington, MN, USA; ^5^Lehigh Valley Hospital, Allentown, PA, USA

###### **Correspondence:** Bijal Balasubramanian (Bijal.A.Balasubramanian@uth.tmc.edu)

**Background:** Practice facilitation is an evidence-based strategy to support primary care practices in implementing quality improvements. Effective practice facilitation requires multiple components, but there is no established method to measure the dose of facilitation. The purpose of this study is to propose an approach to conceptualizing and measuring dose of facilitation delivered to 1,647 primary care practices by seven regional cooperatives to improve cardiovascular preventive care.

**Methods:** We conducted cross-cooperative comparative analyses using a convergent, mixed-methods design. Quantitative logs from facilitators provided data on date, duration, and mode (email, web, in-person) of facilitation encounters with practices. Qualitative data were collected through document review, site visits, and online diary entries from Cooperative members, as well as semi-structured interviews with facilitators and practice members of a subset of practices.

**Findings:** We observed substantial variation in the time from the first to last facilitation visit across practices (ranging from an average of 1.5 to 21 months), in number of in-person facilitation visits (ranging on average from 4 to 28), and in total amount of time practices received in-person facilitation (ranging on average from 7 to 50 hours). Qualitative data suggested these variations were attributable to Cooperative conceptual framework and design, practice engagement and motivation, information technology and data challenges, practice disruptions (e.g., staff changes), and competing demands. Guided by qualitative findings, we further characterized facilitation dose as: delays in starting facilitation visits, “lulls” between visits, and stops to facilitation prior to the intervention end date. We developed a theoretical framework showing components of facilitation dose, the factors that explain variation in dose, and how these factors might relate to facilitation effectiveness.

**Implications for D&I Research:** Comprehensive measurement of facilitation dose is important to assess effectiveness of facilitation as an implementation strategy. We propose an approach to measurement, document the high degree of variation, and identify the potential importance of motivation, engagement, and logistics for affecting dose and impact. Future mixed methods research will be needed to further explore the effect of these aspects of measuring dose on improving practice and patient outcomes.

## Prevention and Public Health

### S123 Understanding the role of readiness in promoting the adoption and effectiveness of evidence-based practices in prevention research

#### Allison Dymnicki^1^, Sabrina Arredondo Mattson^2^, Elizabeth Spier^3^, Beverly Kingston^2^, Frances Miller^4^, Jonathan Scaccia^5^

##### ^1^American Institutes for Research, Washington, DC, USA; ^2^University of Colorado Boulder, Boulder, CO, USA; ^3^American Institutes for Research, San Mateo, CA, USA; ^4^American Institutes for Research, Chicago, IL, USA; ^5^Wandersman Center, Reading, PA, USA

###### **Correspondence:** Allison Dymnicki (adymnicki@air.org)

**Background:** Researchers from the Center for the Study and Prevention of Violence (CSPV) are partnering with educators in 46 middle schools to implement the Safe Communities Safe Schools (SCSS) model. The model seeks to prevent and reduce problem behavior, address mental and behavioral health concerns, and increase prosocial behavior in students by addressing key malleable risk and protective factors. The model consists of three core program components: developing a functioning school-based team, building capacity around using data, and implementing evidence-based programs.

**Methods:** Researchers used a two-step readiness process to ensure that the schools had met a threshold of readiness prior to beginning the intervention. First, CSPV conducted readiness feasibility visits to assess indicators including leadership’s support of SCSS and the presence of a school-based champion. Second, school-based teams completed a 90-item readiness assessment to assess constructs such as the degree to which leadership has created structures that support implementation (Leadership support) and expect, reward, and support the SCSS model(Priority).

**Findings:** Readiness assessment data from the first two years of implementation suggest changes in readiness in the expected direction, with significant increases for leadership support for the SCSS model, *t*(49)= 2.51, *p* < .05, and priority to implement the SCSS model *t*(49)= 2.35, *p* < .05. The data also indicate that schools had a champion that supported the SCSS model at onset (Means for *Champion*=5.65 [Year 1], 5.87 [Year 2), school staff believed early on that this model was compatible with the school’s existing values, cultural norms, and past experiences (Means for *Compatibility*=6.06 [Year 1] , 6.10 [Year 2]), and school staff perceived implementation of the SCSS model to be a positive experience in both years (Means for *Joy* =6.18 [Year 1], *6.17 [Year 2]*.

**Implications for D&I Research:** Two years of data from a randomized controlled trial of the SCSS model suggest that the model shows promising results for improving the accessibility, adoption, and implementation of evidence-based programs in prevention research. We continue to evaluate the implementation of prevention strategies used with multi-sector teams to achieve school-level improvements in a variety of outcomes including violence and bullying, substance abuse, and mental health indicators.

### S124 Is organizational readiness to change an effect modifier in an implementation trial of a workplace wellness program?

#### Christian Helfrich^1,2^, Christine Kava^2^, Marlana Kohn^2^, Amanda Parrish^2^, Kristen Hammerback^2^, Gary Chan^2^, Daron Ryan^2^, Bryan Weiner^2^, Jeff Harris^2^, Peggy Hannon^2^

##### ^1^Seattle – Denver Health Services Research and Development Center of Innovation., VA Puget Sound Health Care System, Seattle, WA, USA; ^2^University of Washington, Seattle, WA, USA

###### **Correspondence:** Christian Helfrich (christian.helfrich@va.gov)

**Background:** Previous studies have found variation in how well implementation strategies are successful. This may be explained by differences in organizational readiness to change. If so, readiness assessed at the outset of a change initiative could be used to prioritize and tailor implementation support. However, few studies have prospectively assessed organizational readiness and its association with implementation outcomes over time.

**Methods:** We analyzed survey data (n=69) collected as part of a three-arm randomized controlled trial to implement evidence-based health promotion practices in small worksites in low-wage industries. We measured five factors adapted from Weiner’s theory of organizational readiness to change: context (favorable broader conditions); change valence (valuing health promotion); information assessment (demands and resources to implement health promotion); change commitment (an intention to implement health promotion); change efficacy (a belief in shared ability to implement health promotion). We used linear regression to test the hypotheses: H1) baseline change commitment and change efficacy will be associated with greater wellness program effort at 15 months (implementation) and 24 months (maintenance), and H2) the relationship between intervention arm and wellness program effort will be moderated by change commitment and context.

**Findings:** Change efficacy exhibited poor reliability (α=0.52) and was excluded from the analysis. Change commitment met reliability thresholds but was not associated with wellness program effort at either 15 or 24 months, and irrespective if the outcome was change in wellness program effort from baseline to follow-up or total wellness program effort score at follow-up. No significant interaction effects for change commitment were found. Wellness program effort exhibited significant increases in both intervention arms at 15 months (β=0.558, *p*=0.026 and β=1.046, *p*=0.000) and in one intervention arm at 24 months (β=0.893, *p*=0.001), relative to controls. We also tested the association of organizational context with wellness program effort as an independent predictor and effect modifier, and found no associations.

**Implications for D&I Research:** Many implementation models include affective states like change commitment as determinants of implementation, but we found no association between change commitment and wellness program effort. Additional research is needed to determine what conditions and for what innovations change commitment might be an important predictor of implementation success.

### S125 The first path to truth: Facilitators and barriers to the dissemination of evidence-based interventions to prevent and control cardiovascular disease

#### Erika Fulmer, Farah Chowdhury, Colleen Barbero, Refilwe Moeti, Sharada Shantharam, Aunima Bhuiya

##### Centers for Disease Control and Prevention, Atlanta, GA, USA

###### **Correspondence:** Erika Fulmer (efulmer@cdc.gov)

**Background:** In the US, heart disease and stroke together cause more premature death than any other condition. Given the scope of the problem, evidence-based population-wide approaches are needed to reverse the trend of increasing cardiovascular disease (CVD). To facilitate the dissemination and evidence-based system-level interventions, we utilized the Centers for Disease Control and Prevention’s Knowledge to Action (K2A) Framework to speed translation of research and practice-based evidence into public health action. The K2A Framework provides a common language and conceptualization of the translation process, from discovery to institutionalization. In this presentation, we examine facilitators and barriers to this work and discuss implications for future research.

**Methods:** We used the Framework to guide planning and execution of a broad portfolio of CVD prevention issues including gestational hypertension; community health worker workforce development; collaborative practice agreements with pharmacists; community clinical linkages; and stroke systems of care. Additionally, we engaged internal and external subject matter experts to develop targeted products, and enhance dissemination to priority audiences best situated to implement evidence-based, system-level interventions.

**Findings:** Dissemination has been facilitated by active engagement of diverse public health and health care partners; incorporation of product findings into conceptual models, funding opportunities, and national policy guidelines; and a commitment to the time intensive work of building capacity at multiple levels and settings using multi-modal dissemination channels. Barriers include the complexity and cost of evaluating a broad array of products as well as the time needed to determine the contribution of translation activities to CVD prevention practice.

**Implications for D&I Research:** Application of the K2A Framework helps bridge understanding across stakeholders from a variety of disciplines, sectors, and approaches to CVD prevention. It provides greater clarity on how public health stakeholders at different levels might contribute to the dissemination and implementation of system-level interventions and helps to highlight where greater investment in translation-related surveillance and monitoring could enhance future research.

### S126 Addressing barriers and facilitators to incorporating cancer prevention clinical decision support into primary care

#### Melissa Harry^1^, Daniel Saman^1^, Anjali Truitt^2^, Hillary Henzler-Buckingham^1^, Clayton Allen^1^, Patrick O’Connor^2^, Heidi Ekstrom^2^, JoAnn Sperl-Hillen^2^, Joseph Bianco^3^, Thomas Elliott^2^

##### ^1^Essentia Institute of Rural Health, Essentia Health, Duluth, MN, USA; ^2^HealthPartners Institute, Minneapolis, MN, USA; ^3^Essentia Health - Ely Clinic, Ely, MN, USA

###### **Correspondence:** Melissa Harry (melissa.harry@essentiahealth.org)

**Background:** Primary care providers (PCP) routinely balance acute, chronic, and preventative patient care delivery, including cancer prevention and screening, within time-limited visits. Clinical decision support (CDS) may assist PCPs in prioritizing cancer prevention and screening with other patient needs. In a three-arm, 36 clinic-randomized control trial, we are testing a CDS system in a large northern Midwestern healthcare system. The electronic health record (EHR)-based CDS integrates evidence-based cancer prevention (HPV vaccination) and screening recommendations (cervical, breast, colorectal, lung) within an existing cardiovascular risk management CDS system. One intervention arm receives the integrated cancer prevention and cardiovascular CDS system, and another the integrated CDS system and shared decision-making tools (SDMT) for breast, colorectal, and lung cancer screening and human papillomavirus vaccination. Control arm clinics receive usual care. From healthcare system key stakeholders, we identified pre-implementation barriers and facilitators to employing the integrated CDS system in primary care settings.

**Methods:** Study team members interviewed 28 key stakeholders (13 PCPs, 2 rooming staff, 13 care delivery leaders). The Consolidated Framework for Implementation Research informed the development of the semi-structured interview guide questions. Transcribed interviews were analyzed using qualitative content analysis. This study was IRB-approved.

**Findings:** Main barriers identified included: PCP time limitations; EHR alert fatigue; competing priorities; concerns about duplicating care; and lack of clinic resources. Main facilitators included: the comprehensive and unified cancer prevention and cardiovascular risk management patient and provider CDS handouts; optimizing workflow; SDMT utility; team use; alignment with institutional aims and quality measures; and employing a multi-modal training plan. Based on this feedback, the research team developed and instituted specialized training, pilot testing, and implementation plans to maximize facilitators and address barriers.

**Implications for D&I Research:** Identifying and addressing barriers and facilitators identified by key stakeholders in primary care settings pre-implementation can assist with intervention implementation and use. We are continuing to elicit feedback through PCP and patient surveys, patient focus groups, and post-implementation stakeholder interviews in later study years, with the dual goals of continual improvement and subsequent adoption of the integrated cancer prevention and cardiovascular risk management CDS intervention across the healthcare system.

### S127 When implementation strategies don’t go as planned: How do community organizations make adaptations?

#### Courtney Luecking, Byron Powell, Dianne Ward

##### University of North Carolina at Chapel Hill, Chapel Hill, NC, USA

###### **Correspondence:** Courtney Luecking (clueckin@email.unc.edu)

**Background:** While efforts to improve the specificity and consistency in reporting implementation strategies are made, adaptations to such strategies are seldom discussed. Adaptations could have important effects on implementation and service outcomes. We aimed to measure adherence to and adaptations of a multifaceted implementation strategy for a community intervention for early care education (ECE) providers and parents in support of healthy eating and physical activity among preschool-aged children.

**Methods:** A two-phase sequential elaboration mixed methods design (Quan-QUAL) was embedded to a quasi-experimental study investigating the effect of an enhanced implementation strategy on parent engagement with a healthy habits intervention. In Phase 1, at the midpoint and end of the intervention, ECE providers (n=52) completed surveys to identify presence or absence of discrete implementation strategies. In Phase 2, after completing the intervention, a subset of ECE providers (n=37) completed semi-structured interviews to provide greater specification about execution and adaption of strategies to encourage parents to implement the intervention at home.

**Findings:** Preliminary survey findings show ECE providers did not consistently adhere to prescribed strategies for involving parents (75%) or obtaining and using feedback from parents (69%). While most ECE providers (77%) spoke with other staff about barriers for parent engagement and brainstormed ideas to overcome barriers, only about half of the providers worked with parents to overcome barriers for implementation at home. During the intervention, ECE providers received support from research staff through educational meetings, dynamic training, and centralized technical assistance; however, adherence for engaging parents did not increase between the midpoint and completion of the intervention. Interviews suggest organizational, interpersonal, and familial barriers resulted in intentional or unintentional modifications to the actors, specific actions, and dose of the advised implementation strategies.

**Implications for D&I Research:** Results indicate measuring adaptation provides important context not only for interpreting the effectiveness, or lack thereof, of implementation strategies within a specific intervention or setting, but also for future selection and tailoring of strategies in similar settings. Results also raise an important concern for achieving adequate implementation support for intended intervention targets in multi-level interventions.

### S128 Understanding country exemplars in under-five mortality reduction: Development and application of an implementation science framework to explore implementation strategies, outcomes and contextual factors

#### Lisa Ruth Hirschhorn^1^, Caroline Beyer^1^, Felix Sayinzoga^2^, Raj Kumar Subedi^3^, Kelechi Udoh^1^, Sall Mohamadou^4^, Mahesh Kumar Maskey^3^, Kateri Donahoe^1^, Agnes Binagwaho^1^

##### ^1^University of Global Health Equity, Kigali, Rwanda; ^2^Rwanda Biomedical Center, Kigali, Rwanda; ^3^Nepal Public Health Foundation, Kathmandu-4, Nepal; ^4^Cheikh Anta Diop University, Dakar, Senegal

###### **Correspondence:** Lisa Ruth Hirschhorn (Lisa.Hirschhorn@Northwestern.edu)

**Background:** Understanding how low- and middle-income countries (Exemplar LMICs) have more effectively dropped under-5 Mortality (U5M) (exemplars) offers important lessons on how evidence-based interventions (EBIs) to reduce U5M were chosen, adapted, implemented and sustained and contextual factors which facilitated or hampered success. However, gaps exist in knowledge of implementation strategies and outcomes which implementation science (IS) methods can help bridge. We report the development and application of a novel IS framework to emerge transferable knowledge on exemplar country implementation strategies and outcomes and lessons learned to inform other countries working to save children’s lives.

**Methods:** We reviewed existing IS frameworks, measures and selected case studies of country U5M reduction to develop a framework to guide data collection and analysis. We used the Exploration, Preparation Implementation, Sustainment (EPIS) Framework to include an explicit Adaptation during implementation, integrating implementation outcomes and implementation strategies (Proctor), and the Consolidated Framework for Implementation Research to explore contextual factors. Exemplar countries were those with higher declines in U5M compared with countries in the region or similar economic resources. EBIs were identified from literature and recommendations. Data analysis included literature extraction, coverage and cause of death from existing surveys, and key Informant Interviews from national, ministry, implementer partner, donor and community.

**Findings:** The framework was successfully used in three countries to inform data collection and analysis, identifying implementation strategies and outcomes and emerging cross-cutting and country specific themes. Implementation strategy lessons include: combining leadership and accountability at all levels; engagement of all stakeholders during and throughout EPIS process; alignment with national priorities and strategies; use of evidence (produced in-country or from other sources) to inform adaptation and scale, and ongoing data use by to assess and inform adaptation of EBI and implementation strategies.

**Implications for D&I Research:** We describe how IS methods and frameworks adapted to areas of study and context can help researchers, policy makers and implementers understand how EBIs were implemented, implementation outcomes and factors which influence success. Knowledge generated is designed to be transferable to other countries working to strengthen progress in saving children’s lives.

Ethics approval received from each country.

### S129 Formative evaluation and adaptation of a safe sleep intervention for rural black infants

#### Rosemary Nabaweesi^1^, Mary Aitken^1^, Leanne Whiteside-Mansell^2^, Samantha Mullins^2^, Keneshia Bryant^2^, Geoffrey Curran^2^

##### ^1^University of Arkansas Medical Sciences/ Arkansas Children's Research Institute, Little Rock, AR, USA; ^2^University of Arkansas Medical Sciences, Little Rock, AR, USA

###### **Correspondence:** Rosemary Nabaweesi (rnabaweesi@uams.edu)

**Background:** In the US, close to 4,000 infants die annually due to Sudden Unexpected Infant Death (SUID). SUID includes SIDS (Sudden Infant Death Syndrome) and other sleep-related deaths due to accidental suffocation and strangulation in bed. Subsequently, the American Academy of Pediatrics developed evidence-based recommendations targeting the modifiable sleep environment. Implementation of these recommendations remains suboptimal, as illustrated by the significant racial and rural-urban health disparities. Currently, Black infants remain twice as likely to die from SUID as White infants, and are less likely to be placed in a supine “safe” position for sleep compared to White infants. Research identifies familial and cultural influences as barriers to safe sleep compliance among Black parents. The safety baby shower (SBS) is an evidence-based intervention shown to increase safe sleep knowledge among Blacks, but not long term behavioral change. Study Aims: 1) Explore rural Black parents’ and their advisors’ perspectives on the SBS’ acceptability, feasibility, and adaptability. 2) Adapt the SBS and identify promising implementation strategies to support adaptation through an Evidence-Based Quality Improvement process with a multistakeholder group.

**Methods:** Collaborating with a local community organization, we explored community advisors’ and expectant women’s SBS experiences to understand intervention delivery and adoption in a rural underserved community (RUC). The Consolidated Framework for Implementation Research guided our data collection and analysis using focus groups and key informant interviews. We used directed content analysis to generate themes and subthemes. In the next study phase, identified themes will inform the SBS adaptation and suggested implementation strategies will support uptake of the adapted SBS.

**Findings:** Five focus groups (21 participants) and one key informant interview were conducted. Identified barriers fit three categories: 1) Intervention- significantly complex and costly, 2) Outer settings- limited transportation and childcare resources, and 3) Inner Settings- small voluntary organization with few incentives. Social media emerged as a facilitator, and integrating safe sleep education into personal baby showers emerged as an implementation strategy.

**Implications for D&I Research:** Identifying transformative implementation strategies and conducting a community-informed SBS adaptation using a collective decision-making process between intervention experts and local community partners will support improved SBS delivery, adoption and sustainability in RUCs.

### S130 Are we there yet? readiness of community-based organizations to adopt and implement evidence-based home visiting programs

#### Sarah Kaye^1^, Deborah Perry^2^

##### ^1^Kaye Implementation & Evaluation, LLC, Riverdale Park, MD, USA; ^2^Center for Child and Human Development, Georgetown University, Washington, DC, USA

###### **Correspondence:** Sarah Kaye (sarah@kayeimplementation.com)

**Background:** The Maternal Infant and Early Childhood Home Visiting (MIECHV) program is an unprecedented nationwide scale-up of evidence-based home visiting (EBHV) programs, funded by the USDHHS Health Resources and Services Administration, to support maternal and child health outcomes. This presentation shares findings from the adoption sub-study of a RE-AIM evaluation of scaling EBHV in one diverse urban community, which funded Local Implementation Agencies (LIA) through a request for proposal (RFP) process. The study aim was to pilot a community-engaged research methodology to study influences on community-based organizations’ decisions to adopt EBHV and progress through implementation stages.

**Methods:** Researchers presented several readiness frameworks to a community advisory board, who selected the Texas Christian University Program Change Model (Simpson & Flynn, 2007) to guide the sub-study, and provided feedback on semi-structured interview guides. In phase one, completed before release of the RFP, key informant interviews with eligible organizations gathered data about the organization’s motivation, resources, and staff attributes (n=10, 67% response rate). In phase two, completed after the state identified LIAs, interviews with program staff collected data about how LIAs were preparing to implement core components of the EBHV model (n=12, 100% response rate). Researchers developed a coding scheme with observable anchors operationalizing each component of the EBHV model as fully ready, ready, and approaching readiness. Degree of adoption and implementation was measured by progression through TCU’s stages of implementation (i.e., adoption, planning and preparation, implementation, practice improvement).

**Findings:** Adoption and successful implementation of EBHV was limited among community-based organizations in this community. In phase one, thematic analysis of decision makers’ deliberations included internal and external considerations that were pragmatic, altruistic, and analytic in nature. Organizations demonstrated significant variation in their infrastructure, particularly their data collection and reporting capacity. Phase two findings provide early indications of criterion and predictive validity; the more mature implementation site had higher readiness scores, and the less ready site did not progress beyond the planning stage.

**Implications for D&I Research:** Implementation researchers can benefit from a realistic understanding of the strengths, challenges, and capacities of the community-based organizations who ultimately are expected to implement evidence-based programs.

### S131 Examining the adoption of a postpartum depression intervention in a state network of home visiting programs

#### J.D. Smith^1^, Molly McGown^1^, Carol Brady^2^, Darius Tandon^3^

##### ^1^Northwestern University, Feinberg School of Medicine, Chicago, IL, USA; ^2^Health Resources and Services Administration, Tallahasse, FL, USA; ^3^Northwestern University, Chicago, IL, USA

###### **Correspondence:** J.D. Smith (jd.smith@northwestern.edu)

**Background:** 30-45% of low-income women exhibit elevated depressive symptoms and are, therefore, at risk for developing postpartum depression. Mothers and Babies (MB) is an intervention with demonstrated efficacy in preventing the onset and worsening of postpartum depression. Home visiting programs exist across all 50 states and are promising settings for scaling up evidence-based interventions (EBIs) like MB. Train-the-trainer approaches are commonly used for scaling up EBIs in practice networks. However, there are challenges to ensuring that adoption spreads evenly across a network and reaches intended recipients due to differences in organizational, staff, and client-level variables. This presentation a) describes a train-the-trainer approach used among 32 home visiting coalitions across Florida to implement MB and b) presents findings on adoption and penetration of MB within these coalitions.

**Methods:** Mental health clinicians trained as MB trainers provided local trainings along with six monthly supervision sessions for home visitors from the 32 coalitions. Post-training surveys were conducted with clinicians and home visitors. A centralized management information system was used to extract data on coalition and home visitor-level adoption--i.e., whether a coalition and each home visitor within a coalition began implementing MB. We also used management information system data to determine the extent to which women at-risk for postpartum depression (based on Edinburgh Postnatal Depression Scale scores 8-12) received MB.

**Findings:** Five train-the-trainer workshops trained 93 mental health clinicians, who subsequently trained 521 home visitors on MB. Ninety-one percent of coalitions adopted MB between 7/1/17 and 7/1/18 with 48% of the home visiting staff trained on MB delivering MB to at least one client. Only 19% of women with EPDS scores 8-12 received MB, indicating relatively poor penetration of the intervention during its first year of implementation. Data on coalition, home visitor, and client variables that influence adoption and penetration will be presented.

**Implications for D&I Research:** This study suggests that the train-the-trainer model paired with ongoing supervision can facilitate the statewide implementation of a postpartum depression EBI. Moreover, we present factors that may be predictive of adoption and/or sustainability and discuss possible best practices for intervening upon those factors to reduce unevenness in the implementation of EBIs.

### S132 Scaling up HPV vaccination coverage: Predictors and implications for implementation

#### Margaret Padek, Melissa Franco, Stephanie Mazzucca, Ross Brownson

##### Washington University in St. Louis, St. Louis, MO, USA

###### **Correspondence:** Margaret Padek (mpadek@wustl.edu)

**Background:** The human papillomavirus (HPV) vaccine is an underutilized cancer control practice in the United States. Although individual contextual factors are known to affect HPV vaccine coverage rates, the impact of macro-level elements (e.g., policies) are unclear. The aim of this study was to understand the underuse of the HPV vaccine, in particular to explore broader-level correlates influencing completion rates. It further provides a base knowledge of how to tailor scale-up and implementation strategies for HPV vaccine uptake to identified underserved populations.

**Methods:** A comprehensive database was developed using individual-level data from the National Immunization Survey (NIS)-Teen (2016) and state-level data collected from publically available sources to analyze HPV vaccine completion. Multi-level logistic models were built to identify significant correlates. Level-1 (individual) and level-2 (state) correlates were fit to a random intercept model. Deviance and AIC assessed model fit, and sampling weights were applied.

**Findings:** The analysis included 20,495 adolescents from 50 U.S. states and the District of Columbia. Age, gender, race/ethnicity, and maternal education were significant individual predictors of HPV completion. Significant state-level predictors included sex education policy, religiosity, and HPV vaccine mandate. The analysis suggests living in highly religious states have an 11-fold decrease (with by far, the largest effect estimates) in HPV vaccine completion rates. Additionally, individuals living below poverty were 1.4 times less likely to complete the HPV vaccine. As various contextual and situational factors affect HPV vaccine completion rates, gender, political ideology, religiosity, and sex education policies were not found to have similar impacts on Tdap and MMR vaccine rates.

**Implications for D&I Research:** Given that gender, religiosity, political ideology, and education policies were predictors of HPV vaccine completion, the interaction and underlying mechanisms of these factors can be used to address the underutilization of the HPV vaccine (i.e., the lack of scale-up). This knowledge can be used to better tailor public health campaigns taking into account contextual conditions and existing implementation strategies can be adapted to maximize uptake of this cancer control tool. This analysis can be used to better understand why some HPV vaccine uptake programs are under-utilized despite their strong evidence-base for cancer control.

### S133 Implementation support for HPV vaccination: Should we target clinic systems, provider behaviors, or both?

#### Jennifer Leeman^1^, Melissa Gilkey^2^, Jennifer MacKinnon^2^, Belinda-Rose Young^2^, Noel T. Brewer^2^

##### ^1^University of North Carolina School of Nursing, Chapel Hill, NC, USA; ^2^Gillings School of Global Public Health, University of North Carolina at Chapel Hill, Chapel Hill, NC, USA

###### **Correspondence:** Jennifer Leeman (jleeman@email.unc.edu)

**Background:** HPV vaccination prevents multiple cancers, and yet only 43% of adolescents have completed the multi-dose series. One approach to supporting implementation of HPV vaccination is for staff in state health departments to deliver quality improvement (QI) coaching to clinics. A strength of QI coaching is its focus on improving care systems, such as flagging eligible patients and using reminder/recall to bring them to clinic. The impact of QI coaching may, however, be limited when clinics lack capacity to change systems, have competing QI priorities, and experience staff turnover. An alternative is to provide continuing medical education (CME) that directly targets healthcare providers’ vaccination recommendation practices. Both approaches increase vaccination rates, but little is known about their relative effectiveness and efficiency.

**Methods:** We are conducting a four-arm randomized controlled trial to compare (1) QI coaching, (2) HPV vaccine-related CME, (3) QI coaching and CME, and (4) a control CME intervention in 360 primary care clinics in three US states. A tracking log collected data on adoption (# and % of clinics that participated) and reach to providers (# and % of providers that participated). An online survey assessed providers’ vaccination knowledge and beliefs and perceptions of intervention acceptability and feasibility immediately post intervention. A second online survey assessed QI capacity at baseline, and clinics’ use of QI processes to change care systems at baseline and three months post-intervention.

**Findings:** As of July 2018, 151 clinics had completed the intervention, and all clinics will have complete in September 2018. At that time, we will analyze data to compare rates of adoption and reach, perceptions of intervention acceptability and feasibility, and impact on providers’ knowledge and beliefs across arms. Data comparing impact on clinics’ QI processes will be available for two thirds of clinics, and will include an analysis controlling for clinics’ baseline QI capacity. Current recruitment data suggest that more clinics adopt QI coaching but CME reaches more providers.

**Implications for D&I Research:** Implementation support is essential to improving HPV vaccination and other preventive services. Findings from this trial will contribute to the evidence base guiding when to target implementation support at care systems versus providers’ behavior.

### S134 Associations between patient navigation activities and number of women navigated within a large federal cancer screening program

#### Wendy Barrington^1^, Cam Escoffery^2^, Annette Maxwell^3^, Allison Cole^1^, Thuy Vu^1^, Peggy Hannon^1^

##### ^1^University of Washington, Seattle, WA, USA; ^2^Emory University, Atlanta, GA, USA; ^3^University of California Los Angeles, Los Angeles, CA, USA

###### **Correspondence:** Wendy Barrington (wendybar@uw.edu)

**Background:** Patient navigation (PN) is promising approach to enhance care coordination and facilitate patient adherence to recommended health services. The National Breast and Cervical Cancer Early Detection Program (NBCCEDP) is a federally funded screening program that supports access to and completion of breast and cervical cancer screening and diagnostic follow-up. NBCCEDP is administered by grantee state, tribal, and territorial entities and most are now implementing PN. Yet, it is unknown what PN activities best predict the number of women navigated through the NBCCEDP.

**Methods:** We used the Annual Survey of the NBCCEDP Grantees’ Program Implementation for program year (PY) 4 and PY5 to obtain measures of PN activities delivered, number of women navigated, as well as program characteristics among 67 grantees. Factor analysis was employed to identify clusters among 10 PN activities that were then used to build and test predictive models of the number of women navigated.

**Findings:** 65 of 67 grantees used PN and provided data on PN activities in PY4 only while 49 grantees provided data on the number of women navigated for PY4 and PY5. Two factors of 5 patient navigation activities were identified that corresponded with activities that centered on logistical and relational support, respectively. Goodness of fit of models for number of women navigated in PY4 were similar for both factors and significant when 3+ activities of each factor were delivered. Only delivery of 3+ “logistical” PN activities was associated with a greater number of women navigated in PY5 (Diff: 3503.2; 95% CI: 1485.9, 5520.4; P=0.001) adjusted for number of navigators used and amount of extra program funding above and beyond NBCCEDP grant dollars. This association remained after also adjusting for delivery of 3+ “relational” PN activities, albeit it was slightly attenuated.

**Implications for D&I Research:** These findings provide the first examination of PN activities delivered within NBCCEDP and suggest a delivery model that facilitates a greater number of women navigated. Assuming that a higher number of women navigated translates to a higher number of women screened, this may inform best practices of PN delivery across grantee programs to promote health equity.

### S135 Going to scale with evidence-based interventions: The next frontier for prevention science

#### Brittany Cooper^1^, Richard Barth^2^, Catherine Bradshaw^3^, Brian Bumbarger^4^, Abigail Fagan^5^, Lauren Supplee^6^, Deborah Walker^7^

##### ^1^Washington State University, Pullman, WA, USA; ^2^School of Social Work, University of Maryland, Baltimore, MD, USA; ^3^Curry School of Education, University of Virginia, Charlottesville, VA, USA; ^4^Colorado State University, Fort Collins, CO, USA; ^5^University of Florida, Gainesville, FL, USA; ^6^Child Trends, Bethesda, MD, USA; ^7^Boston University School of Public Health, Boston, MA, USA

###### **Correspondence:** Brittany Cooper (brittany.cooper@wsu.edu)

**Background:** The prevalence of many behavioral health and related problems have increased over the last decade, even in the face of a growing knowledge and evidence base. Many different programs, policies, and practices have been shown to prevent such problems, but there are very few evidence-based interventions (EBIs) that have been implemented at the scale needed to impact population health. Scaling-up EBIs is a major goal of the Society for Prevention Research (SPR) and therefore, in 2017, they formed the Mapping Advances in Prevention Science (MAPS) IV Task Force, which was charged with identifying the barriers to and facilitators of EBI scale-up. The Task Force focused this work on 5 public systems (education, child welfare, juvenile justice, public health, and behavioral health) because a) they are the interface between EBIs and those that need them, and b) because scale-up efforts will inevitably occur within, not across, systems.

**Methods:** The Task Force’s assessment was conducted in two steps. First, they created system-specific work groups comprised of research scientists working with practitioners and policy makers in these systems. Each group used the scientific literature and the members’ tacit knowledge and experiences to document system-specific barriers and facilitators of EBI scale-up, and make recommendations to increase EBI scale-up in that system. Second, the Task Force reviewed each work groups’ conclusions and identified where there were unique and common themes.

**Findings:** In all, the Task Force identified seven types of barriers to/facilitators of EBI scale-up in public systems. They include: 1) statutory endorsement and funding, 2) community involvement and capacity, 3) data monitoring and evaluation capacity, 4) workforce development, 5) EBI knowledge, 6) public system leadership support, and 7) EBI developer and funder capacity.

**Implications for D&I Research:** The information compiled by the Task Force informed the development of an Ecological Model for EBI Scale-up in Public Systems, which can be used by practitioners to begin breaking down the barriers and creating more opportunities for EBI scale-up at a systems level, and by D&I researchers who aim to systematically study the scale-up process.

### S136 Scale up of a multi-strategic intervention to increase implementation of a mandatory school healthy food service policy: The ‘healthy food@school’ program

#### Kathryn Reilly^1,2,3^, Nicole Nathan^2,3^, Sze Lin Yoong^2,3^, John Wiggers^2,3^, Luke Wolfenden^2,3^

##### ^1^Hunter New England Local Health District, Newcastle, Australia; ^2^Hunter New England Local Health District, Wallsend, Australia; ^3^School of Medicine and Public Health, University of Newcastle, Callaghan, Australia

###### **Correspondence:** Kathryn Reilly (Kathryn.Reilly@hnehealth.nsw.gov.au)

**Background:** Schools internationally have developed healthy eating policies as a key strategy in reducing the burden from chronic disease. Studies indicate that the implementation of such policies is poor. Several barriers to schools’ implementation of these policies have been identified. A number of trials have identified strategies that improve policy compliance however these have not been conducted at-scale. The ability to deliver strategies across a large number of schools and maintain effectiveness is unknown. The aim of this study is to assess the effectiveness and cost-effectiveness of implementation support strategies of a healthy food service policy in Australian schools.

**Methods:** Development of the program involved systematic assessment of a suite of implementation strategies previously shown to be effective in changing professional practice in clinical settings but not in non-clinical settings. The strategies included educational outreach visits, educational materials, audit and feedback, and centralized technical assistance. Assessment of different combinations of strategies was undertaken, including use of different modes of support delivery, including face-to-face, telephone and digital. The development of the program involved a number of rigorous scientific methods not previously applied to the assessment of implementation strategies to enhance school canteen policy adherence; i) Conduct of serial controlled implementation trials using common designs/methods; ii) Objective measurement of child nutrition intake (food purchases) and school guideline adherence (menu audit); iii) Conduct of cost effectiveness analyses of strategies; and iv) Evaluation of implementation at-scale.

**Findings:** Initial development demonstrated high intensity support achieved 63% guideline adherence and significantly reduced child fat intake, but with high implementation costs. Low and mid intensity support approaches were tested, with the latter, having the lowest cost (adherence 36%). The ‘healthy food@school’ program was subsequently delivered to 170 primary schools, achieving 35% guideline adherence across all schools, those in rural areas, urban areas and those with a high proportion of Aboriginal students.

**Implications for D&I Research:** This study extends previous research regarding how best to support schools at a population level to implement evidence-based policies to improve child obesity. These results have the potential to guide health promotion practitioners to facilitate wide-scale adoption and implementation of effective healthy eating interventions.

### S137 Evaluating the effectiveness of a statewide community coalition-based model for scaling up substance use prevention programs

#### Gitanjali Shrestha, Brittany Cooper, Laura Hill

##### Washington State University, Pullman, WA, USA

###### **Correspondence:** Gitanjali Shrestha (gshrestha@wsu.edu)

**Background:** A priority for the prevention field is to identify effective models to scale up evidence-based interventions for adolescent substance use prevention. Coalition-based models in which community coalitions implement prevention strategies while receiving state or federal technical assistance may provide the needed infrastructure for widespread scale-up. The Community Prevention and Wellness Initiative (CPWI) is a strategic, data-informed, community coalition-based model for reducing underage substance use and related risk factors. In the CPWI model, Washington State Division of Behavioral Health and Recovery (DBHR) provides funding, technical assistance, and training to community coalitions in high-risk communities to help them implement evidence-based prevention programs and activities. DBHR started CPWI in 2011 to prioritize prevention fund allocation to traditionally underserved, high-need communities throughout the state. CPWI is unique in its community selection approach because communities are selected based on risk scores computed from key substance use and consequence indicators. In this cross-sectional study deemed exempt by IRB, we evaluated the effectiveness of CPWI in reducing 10th grade substance use and related risk factors in CPWI Cohort 1.

**Methods:** The sample consisted of 5,000 students from Cohort 1 communities and 40,000 students from non-CPWI communities who participated in the statewide Healthy Youth Survey. We used multilevel level modeling on propensity-score adjusted data to examine whether CPWI differed significantly from non-CPWI 10th graders in outcomes of interest in 2016 compared to baseline (2008). Cohort 1 contracts began in July 2011 and direct services began in September 2011.

**Findings:** In 2016, CPWI students did not differ significantly from non-CPWI students in alcohol use outcome, and three risk factors in family, school, and community domains. This is an improvement when compared with baseline results in which CPWI students were at significantly higher risk for all four outcomes. Thus, CPWI was successful in reducing alcohol use and related risk factors.

**Implications for D&I Research:** CPWI is a promising model for scaling up prevention programs which can be used in other states for widespread scale-up of programs. Our next step is to conduct an implementation evaluation to determine what implementation factors distinguish more successful CPWI coalitions from less successful ones.

### S138 Lessons learned in scaling up evidence based practices in public health

#### Peg Allen^1^, Rebekah Jacob^1^, Carol Brownson^2^, Jean O'Connor^3,4^, Natalicio Serrano^1^, Kathryn Bass^1^, Samuel Yang^1^, Ross Brownson^1,5,6^

##### ^1^Brown School, Prevention Research Center, Washington University in St. Louis, St. Louis, MO, USA; ^2^National Association of Chronic Disease Directors, Decatur, GA, USA; ^3^Rollins School of Public Health, Emory University, Atlanta, GA, USA; ^4^The Task Force for Global Health, Decatur, GA, USA; ^5^Division of Public Health Sciences, Department of Surgery, Washington University in St. Louis, St. Louis, MO, USA; ^6^Alvin J. Siteman Cancer Center, Washington University School of Medicine, St. Louis, MO, USA

###### **Correspondence:** Peg Allen (pegallen@wustl.edu)

**Background:** Evidence of the value of applying preventive and disease management approaches in public health programs has increased in recent decades. However, a greater understanding of organizational infrastructure to support capacity to implement evidence-based strategies is needed. This presentation provides actionable lessons learned on building organizational supports for scale up derived from a decade of studies with state and local public health departments in the United States and Europe.

**Methods:** Data to support the recommendations are from rigorous quantitative studies and key informant interviews conducted with state health departments (SHDs) and local health departments (LHDs) from 2008-2017. Surveys and interviews explored and tested supports for evidence-based practice and barriers to implementation. Several published peer-reviewed articles detail each study’s methods; here we synthesize main findings and themes in D&I research areas that need further action

**Findings:** Use of research evidence increased over time among staff with perceived agency leadership support, supervisory support, access to evidence, and participatory decision-making compared to staff without such supports. Access to research evidence increased in SHDs that received intervention (EBPH training and technical assistance) compared to SHDs without intervention. In-person, researcher-led training in evidence-based public health (EBPH) reduced gaps in staff skills. Train-the-trainer models and blended learning models (distance and in-person) similarly reduced skill gaps and facilitated scaling up of trainings. Public health organizations need ongoing tailored training in specific skill areas to supplement basic EBPH training, as well as leadership training for managers. Having a learning orientation, sharing information across administrative units, and embedding procedures to ensure data-driven decision-making create organizational climates and cultures supportive of EBPH scale-up. Scale up can also be supported by engaging health department partners in ongoing and meaningful ways, including soliciting participatory input into written EBPH contract expectations.

**Implications for D&I Research:** D&I research and practice benefit from incorporation of organizational behavior in conceptual frameworks. D&I researchers can further understanding by improving measurement of organizational supports for EBPH. With better measures, D&I researchers can identify which organizational supports are key for EBPH scale-up. D&I researchers can forge partnerships with public health organizations to help provide EBPH trainings, evaluation, and practice-based research.

### S139 *keepin’ it REAL* with D.A.R.E. America: Using partnership and technology to enhance implementation and dissemination

#### Michael Hecht^1^, Michelle Miller-Day^2^

##### ^1^Communication, REAL Prevention LLC, Clifton, NJ, USA; ^2^Communication, REAL Prevention LLC, Placentia, CA, USA

###### **Correspondence:** Michael Hecht (hechtpsu@gmail.com)

**Background:** k*eepin’ it REAL*, one of three evidence-based intervention recommended in the 2016 Surgeon General’s report and believed to be the most widely disseminated curriculum of its kind. Since its adoption by D.A.R.E., kiR is used in 75% of the elementary, middle and high schools in the U.S. as well as those in over 50 other countries. The original curriculum was culturally grounded in Latino, African American and White cultures and then adapted for use by D.A.R.E. Officers as well as for rural audiences based on the Principle of Cultural Grounding and Narrative Engagement Theory. Recently, a technology-based version of the elementary curriculum was developed and evaluated.

**Methods:** A series of studies utilizing group randomized trial designs have been conducted describing development and effectiveness. One study will be highlighted in which implementers were videotapes teaching kiR. Other papers have described cultural adaptation as well as examining the introduction of a digitalized version of the curriculum for use by Police Officers.

**Findings:** kiR demonstrates long-term effects on substance use among various populations. In addition, research demonstrates the role of implementation quality in its delivery is more important than mere fidelity in determine outcomes. Finally, studies demonstrate the challenges of taking technology-based curriculum to scale.

**Implications for D&I Research:** The findings of these studies demonstrate: (1) the importance of partnership and engaging multiple constituencies if programs are to be taken-to-scale; (2) the role of engagement and implementation quality in determine program outcomes; (3) the need to consider culture in curriculum development, implementation, and dissemination. Keepin’ it REAL demonstrates the needs for advanced implementation designs that address the complexity in prevention and public health contexts as well as implementation strategies for engaging key stakeholders. It also, suggests, limitations of the role technology may play in these processes.

### S140 Reducing risky sexual behaviors through encouraging Latinas to become “mighty girls”: Using interactive videogame technology to implement narrative prevention messages

#### Anne Norris (aen16@miami.edu)

##### School of Nursing and Health Studies, University of Miami, Coral Cables, FL, USA

**Background:** Sex education is a highly politicized and morally-charged field. The need for evidence-based interventions that overcome challenges is great given the human and social costs of risky sexual behaviors. These costs are even greater among adolescent females ages 12-14, and particularly great among young Latinas. Mighty Girls adapted the narrative-based keepin’ it REAL curriculum to meet these challenges.

**Methods:** Formative research was conducted to reground keepin’ it REAL for this purpose. Next, Narrative Engagement Theory guided the development of a hybrid curriculum consisting of implementer-led after school sessions as well as an innovative, interactive videogame. Finally, a group randomized trial was conducted in schools in Miami, Florida.

**Findings:** Mighty Girls reduced risky sexual behaviors and the sexual pressure resistance approach overcame political and ideological concerns.

**Implications for D&I Research:** Public health interventions increasing face not only the challenge of developing and implementing evidence-based practices, but, as well, overcoming resistance of a political and ideological nature and getting institutions like schools to deal with risky topics like sex. Mighty Girls demonstrates a model for these processes by engaging administrators, teachers, as well as youth and parent in the process. Moreover, it demonstrates the promise and limitations of highly innovative technological approaches to prevention.

### S141 Implementing and disseminating REAL media through 4-h clubs and D.A.R.E.: Peer-to-peer delivery of messages through websites and social media

#### Kathryn Greene^1^, Anne Ray^2^, Michael Hecht^3^, Shannon Glenn^4^, Brandon Kramer^1^, Stephanie Pena-Alves^1^, Smita Banerjee^5^, HyeJeong Choi^6^, Rachel Lyons^1^, Michelle Miller-Day^4^

##### ^1^Rutgers University, New Brunswick, NJ, USA; ^2^Rutgers University School of Public Health, Piscataway, NJ, USA; ^3^REAL Prevention LLC, Gillette, NJ, USA; ^4^REAL Prevention, LLC, Clifton, NJ, USA; ^5^Memorial Sloan Kettering Cancer Center, New York, NY, USA; ^6^Health Sciences, Missouri, Columbia, MO, USA

###### **Correspondence:** Kathryn Greene (klgreene@rutgers.edu)

**Background:** In this media-saturated environment is becoming more and more important for youth to be “literate” about media influence particularly about topics related to public health. As a result, media literacy interventions have been developed, although with sometimes limited success. REAL media, however, is the exception both for its evidence base as well as for its potential for widespread uptake through engagement with key stakeholders. Build on the Theory of Active Involvement, REAL media is a brief, web-based curriculum that takes 13-17-year-old youth through understanding media influence to developing and disseminating their own substance use prevention messages. NREPP designate the curriculum as evidence-based prior to its termination. Recently, the 4-H clubs in 11 states have begun using the curriculum during a nationwide evaluation of this partnership.

**Methods:** REAL media began as a face-to-face curriculum that demonstrated efficacy when used in both schools and community groups. Adapted for online delivery, a partnership was formed with the 4-H clubs for widespread dissemination. Three group randomized trials demonstrate its effectiveness.

**Findings:** REAL media demonstrates effects on key substance use outcomes. In addition, the partnership model with 4-H has undergone a series of changes during the 2-plus years it has been in existence, demonstrating issues with widespread dissemination, community partnerships and engagement processes.

**Implications for D&I Research:** Media literacy prevention interventions have great promise, particularly when delivered through interactive websites. Technology, however, does not allow straightforward adaptation of face-to-face delivery methods. It has both opportunities/affordances as well as challenges. Moreover, youth have great promise for peer-to-peer dissemination of prevention messages, although not all youth-developed messages are equal in effectiveness. Overcoming these challenges, REAL media demonstrates the use of innovative strategies to improve accessibility, adopting, implementation and sustainability in scaling up evidence-based practices. At the same time, this project demonstrates the promise and limitations of highly innovative technological approaches to prevention.

### S142 Partnering with Planned Parenthood to present Women’s Stories: Using technology to delivery HPV vaccination decision narratives

#### Anne Ray (aer108@sph.rutgers.edu)

##### Rutgers University School of Public Health, Piscataway, NJ, USA

**Background:** HPV is a cause of cancer that exacts great costs. While an effective vaccine exists, take has lagged. To fill this void, Women’s Stories was developed for 18-26-year old women from their stories about decided whether to vaccinate or not. Using video docudramas delivered through laptops, the intervention dramatically increased vaccination and has been designated an NCI evidence-based intervention. Recently, the developers partnered with Planned Parenthood for clinic-based delivery of an expanded intervention

**Methods:** Decision narratives were collected from the target audience and scripted into video docudramas using narrative theory. An RCT was conducted to evaluate the intervention. A second project is underway to integrate Women’s Stories into the Planned Parenthood system through additional formative research to adapt the curriculum to African American, Latino, and Asian American culture, integrate it into the PP culture, and demonstrate usability.

**Findings:** The RCT showed the intervention almost doubled the uptake of the vaccine. Additional analyses identified a causal model. Additional formative research and usability studies have demonstrated the potential for widespread dissemination through Planned Parenthood, although this process has not been without obstacles and challenges.

**Implications for D&I Research:** Women’s Stories demonstrates the power of narratives for overcoming vaccine resistance and engagement hard-to-reach audiences. It also demonstrates the role of technology for taking health promotion to scale in clinics and other community-based organizations along with the attendant challenges of engagement the stakeholders in a politically charged and stressful environment like Planned Parenthood. Women’s Stories demonstrates the need for advanced implementation designs in public health as evidence-based prevention intervention are taken-to-scale as well as the essential nature of partnership through engaging key stakeholders.

## Promoting Health Equity and Eliminating Disparities

### S143 Trends in NCI Dissemination and Implementation Research: Analysis of Health Disparity/Minority Health Grants

#### Dajah Swinton^1^, Antoinette Percy-Laurry^2^, Tanya Agurs-Collins^2^, Gina Tesauro^2^

##### ^1^School of Public Health, University of Alabama at Birmingham, Birmingham, AL, USA; ^2^National Cancer Institute, National Institutes of Health, Rockville, MD, USA

###### **Correspondence:** Dajah Swinton (dswinton@uab.edu)

**Background:** Health disparities, though *preventable,* persist due to unfair policies and the unjust distribution of equitable health and social services. Furthermore, evidence-based interventions with the potential to ameliorate health disparities may stall or fail to reach intended populations due to: lack of awareness, low adoption and adaption rates, low cultural relevance, limited skill set in the field, lack of partnerships and capacity building, and other barriers. As a result, advancements toward health equity are slow which adversely impacts overall health. The purpose of this study is to examine trends of the National Cancer Institute’s (NCI) funded research opportunities in dissemination and implementation (D&I) research and their relation to health disparities and cancer prevention.

**Methods:** This work builds from an existing portfolio analysis conducted by members of the NCI Implementation Science team. They evaluated research funded by the NCI through the Dissemination and Implementation Research in Health Program Announcements, 2006-2018. They identified 34 grants (of 68 total) that addressed minority health/health disparities (MH/HD). We conducted an in-depth analysis of 32 coded health disparity related-grants. Since our research emphasized prevention, screening and survivorship across the cancer continuum, grants which focused on diagnosis and treatment were excluded (n=2). The sections appraised were: abstracts, biosketches, specific aims, research design, and targeted/planned enrollment table.

**Findings:** The composition of grants by population were 69% Multi-racial/ethnic groups, 13% Non-Hispanic Blacks, 9% Asian/American Indian and 9% Hispanic/Latino. Most interventions were adopted and adapted (n=29) according to the specified population of interest; several of which addressed barriers to access and participation. Health disparity related frameworks were not identified. However, most grants were culturally relevant and/or linguistically appropriate. Additionally, 23 research teams were multidisciplinary, five trans- and four inter-disciplinary. Finally, 30 grants engaged stakeholders, established linkages between researchers and key informants, or sustained the intervention through capacity building and partnerships.

**Implications for D&I Research:** D&I research is pertinent in eliminating health disparities. Researchers should focus more on influencing the uptake of evidence-based interventions in vulnerable populations including the usage use of health disparity frameworks. Although ongoing grants address MH/HD, more research is required for underserved populations to achieve health equity.

### S144 Mailed FIT program to increase colorectal cancer screening in two Medicaid/Medicare health plans: Learnings from first-year implementation

#### Laura-Mae Baldwin^1^, Jennifer Schneider^2^, Malaika Schwartz^3^, Jennifer Rivelli^4^, Beverly Green^5^, Jennifer Coury^6^, Amanda Petrik^2^, Gloria Coronado^2^

##### ^1^University of Washington, Institute of Translational Health Sciences, Seattle, WA, USA; ^2^Kaiser Permanente Center For Health Research, Portland, OR, USA; ^3^University of Washington Department of Family Medicine, Seattle, WA, USA; ^4^The Center for Health Research, Kaiser Permanente Northwest, Portland, OR, USA; ^5^Associate Investigator, Kaiser Permanente Washington, Seattle, WA, USA; ^6^CareOregon, Portland, OR, USA

###### **Correspondence:** Laura-Mae Baldwin (lmb@uw.edu)

**Background:** Timely colorectal cancer (CRC) screening is a critical prevention strategy that decreases mortality, yet CRC screening rates remain low, especially among certain racial/ethnic, uninsured and publicly insured groups. Clinics and health care systems have successfully used mailed fecal immunochemical testing (FIT) programs to raise CRC screening rates and address disparities, and health plans are beginning to adopt this approach to reach large-scale populations. Published guidance on successes and challenges to implementation of health plan-based programs is needed to support spread of these programs. Our team studied implementation of mailed FIT programs from the perspective of two health plans in Oregon and Washington state serving Medicare-Medicaid populations.

**Methods:** Guided by the domains of the Consolidated Framework for Implementation Research (CFIR), we interviewed all health plan leaders/staff (five per plan) instrumental in designing and executing the mailed FIT programs 6-9 months after implementation. Additionally, we explored implementation successes and challenges, enrollee and provider reaction/feedback, and observed strengths and weaknesses of each program’s implementation model. Interviewswere audio-recorded, transcribed, and content analyzed for themes. We stratified our analysis by state, since the Oregon’s health plan collaborated with clinics in administering the program, whereas Washington’s health plan ran its program centrally

**Findings:** Each state’s health plan tailored its mailed FIT program to its culture and resources. Both mailed FIT programs matched the health plan’s missions and goals, and the plans received positive feedback from patients and providers. Common challenges included a longer than expected time for program set-up and complexities in working with vendors for program functions (e.g., mailing FITs). Oregon’s collaborative model succeeded in engaging clinical staff and providers in promoting program success, but was challenged by individual clinic preferences, processes and capacity. Washington’s centralized model had a successful centralized tracking system and follow-up for positive FIT tests, but faced challenges in ensuring awareness of the program among other departments (e.g., membership services).

**Implications for D&I Research:** Documentation of mailed FIT program successes and challenges from health plans using two different implementation models can guide health plans in adapting these programs to their own culture and resources, and prepare them for potential obstacles.

### S145 Preliminary outcomes from a pilot health equity learning intervention in a national sample of cancer care organizations

#### Mandi Pratt-Chapman, Allison Harvey

##### George Washington University Cancer Center, Washington, DC, USA

###### **Correspondence:** Mandi Pratt-Chapman (mandi@gwu.edu)

**Background:** The Together, Equitable, Accessible, Meaningful (TEAM) Training was created to increase cancer care providers’ competence in providing affirming, equitable care to diverse patients. The pilot cohort participated in a hybrid training, including an online course, an in-person workshop, action planning and implementation, and virtual technical assistance from November 17 to March 2018.

**Methods:** Organizations applied to participate with documentation of institutional support. At six-month follow-up, each team was asked to complete a survey reporting contextual factors for action plan implementation success based on Golden's framework for organizational change. Two teams dropped out of the intervention, and 68% (n=15) of remaining teams completed the survey. Teams were categorized as high performers if they achieved substantial progress overall (n=7) and as low performers for minimal/some progress (n=8). The presence of eight implementation factors were assessed on a 5-point Likert scale (strongly disagree to strongly agree). High versus low performing teams were compared based on summed scores of implementation factors (lowest possible score=8; highest possible score=40).

**Findings:** Twenty-four organizations were selected, comprised of 2-4 members each (n=92). All teams reported being neutral (n=3), agreed (n=8) or strongly agreed (n=4) that the training provided the skills needed to implement their action plan. High versus low performers did not differ in terms of self-reported organizational culture and vision to implement their action plans, but differed on incentives to accomplish goals; access to organizational resources; access to organizational information; and sufficient time to advance action plans. High performers had greater cumulative scores for implementation factors present (M=32.29, SD=3.450) than low performers (M=27.63, SD=5.397).

**Implications for D&I Research:** These data support Golden’s framework for organizational change. While champions may have the skills and vision to improve health equity goals, findings suggest clear action plans, incentives, resources and time are critical to advance health equity goals in a care setting. Joint data displays will highlight implementation factors and qualitative feedback from the study.

**Primary Funding Source**

Pfizer Foundation

### S146 Adapting evidence-based physical activity interventions for cancer survivors and implementation in rural communities

#### Scherezade Mama^1^, Kathryn Schmitz^2^, Eugene Lengerich^2^

##### ^1^Pennsylvania State University, University Park, PA, USA; ^2^Penn State College of Medicine, Hershey, PA, USA

###### **Correspondence:** Scherezade Mama (skmama@psu.edu)

**Background:** Cancer survivors (CS) residing in nonmetro, medically underserved areas (MUA) are less likely to do physical activity (PA) than those residing in urban areas with low need, emphasizing the need for contextually tailored interventions designed to meet rural CS unique needs. The Partnering to Prevent and Control Cancer (PPCC) study used a community-engaged approach to understand and address PA disparities in rural CS in central Pennsylvania.

**Methods:** Community organizations were invited to join an academic-community partnership. Partnership members assisted with recruiting CS to PPCC via active (e.g., announcements at community events) and passive (e.g., mailings to cancer registries, community organizations) recruitment strategies. CS completed questionnaires assessing PA and barriers to and preferences for engaging in PA.

**Findings:** Forty-one community organizations were initially contacted to join the PPCC partnership, 15 expressed interest, and 10 returned signed letters of commitment. The partnership includes churches (*n*=4), cancer support groups (*n*=2), and other community organizations (e.g., YMCA, county/regional health organization; *n*=4). Through the partnership, 262 CS enrolled in the study (*n*=31 via active and 231 via passive recruitment methods), representing 17 counties and 86 cities in central Pennsylvania. Most participants were prostate (22.5%) or breast (22.1%) CS, followed by gynecological (15.7%), colorectal (8.8%) and lung (7.2%) CS, and 21.7% of participants reported multiple cancer diagnoses. Participants were mostly women (58.4%), in their mid-60s (*M* age=64.9±11.8 years), and overweight (*M* BMI=29.6±6.8 kg/m^2^). Only 28.3% of CS reported being sufficiently active, defined as doing enough strenuous-moderate PA to receive substantial benefits. Most (70.2%) participants reported that they enjoyed walking for PA, followed by do-it-yourself activities (49.2%), housework (40.8%), and gardening (34.4%). Most participants felt they would exercise more if they had an instructor to tell them what to do (44.8%), their doctor suggested they do PA (42.8%), and if they could exercise at home (45.1%). Participants reported 1-5 barriers to being active, including treatment-related side effects (e.g., fatigue, joint or back pain), bad weather, and schedule challenges.

**Implications for D&I Research:** Findings contribute to our understanding of the unique needs of CS residing in MUA in central Pennsylvania and will guide adaptation of evidence-based PA interventions for implementation within rural communities.

### S147 Informing the adaptation of a CHW model to facilitate lung cancer screening for Chinese taxi drivers

#### Jennifer Leng, Randall Li, Francesca Gany

##### Memorial Sloan Kettering Cancer Center, New York, NY, USA

###### **Correspondence:** Jennifer Leng (lengj@mskcc.org)

**Background:** The Chinese population is expected to become the largest immigrant group in New York City (NYC) in the next few years. In a national study, lifetime smoking prevalence among Chinese men was 42.5%. In our preliminary work among Chinese foreign-born male livery drivers in NYC, an alarming 73% were current or former smokers. Chinese drivers in NYC who smoke or smoked may be at exceptionally high risk for lung cancer due to the combined impact of tobacco use and air pollution exposure. The U.S. Preventive Services Task Force recommends annual lung cancer screening with low-dose computed tomography in adults aged 55-80 years with a 30 pack-year history and who currently smoke or quit within 15 years. A substantial body of research demonstrates that Community Health Workers (CHWs) have been effective at improving cancer screening rates among minority populations.

**Methods:** The Diffusion of Innovations theory (Diffusion Theory) describes the process of adopting innovations and factors that increase success. The present study is grounded in Diffusion Theory and qualitatively assesses the needs of Chinese livery drivers to inform a culturally and linguistically responsive adaptation of an existing health promotion intervention for taxi drivers, to facilitate lung cancer screening for eligible, high-risk Chinese drivers. We conducted in-depth interviews until saturation with 13 key Chinese-serving health professionals with a range of areas of expertise. Interviews were transcribed and analyzed using Atlas.ti.

**Findings:** Inductive analysis of the transcripts yielded 66 codes (subtopics) within 7 key themes: 1) Knowledge of Guidelines/Access to Screening, 2) Acceptability of CHW Program (Diffusion Theory), 3) CHW Role in Screening Process, 4) Qualities of an Ideal CHW, 5) Barriers to Facilitating Uptake of Lung Cancer Screening, 6) Challenges to Implementing a CHW Program, and 7) Adaptations to a CHW program for the Chinese Community.

**Implications for D&I Research:** Despite the known impact of CHWs on improving health outcomes, and efforts to integrate them into care, CHWs represent an innovation that has yet to be taken to scale. The adaptation and effective implementation of a CHW program for this extremely high risk group has the potential to have enormous impact, and to be highly sustainable and widely disseminable.

### S148 Adaptation of an effective cervical cancer screening intervention for African American women

#### Judith Lee Smith^1^, Erika Reed-Gross^2^, Erin Loomis^2^, Lumbe Davis^1^, Ingrid Hall^1^, Morenike Bello^2^, Jamila Jones^3^

##### ^1^Centers for Disease Control and Prevention, Atlanta, GA, USA; ^2^Westat, Atlanta, GA, USA; ^3^Community Guide, Centers for Disease Control and Prevention, Atlanta, GA, USA

###### **Correspondence:** Judith Lee Smith (JLeeSmith@cdc.gov)

**Background:** AMIGAS is a cervical cancer screening intervention delivered by community health workers (CHWs) grounded in behavioral theories and based on Community Preventive Services Task Force recommended strategies. A randomized controlled trial found that AMIGAS increased cervical cancer screening in Latinas and was cost-effective. To build on the success of this proven intervention, CDC recently adapted AMIGAS for African American women. African Americans were an appropriate target because of high cervical cancer incidence rates, poor screening uptake, parallel facilitators and barriers to cervical cancer screening, comparable acceptance of the CHW delivery model, and opportunities to demonstrate the application of the CDC Knowledge to Action framework.

**Methods:** We completed a multi-phase study designed to adhere to intervention fidelity. In Phase 1, we reviewed the literature to confirm and assess convergence and divergence in the barriers to and facilitators of cervical cancer screening among Latinas and African American women, acceptability of a CHW delivery model in the African American community, and findings regarding promotion of HPV testing. In Phase 2, we identified and recruited a national work group of experienced African American CHWs and CHW administrators (CHW-WG). In Phase 3, the CHW-WG provided guidance about the acceptability of the intervention delivery, components, messages, and images. The research team ensured compatibility of the modifications with theoretical foundations and scientific evidence. In Phase 4, we conducted focus groups to assess the revised intervention and refined materials and messaging in response to feedback received.

**Findings:** We have adapted AMIGAS for African American women. New messaging, images, and content in the Face Your Health (FYH) intervention address the unique concerns of the target population and changes in screening guidelines.

**Implications for D&I Research:** This adaptation underscores the importance of the interaction between research and translation phases of the CDC Knowledge to Action framework. FYH is primed for testing because we used theory, evidence, qualitative assessment, and input from end users to ensure the needs of practitioners and community members were central to its development. If successful in practice, FYH will provide public health practitioners with a useful tool to address a persistent cancer health disparity.

### S149 Translation, dissemination, and implementation with deaf communities to promote equity

#### Steven Barnett, Erika Sutter, Kelly Matthews, Lori DeWindt

##### University of Rochester, Rochester, NY, USA

###### **Correspondence:** Steven Barnett (steven_barnett@urmc.rochester.edu)

**Background:** Deaf American Sign Language (ASL) users comprise linguistic and cultural minority populations without access to most health information and programs. Few health systems have the knowledge and resources to develop and evaluate health interventions with Deaf ASL-users. Dissemination and implementation research is vital for health systems to include Deaf communities in population health initiatives.

**Methods:** We use a community-based participatory research (CBPR) approach to D&I research. Community members, researchers, clinicians, and other stakeholders worked together to identify community strengths and priority areas for research and intervention. We worked with others in the CDC Prevention Research Centers (PRC) Network to identify an evidence-based health promotion intervention (Weight Wise), and adapted/translated measures, methods, and materials to be communication accessible, language congruent, and culturally appropriate. We conducted a successful randomized trial of Deaf Weight Wise, an in-person group intervention to promote healthy lifestyle with adults ages 40-70. We subsequently adapted DWW for use with younger adults and translated the in-person group intervention for use 1-to-1 via videophone (like Skype), a modality that broadens reach, and began a second randomized trial (DWW-2.0). Qualitative data from participants and counselors identified key components of the DWW interventions; those findings informed intervention adaptation for use with another behavioral health condition (HIV risk).

**Findings:** With the initial DWW randomized trial (n=104), at 6-months the immediate group weight changed -3.35kg (1.0 s.e.; p=.002) compared with the delayed group (no intervention yet). Most of the immediate group (58.3%) lost ≥ 5% of baseline weight versus 14.3% of the delayed group (p≤.0001). Many participants in the immediate- and delayed-groups achieved persistent post-intervention weights that were ≥5% less than their baseline (45.8% and 44.6% at 24-months). Engagement was high -- 82% of participants consistently attended groups during the 16-week intervention, and 92% completed 24-month data collection. Preliminary results from the current DWW-2.0 trial are consistent with the first DWW trial. Qualitatively identified key components include: community-engagement, peer-delivery and support, culturally appropriate approach, language concordant information access.

**Implications for D&I Research:** Long-standing collaborations with Deaf communities were vital to our success. Centers of excellence may be an appropriate model for D&I research with Deaf and other minority populations.

### S150 Contextual factors influencing the implementation of culturally adapted evidence-based hypertension control strategies in Asian American-serving community sites in the New York/New Jersey area

#### Radhika Gore^1^, Shilpa Patel^1^, Catherine Choy^1^, MD Taher^1^, Mary Joy Garcia-Dia^2^, Hardayal Singh^3^, Sara Kim^4^, Sadia Mohaimin^1^, Ritu Dhar^1^, Areeg Naeem^1^, Simona Kwon^1^, Nadia Islam^1^

##### ^1^NYU School of Medicine, New York, NY, USA; ^2^Kalusugan Coalition, Woodside, NY, USA; ^3^UNITED SIKHS, New York, NY, USA; ^4^Korean Community Services of Metropolitan New York, New York, NY, USA

###### **Correspondence:** Radhika Gore (radhika.gore@nyumc.org)

**Background:** Hypertension affects a third of all Americans and is especially high among certain groups of Asian Americans. The Racial and Ethnic Approaches to Community Health for Asian AmeRicans (REACH FAR) project partners with community-based organizations to implement culturally adapted, evidence-based hypertension-related programs among Bangladeshi, Filipino, Korean, and Asian Indian communities in New York and New Jersey. The project introduced evidence-based strategies related to healthy food access and blood pressure screening and counseling in 26 sites: ethnic grocery stores and restaurants, and Muslim, Christian, and Sikh faith-based organizations. While culturally adapted programs have been evaluated for their effectiveness, knowledge of how contextual factors influence their implementation is limited.

**Methods:** We applied the Consolidated Framework for Implementation Research (CFIR) to identify factors that influenced the adoption, adaptation, and perceived sustainability of REACH FAR. Four CFIR categories were analyzed: intervention characteristics, inner setting, outer setting, and individuals’ characteristics. Fifteen semi-structured interviews were conducted with site leaders. Interviews were first coded for implementation outcomes and then re-read to identify influencing factors based on CFIR categories.

**Findings:** REACH FAR programs resonated in sites where leaders perceived a need to address unhealthy diet and lifestyles in their communities (*evidence* supporting intervention characteristics), and where sites had historically engaged in health programs and viewed this as part of their public-service mission (inner setting *culture*). Site leaders’ identification with this aspect of the organizational mission reinforced program adoption (individuals’ characteristics). Sites adapted programs to respond to community preferences (outer setting *needs*), but where community preferences conflicted with core program objectives, site leaders persisted with program implementation (inner setting *leadership engagement*). Leaders noted that staff turnover and insufficient volunteers (inner setting *resources*) can impede program sustainability, while other organizational resources, such as space, can be strategically utilized to enhance it. Leaders suggested reinforcing strategies throughout community networks (outer setting *cosmopolitanism*) to enhance sustainability.

**Implications for D&I Research:** Knowledge of social and organizational contextual factors that influence implementation of culturally adapted evidence-based interventions is needed to address racial/ethnic health disparities. Future culturally adapted programs should consider sites’ leadership, community reach, organizational culture, and resources as important factors shaping implementation.

### S151 Testing effectiveness after cultural adaptation, how much is necessary? a case study – from stepping on to pisando fuerte

#### Maria Mora Pinzon^1^, Elizabeth Jacobs^2^, Sherri Ohly^3^, Militza Bonet-Vázquez^4^, Marcia Villa^5^, Dayanira Rodriguez^5^, Al Castro^5^, Jane Mahoney^1^

##### ^1^University of Wisconsin, Madison, WI, USA; ^2^University of Texas at Austin, Austin, TX, USA; ^3^Wisconsin Department of Health Services, Milwaukee, WI, USA; ^4^Medical College of Wisconsin, Milwaukee, WI, USA; ^5^United Community Center, Milwaukee, WI, USA

###### **Correspondence:** Maria Mora Pinzon (mmora2@wisc.edu)

**Background:** There are no evidence-based fall prevention programs for Hispanic/Latinos even though their age-adjusted death rate from unintentional falls is climbing in the US. We describe the evaluation of “Pisando Fuerte”, a linguistically and culturally appropriate version for Spanish speakers of an evidence-based fall prevention program (“Stepping On” [SO]).

**Methods:** “Pisando Fuerte” consisted of 2-hour group sessions over the course of 8 weeks delivered in Spanish by trained Hispanic/Latino personnel in two communities in Wisconsin. Implementation and intervention fidelity were evaluated by an independent assessor on three sessions using a-priori criteria based on key elements of SO. Uptake, proximal and distal outcomes were assessed through interviews 6 months after the program.

**Findings:** 29 individuals were screened and 24 (82.8%) agreed to participate. Mean age was 70.5 years; 71% were female, five had reported a fall in the year prior to the program, 87.5% completed the program, and 18 (72%) participated in 6 months follow-up. Intervention fidelity was demonstrated (71% overall), however, deviations in protocol were noted in the use of adult learning strategies and advancement of exercises. At 6 months 57.9% of participants continued doing exercises, 94% reported changes in walking and standing, and 67% executed a home safety recommendation. There was an improvement of the Falls Behavioral Risk Scale (FaB) (2.69 vs. 3.16, p<0.001), and a not-statistical decrease in the self-reported number of falls per person in the 6 months post-intervention compared to 12 months pre-intervention [RR: 0.33 (95%CI: 0.096 – 1.13)]. These results are similar to those described for the English version of the program. One organizations is committed to adopt and sustain the program once it is available.

**Implications for D&I Research:** Our study shows that *“Pisando Fuerte”* is a feasible program to prevent falls among Hispanic/Latino communities, and shares similar benefits and challenges to SO. Further revisions to the leader manual and leader training are warranted to maximize fidelity. Applying our study results to the concepts of “Scaling-out”, described by Aarons et al (2017), we conclude that a large randomized clinical trial might not be required before proceeding to further packaging for dissemination to Spanish speaking communities.

### S152 What works when: Methods for authentic and continuous stakeholder engagement in research

#### Hillary Edwards^1^, Jennifer Huang^2^, Liz Jansky^2^, C. Daniel Mullins^1^

##### ^1^University of Maryland School of Pharmacy, Baltimore, MD, USA; ^2^Westat, Rockville, MD, USA

###### **Correspondence:** Hillary Edwards (hedwards@rx.umaryland.edu)

**Background:** Patients’ voices are critical to designing, conducting, and disseminating the results of Comparative Effectiveness Research (CER)/Patient-Centered Outcomes Research (PCOR) studies. There is limited guidance on when to use a particular method of stakeholder engagement and the estimate of time and resources required to have authentic engagement. We identify evidence-based engagement strategies to guide selection of the most appropriate, meaningful, and impactful engagement method for each step of PCOR.

**Methods:** The study was conducted in two phases: The environmental scan included systematic searches for information on stakeholder engagement methods in peer-reviewed literature, gray literature, and key guidance documents; Once we gathered evidence-based methods of engagement, we conducted interviews and focus groups with patients, other stakeholders and researchers. We included a card-mapping activity to identify methods corresponding to each stage of our 10-Step framework and evaluation. Following PRISMA guidelines, we identified 8,394 records through database searching., of which 165 articles were eligible for quantitative synthesis. In Phase II, we identified 50 patients, 56 stakeholders, and 50 researchers to participate in a one-hour focus group or interview. Stakeholders included community leaders, clinicians, payers, and vision-impaired participants.

**Findings:** Community partnerships are most meaningful in the planning and sharing stages of the research continuum. Stakeholders as research members is most meaningful when conducting research. Engagement promotes transparency between researcher and community, across all methods. Flexibility in scheduling and availability of the research team is important for patient engagement. Selecting familiar locations for in-person activities increases community engagement. Use a combination of methods: by showing the diversity within the study, community members are more likely to believe the results and conclusions drawn. Pre- and continuous engagement takes resources: Lay a framework for partnership before data collection; Provide resources/support for community before working on a study; Build a network of people engaged over time; Maintain a continuous presence in the community.

**Implications for D&I Research:** The PETT provides a roadmap for effective patient engagement throughout future PCOR studies so that results of future PCOR studies will be more informative to patients, caregivers, health care providers and the broader healthcare community.

### S153 Recognizing the perspectives of underserved multilingual and multicultural older adults: Learnings from community-based research on social isolation

#### Amanda Parrish^1^, Lesley Steinman^1^, Caitlin Mayotte^1^, Anu Orebiyi^2^, Carol Montoya^3^, Mark Snowden^4^

##### ^1^University of Washington, Seattle, WA, USA; ^2^Catholic Community Services of Western Washington, Seattle, WA, USA; ^3^Florida Health Networks, North Miami Beach, WA, USA; ^4^University of Washington, School of Medicine, Seattle, WA, USA

###### **Correspondence:** Amanda Parrish (parrisha@uw.edu)

**Background:** Social isolation is a topic of growing concern among older adults, practitioners, and policymakers because of the serious mental and physical health effects seen in all populations. Few social isolation interventions exist, so we are evaluating whether an existing evidence-based program (EBP) for late-life depression among low-income older adults (the Program to Encourage Active, Rewarding Lives (PEARLS)) can reduce social isolation. PEARLS empowers older adults with problem-solving and behavioral activation skills, and these skills can be applied to improve social connectedness. To promote greater health equity, we are engaging with community-based social service organizations (CBOs) to understand diverse perspectives from multilingual and multicultural older adults.

**Methods:** We are partnering with fifteen CBOs in five U.S. states to conduct a two-year concurrent triangulation mixed methods study to evaluate whether and how PEARLS can reduce social isolation (IRB exempt status). We purposively sampled CBOs that disseminate and implement PEARLS with underserved older populations including racial/ethnic minorities, rural residents, those with disabilities, and/or with limited English proficiency. PEARLS participants are surveyed and interviewed pre/post PEARLS using in-depth qualitative guides and validated social isolation measures (PROMIS, DSSI-10, UCLA Loneliness) in English, Cantonese, Mandarin, Vietnamese, Russian, and Spanish.

**Findings:** We will discuss challenges and opportunities of community-engaged implementation effectiveness research, with particular emphasis on strategies to reach culturally and linguistically isolated participants in culturally appropriate ways, collaborating with CBOs to reduce provider burden, balancing program fidelity and data generalizability with cultural sensitivity and responsiveness, and leveraging existing community partnerships to research a more culturally fluid outcome for an established EBP in low-resource settings.

**Implications for D&I Research:** *The Principles of Community Engagement* and the HPRC Dissemination Framework call for regular engagement between researchers and CBOs to better match EBPs and D&I strategies to individuals, organizations, and context. We have learned important lessons about strategies to diversify participation in D&I research in low-resource settings, with the goal of eliminating disparities in social isolation and its related health effects. To ensure equal access to needed social isolation interventions across cultural and linguistic groups, it is imperative that we do the work to include diverse perspectives in the adaptation and evaluations of EBPs.

### S154 North Carolina’s response to maternal opioid use: Mapping a KT intervention

#### Tracy Nichols, Meredith Gringle

##### University of North Carolina Greensboro, Greensboro, NC, USA

###### **Correspondence:** Tracy Nichols (trnicho2@uncg.edu)

**Background:** Increases in opioid use during pregnancy have fueled concerns among advocates, policy makers, and providers working in reproductive health. North Carolina initiated a response that included a knowledge transfer (KT) intervention targeting healthcare and social service providers to increase the use of evidence-based practices. Examining KT interventions as they unfold over time and across systems can help identify barriers and facilitators of knowledge uptake as well as reveal important contextual issues for implementing best practices. This presentation reports on a longitudinal case study of a state-level KT intervention focused on a marginalized population.

**Methods:** Qualitative data were used to examine a KT intervention that emerged from stakeholder concerns. Data were collected over a six-year period and include observations of KT activities (conferences, workshops, and stakeholder meetings), focus groups and interviews conducted with stakeholders, and a review of publicly available documents developed from the intervention. The intervention was mapped onto a theoretical framework detailing the Knowledge-to-Action process. From this a thick, rich description of the intervention was developed that captured its iterative nature. Additional analyses included applying Clarke’s situational mapping to identify important contextual factors.

**Findings:** North Carolina’s response to maternal opioid use was proactive and state-of-the-art. It included producing targeted and tailored toolkits and best practice guidelines as well as providing multiple knowledge exchange opportunities between researchers, advocates, and practitioners. Over time several coalitions and workgroups emerged to implement activities at the county level. Yet findings demonstrate stakeholders’ struggles with implicit biases against maternal drug use and ambivalence towards evidence-based recommendations of harm reduction principles. Tensions around knowledge interpretation and the appropriate implementation of best practices occurred between “experts” as well as among practitioners. Resource limitations, stigma, and perceptions of the mother-child dyad emerged as critical contextual issues.

**Implications for D&I Research:** This presentation describes a KT intervention targeting maternal opioid use. Findings highlight the complex and iterative nature of a state-level intervention applied across healthcare and social systems. Lessons to be learned include understanding the inherent limitations within a rational and cognitive approach to knowledge exchange and the need to systematically address stigma and bias in dissemination approaches for health issues that affect marginalized populations.

### S155 Methods to engage stakeholders from hard-to-reach communities to define PCOR priorities

#### Barbara J Turner, Natalia Rodriguez, Paula Winkler, Melissa A Valerio

##### UT Health San Antonio, San Antonio, TX, USA

###### **Correspondence:** Barbara J Turner (turner@uthscsa.edu)

**Background:** To reduce health disparities and improve outcomes, longitudinal engagement of stakeholders from vulnerable or hard-to-reach communities is a priority of patient-centered outcomes research (PCOR). Researchers have few practical guides for initiating an effective relationship with a community and maintaining it through productive, bilateral collaboration on research aligned with community priorities.

**Methods:** Through a PCORI-funded methods project, a multi-disciplinary team of researchers partnered with Texas A&M AgriLife Extension agents in two rural, South Texas counties to evaluate approaches for engaging stakeholders to identify research priorities to address a priority topic for the community. The 11 chapter guide resulting from this work is entitled: *Practical Methods for Community Engagement of Underserved Populations: Advancing Health, Engaging, and Developing Research (UP AHEAD*). It offers an overview of published evidence regarding approaches to establish sustainable partnerships with communities that have not benefited from research partnerships as well as practical lessons from our project which evaluated different non-probability sampling methods to recruit and learn about community-identified research priorities.

**Findings:** *UP AHEAD* shares implementable methods for establishing a dynamic, community-academic partnership along with lessons learned from our work. The guide features a rigorous overview of published evidence about the initial stages of learning about a community and establishing an advisory board of community partners who direct and inform the community engagement and research processes. It summarizes the evidence for a variety of community stakeholder recruitment and engagement methods as well as lessons learned from our real-worked engagement study working with rural, predominantly Hispanic communities to identify research priorities to improve the lives of persons with chronic pain.

**Implications for D&I Research:** *UP AHEAD* offers a summary of evidence-approaches for initial community engagement activities and practical implementation insights from a real-world project involving engagement of remote primarily Hispanic communities. It fills a gap for researchers who are looking to serve communities by developing trusting, equitable partnerships that lead to projects addressing community-identified priorities and needs. Importantly, this guide reflects the shared experience of researchers and community leaders as well as community stakeholder participants in the entire spectrum of community engagement activities.

### S156 Integrating a mental health innovation within the nurse family partnership program: How we developed the innovation with scale-up in mind

#### Linda Beeber^1^, Alasia Ledford^1^, Jennifer Leeman^1^, Paula Zeanah^2^, Sharon Sprinkle^3^, Mariarosa Gasbarro^4^, Joan Loch^5^

##### ^1^University of North Carolina School of Nursing, Chapel Hill, NC, USA; ^2^South Louisiana School of Nursing, Lafayette, LA, USA; ^3^Nurse Family Partnership, Greensboro, NC, USA; ^4^Prevention Research Center, University of Colorado, Aurora, CO, USA; ^5^Nurse Family Partnership, Scranton, PA, USA

###### **Correspondence:** Alasia Ledford (alasia@email.unc.edu)

**Background:** The Nurse Family Partnership® (NFP) is an evidenced-based nurse home visitation program that serves low-income women from pregnancy until their child’s second birthday and has been shown to improve health and other outcomes in both mothers and their children. In a 2009 survey, nurses reported that mothers’ mental health symptoms were a major barrier to NFP delivery and effectiveness. In response, NFP’s national service office partnered with mental health researchers to develop a mental health innovation to enhance nurse home visitors’ skills in addressing mental health symptoms. In this presentation, we describe the formative work done to develop the innovation, integrate it within NFP’s existing systems, and take it to scale nationwide.

**Methods:** NFP national, regional, and agency-level stakeholders were engaged in developing the innovation, which includes six online education modules and multiple downloadable tools that align with existing NFP care domains and link to other NFP resources. The innovation was piloted using a pre-test/post-test design in 57 agencies across four states. Quantitative surveys, focus groups, and interviews assessed the innovation’s acceptability, feasibility, and effectiveness at improving nurses’ mental health practice and self-efficacy. In January of 2018 the innovation was taken to scale in all 263 NFP agencies in 43 states, the US Virgin Islands, and five tribal communities.

**Findings:** Nurses rated the pilot innovation as acceptable and feasible, and reported increased self-efficacy (e.g., to use mental health screening tools [75% to 90%]) immediately following participation in the modules. Six months after participation, nurses increased the number of clients screened but reported a decrease in self-efficacy. To address this gap, we revised the innovation to include (1) tools that supervisors can use to reinforce skills in team meetings and (2) six monthly teleconferenced mental health expert consultations.

**Implications for D&I Research:** We describe the iterative, stakeholder-engaged formative work done to inform the integration of an innovation with the priorities, processes, and infrastructure of an organization with national reach to low-income mothers, thereby contributing to better outcomes for mothers and their children. This type of formative work is essential to maximizing the potential for an innovation to be taken to scale and sustained over time.

### S157 Providers’ perspectives on motivational interviewing for child weight with low-income Latino families: A mixed methods study in community health centers

#### Matthew Haemer, Suhong Tong, Richard Boles, Megan Morris, Russell Glasgow

##### University of Colorado School of Medicine, Aurora, CO, USA

###### **Correspondence:** Matthew Haemer (matthew.haemer@ucdenver.edu)

**Background:** Primary care providers (PCPs) report that low self-efficacy for counseling and insufficient time are barriers to addressing child obesity. Motivational Interviewing (MI) has been effective for weight counseling in controlled trials in higher SES populations. It is unknown how MI could feasibly be implemented and maintained in practices serving those at highest risk of obesity-related health disparities, including Latino families with low income.

**Methods:** As part of a pragmatic pilot trial of primary-care weight counseling, PCPs at safety-net clinics were offered five hours of MI training for healthy child weight divided over 2-3 months. Providers were trained in key MI skills using audit and feedback sessions with standardized patients in English and Spanish. A bilingual eHealth system to support screening and MI counseling was implemented during the training. Providers completed a validated survey of attitudes, beliefs, and practices before and after the training. Paired t-tests compared pre-post responses by survey domain. Focus groups 6 months after training explored providers’ subjective norms for counseling, attitudes toward MI, and intentions to continue counseling. Three coders identified themes using an inductive, grounded theory approach.

**Findings:** Thirty-two providers from four clinics completed a mean of 3.5 hours of training. Twenty-two (69%) completed both pre/post surveys; demographics did not differ from non-completers. Providers reported increased: confidence in the effectiveness of obesity counseling +13% (p=0.01), self-efficacy for counseling +26% (p=0.001), and lifestyle screening frequency +22% of baseline response range (p=0.008). Forty providers participated in 6 focus groups. Providers described benefits for time-management, patient relationships, and family engagement from the eHealth-MI protocol. These benefits facilitated adoption and maintenance. Some providers desired additional training to help low-income families to set lifestyle change goals, perceiving a socio-economic divide between provider and family.

**Implications for D&I Research**: The eHealth-supported bilingual MI protocol was feasible to implement and improved providers’ self-efficacy, outcome efficacy, and self-reported screening practice. Additional training for providers to support low-income families in setting goals may contribute to adoption and effectiveness. The findings support conduct of an implementation study on a scale that could confirm effectiveness of this MI protocol at lowering BMI in Latino children from families with low income.

### S158 Acceptability, appropriateness and appeal of implementing support for guideline-based cancer care in rural and minority urology practices

#### Shellie Ellis^1,2^, Mugur Geana^3^, Christine Mackay^1^, Ariel Shifter^2^, Jessie Gills^4^, Kelly Stratton^5^, Tomas Greibling^2^, Charles McWilliams^5^, Andrew Zganjar^6^, Brantley Thrasher^2^

##### ^1^University of Kansas Cancer Center, Fairway, KS, USA; ^2^University of Kansas School of Medicine, Kansas City, KS, USA; ^3^University of Kansas, Lawrence, KS, USA; ^4^Louisiana State University, Metairie, LA, USA; ^5^University of Oklahoma Health Science Center, Oklahoma City, OK, USA; ^6^University of Kansas Medical Center, Kansas City, KS, USA

###### **Correspondence:** Shellie Ellis (sellis4@kumc.edu)

**Background:** National guidelines recommend management of all cancer patients within a clinical trial when available. However, 20% of cancer in the US is urological and most often managed by community urologists who lack direct access to trials. These providers are more likely to treat rural and minority patients, potentially explaining lower rates of trial participation among these populations. We developed LEARN|INFORM|RECRUIT, a multi-modal intervention to support the referral of rural urological cancer patients to clinical trials. The acceptability, appropriateness and appeal of the intervention to practices serving other underserved populations is unknown.

**Methods:** We presented LEARN|INFORM|RECRUIT to urologists attending state urological professional society meetings serving Oklahoma, Kansas, Missouri and Louisiana in Spring 2018. We surveyed attendees to assess the intervention’s appropriateness and acceptability using Weiner’s four-item, validated scales: Acceptability of Intervention Measure and Intervention Appropriateness Measure. Participants assessed the intervention’s appeal using a novel measure of Attributes of Innovation Adoption. All items were assessed on a Likert scale. Appropriateness and acceptability responses were summed to create scale scores, ranging from 1 to 5 with higher scores indicating greater acceptability and appropriateness. Scores were averaged and t-tests used to compare those who do/do not offer clinical trials. Individual innovation attribute scores were averaged and ranked.

**Findings:** Across all sites, more than 50 urologists attended; 32 evaluated the intervention. Average acceptability and appropriateness ratings were 4.5 and 4.3, respectively. Scores did not differ between those who offering trials and those not (p=0.29 and p=0.72). Top-rated attributes included 1) helping the urologist match the right patient to the right treatment; 2) increasing the practice’s reputation as offering cutting edge treatment options; 3) helping the urologist adhere to practice guidelines; 4) making care more patient-centered; and 5) differentiating the urologists’ practice from other specialty practices.

**Implications for D&I Research:** A multi-modal intervention to support urologists’ referral to clinical trials is acceptable and appropriate to professionally engaged, community-practicing urologists in 3 predominately rural states and the state with the second largest African-American population. Future dissemination can highlight the intervention’s potential to differentiate participating practices and help urologists provide guideline-concordant, patient-centered care. Interventions to support guideline-based care appeal to practices serving underserved populations.

### S159 Development and implementation of EHR-based tools in a large NYC healthcare system to facilitate H. Pylori eradication strategies

#### Simona Kwon^1^, Yi-Ling Tan^1^, Janet Pan^1^, Devin Mann^2^, Sara Chokshi^1^, Renee Williams^2^, Qiuqu Zhao^2^, Anju Malieckal^2^, Karyn Singer^2^, Benyam Hailu^3^, Chau Trinh-Shevrin^1^

##### ^1^NYU School of Medicine, New York, NY, USA; ^2^NYU Langone Health, New York, NY, USA; ^3^National Institute on Minority Health and Health Disparities, National Institutes of Health, Bethesda, MD, USA

###### **Correspondence:** Yi-Ling Tan (yi-ling.tan@nyumc.org)

**Background:** Stomach cancer is the most common infection-related cancer worldwide. In the United States, Chinese Americans experience a disproportionate burden of stomach cancer mortality. The bacterium Helicobacter pylori (H. pylori) is the strongest risk factor for stomach cancer, with eradication of H. pylori through triple antibiotic therapy the most effective prevention method for stomach cancer. However, clinician adherence to the American College of Gastroenterology (ACG) guidelines on H. pylori treatment is not high. Medication adherence to the complex H. pylori treatment regimen is challenging, especially for Chinese New Yorkers for whom 61% have limited English proficiency and low health literacy. Working collaboratively with a coalition of community and health care provider stakeholders, we developed a health-systems level intervention using tools built for Epic, the electronic health record (EHR) software, to facilitate H. pylori treatment strategies.

**Methods:** The tool development process included 4 site workflow analyses, which consisted of ethnographic observation and key informant interviews with 5 providers who work with Chinese communities with limited English proficiency, to provide contextual data on organizational workflow, culture and practice. 15 key informant interviews with stakeholders from community-based organizations and former patients revealed the need for basic health education materials about H. pylori. Through iterative engagement of a transdisciplinary advisory group, we refined and implemented the EHR tools across an urban healthcare system which includes two hospitals and a network of Federally Qualified Health Centers based in community settings.

**Findings:** We developed 3 EHR-based tools: 1) a H. pylori medication order set for the most common first and second-line therapies; 2) culturally and literacy-appropriate basic health education materials for the patient in English and Chinese; and 3) a reminder for follow-up testing in 2 months, which is routed to the patient’s primary care physician. We will present barriers and facilitators to implementation, as well as findings from utilization reports on patterns of use.

**Implications for D&I Research:** There is a need to integrate system-wide EHR-based tools for under-served, vulnerable communities to enhance and sustain evidence-based practices for treatment adherence and cancer prevention and reduce H. pylori-related stomach cancer disparities for high-risk patient populations.

### S160 Sacred connections: Dissemination & implementation of a substance use intervention with native American youth via a university-tribal research partnership

#### Staci Morris^1^, Michelle Hospital^1^, Eric Wagner^1^, John Lowe^2^, Michelle Thompson^1^, Rachel Clarke^1^, Cheryl Riggs^2,3^

##### ^1^Florida International University, Miami, FL, USA; ^2^Florida State University, Tallahassee, FL, USA; ^3^University Of Arkansas - Fort Smith, Sallisaw, OK, USA

###### **Correspondence:** Staci Morris (morrisl@fiu.edu)

**Background:** Native American (NA) youth report higher rates of alcohol, marijuana and illicit drug use than U.S. adolescents from any other racial/ethnic group. This is a significant research priority across public health, health disparities, and dissemination and implementation (D&I) sciences, and underscores the need for empirically-based substance use interventions tailored for NA youth. Effective D&I incorporates NA cultural values and involves tribal elders and stakeholders as partners. To address these issues, this study established a university-tribal research partnership; SACRED Connections (NIDAR01DA02977) utilized a culturally derived Native-Reliance theoretical framework and a CBPR approach in development and implementation. A significant objective of this RCT was to close D&I gaps utilizing RE-AIM as a model with adherence to the National Standards for Culturally and Linguistically Appropriate Services in Health and Health Care (CLAS Standards) to: (1) disseminate information regarding the efficacy and advantages of this intervention among tribal members who were locally employed counselors, and (2) implement and evaluate the effectiveness of the developmentally and culturally tailored brief substance use intervention with NA youth.

**Methods:** The target population of this 5 year RCT included NA tribal community elders, counselors, and youth (N=405) (**R**EACH). The tribal community remained involved throughout the study and offered tribal IRB approval (**A**DOPTION). **I**MPLEMENTATION of a developmentally and NA culturally tailored brief protocol was successful. The study was expanded to additional schools, tribes, and NA counselors than were originally planned for (**M**AINTENANCE).

**Findings:** Select findings revealed a statistically significant protective relationship between Native-Reliance and baseline lifetime and past month alcohol and marijuana use; additionally, the likelihood of reporting marijuana use at 3 months post-intervention was significantly lower among the active condition than among the control condition (**E**FFECTIVENESS).

**Implications for D&I Research:** This study revealed: (1) partnering with Native Americans and utilizing CBPR facilitated effective engagement with this hard-to-reach and underserved community; (2) age and culture are important determinants in substance use severity among NA teens; (3) a culturally adapted MI-based brief intervention may be effective in reducing marijuana use among NA youth; (4) the Native-Reliance theory served as an appropriate guiding framework for working with this population; and (5) RE-AIM provided an effective conceptualizing D&I model.

### S161 Applying a structural competency framework to the implementation of strategies to reduce disparities for sexual and gender minority youth

#### Cathleen Willging^1^, Daniel Shattuck^1^, Amy Green^2^, Mary Ramos^3^

##### ^1^Behavioral Health Research Center of the Southwest, Pacific Institute for Research and Evaluation, Albuquerque, NM, USA; ^2^UC San Diego, La Jolla, CA, USA; ^3^University of New Mexico, Albuquerque, NM, USA

###### **Correspondence:** Cathleen Willging (cwillging@pire.org)

**Background:** Sexual and gender minority (SGM) youth are at higher risk for adverse health outcomes compared to cisgender, heterosexual peers. Safer school environments decrease this risk. The Centers for Disease Control and Prevention (CDC) recommends six evidence-based strategies that make schools safer for SGM youth, yet fewer than 12.2% of U.S. schools implement all of them. Efforts to close this implementation gap must attend to contextual issues influencing strategy uptake. Structural competency recognizes that structural forces (e.g., discrimination, community norms, institutional policies) produce disparities. We apply a structural-competency framework to elucidate factors affecting implementation of the CDC strategies to enhance SGM youth wellbeing in high schools across New Mexico.

**Methods:** We undertook and transcribed 81 qualitative interviews and 16 focus groups with school health professionals and administrators to assess factors impacting implementation of the CDC strategies. We also compiled anthropological fieldnotes within implementation coaching logs maintained by technical-assistance staff. We analyzed these textual data using iterative coding and thematic identification techniques. We then interpreted the themes in relation to the sensitizing concept of structural competency.

**Findings:** Six themes were identified: (1) rendering an invisible population visible through staff and student education; (2) encouraging critical thinking about SGM inequalities; (3) understanding intersections among religion, politics, and cultural conflicts undermining support for SGM youth; (4) building school personnel capacity to identify and address SGM student needs; (5) cultivating school environments *inclusive* of SGM students through structural innovations; and (6) tackling community-based sources of stigma and discrimination that contribute to harmful outcomes.

**Implications for D&I Research:** School personnel pointed to a dearth of community resources, calling for actions to facilitate SGM student access to formal and informal support systems and development of best-practice guidance documents. Much of what can be accomplished in schools is constrained by the invisibility of SGM students and underlying culturally-based biases and structural forces that perpetuate inequities. To reduce inequities for SGM youth, we must consider root causes rather than only intercede in the ways disparities are expressed. Applying a structural-competency framework to implementation science encourages concern for contextual factors that impact disparities and, in turn, improves the ability to implement interventions to reduce them.

### S162 Implementing routine HIV testing for adolescents in urban school-based health centers

#### Neal Hoffman^1^, Susanna Schneider Banks^2^, Sarah Overholt^3^, Stephanie Serafino^4^, Laurie Bauman^1^, Theo Sandfort^4^

##### ^1^Albert Einstein College of Medicine, Bronx, NY, USA; ^2^Montefiore School Health Program, Bronx, NY, USA; ^3^Montefiore Medical Center, Bronx, NY, USA; ^4^HIV Center for Clinical and Behavioral Studies, Columbia University, New York, NY, USA

###### **Correspondence:** Neal Hoffman (nhoffman@montefiore.org)

**Background:** HIV testing is a critical step in facilitating access to care and preventing onward transmission. New HIV infections are increasing among adolescents, while only a small proportion is tested. The CDC recommends routinely offering HIV testing in health care settings to persons 13 and over regardless of risk. With over 1,900 School-based Health Centers (SBHCs) in 45 US states, SBHCs provide a structural opportunity to offer adolescents HIV testing. We initiated an implementation project to set up routine HIV testing in SBHCs serving over 9000 adolescents in six public high school campuses in the Bronx, New York.

**Methods:** The intervention model included system-level initiatives (development of practice work flows and use of an Implementation Coach) and provider-level initiatives (didactic and small –group trainings and technical assistance to optimize use of electronic health record tools). Components included engaging staff in workflow development, providing HIV testing data, using performance improvement tools, and providing incentives for exceeding targets. We initiated workflows at the three SBHCs in Fall 2016 and at three additional campuses in Fall 2017. We focus here on preliminary lessons learned, guided by the Consolidated Framework for Implementation Research (CFIR). Logs were maintained for all individual and group contacts to describe different aspects of the implementation process.

**Findings:** Preliminary qualitative analysis suggests that most health center staff were supportive of routine HIV testing (Innovation Evidence Strength & Quality). However, competing clinical and operational priorities (Implementation Climate, Innovation Adaptability), staffing irregularities (Available Resources), and limited meeting/training time (Access to Knowledge & Information) served as barriers. Self-efficacy, individual stages of change (Individual Characteristics), and sense of ownership (Innovation Source) varied between health centers and associates. Consistent data feedback (Reflecting & Evaluating), staff engagement, and team-based incentives (Rewards) served to address these barriers. Initial findings indicate an increase in the proportion of adolescents seen at the SBHCs that received HIV testing.

**Implications for D&I Research:** Implementation activities to increase HIV testing within these six urban SBHCs demonstrate that adoption of new practices is a gradual process, requiring attention to various CFIR constructs at the innovation, individual, inner setting, and process levels.

### S163 Addressing occupational safety and health (OSH) inequities through implementation science: Lessons from a multilevel evaluation of a foundational curriculum for young workers

#### Rebecca J. Guerin^1^, Andrea H. Okun^1^, John P. Barile^2^

##### ^1^Centers for Disease Control and Prevention, Cincinnati, OH, USA; ^2^University of Hawai‘i at Mānoa, Honolulu, HI, USA

###### **Correspondence:** Rebecca J. Guerin (rguerin@cdc.gov)

**Background:** Work is an important, but overlooked, aspect of public health inequities research. Moreover, occupational safety and health (OSH) inequities experienced by adolescent workers are an enduring public health challenge. More than 80% of U.S. teens hold a job before completing high school. Work has benefits, but it also has risks. Adolescents in the United States aged 15-17 suffer more than twice the rate of serious injuries compared to adults over age 24. These incidents have a long-term impact on adolescent health. To reduce injury rates among teen workers, a *Healthy People 2020* goal, the National Institute for Occupational Safety and Health (NIOSH) and its partners developed a foundational curriculum, *Talking Safety*, that teaches teens critical competencies for safe and healthy work.

**Methods:** An intervention/evaluation study was conducted in 2015-2016 with approximately 1,700 eighth graders and 34 teachers across 30 middle schools in one of the largest U.S. school districts. Using a modified theory of planned behavior, we evaluated the effectiveness of *Talking Safety* to change students’ workplace safety and health knowledge, attitude, self-efficacy, and intention to engage in workplace safety activities. We also explored the impact of teacher implementation fidelity, the extent to which *Talking Safety* was delivered as designed, on student outcomes. Linking outcomes to implementation is essential for establishing evidence-based, public health interventions.

**Findings:** Post-intervention, students demonstrated statistically significant increases (*p* = .05) in mean scores across outcomes: workplace safety knowledge (32%); attitude (8%); self-efficacy (9%); and behavioral intention (9%). Multilevel analyses demonstrated gains (*p* < .001) in attitude, and self-efficacy were associated with gains in students’ intention to engage in workplace safety behaviors. Knowledge had indirect effects on intention. Teachers demonstrating higher levels of implementation fidelity had students who scored significantly higher (on average) on study measures.

**Implications for D&I Research:** Findings provide empirical support for teaching *Talking Safety* to teens and highlight the importance of implementation fidelity to achieving positive intervention outcomes. Building the evidence base for *Talking Safety*, a foundational curriculum that equips teens with knowledge and skills to prepare them for safe and healthy work, may contribute to the long-range goal of reducing OSH inequities experienced by this vulnerable population.

### S164 The power of a kmb network: Mobilizing the wisdom of youth and community to improve the mental health and well-being of young people

#### Lisa Lachance (Lisa.Lachance@dal.ca)

##### School of Social Work/Wisdom2Action, Dalhousie University, Halifax, NS, Canada

**Background:** Young people who experience social marginalization, adverse experiences, complex needs or multiple service use, experience mental illness at higher rates than their less vulnerable peers (Farmeret al., 2001; Newton et al., 2012). Young people may use formal clinical services to address mental health concerns but are also likely to require the services of non-profit organizations (NPOs) to address issues of housing, employment, education, recreation, and other social determinants of health, essential in supporting the well-being of young people (Ungar, Liebenberg, Dudding, Armstrong & van de Vijver, 2013). The quality of programs that youth access is critical to ensuring a positive impact on young people (Yohalem & Wilson-Ahlstrom, 2010). NPOs often have limited access to, and internal capacity to use, research or evaluation information, which means their programs do not benefit from emergent and evidence-based approaches (Mitchell, 2011). Over the past few years, there have been several efforts to overcome these internal and external barriers and ensure that NPOs are well placed to respond to the complex mental health needs of youth in Canada.

**Methods:** Wisdom2Action (wisdom2action.org) was funded by Canada's Networks of Centres of Excellence as a Knowledge Mobilization network from 2011-2018 with $2.6 million Cdn in funding. W2A uses the PARiHS framework to plan projects to gather, contextualize and facilitate the uptake of best and promising promises in the youth-serving sector and thus support the mental health and well-being of children and youth in challenging contexts.

**Findings:** This presentation will include examples of how to do KM in the informal youth-serving sector, including embedding youth engagement in practice, facilitating the use of evidence-based practice through organization-to-organization mentorship, knowledge sharing videos, innovation funding and more. Evaluation results from programs will be shared.

**Implications for D&I Research:** Community-based organizations are often excluded from implementing evidence-based interventions sue to a number of internal and external barriers. Yet, strengthening their programs is important for population-level health outcomes.

## Poster Slam

### S165 Asking relevant stakeholders: Providers and community health workers perceptions on scaling-up cervical cancer screening programs in the public health sector in India

#### Prajakta Adsul^1^, Purnima Madhivanan^2^

##### ^1^National Cancer Institute, National Institute of Health, Rockville, MD, USA; ^2^Florida International University, Miami, FL, USA

###### **Correspondence:** Prajakta Adsul (prajakta.adsul@nih.gov)

**Background:** Cervical cancer is the second most frequent cancer diagnosed among women in India and accounts to nearly 1/3rd of the global cervical cancer deaths. Screening using visual inspection methods presents an evidence-based intervention for reducing cancer related mortality. Consequently, India has recently launched a national program for cervical cancer prevention in the public-sector led primary health care settings. The goal of this study was to conduct a qualitative assessment of health system stakeholders that could influence the scale-up of cervical cancer screening programs in the public health systems.

**Methods:** We used the scaling-up framework for action proposed by Simmons and Shiffman and focused on understanding the attributes of the user organizations (primary care settings in the public health sector) from the perspectives of the providers and community health workers. To provide an in-depth understanding of the current situation, we chose a qualitative approach for this study. A total of 15 primary care providers were interviewed and 6 focus groups discussions were conducted with 35 community health workers; all working in the public health system in the Mysore district, India. All interviews were transcribed, and transcripts were analyzed with Atlas.ti, informed by a grounded theory approach, to identify emerging themes.

**Findings:** Both providers and healthcare workers perceived a strong need for cervical cancer screening services in their community however, neither group members reported being familiar with the screening tests for cervical cancer. In general, all providers reported that the existing public health care delivery system is setup for maternal and child care and cancer screening services have different objectives and functions which can be challenging for point of care providers and community health care workers. These challenges were mainly associated with promoting a culture of cancer prevention, motivating asymptomatic women, and providing financial support for screen positive women for diagnosis and treatment. When explained, almost half the stakeholders reported visual inspection methods would be acceptable to them and the women in their communities, and feasible to implement within their resource limited settings.

**Implications for D&I Research:** Provider-, patient-, and system-level perspectives need to be incorporated to generate an effective scale-up strategy.

### S166 Clinical champions: Five characteristics of effective change agents

#### Katie Allan^1^, Marisa Wetmore^1^, Erica Heisel^2^, Laura Damschroder^3^, Jane Forman^4^, Vanessa Dalton^1^, Michelle Moniz^1^

##### ^1^University of Michigan, Ann Arbor, Ann Arbor, MI, USA; ^2^Beaumont Hospital, Royal Oak, MI, USA; ^3^Implementation Pathways, LLC, Ann Arbor, MI, USA; ^4^VA Ann Arbor Healthcare System, Ann Arbor, MI, USA

###### **Correspondence:** Katie Allan (krallan@umich.edu)

**Background**: Implementing immediate postpartum LARC in maternity facilities is a complex process involving multiple departments, stakeholders, steps, and care settings (outpatient prenatal, inpatient intrapartum, outpatient postpartum). Prior work suggests that clinical champions are important, but little is known about the characteristics of individuals who successfully lead efforts to implement postpartum contraceptive care in hospital settings. There is need to distinguish the defining characteristics of champions from the implementation strategies they use, to better understand how champions exert effects on implementation (Saint 2010).

**Methods:** We conducted a systematic literature search to identify US maternity hospitals that conducted prospective cohort studies of inpatient contraceptive care. We purposively sampled academic hospitals with unique organizational or population characteristics. We conducted site visits with face-to-face, in-depth, semi-structured interviews with 66 key informants across 11 birthing facilities, using an interview guide informed by the Consolidated Framework for Implementation Research (CFIR). Interviews (lasting 11 to 61 minutes) were audio-recorded, transcribed, and analyzed using framework analysis and comparisons across sites to identify characteristics of champions associated with perceived implementation success.

**Findings:** Preliminary analysis revealed five defining characteristics of successful clinical champions: 1) expertise with the clinical innovation (e.g., serving on national workgroups or conducting personal research on the topic); 2) rich institutional knowledge (e.g., training at or working for many years at the implementing institution); 3) institutional power (e.g., serving in formal leadership roles and/or having the ability to leverage the support of proxy leaders); 4) persuasiveness (e.g., strong communication skills with ability to inspire stakeholders across organizational boundaries); and 5) propensity for detail-oriented systems-thinking (ability to see both the forest and the trees, anticipating effects both across the organization and in day-to-day clinical workflow).

**Implications for D&I Research:** Using a multiple case study of implementation processes in maternity hospitals, we identified five important baseline characteristics of clinical champions in maternity settings: prior experience with the innovation, institutional knowledge, institutional power (or proxy power), persuasiveness, and propensity for detail-oriented systems-thinking. These characteristics are distinct from the implementation strategies used by champions and may influence whether and how champions influence implementation outcomes. Further work is needed to see if our findings apply in other implementation settings.

### S167 Implementation of guideline-based care for sickle cell disease: Findings from a mixed methods needs assessment

#### Lisa DiMartino^1^, Joseph Telfair^2^, Cecilia Calhoun^3^, Lucia Rojas-Smith^4^, Kim Erwin^5^, Robert Gibson^6^, Jane Hankins^7^, Jana Hirschtick^8^, Danielle Hessler^9^, George Jackson^10^, Aimee James^11^, Julie Kanter^12^, Allison King^11^, Raymona Lawrence^13^, Sarah Norell^14^, Shannon Phillips^12^, Lynne Richardson^15^, Jena Simon^15^, Matthew Smeltzer^16^, Paula Tanabe^10^, Marsha Treadwell^17^

##### ^1^Research Triangle Institute, Research Triangle Park, NC, USA; ^2^College of Public Health, Georgia Southern University, Statesboro, GA, USA; ^3^Washington University, Saint Louis, MO, USA; ^4^RTI International, Washington, DC, USA; ^5^School of Design, University of Illinois at Chicago, Chicago, IL, USA; ^6^Georgia Regents University, North Augusta, SC, USA; ^7^St. Jude Children's Research Hospital, Memphis, TN, USA; ^8^Sinai Urban Health Institute, Chicago, IL, USA; ^9^UCSF School of Medicine, San Francisco, CA, USA; ^10^Duke University, Durham, NC, USA; ^11^Washington University School of Medicine, St Louis, MO, USA; ^12^Medical University of South Carolina, Charleston, SC, USA; ^13^Jiann-Ping Hsu College Of Public Health, Georgia Southern University, Statesboro, GA, USA; ^14^University of Illinois at Chicago, Chicago, IL, USA; ^15^Icahn School of Medicine at Mount Sinai, New York, NY, USA; ^16^School of Public Health, University of Memphis, Memphis, TN, USA; ^17^UCSF Benioff Children’s Hospital, Oakland, CA, USA

###### **Correspondence:** Lisa DiMartino (ldimartino@rti.org)

**Background:** Clinical practice guidelines recommend use of evidence-based interventions (EBIs) to improve quality of care for patients with sickle cell disease (SCD). However, these EBIs (e.g., opioids and hydroxyurea) are often underused. The Sickle Cell Disease Implementation Consortium (SCDIC), funded by the National Heart Lung and Blood Institute, aimed to translate data from a community-based needs assessment on barriers to using EBIs for SCD to inform development of implementation studies designed to improve care for persons with SCD.

**Methods:** Eight clinical sites of the SCDIC from across the U.S. conducted a needs assessment utilizing a mixed-methods approach designed to obtain diverse stakeholder perspectives (e.g., patients, primary care, emergency department (ED), and community providers) on barriers and facilitators to SCD care. Semi-structured interviews (n=169), focus groups (n=30), and surveys (n=920) elicited perceptions on care quality and access, and attitudes towards EBIs. Sites entered transcript data into a common matrix using codes identified deductively from interview guides. Codes were synthesized and thematically analyzed. Survey data were analyzed descriptively to complement qualitative findings and provide information on stakeholder demographics. Data were analyzed within and across clinical sites.

**Findings:** Surveys of providers highlighted stigma around SCD (35%), addiction concerns (47%), and the opioid epidemic (62%) as barriers to care. Likewise, in qualitative analyses both patients and providers reported patients were perceived as “drug seekers”, particularly in EDs. Other key barriers were: providers’ discomfort prescribing opioids and patients’ inability to travel to clinic. Multiple stakeholders also identified hydroxyurea side effects, lack of provider collaboration, and insurance issues as barriers to care. Facilitators were: access to ED pain protocols, health applications to support hydroxyurea use, availability of social workers/patient navigators, and community resources for transportation.

**Implications for D&I Research:** The needs assessment was a critical first step in the development of strategies for implementation of EBIs for SCD. These results will be used to inform development and testing of strategies related to care redesign, improving ED care, and linking unaffiliated individuals to SCD-specialty care. We anticipate findings will have the potential to contribute to a growing knowledge base of methods for addressing multilevel barriers to care by tailoring strategies to local clinical contexts.

### S168 Uptake of a smartphone app for self-assessment of functional capacity as the sixth vital sign: RE-AIM evaluation

#### Razan M. Fayyad, Ryan Shaw, Janet Bettger

##### Duke University, Durham, NC, USA

###### **Correspondence:** Razan M. Fayyad (razanfayyad@gmail.com)

**Background:** Walking speed—recently labeled the sixth vital sign for its ability to assess physical function and predict morbidity and mortality—can now be measured remotely on a smartphone, overcoming limitations of self-report and clinic-based assessments. The purpose of this study was to apply the RE-AIM framework to examine initial uptake of the 6th Vital Sign application (app) in order to plan clinical and population strategies for scaling up for broader use.

**Methods:** The 6th Vital Sign is a publicly available iPhone app designed using Apple's Researchkit to consent adults in a study that measures normal walking speed over 2 minutes (using the phone's accelerometer), self-reported health and sociodemographics. People without an iPhone could participate with someone else’s device via a personalized account. People outside the United States or unable to read English were ineligible. Each RE-AIM dimension (reach, effectiveness, adoption, implementation, and maintenance) was assessed independently by incorporating data from the app, American Community Survey, and iTunes.

**Findings:** Of the 6,459 people who visited iTunes to download the app, 1,072 consented to participate from 32 states. The study’s reach (completed walk test) included 671 adults (63%). People who consented but did not complete the walk test were younger than those who did (46.9 vs. 48.9 years, respectively), but the age distribution of walk test participants was comparable to the U.S. population for percent of people per decade of age. The proportion of male/female participants was similar between those who did/did not complete the walk test (58% female). Leading factors associated with individual adoption were referral (31%) and email notification of app availability (11%). Walking speed reliability and accuracy were tested against the gold-standard clinic assessment; 3 of 4 approaches to carrying the phone during testing (in hand in-front, hand swinging, bag/purse) resulted in no significant differences in walking speed. We identified 10.6% at risk of health decline (speed<1.0m/s), 9.5% continued to use the app to self-monitor, and 40% expressed interest in future related studies.

**Implications for D&I Research:** Remote assessment of functional capacity is feasible and reliable. Clinical referral could promote uptake and strategies to reach and mitigate individuals at risk of decline need to be identified.

### S169 Characterizing the process of adapting an early child obesity prevention intervention to address psychosocial stressors

#### Rachel Gross^1^, Kate Cuno^2^, Pamela Icochea Calenzani^2^, Tanisha Arnold^2^, Kimberly Tom^2^, Miguelina German^2^, Mary Jo Messito^1^, Alan Mendelsohn^1^

##### ^1^New York University School of Medicine, New York, NY, USA; ^2^Children’s Hospital at Montefiore, Bronx, NY, USA

###### **Correspondence:** Rachel Gross (Rachel.Gross@nyumc.org)

**Background:** The “Starting Early Program” is a comprehensive prenatal and pediatric primary care-based early child obesity prevention program, with demonstrated impacts on infant feeding, activity and growth trajectories. We designed adaptations to the program to address the diverse needs of low-income populations, with a focus on psychosocial stressors that can be barriers to engagement and impact. Characterizing the specific types of adaptations purposefully designed to address psychosocial stressors will aid in studying how they impact intervention effectiveness.

**Methods:** We conducted a pilot study to assess the feasibility of implementing the prenatal and early infancy sessions of the Starting Early obesity prevention program in a new primary care setting with an existing, evidence-based, low-cost, population-scalable pediatric platform called Healthy Steps, a parenting program that integrates behavioral health specialists in pediatric primary care. Mother-infant pairs received the intervention with three prenatal/postpartum individual counseling sessions and two nutrition and parenting support groups coordinated with pediatric visits at infant ages 1 and 2 months (5 sessions in total). Utilizing a comprehensive framework of classifying intervention adaptations (Stirman et al., 2013), we classified the specific types of adaptations based on whether they targeted the context or the content of the intervention.

**Findings:** 50 pregnant women were enrolled. Context adaptations included changes in: 1) format (the addition of a confidential texting service and electronic medical record system communication with providers); 2) personnel (program originally delivered by registered dietitians is now being co-delivered by a health educator and a psychologist); and 3) population (program specifically developed for low-income Hispanic families is now being delivered to more racially and ethnically diverse patients). Content adaptations included: 1) directly screening for psychosocial stressors and providing services as needed; and 2) adding curriculum about general parenting skills and early child development.

**Implications for D&I Research:** One challenge to understanding how adaptations enhance or hinder intervention impacts is the lack of attention to characterizing the different types of modifications that are planned. Exploring the relationships between context and content adaptations and later infant feeding, activity and growth outcomes will be critical to the scaling up of this adapted intervention.

### S170 Getting intimate partner violence screening implementation right: Identifying best clinical practices, implementation strategies and contextual factors for success

#### Katherine Iverson^1^, Omonyele Adjognon^2^, Grillo Alessandra^2^, Melissa Dichter^3^, Megan Gerber^4^, Cassidy Gutner^3^, Alison Hamilton^5^, Shannon Wiltsey Stirman^6^

##### ^1^National Center for PTSD, VA Boston Healthcare System, Veterans Health Administration, Boston, MA, USA; ^2^VA Boston Healthcare System, Center for Healthcare Organization and Implementation Research, Veterans Health Administration, Boston, MA, USA; ^3^Center for Health Equity Research and Promotion, U.S. Department of Veterans Affairs, Philadelphia, PA, USA; ^4^VA Boston Healthcare System, Veterans Health Administration, Boston, MA, USA; ^5^Department of Veterans Affairs, Health Services Research & Development Center for the Study of Healthcare Innovation, Implementation & Policy, Los Angeles, Veterans Health Administration, Los Angeles, CA, USA; ^6^Dissemination and Training Division, National Center for PTSD and Stanford University, Veterans Health Administration, Menlo Park, CA, USA

###### **Correspondence:** Katherine Iverson (katherine.iverson@va.gov)

**Background:** Women who experience intimate partner violence (IPV) are among the most vulnerable patients seen in primary care. The Veterans Health Administration (VA) recommends routine screening for IPV among women seen in primary care, but there is evidence of low uptake of IPV screening in VA. Our goal is to a) characterize best practices of IPV screening programs in VA, b) understand multi-level barriers to and facilitators of IPV screening program implementation, and c) identify context-sensitive implementation strategies to enhance implementation effectiveness of IPV screening programs in VA.

**Methods:** We conducted key stakeholder interviews with clinicians and administrators (n=32) at six high and five low adopting VA facilities. Facilities were identified based on performance measures from our partners in VA Women’s Health Services and the IPV Assistance Program. Key informants included women’s health medical directors, IPV coordinators, women’s clinic physicians, social workers, and psychologists. These phone recorded, individual, 60-minute, interviews were guided by the integrated-Promoting Action on Research Implementation in Health Services (i-PARIHS) framework. Interviews were transcribed, coded, and analyzed both at the facility level and across facilities to identify best practices, barriers and facilitators and implementation strategies of IPV screening and response.

**Findings:** Findings across facilities identify a) 4 best clinical practices (the 5-item *E-HITS* screening tool, the 3-item danger assessment to triage patients at highest risk to immediate resources, brief counseling that includes IPV education and referral options, and ‘warm handoffs’ for needs assessment and counseling, b) 3 key implementation strategies (an engaged and highly visible champion such as an IPV Coordinator, ongoing training of staff and providers, learning collaborative/network, on-site consultation with clinicians expert in IPV and psychosocial health issues), and c) several contextual factors (e.g., leadership support, facility’s culture/environment, resources, unclear and inconsistent policy guidance, etc.) affecting facilities’ effective IPV screening implementation.

**Implications for D&I Research:** Knowledge of best clinical practices, successful implementation strategies and an understanding of contextual barriers and facilitators are critical to effective implementation processes and long-term health outcomes for women, in and outside the VA. These findings inform context-sensitive implementation strategies to scale-up IPV screening programs in VA and scale-out IPV screening programs to other health care settings.

### S171 Strategies for full system scale and spread: A systematic review

#### Isomi Miake-Lye^1,2^, Selene Mak^1,2^, Christine Lam^3,4^, Anne Lambert-Kerzner^5^, Deborah Delevan^1^, Pamela Secada^1^, Paul Shekelle^6^

##### ^1^VA Greater Los Angeles Healthcare System, Veterans Health Administration, Los Angeles, CA, USA; ^2^UCLA Fielding School of Public Health, Los Angeles, CA, USA; ^3^Department of Medicine, UCLA, Los Angeles, CA, USA; ^4^VA Greater Los Angeles Healthcare System, Veterans Health Administration, Sepulveda, CA, USA; ^5^University of Colorado - Denver, Denver, CO, USA; ^6^Veterans Administration, Los Angeles, CA, USA

###### **Correspondence:** Selene Mak (selenemak@yahoo.com)

**Background:** While innovations and improvements in care delivery are continuously available, they are often not spread across all settings that would benefit from their uptake. This systematic review seeks to describe what strategies have been used to scale up and spread clinical and administrative practices across multi-site healthcare systems, with special attention paid to sites with poor performance or that may be hard to engage in improvement initiatives. These include macro level strategies, such as learning health system infrastructure, as well as micro strategies such as coaching or education of individuals.

**Methods:** We searched for literature in multiple databases using terms related to “scale and spread” and “learning health systems”. We also identified publications from relevant projects in a VA-specific database. Publications are excluded if they are not relevant to healthcare delivery settings, discuss spread in low income countries, discuss spread in a theoretical way without specific examples, or include a limited roll out (less than ten sites) that does not specifically include hard to engage or low performing sites. The data synthesis is narrative.

**Findings:** Our searches identified 1,919 titles for review. Of these, 247 full text articles were screened and 65 articles were eligible for inclusion. Three models for spread identified at the macro level include (1) Collaborative or exchange to support spread of multiple initiatives within a specific topic area, like pediatric rheumatic diseases; (2) initiative-specific spread, which describes the uptake of a specific innovation across a large number of unaffiliated sites, such as the national spread of telemedicine in Norway; and (3) embedded spread within a system, like Kaiser Permanente Northern California and Geisinger, which have systems that spread related to high priorities within their institutions. Common micro strategies such as peer-to-peer engagement and multi-level stakeholder conversations were employed across models.

**Implications for D&I Research:** Findings suggest that there are several macro models for spread that describe different overarching strategies or objectives, and may employ similar micro strategies. Little evidence has been identified that focuses on or provides discussion of strategies for reaching those sites that may be harder to engage or that have particularly low performance.

### S172 Patient feedback regarding an improved method of incorporating racial/ethnic minority patients’ treatment preferences into clinical care

#### Ana Progovac^1,2^, Dharma Cortés^2,3^, Esther Lee^2^, Selma de Castro^2^, Maria Jose Sanchez^2^, Natasha Kaushal^4^, Rajan Sonik^2^, Timothy Creedon^2,5,6^, Tali Flomenhoft^7^, Jonathan Delman^8^, Deborah Delman^9^, Sherry Hou^10^, Valeria Chambers^9^, Catherine Rodriguez Quinerly^9^, Ziva Mann^2^, Danny McCormick^1,2^, Ruth Nabisere^2^, Farah Shaikh^2^, Dierdre Jordan^2^, Afsaneh Moradi^2^, Heba Abolaban^2^, Adam Carle^11^, Nicholas Carson^1,2^, Susan Busch^12^, Michael Flores^1,2^, Frederick Lu^2^, Benjamin Cook^1,2^

##### ^1^Harvard Medical School, Boston, MA, USA; ^2^Cambridge Health Alliance, Cambridge, MA, USA; ^3^Institute on Urban Health Research and Practice, Northeastern University, Boston, MA, USA; ^4^Boston University, Boston, MA, USA; ^5^Heller School for Social Policy and Management, Brandeis University, Waltham, MA, USA; ^6^IBM Watson Health, Cambridge, MA, USA; ^7^Brandeis University, Waltham, MA, USA; ^8^Reservoir Consulting Group, Boston, MA, USA; ^9^The Transformation Center, Roxbury, MA, USA; ^10^Acumen, LLC, Burlingame, CA, USA; ^11^Cincinnati Children's Hospital Medical Center, Cincinnati, OH, USA; ^12^Yale University, New Haven, CT, USA

###### **Correspondence:** Ana Progovac (aprogovac@cha.harvard.edu)

**Background:** A nationally representative community-partnered survey of 1512 people with depression and/or diabetes (including 503 non-Hispanic white, 505 non-Hispanic Black, and 504 Hispanic participants) found racial/ethnic minorities experienced healthcare-based discrimination (or knew someone who had) 2-10 times more often than white participants and that experiencing discrimination shifted treatment feature preferences (assessed via Conjoint Analysis). We sought to understand how healthcare-based discrimination impacted future treatment preferences, as well as assess participant feedback regarding acceptability, appropriateness, and feasibility of implementing the survey clinically.

**Methods:** We conducted 40 45-minute semi-structured interviews with survey respondents to characterize how discrimination experiences impacted treatment, and to assess receptiveness to using the survey in clinical settings. Interviews were transcribed, coded by a team of 6 independent coders (2 per interview), consolidated, and themes were extracted using a combination of a-priori themes and grounded theory.

**Findings:** Discrimination experiences included race-based assumptions about: sexual activity and risk, ability to perform health behaviors, and propensity to abuse medications/illicit substances. Participants recalled feeling offended, leaving the office, delaying needed care, and/or avoiding bringing up specific health issues, but rarely reported the experience to the clinic. These experiences resulted in poorer therapeutic alliance; neglect or misdiagnosis; delay/avoidance of needed care, and decreased patient satisfaction. Participants thought the survey could help patients advocate for themselves during clinical appointment, and would be comfortable completing it in real-world clinical settings, particularly prior to an appointment.

**Implications for D&I Research:** Survey participants who experienced discrimination elucidated the ways in which discrimination experiences disrupt quality patient care and could contribute to disparities in depression and diabetes care, particularly for minority patients. Participants overall felt the survey and Conjoint Analysis elicitation method would be acceptable and appropriate, and provided suggestions to increase feasibility for patients. Our next step is combining quantitative survey results and qualitative participant responses to assess provider and healthcare administrator feedback regarding barriers and facilitators to implementing the survey in primary care clinical settings. This project showcases an effective mixed-methods community-research partnership spanning from survey development to pre-implementation outcomes assessment, with a goal of reducing racial/ethnic disparities in patient care for chronic physical and behavioral health conditions.

### S173 Scaling up advanced care coordination to improve transitions of care and longitudinal care coordination for dual use veterans

#### Heidi Sjoberg^1^, Roman Ayele^2^, Marina McCreight^2^, Ashlea Mayberry^3^, Courtney Bauers^1^, Lynette Kelley^1^, Rachel Johnson^1^, Lindsay Miller^1^, Catherine Battaglia^4^

##### ^1^Denver Seattle Center of Innovation for Veteran-Centered and Value Driven Care, Veterans Health Administration, Aurora, CO, USA; ^2^Denver/Seattle Center of Innovation, VA Eastern Colorado Healthcare System, Veterans Health Administration, Aurora, CO, USA; ^3^Denver/Seattle Center of Innovation, Denver VA Medical Center, Veterans Health Administration, Aurora, CO, USA; ^4^Denver-Seattle Center of Innovation (COIN), VA Eastern Colorado Healthcare System, Veterans Health Administration, Denver, CO, USA

###### **Correspondence:** Heidi Sjoberg (heidi.sjoberg@va.gov)

**Background:** Veterans with complex healthcare needs utilize both the Veterans Administration (VA) and community hospitals. These dual-use Veterans require longitudinal care coordination that addresses social determinants of health (SDOH). VA is inconsistently notified when Veterans access community emergency departments (ED), causing fragmented care and adverse outcomes. We collaborated with VA’s Patient Aligned Care Team (PACT) to scale up our Advanced Care Coordination (ACC) program, a social worker driven intervention that focuses on improving longitudinal care coordination for dual-use Veterans who access community EDs.

**Methods:** ACC includes four core components: 1) Community ED notifies ACC of a Veteran’s visit, 2) Completing the national Social Work Comprehensive Assessment, which we identified as the optimal screening tool for individualized interventions, 3) Implementing interventions that address SDOH by scheduling mental health and PACT appointments and linking Veterans to resources, and 4) Transferring care to the Veteran’s PACT and coordinating care by mailing Veterans cards with their PCP’s information. These components enhance longitudinal care coordination by improving collaboration and communication across healthcare systems. To scale up ACC we engaged internal and external stakeholders by disseminating educational/outreach materials outlining ACC’s services and conducting meetings. We created a data collection tool to track intervention effectiveness and measure outcomes through the RE-AIM framework.

**Findings:** We conducted 10 educational meetings at VA and 3 community EDs and received 56 Veteran referrals over 4 months. Our social worker completed clinical and SDOH assessments using the screening tool to identify interventions that provide longitudinal care coordination. Seventy-one interventions were delivered through home visits and phone calls. We learned these interventions provided quicker access to PCP appointments, mental health care, and linkage to resources. On average our Veterans’ acuity levels required supportive/moderate services. The average length of enrollment was 5 weeks. We learned calling, emailing, and regular meetings enhanced stakeholder engagement.

**Implications for D&I Research:** Recognizing the importance of longitudinal care coordination based on the SDOH is necessary for effective care coordination across healthcare systems. For successful scaling up of clinical interventions, continuous multi-level stakeholders’ engagement is imperative. Lessons learned from ACC will guide the use of actionable data to improve patient care, reduce unnecessary costs, and enhance sustainability.

### S174 Using a systems-based participatory approach to explore the multi-sector impact of housing for health, an implementation model for permanent supportive housing

#### Irene Vidyanti^1^, Ricardo Basurto-Davila^2^, Emily Caesar^1^, William Nicholas^1^, Faith Washburn^1^

##### ^1^Center for Health Impact Evaluation, Los Angeles County Department of Public Health, Los Angeles, CA, USA; ^2^Analytics Center of Excellence, Los Angeles County Chief Executive Office, Los Angeles, CA, USA

###### **Correspondence:** Irene Vidyanti (ividyanti@ph.lacounty.gov)

**Background:** Permanent supportive housing (PSH) is an evidence-based approach that provides chronically homeless individuals with housing, case management, and voluntary supportive services. PSH implementation can suffer from a lack of coordination given the complexity in operationalizing its multiple components. Housing for Health (HFH) is a Los Angeles County initiative that seeks to improve PSH implementation by consolidating the management of PSH elements. Given the range of services and sectors involved, PSH stakeholders often have cognitive models that only partially represent the complex system in which PSH is embedded. Participatory modeling, a systems science methodology, can be used to facilitate the alignment and integration of these partial cognitive models into a holistic system description. Our purpose was to achieve a better understanding of how a more effective implementation model can impact individual and system-level outcomes.

**Methods:** We used a participatory systems modeling approach to develop a causal loop diagram of the pathways of chronically homeless populations across multiple sectors (housing, health, social services, and criminal justice). We convened stakeholders representing these sectors and split them into sector-specific breakout groups facilitated by research team members. After the convening, we synthesized sector-specific diagrams into a single causal loop diagram. We then used the resulting simulation model to generate insights into the cross-sector impact of PSH in general, and the HFH implementation approach in particular.

**Findings:** The participatory model revealed several reinforcing mechanisms (vicious cycles) that—if left unchecked by an intervention such as PSH—lead to increasingly worse outcomes and higher burden on the system, particularly the health and criminal justice sectors. The model also illuminated how individual PSH components —housing placement and supportive services— can work in concert to help the chronically homeless stabilize their situation and manage their complex chronic conditions. Better service coordination through the HFH approach facilitates provision of housing and supportive services to more effectively achieve the goals of PSH.

**Implications for D&I Research:** Systems science methods can increase our understanding of the systemic impact of complex interventions that span multiple sectors, such as PSH. Moreover, these methods can help identify implementation elements that need to be supported to improve effectiveness and ensure system sustainability.

